# Global Patterns of the Fungal Pathogen *Batrachochytrium dendrobatidis* Support Conservation Urgency

**DOI:** 10.3389/fvets.2021.685877

**Published:** 2021-07-16

**Authors:** Deanna H. Olson, Kathryn L. Ronnenberg, Caroline K. Glidden, Kelly R. Christiansen, Andrew R. Blaustein

**Affiliations:** ^1^Pacific Northwest Research Station, United States Department of Agriculture (USDA) Forest Service, Corvallis, OR, United States; ^2^Department of Biology, Stanford University, Stanford, CA, United States; ^3^Department of Integrative Biology, Oregon State University, Corvallis, OR, United States

**Keywords:** amphibian chytrid, *Bd*, climate associations, emerging infectious disease, fungal pathogen

## Abstract

The amphibian chytrid fungus *Batrachochytrium dendrobatidis* (*Bd*) is a skin pathogen that can cause the emerging infectious disease chytridiomycosis in susceptible species. It has been considered one of the most severe threats to amphibian biodiversity. We aimed to provide an updated compilation of global *Bd* occurrences by host taxon and geography, and with the larger global *Bd* dataset we reanalyzed *Bd* associations with environmental metrics at the world and regional scales. We also compared our *Bd* data compilation with a recent independent assessment to provide a more comprehensive count of species and countries with *Bd* occurrences. *Bd* has been detected in 1,375 of 2,525 (55%) species sampled, more than doubling known species infections since 2013. *Bd* occurrence is known from 93 of 134 (69%) countries at this writing; this compares to known occurrences in 56 of 82 (68%) countries in 2013. Climate-niche space is highly associated with *Bd* detection, with different climate metrics emerging as key predictors of *Bd* occurrence at regional scales; this warrants further assessment relative to climate-change projections. The accretion of *Bd* occurrence reports points to the common aims of worldwide investigators to understand the conservation concerns for amphibian biodiversity in the face of potential disease threat. Renewed calls for better mitigation of amphibian disease threats resonate across continents with amphibians, especially outside Asia. As *Bd* appears to be able to infect about half of amphibian taxa and sites, there is considerable room for biosecurity actions to forestall its spread using both bottom-up community-run efforts and top-down national-to-international policies. Conservation safeguards for sensitive species and biodiversity refugia are continuing priorities.

## Introduction

The Earth is undergoing a “biodiversity crisis,” with population losses and species extinctions occurring at unprecedented rates (1–6). Contributing factors to biodiversity losses are multifaceted and are complicated by species, population- and site-specific differences. Anthropogenic stressors such as habitat loss and fragmentation, chemical contamination, introduced species, and climate change are key factors influencing losses across taxonomic groups. Furthermore, there is increasing recognition of health concerns as species are exposed to emerging infectious diseases (EIDs) [e.g., coral disease outbreaks (7, 8); sea star wasting disease (9); bat white-nose syndrome (10, 11); avian West Nile virus (12); >50 United States (US) wildlife disease factsheets (13)].

For the especially vulnerable vertebrate class Amphibia, the Global Amphibian Assessment first reported 32.5% of species as threatened with extinction (14) and that estimate has since risen to 40% of species (5, 6), with an increase in concern for disease impacts (2, 15–20). Amphibian diseases span both lethal and sublethal multiple-host species infections by microparasites such as trematodes (21), bacteria (22), fungi (15, 23, 24), protists (25, 26), and viruses (16, 24). Information on amphibian disease-causing pathogens has increased substantially in the last two decades, especially relative to field surveillance of taxonomic and geographic patterns of pathogen occurrences at global scales (27–31) and experimental research that illustrates species-specific vulnerabilities and interacting factors (24, 32). Keeping informed about rapid advances in research and monitoring of amphibian diseases is challenging. Further complicating the challenge of tracking host-pathogen patterns of disease threats, anthropogenic processes are linked with amphibian disease dynamics. For example, human-mediated translocation of amphibian EIDs is an increasing concern, especially for chytridiomycosis, the disease caused by the chytrid fungal pathogens *Batrachochytrium dendrobatidis* (*Bd*) and *B. salamandrivorans* (*Bsal*). These two pathogens are associated with amphibian infections across continents and disease-caused mortality resulting in population losses (14, 17–20, 33, 34).

Amphibian chytridiomycosis research has transitioned from initial pathogen identification associated with amphibian mortality [*Bd* (35); *Bsal* (23)] to understanding pathogen occurrences and patterns of amphibian losses as knowledge of host-susceptibility, pathogen strain virulence, and transmission scenarios has unfolded (15, 17, 19, 24, 33, 34). Several geographic origins of *Bd* have been proposed, spanning Asia, Africa, and North and South America (36–40). A recent genetic analysis reported east Asia to be a *Bd* biodiversity hotspot, where the source of *Bd* was traced to the Korean Peninsula and one lineage showed the signature of an ancestral population tied to global emergence in the early twentieth century (34). Scheele et al. (17) estimated that *Bd* chytridiomycosis has contributed to the declines of 6.5% of amphibian species, and categorized *Bd* as one of the most destructive invasive species. Lambert et al. (18) concurred that *Bd* chytridiomycosis irrefutably harmed amphibians but because their re-analysis could not reproduce the specific results of Scheele et al. (17), they called for a more comprehensive approach to quantify the complexities of interacting amphibian threat factors.

The globalization of amphibian diseases and increasing need for both researchers and natural-resource stewards to understand EIDs and their incremental science advances has been aided by the advent of amphibian pathogen databases at world-accessible web portals. The Global *Bd* Mapping Project began in 2007, with its database of *Bd* occurrences by host taxon and geographic location going online at the web portal *Bd*-Maps.net in 2008, hosted by Imperial College, UK. This exportable database and mapping application provided the first global-scale visualization of an amphibian panzootic (19, 28). The broad use of *Bd*-Maps.net led to the development of an analogous but more comprehensive online database for Ranavirus, the Global Ranavirus Reporting System [GRRS (31)]. The GRRS inspired the development of the more sophisticated amphibian chytrid disease portal for both *Bd* and *Bsal*, AmphibianDisease.org, hosted by AmphibiaWeb and the University of California at Berkeley (41). The *Bd*-Maps.net dataset is currently in transition to AmphibianDisease.org, providing continuity of and a means to archive the Global *Bd* Mapping project from 2007 to present.

The importance of globally accessible online databases for discerning pathogen occurrence patterns is multifold. First, gaps in knowledge are readily apparent by species and location and can guide subsequent inventory and monitoring efforts; there is support that taxonomic and geographic gaps have been filled over time (28, 42, 43). Second, world occurrence maps of amphibian pathogens [e.g., *Bd* (28, 42, 43)] have been widely used for education and outreach across disciplines, raising awareness of potential emerging threat factors and informing conservation efforts. For example, global *Bd* maps have appeared in textbooks (44), museum and zoo exhibits (e.g., Panama exhibit by Smithsonian Institution; US National Zoo exhibit, Washington, DC; *Fungi and Their Diversity* exhibit, Hesse Museum, Wiesbaden, Germany), and other multimedia venues [(45, 46); e.g., ArgoFilms 2009 film for the Public Broadcasting System's *Nature* TV show, *Frogs: The Thin Green Line*]. Third, knowledge of pathogen occurrences can inform biosecurity procedures to forestall human-mediated translocation (47–49). In addition, global datasets can enable novel metadata analyses of specific hypotheses; the global *Bd* database has contributed to a variety of analyses of host-pathogen and disease-threat dynamics [e.g., (50–53)]. However, the initial *Bd* online database had some constraints. Occurrences of the disease chytridiomycosis were not tracked, as *Bd* occurrence studies often do not report the development of disease signs in sampled animals. Additionally, we now understand that disease emergence varies with *Bd* strain (34, 54), and as of this writing, the *Bd* database has not recorded *Bd* lineages with surveillance data, nor have most published sources isolated or reported the strain(s) surveyed. Furthermore, the initial *Bd* database was not set up to report zoospore loads for samples—these were not being reported in 2007, and even today, not all publications report *Bd* zoospore loads. Owing to continuing requests for the world *Bd* database, renewed calls for comprehensive analyses of world-scale data of amphibian disease threat patterns (18), and significantly increasing reports of *Bd* research and surveillance (24, 29, 30, 32), the global *Bd* database and web portal warrant maintenance and improved capacity. The new web portal AmphibianDisease.org (41) is developing to enable broader chytrid data reporting (e.g., strains, captive hosts, eDNA, zoospore loads) and user-friendly data import and export functions.

Our aim in this paper is to provide updated summaries of taxonomic and geographic detections and non-detections from newly compiled world *Bd* data through 2019. We use a format for quick comparison to *Bd* occurrence patterns previously reported (28, 42, 43). We summarize *Bd* detection and no-detection data from wild and captive specimens, inclusive of wild-caught museum specimens that have been tested for *Bd*. Geographic patterns of *Bd* detection are assessed for countries, sites, and US 5^th^-field hydrologic unit code (HUC) watersheds (42) which have been used in some land-management decisions to forestall inadvertent *Bd* translocation during water draws for fire-fighting (55, 56). Furthermore, we examine environmental correlates with *Bd* site-level occurrence data compiled through 2019. Several studies have investigated the importance of temperature and moisture regimes for *Bd* occurrence, growth, and host-infection dynamics using laboratory and local- to landscape-scale analyses (57–71). Here, we examine elevation and climate parameters analyzed previously with the *Bd*-Maps.net dataset compiled through 2013 (28) and June 2014 (43) to investigate whether there is stability in *Bd* predictors (e.g., temperature range at a site). Owing to an abundance of new occurrence data and the potential climate-change implications for significant temperature and precipitation metrics with *Bd* occurrences, we conduct downscaled analyses of environmental associations with *Bd* occurrence for North America, South America, Europe, Africa, eastern Asia, and Australia.

Lastly, we compare our *Bd* database tallies by taxon and countries through 2019 with the 2020 results reported by Castro Monzon et al. (30) who examined the peer-reviewed literature of *Bd* occurrences aggregated by a web-search engine. We combine unique taxonomic and country data from Castro Monzon et al. (30) with our findings for an overarching summary of the taxonomic and geographic scope of *Bd* knowledge to date.

## Materials and Methods

*Bd* occurrence data management was based on methods reported previously, whereas analyses conducted here were intended to complement previous assessments (28, 43). To standardize methods among years, *Bd* occurrence database oversight including limited data quality assurance and quality control were conducted by the 2007–2019 *Bd* database manager (KLR).

### Data Compilation

*Bd* occurrence data were compiled primarily by four methods. First, an initial dataset was compiled by regional data coordinators who submitted project data or reports for their regions for the 2007 Global *Bd* Mapping Project, presented in the first *Bd* map at the International *Bd* Conference, Tempe, Arizona, USA, in November 2007; this Global *Bd* Mapping Project database initiated the development of the *Bd*-Maps.net web portal (28). Second, *Bd* surveillance data were directly uploaded to *Bd*-Maps.net by principal investigators, 2007–2014. Third, web-based literature searches were conducted of the main international and regional journals reporting on *Bd* studies (Supplementary Appendix 1). Fourth, published or unpublished reports were sent directly to us (DHO, KLR) for import to the *Bd*-Maps database. We quantified the number of data sources in our 2019 database by five types: (1) peer-reviewed journal articles; (2) reports; (3) theses and dissertations; (4) online sources (newspapers, newsletters, online compilations); and (5) unpublished contributed datasets.

Amphibian taxonomy and geographic locations of *Bd* sampling per report were examined for reporting consistency (Supplementary Appendix 1). Taxonomy used herein followed Frost [(72); Supplementary Appendix 1]. Geographic locations with detectable errors (e.g., coordinates clearly outside the study area) were corrected by consultation with principal investigators, or based on other location information provided in the report. Laboratory results of *Bd* analyses were not examined for scientific integrity, including analytical sensitivity or accuracy (e.g., sample size analyzed; histological or PCR analyses). Hence, we caution that “no detection” is not synonymous with *Bd* absence in a sample, as likelihood of detection can vary with population size, sample size, *Bd* prevalence, and analysis method [e.g., (47, 73)]. Data duplication was assessed for studies imported to the *Bd*-Maps database prior to publication that were later identified in literature searches of published papers.

### *Bd* Occurrence Categorization by Taxa and Geography

*Bd* data were compiled for species, records, sites, watersheds (USA only), and at the region or country level when precise coordinates or locations were not available. To assess whether *Bd* had ever been detected in an amphibian taxon, the composite data records were compiled and the taxon was labeled as “*Bd* detected” if there had ever been a single *Bd*-positive report. “*Bd* not detected” was the usual alternative, however a few reports have been challenged in the literature due to diagnostics concerns, and as a precaution those were labeled as “uncertain,” as were cases where the authors themselves reported an uncertain result of a diagnostics test. A “record” was a database entry for a species at a particular location for a study (28). There were multiple records for a location if *Bd* sampling occurred for multiple species, sampling occasions, or studies.

Site-level data compilations were composite records for a common latitude/longitude coordinate, or a specific locality description (28). Site-level *Bd* occurrence was assigned to one of three categories: *Bd* detected; *Bd* not detected; *Bd* detection uncertain. Thus, even if multiple species were sampled for *Bd* at a unique geographic coordinate, or the location was sampled over multiple years, the site was designated “*Bd* detected” if *Bd* had ever been detected at that location for any species in any year. Site-level *Bd* detected and not-detected data were included in geospatial analyses described below. Countries were designated as “*Bd* detected” based on field or museum specimens sampled or collected from the wild. If the only positive sample for a country came from a captive sample, the country was not designated as “*Bd* detected.” An analysis of continental USA watershed-scale *Bd* occurrence was conducted. As for sites, an individual watershed was designated as “*Bd* detected” if *Bd* had ever been detected in samples of any species in any year. If a watershed had been sampled but *Bd* had never been detected there, it was labeled as “*Bd* not detected.” For country-scale patterns, a country was labeled as *Bd* detected or not detected based on the composite data in the database for that nation.

### Environmental Predictors of *Bd* Occurrence

Analyses of *Bd* occurrence associations with environmental attributes focused on elevation and 14 climate metrics (Table 1) of world sites with *Bd* sampling compiled through 2019. Elevation and climate data were derived from online global geographic models (Supplementary Appendix 1). World climate data were available for 0.5-degree latitude/longitude grid cells, hence *Bd* site-level occurrences were consolidated per grid cell for consistency with climate data (i.e., per grid cell, *Bd* was either detected or not). This consolidation likely reduces potential spatial autocorrelation, data collection biases among sampling events, and geographic- and population-level redundancy considerations of the reported source data.

**Table 1:** Environmental attributes analyzed for associations with *Batrachochytrium dendrobatidis* (*Bd*) occurrence (detection, no detection) across world sites with *Bd* sampling compiled through 2019.

**Attribute (units)**	**Description**
Elevation (m)	Altitude above sea level
Mean annual precipitation (mm)	10-year mean annual precipitation
Low average monthly precipitation (mm)	10-year average of lowest monthly precipitation
Mean average monthly precipitation (mm)	10-year mean of average monthly precipitation
High average monthly precipitation (mm)	10-year average of highest monthly precipitation
Temperature range (°C)	10-year monthly average daily maximum temperature (tmax) minus 10-year monthly average daily minimum temperature (tmin)
Low average monthly temperature (°C)	10-year average of lowest monthly temperature
Mean average monthly temperature (°C)	10-year mean of average monthly temperature
High average monthly temperature (°C)	10-year average of highest monthly temperature
Low average monthly minimum temp. (°C)	10-year average of lowest monthly minimum temperature
Mean average monthly minimum temp. (°C)	10-year mean of average monthly minimum temperature
High average monthly minimum temp. (°C)	10-year average of highest monthly minimum temperature
Low average monthly maximum temp. (°C)	10-year average of lowest monthly maximum temperature
Mean average monthly maximum temp. (°C)	10-year mean of average monthly maximum temperature
High average monthly maximum temp. (°C)	10-year average of highest monthly maximum temperature

To avoid collinearity issues, we removed highly correlated predictor variables (74). Consequently, we refined elevation and climate data to six parameters for analyses. Three elevation metrics were determined per 0.5-degree latitude and longitude grid cell and used in analyses: mean elevation; minimum elevation; and maximum elevation. For climate metrics, 10-year mean annual precipitation was highly correlated (>0.7) with: 10-year average of lowest monthly precipitation, 10-year average of highest monthly precipitation, and 10-year average of average monthly precipitation. Thus, only 10-year mean annual precipitation was used in analyses. Similarly, 10-year mean annual daily temperature was highly correlated (>0.7) with all other temperature variables (10-year lowest mean temperature, 10-year highest mean temperature, 10-year mean low temperature, 10-year lowest mean low temperature, 10-year highest mean low temperature, 10-year mean high temperature, 10-year lowest mean high temperature, 10-year highest mean high temperature). Hence, only 10-year mean annual daily temperature was used in analyses. The final six covariates used in the models of environmental associations with *Bd* occurrence were: 10-year mean annual precipitation, 10-year mean annual daily temperature, 10-year average temperature range, mean elevation, minimum elevation within the cell, and maximum elevation within the cell.

Both presence-only and presence-and-absence (i.e., absence = no detection) Species Distribution Models (SDMs) were evaluated. Given the uncertain nature of true absences, presence-only models have been considered more robust (75), whereas presence-absence data include the broader dataset assembled for *Bd* and can be compared with previous models. With global and regional subsets of data, using presence-only (detections-only) data, a maximum-entropy SDM was used to estimate the effect of environmental covariates on relative odds of *Bd* occurrence. With global and regional datasets, using both detection and no-detection data, a logistic regression SDM was used to estimate the effect of environmental covariates on odds of *Bd* occurrence. As we expect non-linear relationships between environmental covariates and probability of *Bd* occurrence, we transformed each covariate (linear, monotonous, deviation, forward hinge, reverse hinge, threshold) and used forward selection to select the transformations that best-explained variation in *Bd* occurrence. After variable transformation, a subset selection procedure was used to determine the best-fit model (i.e., select the final environmental covariates). As we expected interactions among covariates (e.g., the effect of mean temperature depends on annual precipitation), we allowed for interactions among all covariates. To visualize the form of the relationship between final model covariates and probability of *Bd* occurrence (e.g., unimodal), we plotted model predictions for a range of the environmental covariate while holding all other environmental covariates at their mean. To visualize the form of interactions among covariates, we plotted model predictions for a range of the environmental covariate while holding the interacting covariate at the 0.25% percentile, mean, and 0.75%, and all other covariates at the mean.

Per SDM, we determined the fraction of total variation accounted (FTVA) for by main parameters in the best-fit model [i.e., measure of the parameter contribution to explain variation in *Bd* occurrence (76)]. We evaluated model performance by calculating the area under the curve (AUC) which provides an aggregate measure of model sensitivity (i.e., ability to correctly classify grid cells with *Bd* detection) and specificity (i.e., ability to correctly classify grid cells with no *Bd* detection). At AUC = 1.0, the model can perfectly categorize true negatives and positives, whereas if AUC = 0, it incorrectly categorizes all true negatives and positives. If AUC = 0.5, the model makes predictions equivalent to random guesses. For each SDM, we trained the model using 75% of the data and tested the model with the remaining 25%. The data were randomly split into a training and test set using the R package *caTools* (77). Final models were fit with the entire dataset. FTVA and AUC were calculated using *MIAMaxent*.

Finally, to visualize habitat suitability of *Bd* using analyzed environmental parameters, we calculated model predictions for each grid location within a global or regional map and plotted using model predictions. Importantly, predictions for the presence-only models were scaled to the probability ratio output [PRO (78)] and can be interpreted as relative habitat suitability of *Bd* occurrence (79), whereas predictions from the presence-absence models represented absolute probability of *Bd* occurrence. The probability ratio output was log_2_ (log base 2) transformed to improve visualization. We excluded data from Madagascar when fitting the African regional models owing to some uncertain results for the area in the literature (see Supplementary Table 1 footnote), but we projected the African regional models to Madagascar to show potential *Bd* occurrence. We also excluded data from Papua New Guinea when fitting the regional model because the amphibian fauna has similarities to Australia, whereas habitat may be more reflective of Southeast Asia; we projected the Asian model to Papua New Guinea to predict potential *Bd* occurrence probability based on Asian *Bd* environmental associations. SDM model predictions were calculated in the R package *MIAMaxent* (80) and global and regional predictions were plotted in the R package *ggplot2* (81).

## Results

**Figure 1:**
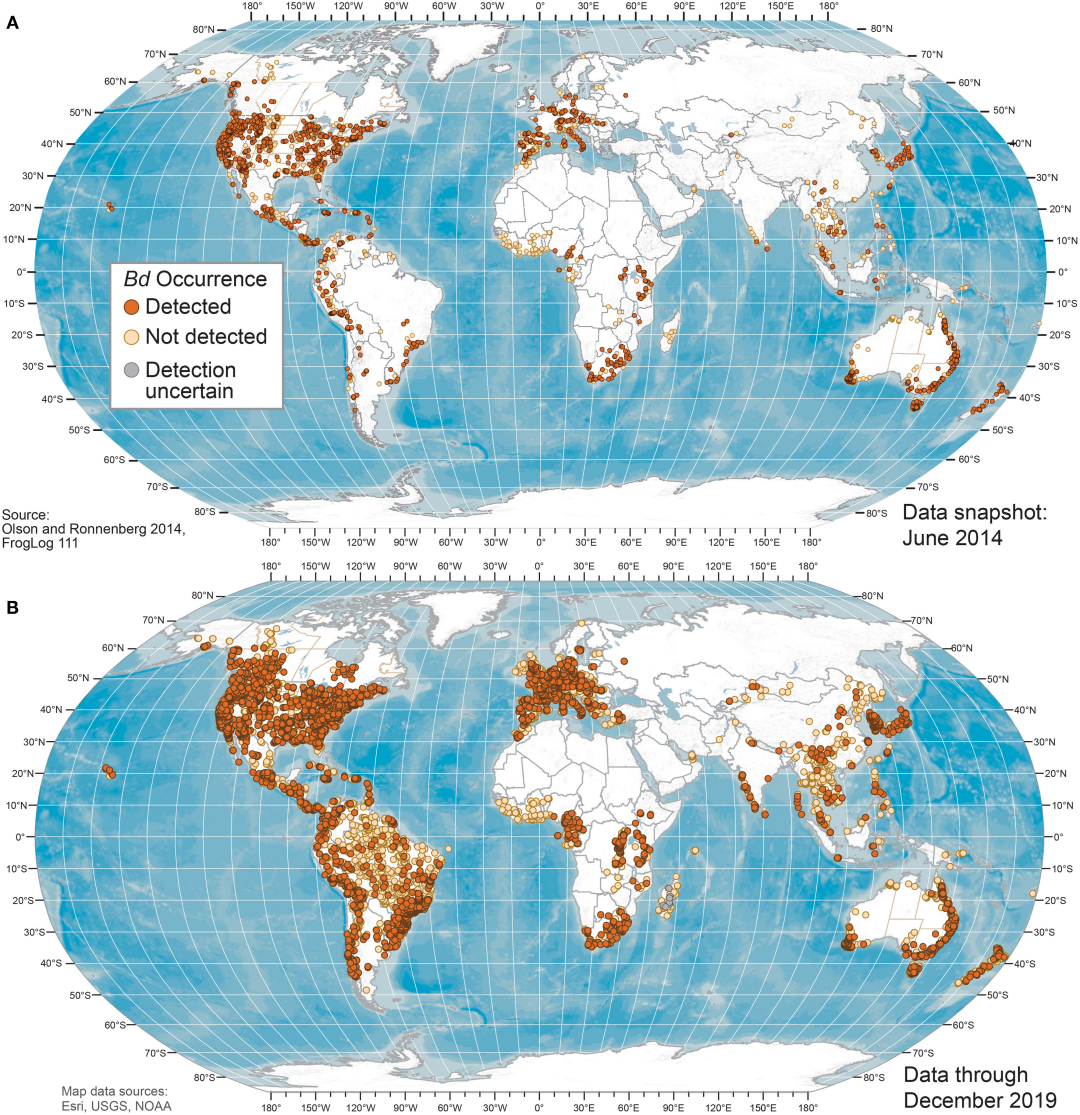
World maps of *Batrachochytrium dendrobatidis* (*Bd*) occurrences at unique sites from data compiled for the Global *Bd* Mapping project through: **(A)** June 2014 (42); and **(B)** December 2019. Sites shown with *Bd* detections also may have sampling results with no detection; records with only country-level coordinates are not mapped.

**Figure 2:**
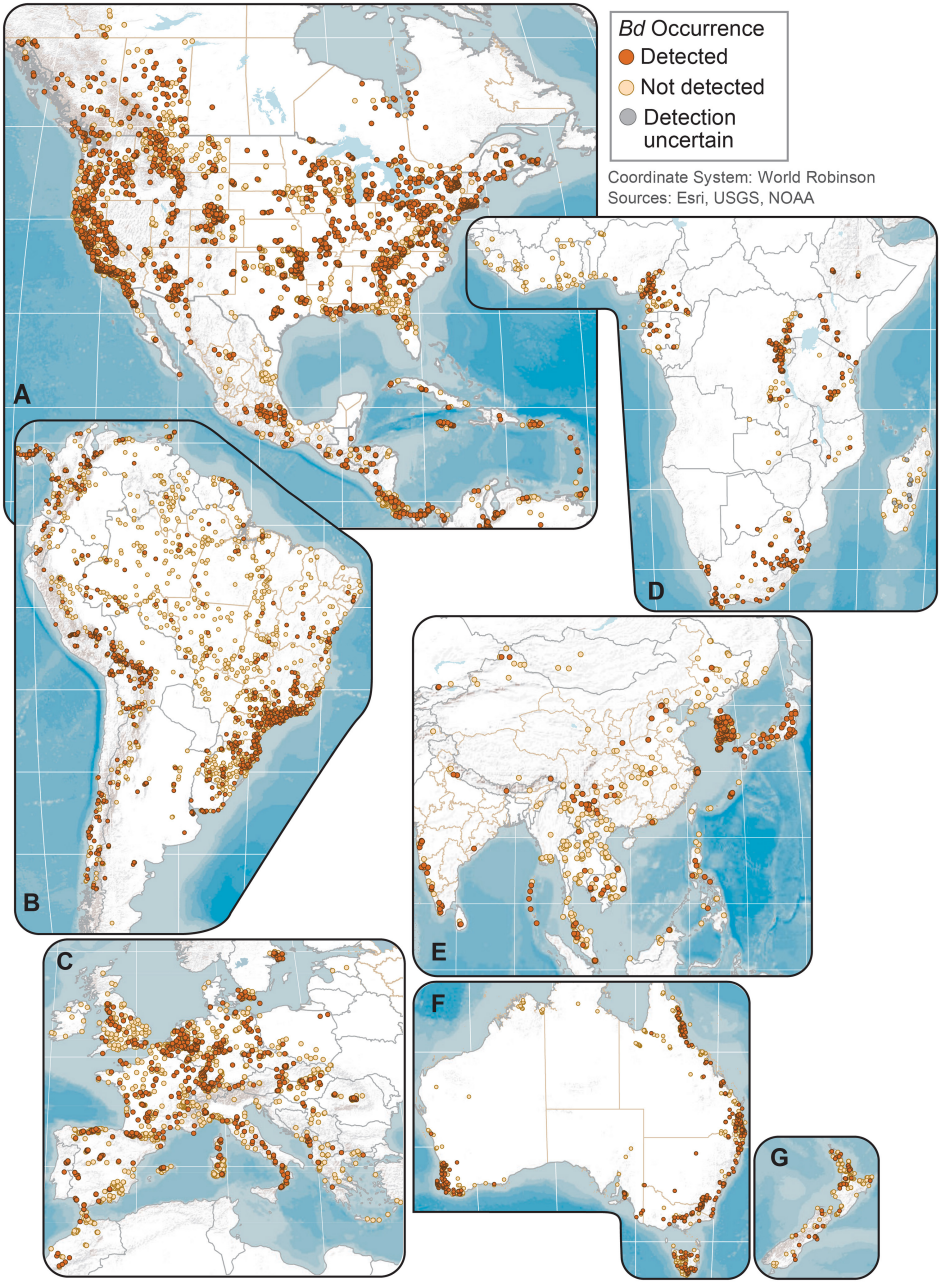
Regional maps of *Batrachochytrium dendrobatidis* (*Bd*) occurrences at unique sites from data compiled for the Global *Bd* Mapping project: **(A)** North America; **(B)** South America; **(C)** Europe; **(D)** Africa; **(E)** Eastern Asia; **(F)** Australia; and **(G)** New Zealand. Sites shown with *Bd* detections also may have sampling results with no detection; records with only country-level coordinates are not mapped.

**Figure 3:**
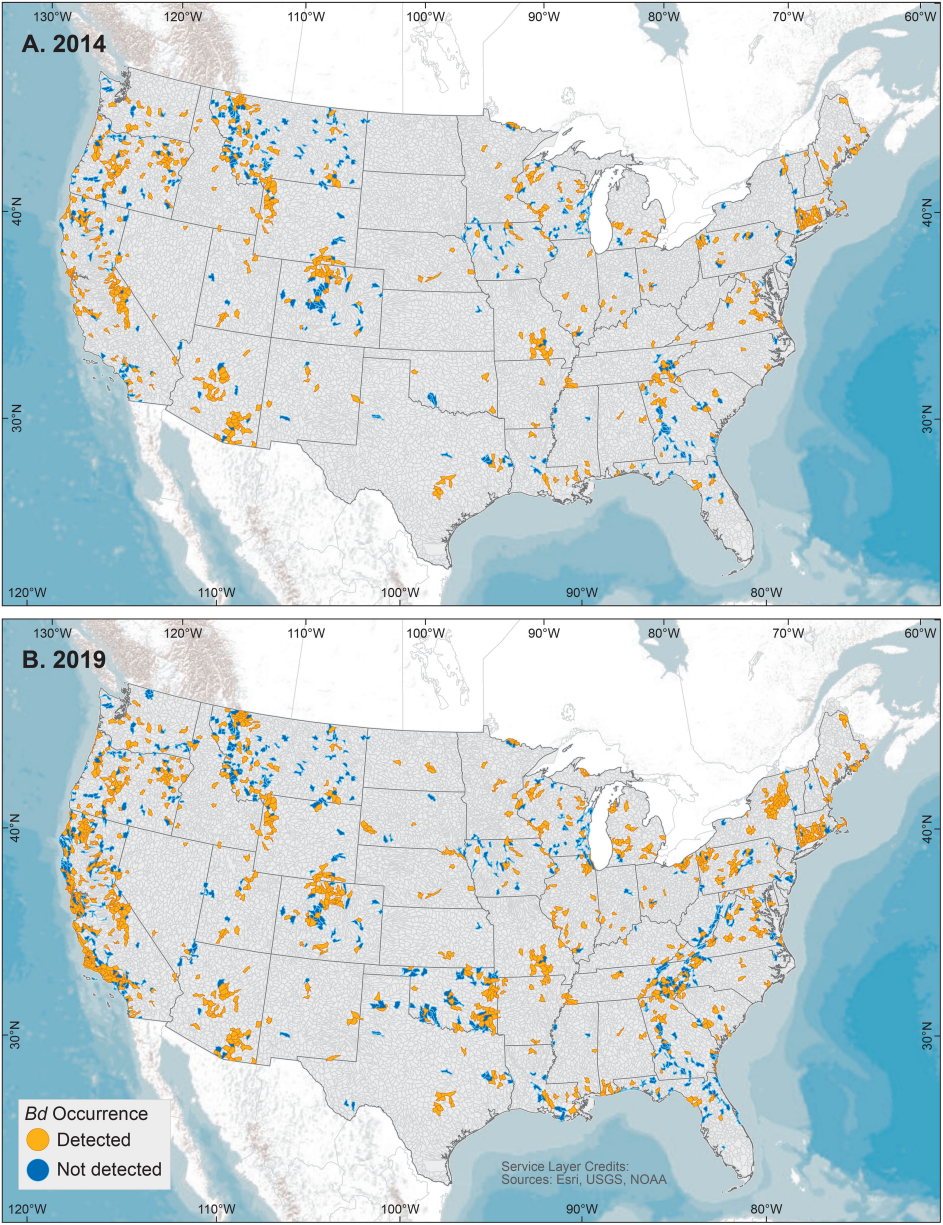
United States 5^th^-field hydrologic unit code watershed maps of *Batrachochytrium dendrobatidis* (*Bd*) occurrences from data compiled for the Global *Bd* Mapping project through: **(A)** June 2014 (42); and **(B)** December 2019. Watersheds shown with *Bd* detections also may have sampling results with no detection.

Our *Bd* occurrence data compilation through 2019 included 773 sources: 661 peer-reviewed journal articles; 16 reports or proceedings; 13 theses and dissertations; 5 online sources; and 78 unpublished contributed datasets. Worldwide *Bd* surveillance across amphibian taxa and geographies through 2019 showed advancing knowledge of *Bd* occurrences, with geographic knowledge gaps filled compared to June 2014 (Figures 1–**3**). *Bd* data were summarized across 33,753 overall sampling records (e.g., sampling effort for a species for a project location in a year) with *Bd* detections and no-detections for wild (including museum specimens of wild-caught animals) and captive animals (Supplementary Table 1).

### Taxonomic Patterns

Through 2019, our world *Bd* data compilation showed that *Bd* had been detected in 1,294 of 2,412 (54%) amphibian species sampled, and that sampling had been conducted in 29% of all amphibian species (Table 2 and Supplementary Table 2). Anurans (frogs and toads) had the highest species-level prevalence of infection (54.7%) compared to caudates (newts and salamanders: 49.2%), and gymnophionans (caecilians: 29.2%). Through 2019, there were *Bd* detections in 86% of amphibian families. *Bd* surveys have been reported for all amphibian families except one anuran family (Nasikabatrachidae, 2 spp.: Western Ghats, India); one caudate family (Rhyacotritonidae, 4 spp.: Pacific Northwest USA); and one gymnophionan family (Chikilidae, 4 spp.: Northeast India) (Tables 2, 3). However, we are aware that in ongoing experiments of *Bsal* susceptibility in USA salamanders, wild-caught members of Rhyacotritonidae have been screened for *Bd* prior to use in laboratory trials, and *Bd* has not been detected (J. Piovia-Scott, Washington State University, Vancouver, WA, USA; pers. commun.). Species-level *Bd* prevalence among families was highly variable (Table 3). Through 2019, 6 of 55 (11%) Anura families and 3 of 9 (33%) Gymnophiona families had no *Bd* detections among sampled species (Tables 2, 3).

**Table 2:** Global *Batrachochytrium dendrobatidis* (*Bd*) detections in amphibians as compiled through December 2019.

	**Species**				**Families**			
	***Bd* detected**	**Tested**	**Prevalence (%)**	**Total species**	***Bd* detected**	**Tested**	**Prevalence (%)**	**Total families**
Anura	1,153	2,106	54.7	7,311	49	55	89.1	56
Caudata	127	258	49.2	762	8	8	100	9
Gymnophiona	14	48	29.2	210	6	9	66.7	10
Total	1,294	2,412	53.6	8,283	63	72	85.9	75

**Table 3:** Family-level summary of *Batrachochytrium dendrobatidis* (*Bd*) detections among species sampled for *Bd*.

**Family**	**No. spp. *Bd* detected**	**No. spp. tested**	**Spp. prevalence**	**Total spp. in family**
**Anura** ^**a**^				
Allophrynidae	0	1	0.00	3
Alsodidae	8	17	0.47	26
Alytidae	7	9	0.78	12
Aromobatidae	17	25	0.68	128
Arthroleptidae	52	96	0.54	149
Ascaphidae	1	2	0.50	2
Batrachylidae	6	7	0.86	12
Bombinatoridae	4	5	0.80	8
Brachycephalidae	9	12	0.75	74
Brevicipitidae	1	7	0.14	37
Bufonidae	102	204	0.50	630
Calyptocephalellidae	2	2	1.00	5
Centrolenidae	18	33	0.54	156
Ceratobatrachidae	2	14	0.14	102
Ceratophryidae	7	7	1.00	12
Conrauidae	2	5	0.40	6
Craugastoridae^b^	97	185	0.52	874
Cycloramphidae^c^	13	19	0.68	36
Dendrobatidae	33	49	0.67	203
Dicroglossidae	17	46	0.37	215
Eleutherodactylidae	36	68	0.53	230
Heleophrynidae	5	5	1.00	7
Hemiphractidae	20	225	0.80	118
Hemisotidae	0	2	0.00	9
Hylidae^d^	190	255	0.74	734
Hylodidae	23	29	0.79	47
Hyperoliidae	68	107	0.64	228
Leiopelmatidae	1	4	0.25	4
Leptodactylidae	49	87	0.56	231
Limnodynastidae	14	23	0.61	43
Mantellidae	1^e^	77	0.01	233
Megophryidae	5	31	0.16	280
Micrixalidae	1	2	0.50	24
Microhylidae	26	96	0.27	703
Myobatrachidae	20	37	0.54	89
Nyctibatrachidae	2	2	1.00	39
Odontobatrachidae	0	1	0.00	5
Odontophrynidae^f^	7	11	0.64	50
Pelobatidae	3	4	0.75	5
Pelodryadidae^g^	35	69	0.51	219
Pelodytidae	0	1	0.00	5
Petropedetidae	6	11	0.54	13
Phrynobatrachidae	20	40	0.50	95
Phyllomedusidae	18	25	0.72	67
Pipidae	21	26	0.81	41
Ptychadenidae	12	23	0.52	64
Pyxicephalidae	20	28	0.71	85
Ranidae	96	166	0.58	419
Ranixalidae	2	6	0.33	18
Rhacophoridae	27	62	0.44	434
Rhinodermatidae^h^	2	2	1.00	3
Rhinophrynidae	0	1	0.00	1
Scaphiopodidae	4	7	0.57	7
Sooglossidae	0	3	0.00	4
Telmatobiidae	21	25	0.84	63
Total Anura	1,153	2106	0.55	7,311
**Caudata** ^**i**^				
Ambystomatidae	20	24	0.83	37
Amphiumidae	2	3	0.67	3
Cryptobranchidae	3	3^j^	1.00	4
Hynobiidae	3	20	0.15	85
Plethodontidae	70	150	0.47	491
Proteidae	4	4	1.00	9
Salamandridae	22	50	0.44	128
Sirenidae	3	4	0.75	5
Total Caudata	127	258	0.49	762
**Gymnophiona** ^**k**^				
Caeciliidae	0	7	0.00	43
Dermophiidae	2	5	0.40	14
Herpelidae	2	3	0.67	10
Ichthyophiidae	0	5	0.00	57
Indotyphlidae	1	7	0.14	24
Rhinatrematidae	0	2	0.00	14
Scolecomorphidae	2	3	0.67	6
Siphonopidae	3	7	0.43	26
Typhlonectidae	4	9	0.44	14
Total gymnophiona	14	48	0.29	210

*Total species in family as of November 2020 based on Frost 2020 (72)*.

a *Family Nasikabatrachidae (Western Ghats of India, with 2 species) not yet sampled*.

b *Includes former family Strabomantidae*.

c *Not including family Rhinodermatidae, listed separately below; genus* Proceratophrys *moved to Odontophrynidae*.

d *Not including species split off into new family Pelodryadidae*.

e *Twelve species with uncertain positive tests in Madagascar, 1 positive captive animal in the USA, see Supplementary Material and discussion of Madagascar results in the text*.

f *Genus* Proceratophrys *moved from Cycloramphidae to Odontophrynidae*.

g *New family split from Hylidae*.

h *New family split from Cyclorhamphidae*.

i *Family Rhyacotritonidae (Pacific Northwest United States, 4 spp.) may have been sampled, but results have not yet been reported*.

j *Prior versions of this table treated* Cryptobranchus alleganiensis alleganiensis *and* C. a. bishopi *as separate species, but the current taxonomy regards them as one species*.

k *Family Chikilidae (Northeast India, 4 spp.) has not been sampled*.

### Geographic Patterns

Geographically, our compilation of studies detected *Bd* in the wild in 88 of 124 (71%) countries sampled through 2019 (Figures 1, 2 and Supplementary Table 3); these 124 countries included 6 countries for which only *Bd*-negative (no-detection) samples with a “country-centroid coordinate” were reported (i.e., no location reported: Armenia, Barbados, Central African Republic, Gambia, Iran, Latvia). We recognize that some country names and boundaries have been dynamic, our intention here is to include recognizable principalities over time. For example, the record of *Bd* occurrence in North Korea is from the analysis of a museum specimen reported in 2015 (82) yet the animal had been collected in the year 1911 when the Korean Peninsula was a single political entity; Hong Kong is included separately here although it is now part of China. Our limited quality assurance and quality control of reported data resulted in correction of a small minority of location coordinates (Supplementary Appendix 1).

We examined our data by sites (i.e., locations having a common latitude/longitude coordinate for sampling of one or more amphibian species for *Bd* infection), compiling *Bd* sampling across 14,647 discrete sites worldwide, inclusive of both wild and captive animals but not including 67 reports from regions lacking geographic specificity (Supplementary Table 1). We could not know if captive animals were infected with *Bd* in the wild or during captivity, so captive sites were not used in subsequent analyses of environmental associations. Excluding captive animals and a small number of results with diagnostic uncertainties, *Bd* in wild-caught amphibians was detected at 5,550 of 14,413 (38.5%) sites. These 14,413 site-level *Bd* detection and no-detection data were used in analyses of climatic and geographic correlates (below). We mapped *Bd* detections and no-detections for continental-USA 5th-field HUC watersheds: *Bd* detections were reported for 916 of 1,874 (49%) sampled watersheds (Figure 3).

### Environmental Associations With *Bd* Occurrence

Consolidation of site-level *Bd* occurrence data compiled through December 2019 into 0.5-degree grid cells resulted in 3,777 grid cells used in global SDMs analyzed with both detection and no-detection data. Using only the detection data in global SDMs (presence-only models), 2,012 grid cells were analyzed.

Using only detection data, the best-fit global model included four environmental parameters and their interactions per grid cell: 10-year mean annual daily temperature (mean temp); 10-year mean annual precipitation (annual precipitation); 10-year average temperature range (temp range); and maximum elevation within the grid cell (elevation max). The relative probability of *Bd* occurrence was a function of mean temp + annual precipitation + temp range + elevation max + (annual precipitation ^*^ elevation max) + (annual precipitation ^*^ temp range) + (mean temp ^*^ elevation max) + (mean temp ^*^ temp range) + (mean temp ^*^ annual precipitation) + (elevation max ^*^ temp range). Mean annual daily temperature accounted for the highest fraction of total variation in probability of *Bd* occurrence (0.713), with annual precipitation accounting for the second highest fraction (0.218; Table 4).

**Table 4:** Fraction of total variation accounted for (FTVA) by each variable in best-fit global species distribution models (SDMs) of *Batrachochytrium dendrobatidis* (*Bd*) occurrence from data compiled through December 2019.

**Model**	**Variable**	**FTVA**
Presence-only global (AUC = 0.86)	10-year mean annual daily temperature	0.713
	10-year mean annual precipitation	0.218
	maximum elevation in a 55-km grid cell	0.046
	10-year average temperature range	0.023
Presence-absence global (AUC = 0.63)	10-year mean annual daily temperature	0.969
	10-year average temperature range	0.031

*The presence-only model used Bd detection data and the presence-absence model used Bd detection and no-detection data. AUC, area under the curve, measure of model sensitivity (ability to correctly classify 0.5-degree latitude/longitude grid cells with Bd detection)*.

In the SDM derived from both detection and no-detection data at the global scale, the best-fit model included two environmental parameters and their interactions: mean temp and temp range. Probability of *Bd* occurrence was a function of mean temp + temp range + mean temp ^*^ temp range. Variation in probability of *Bd* occurrence was primarily described by mean temperature (0.969; Table 4).

**Figure 4:**
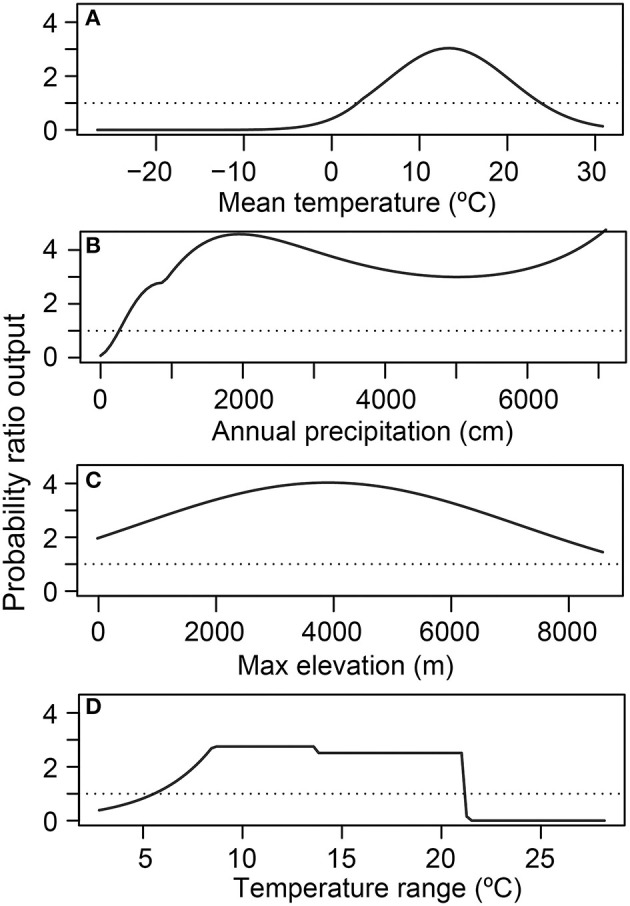
Relative habitat suitability of *Batrachochytrium dendrobatidis* (*Bd*) occurrence (probability ratio output) for each environmental attribute [**(A)** mean temperature; **(B)** annual precipitation; **(C)** maximum elevation; **(D)** temperature range] from the global presence-only best-fit species distribution model. Each environmental attribute marginal-response plot is calculated while holding all other covariates at the mean.

**Figure 5:**
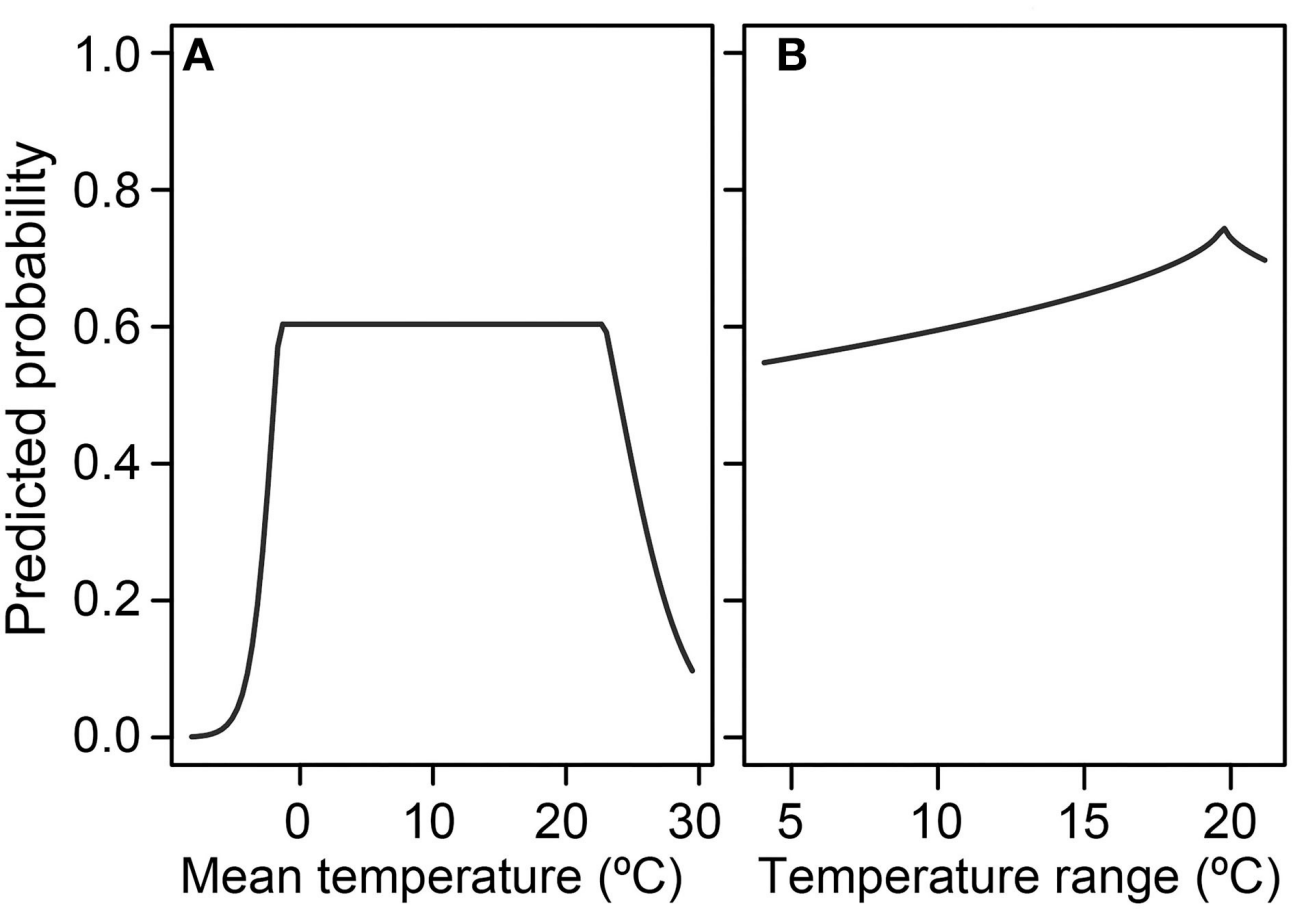
Absolute probability of *Batrachochytrium dendrobatidis* (*Bd*) occurrence for the environmental attributes **(A)** mean temperature and **(B)** temperature range from the global presence-absence best-fit species distribution model.

In the global SDMs, the relative probability (presence-only model) or absolute probability (presence-absence model) of *Bd* occurrence responded non-linearly to each environmental covariate (Figures 4, 5). A more detailed representation of these figures showing frequency of observations for each environmental covariate and kernel-estimated data density showing the sampling effort (no. grid cells) are depicted in Supplementary Figures 1, 2.

In the SDM derived from detection-only data, there was a unimodal relationship between 10-year mean annual daily temperature and relative probability of *Bd* occurrence, peaking at ~12–13°C, when holding all other covariates at their mean (Figure 4A). Relative probability of *Bd* occurrence had a more complex relationship with mean annual precipitation, showing an initial modal maximum at ~1,200–1,400 mm, a dip at ~3,000 mm, before increasing around 4,000 mm, when holding all other covariates at their mean (Figure 4B). Relative probability of *Bd* also formed a unimodal relationship with maximum elevation, peaking at ~4,000 m, when holding all other covariates at their mean (Figure 4C). The relationship between temperature range and *Bd* occurrence plateaued between ~8 and 21°C, with a stark decrease in relative probability of *Bd* occurrence at both cooler and warmer temperatures, when holding all other covariates at their mean (Figure 4D). To visualize interactions among covariates, model predictions were plotted with interacting covariates held at the 0.25% percentile, mean, and 0.75% percentile (Supplementary Figures 3A,B). The AUC for the global presence-only model was 0.86, indicating a model with high sensitivity and specificity.

In the presence-absence model, at the average temperature range, probability of *Bd* detection increased once mean temperature increased past 0°C and decreased when mean temperature exceeded ~20°C (Figure 5A). At the mean temp, probability of *Bd* detection tended to increase as temperature range increased, with a peak around 18°C (Figure 5B). Interactions between mean temperature and temperature range are plotted in Supplementary Figure 4. The AUC for the presence-absence global model was 0.63, indicating a model with less sensitivity and specificity than the presence-only model.

**Figure 6:**
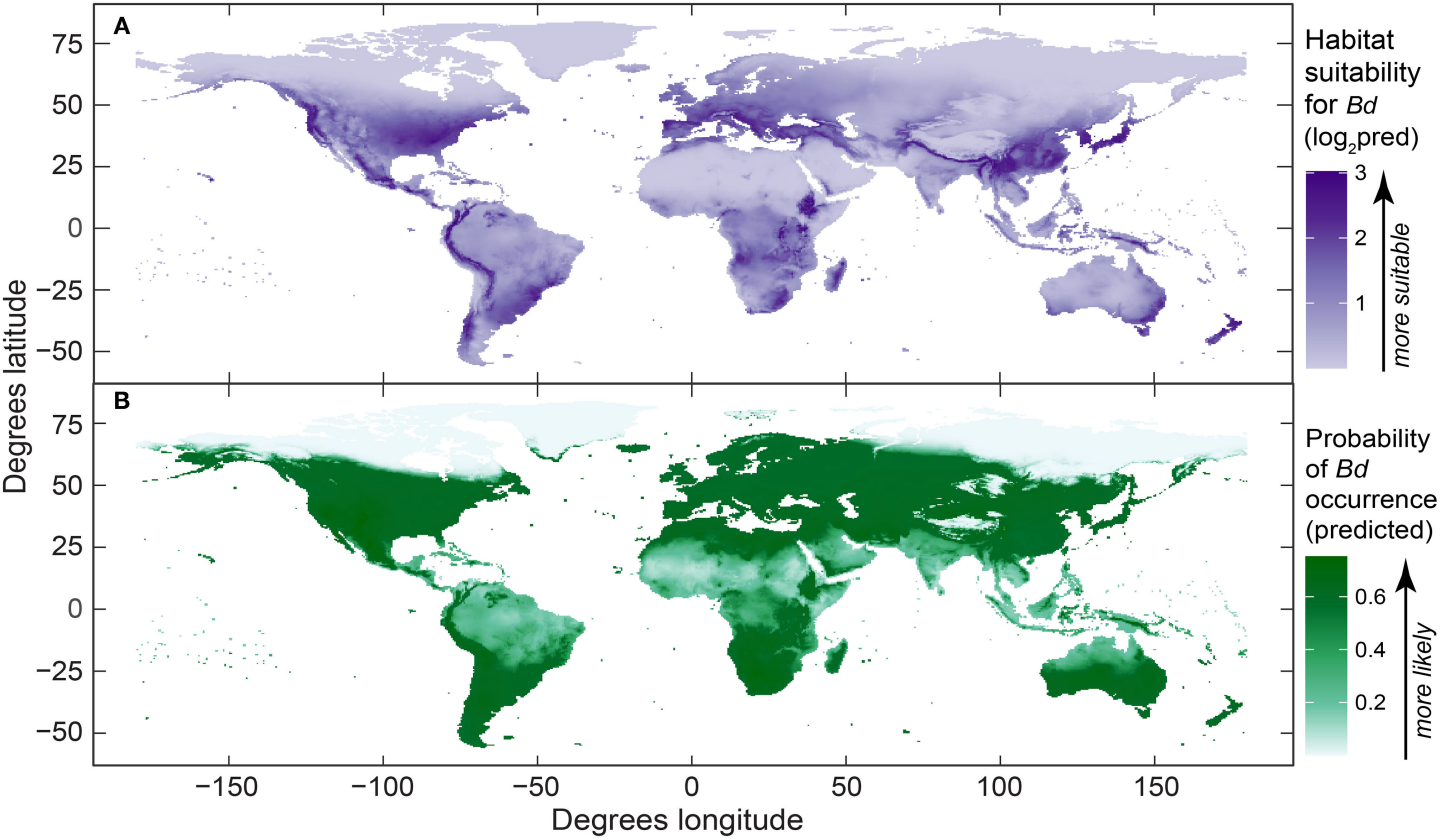
Global maps of predicted *Batrachochytrium dendrobatidis* (*Bd*): **(A)** habitat suitability derived from the best-fit presence-only species distribution model (log_2_-transformed [log base 2] probability ratio output, area under curve [AUC] = 0.86); and **(B)** probability of *Bd* occurrence from the best-fit presence-absence species distribution model (AUC = 0.62). Both maps were derived using *Bd* data compiled through December 2019.

The maps of *Bd* habitat suitability (presence-only model) and probability of *Bd* occurrence (presence-absence model) from our best-fit global models (Figure 6) were reflective of our dot distribution of *Bd* occurrences (Figure 1). Areas of heightened likelihoods of *Bd* occurrence included mesic mid-latitude and coastal influences, especially when considering the presence-only model. North-temperate, interior-continental, and arid zones had lowest *Bd* probabilities.

**Figure 7:**
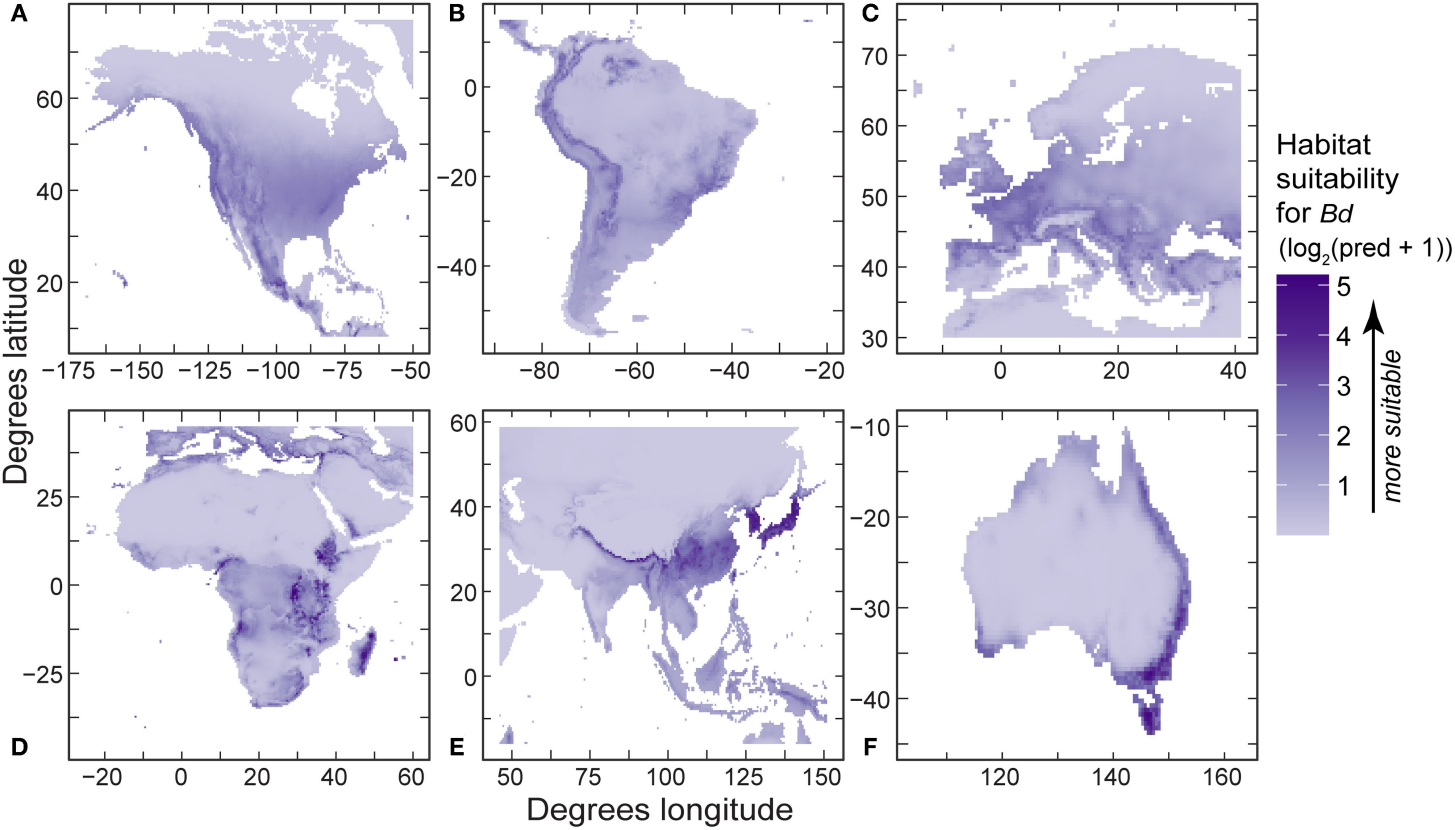
Regional predictions of *Batrachochytrium dendrobatidis* (*Bd*) habitat suitability from our best-fit presence-only species distribution models (log_2_-transformed [log base 2] probability ration output, area under curve values in Table 5) using *Bd* data compiled through December 2019 for: **(A)** North America; **(B)** South America; **(C)** Europe; **(D)** Africa; **(E)** eastern Asia; and **(F)** Australia.

In regional SDMs, relative probability (using detection-only data) and absolute probability of *Bd* (using detection and no-detection data) also responded non-linearly to environmental covariates. For regional SDMs using detection-only data, 10-year mean annual daily temperature (mean temp) was retained in all final models (Table 5), with mean temperature capturing the most variation in relative probability of *Bd* occurrence in North America, South America, and Europe (Table 5). In all three regional models, shape of the response of *Bd* occurrence reflected that of the regional model (unimodal with a peak around ~12–13°C). Notably, in North America and South America, the effect of mean temperature changed with temperature range and max elevation, respectively. In Africa, max elevation in a ~55-km grid cell (max elevation) and annual precipitation contributed the most variation to relative probability of *Bd* occurrence (0.413 and 0.339, respectively; Table 5). When all other covariates were held at their mean, relative probability of *Bd* occurrence increased with max elevation until a plateau around 2,000 m and linearly increased with annual precipitation. However, the effect of precipitation was dependent upon mean temperature. In Asia, annual precipitation contributed the most variation in relative probability of *Bd* occurrence followed by mean temperature (0.626 and 0.374, respectively; Table 5). The relative probability of *Bd* occurrence increased with annual precipitation, whereas model response to mean temperature followed a unimodal pattern reflective of the global presence only model. In Australia, temperature range contributed the most variation to relative probability of *Bd* occurrence (0.675, Table 5). When all other covariates were held at their mean, relative probability of *Bd* followed a unimodal response to temperature range, with relative probability of *Bd* occurrence peaking at a temperature range of ~10°C. Model predictions from the presence-only models (PRO of *Bd* occurrence) were projected in regional maps (Figure 7). AUC for regional presence-only models ranged from 0.79 to 0.93 (Table 5).

**Table 5:** Final best-fit model covariates of regional presence-only species distribution models (North America, South America, Europe, Africa, Asia, Australia), including regional model area under the curve (AUC), and fraction of total variation accounted for (FTVA) for each variable.

**Region and final best-fit model**	**Variable**	**FTVA**
**North America (AUC** **=** **0.89)**		
Mean temp + annual precipitation + elevation max + mean temp*elevation max	Mean temp	0.910
	Annual precipitation	0.034
	Elevation max	0.056
**South America (AUC** **=** **0.91)**		
Mean temp + elevation max + temp range + mean temp*elevation max	Mean temp	0.667
	Elevation max	0.292
	Temp range	0.042
**Europe (AUC** **=** **0.79)**		
*Mean temp + annual precipitation*	Mean temp	0.833
	Annual precipitation	0.167
**Africa (AUC** **=** **0.90)**		
Elevation max + annual precipitation + mean temp + temp range + annual precipitation*mean temp	Elevation max	0.413
	Annual precipitation	0.339
	Mean temp	0.183
	Temp range	0.064
**Asia (AUC** **=** **0.91)**		
*Annual precipitation + mean temp*	Annual precipitation	0.626
	Mean temp	0.374
**Australia (AUC** **=** **0.93)**		
*Temp range + elevation max + mean temp + annual precipitation*	Temp range	0.675
	Elevation max	0.211
	Annual precipitation	0.066
	Mean temp	0.048

**Figure 8:**
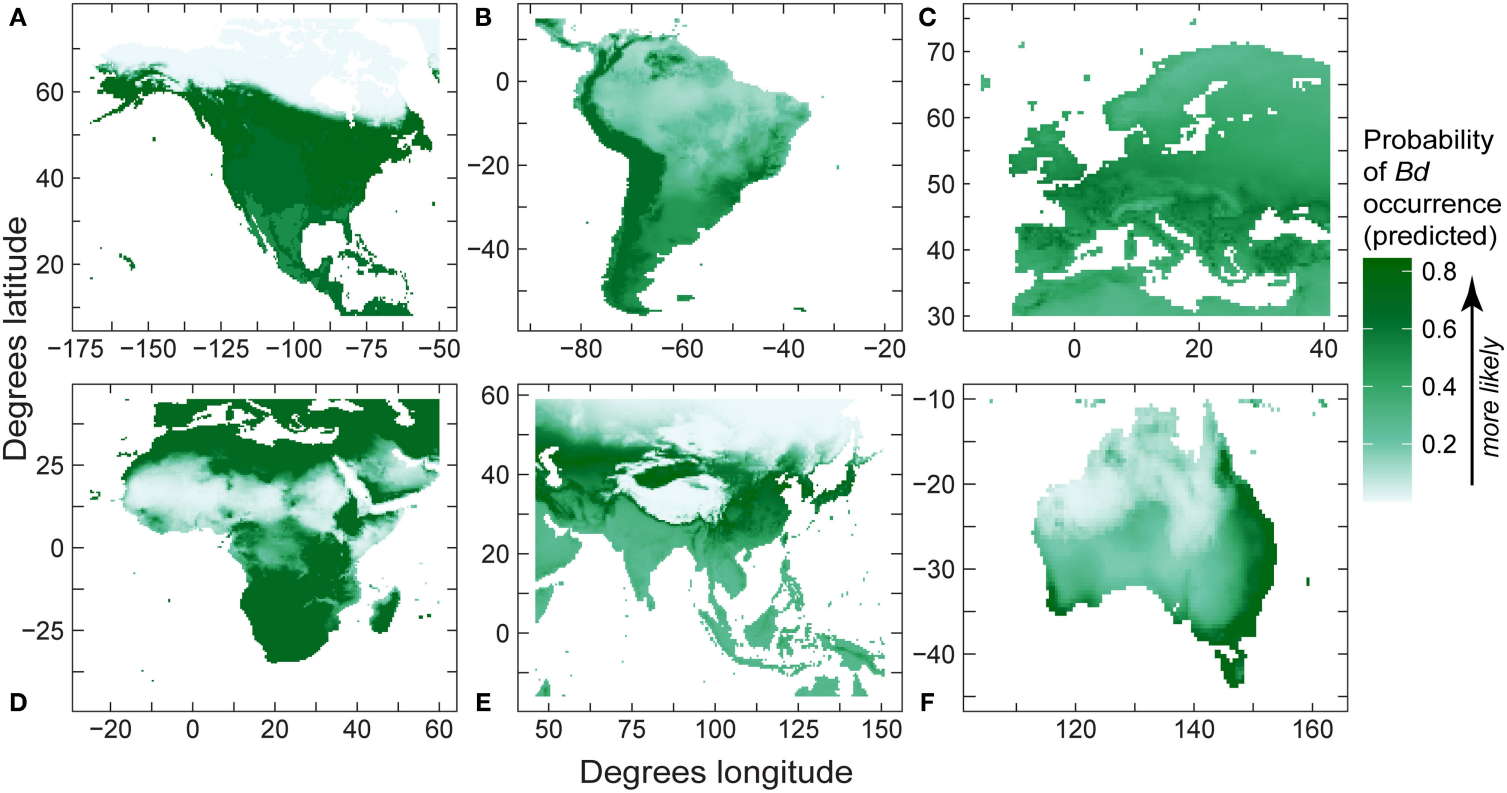
Regional predictions of *Batrachochytrium dendrobatidis* (*Bd*) absolute probability of *Bd* occurrence from our best-fit presence-absence species distribution models (area under curve values in Table 6) using *Bd* data compiled through December 2019 for: **(A)** North America; **(B)** South America; **(C)** Europe; **(D)** Africa; **(E)** eastern Asia; and **(F)** Australia.

Like regional models using detection-only data, for regional SDMs using detection and no-detection data, mean temperature was retained in all final models (Table 6), with mean temperature capturing the majority of variation in relative probability of *Bd* detection in North America, South America, and Europe (Table 6). Furthermore, mean temperature accounted for the majority (or all) of variation in *Bd* detection in Africa, Asia, and Australia (Table 6). In North America, South America, Africa and Australia, response of probability of *Bd* detection to mean temperature followed a hinge-type pattern similar to the global presence-absence model, where probability of *Bd* increased until ~0°C, plateaued, and then decreased after ~20°C. In Europe and Asia, probability of *Bd* detection to mean temperature was more typically unimodal and similar in shape to the global presence-only model: probability of occurrence increased up to ~12–13°C and then decreased. In North America, temperature range contributed a substantial portion of variation to probability of *Bd* detection (0.355; Table 6). In South America, max elevation also contributed a small portion of variation to probability of *Bd* detection (0.079; Table 6). In Australia, annual precipitation accounted for almost half of variation in probability of *Bd* detection (0.433; Table 6). Regional model predictions (probability of *Bd* detection) from the *Bd* detection and no-detection model were projected in maps (Figure 8). AUC for regional presence-absence models ranged from 0.495 to 0.724 (Table 6).

**Table 6:** Final best-fit model covariates of regional presence-absence species distribution models (North America, South America, Europe, Africa, Asia, Australia), including regional model area under the curve (AUC), and fraction of total variation accounted for (FTVA) for each variable.

**Region and final best-fit model**	**Variable**	**FTVA**
**North America (AUC** **=** **0.545)**		
*Mean temp + temp range*	Mean temp	0.645
	Temp range	0.355
**South America (AUC** **=** **0.714)**		
*Mean temp + temp range*	Mean temp	0.921
	Elevation max	0.079
**Europe (AUC** **=** **0.495)**		
*Mean temp*	Mean temp	1.00
**Africa (AUC** **=** **0.724)**		
*Mean temp*	Mean temp	1.00
**Asia (AUC** **=** **0.664)**		
*Mean temp*	Mean temp	1.00
**Australia (AUC** **=** **0.664)**		
*Mean temp + annual precipitation*	Mean temp	0.57
	Annual precipitation	0.433

## Discussion

Our results provide new insights into a pathogen that has emerged as one of the most severe threats to amphibian biodiversity (2, 34), representing a “paradigm shift in our understanding of how emerging infectious diseases contribute to global patterns of biodiversity loss” (19). Our compilation of *Bd* sampling shows increasing taxonomic scope of sampled families and species, increased incidence of species infection, and increased geographic occurrence. Below, we combine our findings with those of Castro Monzon et al. (30) to yield a more comprehensive tally of total *Bd* occurrence patterns. In addition, our analyses support emergence of new key climate predictors of *Bd* occurrence and geographic variance in climate metrics associated with the occurrence of *Bd*. These support new hypotheses for downscaled analyses of regional contexts associated with pathogen occurrence patterns and renewed efforts for species and microrefugia identification and management for more effective conservation. The large number of *Bd* occurrence reports accruing over time (Table 2 and Supplementary Table 2) points to the common aims of worldwide investigators to understand the taxonomic and geographic scope of *Bd* infections and the underlying global conservation concerns for amphibian biodiversity in the face of potential disease threat. A better understanding of linkages between these pathogen occurrence patterns and amphibian disease threats are needed across continents.

### Global *Bd* Occurrence

To gain a more comprehensive summary of *Bd* occurrence by taxonomy and geography, we compared our *Bd* data compilation through 2019 with the web search conducted by Castro Monzon et al. (30), who independently compiled *Bd* occurrences from the published literature through mid-2020. We summed unique reports from our analyses and Castro Monzon et al. (30) to calculate the total numbers of families, species, and countries with *Bd detections* from these combined datasets. Our two data compilation approaches differed in data sources. Castro Monzon et al. (30) cited 554 papers produced by their web search. In comparison, our data compilation through 2019 included 773 total sources, including sources outside the peer-reviewed literature. Together, a more comprehensive perspective is gained by combining our two approaches, but we acknowledge that even together, the compilation is incomplete; past reports that have not yet been included in these composite summaries are continually brought to our attention. Below, we also compared our compilation by taxonomy and geography to the earlier *Bd*-Maps database (28, 42) to estimate accretion of knowledge over time.

Castro Monzon et al. (30) reported *Bd* sampling across 71 amphibian families; these families were included in our compilation, although in our data compilation we added an unpublished captive report of a detection in Mantellidae (*Mantella* sp.; Supplementary Table 2), bringing the total to 72 families. Also, if the caudate family Rhyacotritonidae is included (Piovia-Scott, pers. commun.), then *Bd* is now known to have been sampled in a total of 73 amphibian families. Since 2014, 6 additional families (including Rhyacotritonidae, plus Pelodryadidae which has since split off from Hylidae) have been sampled for *Bd* (42). Compared to amphibian family tallies of *Bd* occurrence from 2014 (42), family patterns changed slightly over the ~5 years. For example, for species-level prevalence in families with over 100 species sampled, in 2014, *Bd* occurrence was highest in hylids (60%), ranids (59%), craugastorids (57%), and bufonids (44%), whereas in 2019, *Bd* occurrence was higher in hylids + pelodryadids (67%) and hyperoliids (64%), followed by ranids (58%), craugastorids (52%), bufonids (50%), and plethodontids (47%). Species tested nearly doubled for four relatively under-sampled families in 2014 (Microhylidae, Rhacophoridae, Hynobiidae, Typhlonectidae). Overall, knowledge of previously under-sampled species and families grew, suggesting there have been focal efforts targeting taxonomic knowledge gaps. Previously, amphibian family has been reported to be a strong predictor of *Bd* infection status, including severity of infection and development of chytridiomycosis (51, 83).

Castro Monzon et al. (30) reported 1,062 of 1,966 (54%) *Bd*-infected species from their web search. We report on *Bd* occurrences in 1,286 of 2,389 (54%) amphibian species, an additional 423 species but a comparable rate of infection. Upon closer comparison of these two datasets, we found that Castro Monzon et al. (30) included *Bd* sampling in 126 different, additional species (excluding hybrid species and uncertain species designations, i.e., *Genus* sp.: Supplementary Appendix 2) which were not in our data compilation. Adding these species to our total, *Bd* has been detected in 1,375 of 2,525 (55%) species sampled and compiled from both datasets. Our knowledge of world *Bd* surveillance across species has more than doubled since the 2013 paper by Olson et al. (28) where *Bd* detection was reported in 516 of 1,240 (42%) sampled species. The incidence of known species infection has increased by 13% over this relatively short time period, 2013 to 2020.

Geographically, we compared our *Bd*-in-the-wild occurrence results by country with the web search conducted by Castro Monzon et al. (30) (Supplementary Appendix 2). In comparison to our reported *Bd* sampling in 124 countries through 2019 (88 with detections), they reported sampling in 119 countries (86 with detections) through early May 2020. Our country lists differed (Supplementary Appendix 2) in that we reported *Bd* sampling in 13 countries that they did not include, and they reported *Bd* sampling from 9 countries that we did not include; hence, our datasets compiled different reports for 22 countries. Adding their 9 additional countries with 4 additional *Bd* detections to our sample (124 countries) yields 133 countries with known *Bd* sampling, with *Bd* detected in a total of 92 countries. From a very recent publication, we became aware of *Bd* sampling in one additional country that had not been included in either compilation, the Kingdom of Bhutan [*Bd* not detected (84)]. Adding this to the grand total, *Bd* has been detected in wild samples from 93 of 134 (69%) countries to our knowledge at this writing. This compares to *Bd* detections in 71 of 105 (68%) sampled countries in 2014 (42) and in 56 of 82 (68%) countries in 2013 (28).

For comparisons of site-level knowledge gain over time, using similar methods, Olson et al. (28) reported compilation of *Bd* sampling data at 4,281 sites, Xie et al. (43) reported 5,166 site-level records through June 2014, and herein we report 14,647 total sites. Site-level *Bd* data more than tripled since our initial report in 2013. In data compiled through June 2014, *Bd* sampling had occurred in 923 total USA 5^th^-field HUC watersheds, with 560 (60%) watershed having *Bd* detections (42). In comparison, by December 2019, our knowledge of *Bd* sampling had doubled across US watersheds, with 1,874 watersheds sampled, and *Bd* detections were reported for 916 (49%) watersheds.

### Environmental Associations of *Bd* Occurrences

Our analyses of environmental associations with *Bd* occurrence through 2019 further support the importance of climate-niche space for this pathogenic aquatic fungus [e.g., (28, 43, 57, 59–61, 63–66)]. In our global SDMs using the largest dataset to date with both detection and no-detection data, mean temperature was the most important environmental correlate of *Bd* occurrence, and accounted for 97% of the variation in *Bd* occurrence. In the more predictive global model using *Bd* detection-only data, mean temperature accounted for 71% of the variation in *Bd* occurrence whereas annual precipitation accounted for 22%. Although the relationship between probability of *Bd* occurrence and mean temperature (Figure 4A) is consistent with our knowledge of temperature constraints on *Bd* growth [e.g., (57)], the pattern of *Bd* occurrence with annual precipitation is not easily reconciled (Figure 4B) and may result from: a sampling artifact of *Bd* occurrence patterns in our dataset—perhaps relating to underlying, complex host-pathogen interactions with temperature; an artifact of our 0.5-degree latitude and longitude grid cells being the unit of analysis, within which heterogeneous precipitation patterns are likely; regional diversity in *Bd* environmental associations; or *Bd* lineage effects. Sampling intensity across each covariate (Supplementary Figure 1) and interactions among covariates are important considerations to fully understand the role of the different parameters across their range extents (Supplementary Figures 3A,B). Mean temperature was not the top predictor in previous presence-absence models of globally compiled data, as temperature range had previously emerged as a highly predictive covariate (28, 43). Our approach of examining correlations among similar climate metrics and consolidating to fewer potential covariates prior to SDM analyses may have contributed to this difference. Additionally, the change in surveillance patterns geographically, 2014 to 2019 (Figures 1, 3), may have led to emergence of different predictor covariates at the global level. Previously, sampling bias favoring species or locations in the United States, for example, may have led to skewed environmental associations during global assessments. With many former data gaps filled by the time of our 2019 data snapshot, this single-region bias is a lesser concern. However, the different covariates that emerged in our regional SDMs support the unique role that different climate metrics in each area may have on emerging *Bd* patterns.

Differences among regional SDMs with our more robust 2019 dataset support the importance of additional downscaled analyses to understand potential geographic context-specific patterns of *Bd* emergence. In the presence-only regional models (Table 2), the models with highest sensitivity: (1) mean temperature dominated *Bd* predictors in North America (0.91), Europe (0.83), and South America (0.67), and was a close-second predictor in Asia (0.37); (2) annual precipitation was a top predictor or close-second predictor in Asia (0.63) and Africa (0.34); (3) maximum elevation was a top predictor or close-second predictor in Africa (0.41) and Australia (0.21); and (4) temperature range was a top predictor in Australia (0.67). Per continent, scenarios could be developed to add more specificity to these regional patterns and explore their complexities. For example, the high gradients in temperature ranges across coastal-to-interior Australia were a significant contributing factor to the downscaled models already developed by Murray et al. (65). Regional patterns warrant additional study at smaller spatial scales, and relative to additional interactions among environmental covariates. At smaller scales, relationships between temperature and the pathogen biology, host biology, and their interplay could be further explored (85). Spatially downscaled approaches could have ramifications for the direction of regionally specific conservation actions to forestall disease threat, such as site-specific efforts to manage microclimate conditions (86).

### Research and Management Implications

Although *Bd* is globally distributed, occurring on every continent with amphibians, support is growing for *Bd* to have expanded its global range relatively recently (34, 36–39). Our analyses do not quantify recent spread but are an update of knowledge of *Bd* occurrence. Despite its present broad occurrence, *Bd* is clearly not ubiquitous across amphibian taxa or geographies, likely owing to a complex combination of transmission dynamics, host susceptibilities to infection, and pathogen environmental associations. Our updated *Bd*-occurrence data (downloadable from the AmphibiaWeb portal, AmphibianDisease.org: previously released public data to 2014, DOI = https://n2t.net/ark:/21547/DsA2; updated public data 2015 to 2019, DOI = https://gcc02.safelinks.protection.outlook.com/?url=https%3A%2F%2Fn2t.net%2Fark%3A%2F21547%2FDsM2&data=04%7C01%7C%7Cd307ffc12a5e407dad1e08d92ea75cce%7Ced5b36e701ee4ebc867ee03cfa0d4697%7C0%7C0%7C637592118618600952%7CUnknown%7CTWFpbGZsb3d8eyJWIjoiMC4wLjAwMDAiLCJQIjoi V2luMzIiLCJBTiI6Ik1haWwiLCJXVCI6Mn0%3D%7C1000&sdata=hBkSl%2FScrh83TNo1HeQkG7QTovBwrI3OJ8E8RblAXFY%3D&reserved=0), showed significant *Bd*-knowledge gaps have been filled across amphibian families and countries, with updated world occurrence patterns likely to inform novel research investigations and conservation actions. In combination with additional more-recent data including the independent *Bd*-data compilation by Castro Monzon et al. (30), these composite surveillance efforts are an unparalleled accomplishment by a vast global community of natural-resource managers and amphibian scientists. With about half of sampled amphibian species being infected and *Bd* occurring at <40% of sites sampled, the need for effective pathogen biosecurity-and-mitigation is paramount to reduce further *Bd* transmission and losses of vulnerable hosts.

Management actions to curtail *Bd* can take several paths. Sampled taxa and geographies without *Bd* detections warrant continued assessments for novel pathogen detection and disease threat, and consideration for heightened biosecurity to forestall human-mediated pathogen translocation pathways [e.g., global trade markets (19, 20, 34)]. Priorities for *Bd* monitoring and exclusion include sensitive species habitat strongholds, both micro- or macro-scale refugia, and broader *Bd*-free geographies that are habitat for high- or unique-diversity communities (49). Biodiverse locations of Africa, Asia, and South America with fragmented *Bd* occurrences warrant attention for elevated biosecurity to protect rich endemic fauna from potential disease threats. In more highly sampled areas, such as the USA, similar patterns of patchy *Bd* occurrences are evident at finer geographic scales (Figures 2, 3), supporting the value of downscaled biosecurity efforts to reduce human-mediated spread into current apparent host macro- or micro-refugia from the pathogen, such as *Bd*-free watersheds (Figure 3). Watersheds could be a practical spatial unit for aquatic-pathogen management (28), as a variety of water resources are often managed by watershed boundaries.

As supported by O'Hanlon et al. (34), listing *Bd* as a notifiable disease by the World Organization for Animal Health (OIE) in 2008 has had little effect on human-mediated translocation. Instead, reliance on local, regional, and national jurisdictions with *Bd*-clean trade, transportation, and fieldwork procedures is a more effective biosecurity strategy. Pathogen biosecurity approaches for wild amphibians include species- and geographic-specific risk assessment and prioritization of between-site measures that are applicable for public and natural-resource manager implementation (49, 87, 88). Enforceable regulations protecting the national heritage of non-game wildlife in separate jurisdictions could be considered for development with a focus on wildlife health, clean trade, and management of injurious invasive species [e.g., salamander import restrictions to forestall *Bsal* transmission to the USA and Canada (89, 90); inclusion of *Bd* on aquatic invasive species lists of injurious species, with hygiene measures promoted across geographic boundaries]. Biosecurity guidance is available for some types of field activities that could have broad implementation [e.g., large equipment use at field sites (48); water draws for wildfire management (55, 56)], as well as for amphibian research where within-site methods are applicable (47). These biosecurity measures transcend application to *Bd* and are relevant for cross-taxonomic pathogens, parasites, and invasive species. These actions are consistent with One Health approaches, to recognize the interconnections among people, species, and our shared environment, and to work collaboratively to optimize health of each component [e.g., CDC (91)]. Building upon the increasing public awareness of the linkages between human and wildlife pathogens and their diseases resulting from the recent coronavirus pandemic could bolster biosecurity implementation for broader One Health aims.

The value of forestalling human-mediated spread of *Bd* is several-fold. First, we are still learning about the pathogen and its complex context-dependent interactions with hosts, environmental conditions, and other threats factors [e.g., (24, 32)]. *Bd*-strain differences have emerged as a key element in host-pathogen dynamics [e.g., (34, 54, 92)]. Our current *Bd* dataset does not record *Bd* strains, which sets a new challenge for the global community conducting surveillance to contribute strain data to the next phase of the *Bd* web portal (41). Also, there is increasing information about the role of microbiotic community interactions on amphibian skin, with some bacteria having antifungal properties that afford protection to host amphibians from adverse effects of *Bd* infections (93–96). Despite advances in understanding amphibian immune responses, this is an active area of ongoing research that is likely to offer new insights to *Bd* control (97–100). Such ongoing research interleaves with novel *Bd*-management opportunities (e.g., microbiota vaccines) and could help forestall mass-mortality events in susceptible species. In situations where at-risk taxa appear threatened, the ability to develop rescue measures and learn from their efficacy can inform later efforts [e.g., (101, 102)]. Furthermore, relative efficacy of additional conceptual field mitigations for amphibian EIDs have been qualitatively evaluated but warrant field trials to see if they can alter site-scale *Bd* infection dynamics [e.g., habitat attributes such as shading and water temperature management—essentially microclimate manipulations—could alter site-scale *Bd* infection dynamics (86)]. Adaptive management and learning from such field trials is needed to advance effective mitigations with knowledge of the risks and benefits they may entail. Each intervention that might safeguard species from severe infection merits study for efficacy and practicality as part of research and conservation trials, while biosecurity measures could stall inadvertent spread.

### Next Steps

Our newly updated dataset points to the broad human dimensions of *Bd* surveillance, and specifically the contributions to our current understanding of global *Bd* occurrences from a broad world community. Our 769 data sources show international partnerships have been established between local faunal and land-management experts and personnel from numerous universities and institutions to pursue *Bd* surveillance (Supplementary Table 2). Such co-production underscores the local and global interest in *Bd* occurrence, spread, and threat. Although published data from peer-reviewed journals dominate our compilation of data sources (86%), incentives are needed to improve this rate to ensure data quality assurance of sampling and analytical procedures. Publishing could be promoted prior to graduate student defenses, or with permitting procedures.

Communication of the global *Bd* database move to AmphibianDisease.org and encouragement of ongoing project plans as well as data imports is important going forward, as metadata analyses of global data compilations can be important for hypothesis testing and pattern revelations [e.g., (50–53)]. The consistency of the results presented here from joint web-portal data imports compilation and literature searches was comparable to a more technical web search of journals (30). Clearly, the combined approaches yield a more comprehensive picture, and may be useful for a more complete *Bd* dataset at AmphibianDisease.org into the future.

With initiation of the new web portal AmphibianDisease.org, we anticipate phases of *Bd* database updates over the near term. First, as novel *Bd*-data imports have been made already to the new portal from early users and local projects, cross-checking between the *Bd*-Maps database upload and these datasets will be needed to reconcile redundancies. Data gaps discovered from 2019 and earlier should be addressed as they are identified, including data sources uncovered by Castro Monzon et al. (30) that we had not included in our data compilation for this paper. Ongoing efforts to educate researchers on new procedures to archive their data will be critical. The Amphibian Disease database may include records of captive animals and museum specimens, allowing unpublished data to be added. Additionally, *Bd* occurrence patterns derived from eDNA or fomite samples provide additional data sources requiring special attention for compilation, especially as new multi-taxonomic community-based analyses are conducted [e.g., (103)]. Analyses of *Bd* samples for genetic variants is a needed technological bridge, expanding basic *Bd* surveillance and monitoring objectives to another level of specificity. Innovative collaborations among diagnostics laboratories and amphibian researchers in a variety of subdisciplines is likely needed to meet this objective. In addition to pathogen strains, virulence parameters could be included in the updated database, addressing new research priorities to understand pathogen demographics, pathogenicity, and disease dynamics (19, 34, 92). Most importantly, expanding the new portal to identify chytridiomycosis occurrences, rather than simply the pathogen *Bd*, is a key goal.

The Amphibian Disease database has new web applications compared with *Bd*-Maps.net (41). Importantly, user-friendly import and export capacities have been a priority in portal development. Data can be uploaded by “projects” for both *Bd* and *Bsal* studies. Data for a project can be assigned a DOI. Koo and Olson (41) explain further how AmphibianDisease.org can tap into additional online databases via their network partnerships, such as genetic and genomic public databases.

As *Bd* data accumulation accelerates, a corresponding increase in the depth of knowledge of species status and threat occurs, echoing calls for conservation urgency. As *Bd* chytridiomycosis appears to be about a half-Earth pandemic across amphibian taxa and sites, there is considerable room for action from both bottom-up community-run efforts and top-down national-to-international policies having importance.

## Data Availability Statement

The raw data supporting the conclusions of this article will be made available by the authors, without undue reservation.

## Ethics Statement

Ethical review and approval was not required for the animal study because no live animals were used for this research, we only summarized existing datasets.

## Author Contributions

DO designed research and led manuscript development. KR compiled and summarized data, drafted figures and tables, and developed Supplementary Material. CG conducted statistical analyses. KC developed geographic information for analyses and drafted figures. AB assisted with logistical support and manuscript development. All authors provided critical feedback and contributed to the final manuscript.

## Conflict of Interest

The authors declare that the research was conducted in the absence of any commercial or financial relationships that could be construed as a potential conflict of interest.

## Supplementary Material

Supplementary Appendix 1 Detailed methods.

Supplementary Appendix 2 Comparing the current study with results from Castro Monzon et al. (30).

Supplementary Figure 1 Relative habitat suitability of *Batrachochytrium dendrobatidis* (*Bd*) occurrence (probability ratio output) for each environmental attribute from the global presence-only best-fit species distribution model. Each environmental attribute [**(A)** mean temperature; **(B)** annual precipitation; **(C)** temperature range; **(D)** maximum elevation] marginal-response plot is calculated while holding all other covariates at the mean. The frequency of observed presence (FOP) plots (black dots, orange line estimating trend) show the number of sites with *Bd* occurrence across the range of the explanatory variable (e.g., the frequency of *Bd* occurrence points increases as max elevation increases, until about 4,000 m). The kernel estimated data density (light gray background) shows the sampling effort.

Supplementary Figure 2 Absolute probability of *Batrachochytrium dendrobatidis* (*Bd*) occurrence for the one environmental attribute (mean temperature, °C) from the global presence-absence best-fit species distribution model. The frequency of observed presence (FOP) plot (black dots, orange line estimating trend) shows the number of sites with *Bd* occurrence across the range of the variable. The kernel estimated data density (light gray background) shows the sampling effort.

Supplementary Figure 3 Marginal-response plots [*N* = 24; 18 shown in **(A)**, 12 shown in **(B)**] depicting interactions of covariates from the best-fit presence-only global species distribution model of *Batrachochytrium dendrobatidis* (*Bd*) occurrence using detection-only data for 0.5-degree latitude/longitude grid cells. The probability ratio output (PRO, model predictions) is calculated for a range of the environmental covariate (named below plot) while holding the interacting covariate (named above plot) at the 0.25 percentile (left), mean (center), and 0.75 percentile (right). Non-interacting covariates per plot are held at their mean.

Supplementary Figure 4 Marginal-response plots (*N* = 6) depicting interactions of covariates from the best-fit presence-absence global species distribution model of *Batrachochytrium dendrobatidis* (*Bd*) occurrence, using detection and no-detection data for 0.5-degree latitude/longitude grid cells. The predicted probability of *Bd* occurrence is calculated for a range of the environmental covariate (named below plot) while holding the interacting covariate (named above plot) at the 0.25 percentile **(Left)**, mean **(Center)**, and 0.75 percentile **(Right)**.

Supplementary Table 1 Summary of *Batrachochytrium dendrobatidis* (*Bd*) detection (+) and no-detection (–) records and sites in the Global Bd Mapping Project (*Bd*-Maps) database through December 2019.

Supplementary Table 2 *Batrachochytrium dendrobatidis* detections by species, with references and countries of detection.

Supplementary Table 3 *Batrachochytrium dendrobatidis* (*Bd*) detections by country, with source citations (see Data References following Supplementary Table 2).
