# Corrigendum: Global Patterns of the Fungal Pathogen *Batrachochytrium dendrobatidis* Support Conservation Urgency

**DOI:** 10.3389/fvets.2022.825058

**Published:** 2022-02-08

**Authors:** Deanna H. Olson, Kathryn L. Ronnenberg, Caroline K. Glidden, Kelly R. Christiansen, Andrew R. Blaustein

**Affiliations:** ^1^Pacific Northwest Research Station, United States Department of Agriculture (USDA) Forest Service, Corvallis, OR, United States; ^2^Department of Biology, Stanford University, Stanford, CA, United States; ^3^Department of Integrative Biology, Oregon State University, Corvallis, OR, United States

**Keywords:** amphibian chytrid, *Bd*, climate associations, emerging infectious disease, fungal pathogen

In the original article, the word “not” was inadvertently omitted in the **Results** section, page 5, column 2, line 7. The correct text should read “and *Bd* has not been detected (J. Piovia-Scott, Washington State University, Vancouver, WA, USA; pers. commun.)” and the corrected paragraph appears below.

**RESULTS**, **Taxonomic Patterns**, Paragraph 1

Through 2019, our world *Bd* data compilation showed that *Bd* had been detected in 1,294 of 2,412 (54%) amphibian species sampled, and that sampling had been conducted in 29% of all amphibian species (Table 2 and Supplementary Table 2). Anurans (frogs and toads) had the highest species-level prevalence of infection (54.7%) compared to caudates (newts and salamanders: 49.2%), and gymnophionans (caecilians: 29.2%). Through 2019, there were *Bd* detections in 86% of amphibian families. *Bd* surveys have been reported for all amphibian families except one anuran family (Nasikabatrachidae, 2 spp.: Western Ghats, India); one caudate family (Rhyacotritonidae, 4 spp.: Pacific Northwest USA); and one gymnophionan family (Chikilidae, 4 spp.: Northeast India) (Tables 2, 3). However, we are aware that in ongoing experiments of *Bsal* susceptibility in USA salamanders, wild-caught members of Rhyacotritonidae have been screened for *Bd* prior to use in laboratory trials, and *Bd* has not been detected (J. Piovia-Scott, Washington State University, Vancouver, WA, USA; pers. commun.). Species-level *Bd* prevalence among families was highly variable (Table 3). Through 2019, 6 of 55 (11%) Anura families and 3 of 9 (33%) Gymnophiona families had no *Bd* detections among sampled species (Tables 2, 3).

The authors apologize for this error and state that this does not change the scientific conclusions of the article in any way. The original article has been updated.

## Publisher's Note

All claims expressed in this article are solely those of the authors and do not necessarily represent those of their affiliated organizations, or those of the publisher, the editors and the reviewers. Any product that may be evaluated in this article, or claim that may be made by its manufacturer, is not guaranteed or endorsed by the publisher.

