# Supplementary material for: Global Patterns of the Fungal Pathogen Batrachochytrium dendrobatidis Support Conservation Urgency
Source: Front Vet Sci. 2021 Jul 16;8:685877. doi: 10.3389/fvets.2021.685877 (PMC8322974; doi:10.3389/fvets.2021.685877)
Supplement: Supplementary Appendix 1 — Detailed methods. [file Data_Sheet_1.zip › Data Sheet 1/Appendix S1.docx]

**Frontiers in Veterinary Science, section Zoological Medicine**

**Special Issue: Emerging Infections and Diseases of Herpetofauna**

**Hosted by Amanda L.J. Duffus; Rachel E. Marschang**

**Global Patterns of Fungal Pathogen *Batrachochytrium dendrobatidis* Support Conservation Urgency**

Deanna H. Olson^1^*, Kathryn L. Ronnenberg^1^, Caroline K. Glidden^2^, Kelly R. Christiansen^1^, Andrew R. Blaustein^3^

^1^U.S. Department of Agriculture, Forest Service, Pacific Northwest Research Station, Corvallis, Oregon, USA

^2^Stanford University, Department of Biology, Stanford, California, USA

^3^Oregon State University, Department of Integrative Biology, Corvallis, Oregon, USA

**Appendix S1: Methods**

Methods are provided for transparency in database development, and to inform future database advances. Methods are presented in three sections, each with their own references: Data Compilation Processes; Taxonomic Notes; and Environmental Associations with *Bd* Occurrence. At the end of the appendix are the Data Field Definitions for the database.

1. **Data Compilation Processes**

**Literature Search**

To compile data from the published, peer-reviewed literature, we searched the journal archives of an extensive list of journals known to have published papers on *Bd*, using the search terms “chytrid”, “*Bd*”, and “amphibian”. Where possible, journal publishers’ main sites and services such as JSTOR and BioOne were searched to capture articles from any journal that outlet might publish. Additional papers were tracked down by cross-checking the reference sections of the papers found from the first process. These reference checks also produced a trove of non-peer-reviewed sources such as agency reports and theses and dissertations, which were duly tracked down and recorded as well.

**Data Acquisition**

The original *Bd*-Maps database included data compiled by regional contributors, uploaded by individual authors directly to the online database, or sent to us as digital files. The current update consists mainly of data pulled from publications through December 2019, some of it downloaded as supplemental data files, plus one large contribution of data relating to four studies from Jessa Watters’ laboratory at the University of Oklahoma; two of these were still in press when data were sent, but all have subsequently been published in peer-reviewed outlets (Watters et al. 2016, 2018, 2019; Marhanka et al. 2017; see references for Table S2).

For downloaded or contributed data in spreadsheet or CSV formats, the record-building process commenced with reformatting and adding fields to the existing data rather than starting-from-scratch with data tables in a publication and empty spreadsheet template rows, but the decision process is the same for both types of data sources. An important note: when digital data were available as individual records for every animal sampled, sometimes these were retained as single records, but more often they were summarized to one record per location per species per sampling occasion (sex, life stage). The rationale was that these data were originally intended for mapping and geospatial analysis products that require that even the record data be distilled down to site level to avoid the inevitable autocorrelation that would result from hundreds of stacked points. Also, this project began before qPCR was common, and even now many authors do not report zoospore loads for every sample. Thus, there was no real need to keep an individual record of every sample, and it made the digital data more easily comparable to the data collated from the text and tables of publications, which are always summarized in this manner. Finally, if we’d kept every single record, the data files would have been unmanageably large—earlier versions of MS Excel already struggled to sort them.

**Data Formats**

The original *Bd*-Maps interface provided dropdown menus for some data fields, but source data QA/QC and coordinate precision were unknown or not provided.

If we had known at the inception how this database would develop, we might have done things differently (e.g., including a coordinate error field in meters, including a range of elevation fields, having separate verbatim coordinate fields for any coordinates that were converted). A field for coordinate error in meters has since been added, along with fields for georeferencing sources and remarks, to reflect available Darwin Core fields in the new data portal and capture some of our existing data that didn’t match those available fields perfectly. For details of how data fields from the original format map to the Darwin Core fields that will show up on the new portal, see **Section IV.** **Data Field Definitions**.

**Record-building Decision Tree**

The database includes studies published up through December 2019. Some published studies provided very good breakdowns of results by species and location, with coordinates for study site locations (the ideal case); others did not. Because we wanted to make sure that all species and all locations that were tested for *Bd* were included, we developed additional record types to cover all contingencies. The process for deciding how to represent the published results followed a decision tree based on the information provided in the paper.

**Note:** Data field names in original data formats are shown in **BoldfaceBlue**; new Darwin Core names for equivalent or successor fields follow them in [square brackets, in **BoldfaceOrange**] at first occurrence.

1. Was the research (a) a sampling study (field or museum specimens), or was it (b) an experimental field or laboratory study?
   1. Go to step 2
   2. Did the experimental study collect animals for the experiment from the wild?
      1. NO – study was not included in the database – STOP. No data to include.
      2. YES – Go to step 2
2. Were coordinates provided for the study sites or collection sites (for museum or experimental specimens)?
   1. YES – Go to step 3
   2. NO – Was other information such as a location name, description, or map provided?
      1. YES – Go to step 7 Geocoding
      2. NO – Go to step 9 Centroid/Region Records
3. Were coordinates provided as decimal degrees latitude/longitude? **Note:** The number of decimal places coordinates are reported to affects the precision that can be claimed for a location. [See notes in **Section IV. Data Field Definitions**]
   1. YES – Assign the **Coordinate Source** [= part of **georeferenceProtocol**] value as “DDLL” for decimal degrees latitude/longitude. Go to step 10 Coordinate Accuracy and Error.
   2. NO – Go to step 4 Coordinate Conversion Methods

Coordinate Conversion Methods

1. Were coordinates provided as degrees and decimal minutes or degrees/minutes/seconds ? **Note:** The number of decimal places coordinates are reported to affects the precision that can be claimed for a location. [See notes in **Section IV. Data Field Definitions**]
   1. YES – Convert to decimal degrees lat/long using spreadsheet formulas or the conversion utility on the FCC website: <https://www.fcc.gov/media/radio/dms-decimal>. Assign the **Coordinate Source** [= part of **georeferenceProtocol**] value as “DMS” for degrees/minutes/seconds.
      1. **Note:** assume coordinates are WGS84 datum, but check just in case they’re in a national datum of some kind or the older NAD27 for North America.
      2. If coordinates are in a national datum or NAD27 they will need to be converted in a GIS (e.g., Australian National Datum)
   2. NO – Go to step 5
2. Were coordinates provided in the UTM (Universal Transverse Mercator) system?
   1. NO – Go to step 6
   2. YES – Assign the **Coordinate Source** [= part of **georeferenceProtocol**] value as “UTM”. Is the zone provided?
      1. YES – go to step 5c
      2. NO – using a UTM zone map of the world and any map or location information in the paper, determine the proper UTM zone; then go to step 5c
   3. Is the datum WGS84?
      1. YES – convert to decimal degrees lat/long using an online conversion utility, <http://home.hiwaay.net/~taylorc/toolbox/geography/geoutm.html>, or in a GIS for large data files, then go to step 10 Coordinate Accuracy and Error
      2. NO – convert to decimal degrees lat/long in a GIS, then go to step 10 Coordinate Accuracy and Error
3. Were coordinates provided as township/range/section US Public Lands survey locations?
   1. YES – Assign the **Coordinate Source** [= part of **georeferenceProtocol**] value as “TRS” for township/range/section. Convert to decimal degrees lat/long using an online utility such as <https://www.usna.edu/Users/oceano/pguth/md_help/html/get_plss.htm>. You may need to know or determine the appropriate meridian. With coordinates converted, go to step 10 Coordinate Accuracy and Error
   2. NO – Coordinates were provided in a system that was none of the above. Determine what system was used (e.g., Swedish National Grid, Luxembourg National Grid). Coordinates will need to be converted in a GIS. Can conversion parameters be found for the stated grid system?
      1. YES – Convert coordinates to decimal degrees lat/long (e.g., Swedish National Grid). All coordinates of this type have so far been reported in meters, in the style of UTM coordinates. Assign the **Coordinate Source** [= part of **georeferenceProtocol**] value as ‘UTM’, but note also as part of the remarks what grid system the coordinates were converted from. Then go to step 10 Coordinate Accuracy and Error.
      2. NO – If no conversion parameters can be found in GIS (e.g., Luxembourg National Grid), the locations may need to be geocoded using other information provided. Go to step 7 Geocoding.

Geocoding

No coordinates were provided, but some other information is available to tie sampling results to a location.

1. Were location names or descriptions given in a data table or spreadsheet file, associated with test results?
   1. YES – From these location names or descriptions, determine coordinates as accurately as possible by geocoding using any combination of the following sources, as applicable:
      1. Google Earth, Google Maps, MapCarta, TopoZone, and national topographic map servers and gazetteers (e.g., QTopo for Queensland, Australia)
      2. GNIS feature search, for US place names—the US Board on Geographic Names information server: <https://www.usgs.gov/core-science-systems/ngp/board-on-geographic-names/domestic-names>
      3. online maps of parks, reserves, and natural areas
      4. any map published in the paper, or maps of the same study area published in other works
      5. coordinates published in other research at the same site, which may be cited in the paper or can be found by online searches
      6. searching for the place name in a search engine, to guide map searches
      7. One or more of these sources may provide coordinates directly; otherwise, use source material to pull location coordinates from Google Earth
      8. **Note:** sources for each location were not recorded at the time of geocoding. In many cases, they have been added (as best could be determined) to the data after the fact in the **georeferenceProtocol** field in the new format. Any notes on the precision of the coordinates originally recorded in the **Notes** field have been added to the **georeferenceRemarks** field.
      9. Assign the **Coordinate Source** [= part of **georeferenceProtocol**] value as ‘GEOCD’. **When coordinates have been determined, go to step 10** Coordinate Accuracy and Error
   2. NO – go to step 8
2. Was a map showing the sampling locations published in the source paper?
   1. YES – Did the map include enough context information to make it possible to geocode coordinates using a combination of sources such as Google Earth, TopoZone, MapCarta, and other online resources?
      1. YES – Use context information such as site labels, feature names, park boundaries, topography, streams, or roads to geocode coordinates in Google Earth. Assign the **Coordinate Source** [= part of **georeferenceProtocol**] value as ‘MAP’. Go to step 10 Coordinate Accuracy and Error
      2. NO – Can coordinates be determined for the general vicinity covered by the map?
         1. YES – Geocode generalized coordinates in Google Earth; location will have to be assigned a less precise **CoordinateAccuracy** and a larger **CoordinateError.** Assign the **Coordinate Source** [= part of **georeferenceProtocol**] value as ‘MAP’. Go to step 10 Coordinate Accuracy and Error
         2. NO – go to step 9 Centroid/Region Records
   2. NO – go to step 9 Centroid/Region Records
3. Centroid/Region Records. Presumably the publication reports some very basic information about the location of the sample.

Is location reported to state/province/department level?

- 1. YES – In Google Earth, geocode coordinates from the center of the regional subdivision. This record will be assigned a **CoordinateAccuracy** value of ‘Region’. Assign the **Coordinate Source** [= part of **georeferenceProtocol**] value as ‘GEOCD’. Go to step 10 Coordinate Accuracy and Error.
  2. NO – Location is reported only to the level of the country sampled. This record will be assigned **CoordinateAccuracy** value of ‘Country Centroid’. Assign the **Coordinate Source** [= part of **georeferenceProtocol**] value as ‘GEOCD’. Consult existing data to determine if a country centroid coordinate and **LocationNumber** have already been assigned to this country. If so, record these values; if not, geocode coordinates for the center point of the country in Google Earth. Go to step 10 Coordinate Accuracy and Error.

Coordinate Accuracy and Error

Because almost none of the data sources reported measured or estimated error values for the coordinates they provided, and we had no way to determine how precise their coordinates were or how they obtained them, the original conception of the **CoordinateAccuracy** field was as a categorical value that gave us an idea of how close to the on-the-ground site of sampling the coordinate would place you, so that we could sort and select the more accurate categories for geospatial analysis. The **CoordinateAccuracy** field has now been folded into the Darwin Core field **georeferenceRemarks** for future iterations of the data. See **Section IV. Data Field Definitions** for specifics.

1. Is the location for a conservation-sensitive species that should not be made public with any precision?
   1. YES – Assign coordinates a **CoordinateAccuracy** value of ‘Fuzzy’. The latitude and longitude should be deliberately changed (either made much less precise, such as decimal degrees recorded to only one decimal place, or a location deliberately chosen to be safely distant from the real site) to mask a sensitive location. Go to step 12, Coordinate Error.
   2. NO – Go to step 11.
2. Were the coordinates taken from the exact point of the amphibian sampling site, though not necessarily of the individual animal sampled? OR For coordinates reported in decimal degrees, were coordinates reported to 3 or more decimal places?
   1. YES – Assign coordinates a **CoordinateAccuracy** value of ‘Exact’ based on the Data Field Definitions. Go to step 12, Coordinate Error.
   2. NO – Do the latitude and longitude represent a position close to where the samples were collected, but not the exact site (such as one end of a transect), OR Were coordinates reported in degrees/minutes/seconds given only to degrees and minutes?
      1. YES – Assign a **CoordinateAccuracy** value of ‘Approximate’. Generally, error should be less than 2 kilometers. Go to step 12, Coordinate Error.
      2. NO – Do the latitude and longitude denote the general vicinity of the sample, but are estimated and may be up to several kilometers off? This could include centroids of a county or municipality.
         1. YES – Assign a **CoordinateAccuracy** value of ‘Vague’. Go to step 12 Coordinate Error.
         2. NO – Do these coordinates represent a larger area such as the range of a species in the sampled country, or a 1^st^-level political division (state/province/department)?
            1. YES – Assign a **CoordinateAccuracy** value of ‘Species’ for the former, ‘Region’ for the latter. Go to step 12 Coordinate Error.
            2. NO – Are these coordinates accurate only to the level of the country sampled?

YES – Assign a **CoordinateAccuracy** value of ‘Country Centroid’. Go to step 12, Coordinate Error.

NO – Do these coordinates represent several sampled countries or a continent? Assign **CoordinateAccuracy** value of ‘Species’. Go to step 12, Coordinate Error.

1. Coordinate Error

For the reasons stated under Coordinate Accuracy and Error above, most data were not published with a measure of coordinate error in meters. In addition, we did not measure error for coordinates at the time that we geocoded them, preferring the simpler categorical assessment of **CoordinateAccuracy**. We added the **Coordinate Error** [= **coordinateUncertaintyInMeters**] field after the fact to better reflect Darwin Core data specifications in the new format. Consequently, this field is often blank, or is based on an estimate from the apparent precision of reported coordinates in decimal degrees or DMS. Decimal degrees reported to 2 decimal places were assigned an 800-m error; to 3 decimal places, a 110-m error. Coordinates given to only degrees and minutes in DMS were assigned a 1100-m error. ‘Fuzzy’ coordinates were assigned a 10000-m error (10 km). For a few of the most recent increment of data entered, values were measured for geocoded coordinates.

When **Coordinate Error** has been assigned, go to step 13 Coordinate QA/QC.

1. Coordinate QA/QC

Examine recorded coordinates, especially those reported by the source as decimal degrees lat/long or converted from reported DMS, for potential errors. If possible, map each coordinate in Google Earth (you may already have done this as part of geocoding) and have an eye to political boundaries, natural features, and place names. Do they plot in the right place? Clearly in the wrong place, such as in the wrong country or far out at sea? Fail to plot at all? Potential sources of error include:

- 1. Either lack of or an extra negative sign in the latitude or longitude: point might plot in the wrong hemisphere.
  2. A misplaced or extra decimal point: point might plot outside the lat/long grid or fail to plot.
  3. Coordinate conversion errors based on mistakes in recording GPS coordinates, for instance having set the GPS to record degrees and decimal minutes, but reporting the results as decimal degrees, or if coordinates are reported in DMS, recording degrees and decimal minutes, but reporting the results as degrees, minutes, and seconds. A clue here would be any value greater than 60 for minutes or seconds.
  4. UTM coordinates converted by the author, but using the wrong zone (or more subtly, the wrong datum): an instance of this problem landed a point supposedly from Idaho out in the Pacific Ocean, but it is not always quite that obvious.
  5. A typo omitting a digit.
  6. Author error, duplicating the same coordinate for two sites.

Errors a and b are fairly easy to spot and fix. Types c and d can sometimes be corrected by back-calculation and recalculation, but along with types e and f, may require consultation with the data originator to sort out.

With large datasets that may have hundreds of locations, rather than plot them individually in Google Earth, this process may have to wait for points to be pulled into a GIS and mapped to look for obvious errors, though they’re then usually more difficult to spot.

If erroneous coordinates cannot be corrected, it may be necessary to geocode a less accurate location based on whatever locality information is available, or even to drop the record, if it’s not a new or critical report. When coordinates have been checked, continue to Record Building.

Record Building

At this point, zoom out to the source level to enter background information.

Start a data line by filling in the **Permission, Data Source Type, Citation**, and **Contact** fields. If the data came from a published source, **Permission** will be coded as ‘Public’; if submitted directly from or by a principle investigator, they may have asked to have it coded ‘Private’; one working group coded their data ‘RACE’. **Data Source Type** is the origin of the data: choose from Journal Publication, Thesis or Dissertation, Report (gray literature), Email communication (sent to us directly, usually unpublished data), Fieldwork (input by data owner), Map, or Newspaper. **Citation** is the short reference to the publication, in the format Smith 2004, Garcia and Smith 2005, or Garcia et al. 2008. **Contact** [**= principalInvestigator**] is the originator of the data, the contact listed in the publication. Usually includes the email address, with [at] substituted for the @. For the new platform, we added **Reference** [**= associatedReferences**]**,** which is the citation concatenated with the full reference to the source publication; **Data Source Type** and **Citation** are omitted as duplicating that information.

Also, if consistent for all data in the publication, fill in the **Method** [**= testMethod**] field; if the method varies by sample, this can be done later. Allowed values: Histology, PCR, qPCR, rtPCR, Isolation, Visual, or combinations of these methods. If available or desired, fill the **Laboratory** [**= diagnosticLab]** field with the name of the lab where *Bd* testing was carried out.

Copy these data to sufficient lines in the database to have one line per site, and fill in the location fields: **Latitude** [= **decimalLatitude**], Longitude [= **decimalLongitude**], Elevation [= Elevation] in meters (if given), **Coordinate Source, Coordinate Accuracy** [= **georeferenceRemarks**], and **Coordinate Error** [**= coordinateUncertaintyInMeters**]. Add location descriptive information in the **Location** [**= locality**] field and populate the **Continent** [= **continentOcean**], **Country**, and **Region** [= **stateProvince**] fields for each location as outlined in **Section IV. Data Field Definitions**.

Then continue below.

1. Can sampling results reported for species be tied directly to site coordinates?
   1. YES – Create full records. Copy location information for each sampling site to sufficient data lines to account for all species sampled at that site. Provisionally enter the reported genus and species (or where applicable, sp., spp, etc.) in the **Genus** [= **genus**] and **Species** [= **specificEpithet**] fields, and populate the other species-related fields, including higher-level taxonomy (**Order, Family**) and qualifiers (cf., aff., kl.) if known or reported. If the species is listed in the source with ‘cf.’, ‘aff.’, or ‘kl’, enter numeric values for these qualifiers in the **Cf/Aff/Kl** field [no equivalent in new format]: 1 = cf., 2 = aff., 3 = kl. Also spell out full provisional name in **Synonyms** field. For **NatInt** [= **establishmentMeans**], values are “Native” if the species sampled was native to the country or region in which it was found; “Introduced” if it was not. **WildCapt** [= **originalSource**] should be “Wild” if animals were sampled or collected in the wild, or in an outdoor setting where they could have easy contact with wild animals (such as outdoor holding facilities on farms), or “Captive” if animals tested were indoor captives with no easy contact with wild animals, but which could potentially have contact with other animals of the same or different species in a captive setting. This includes animals from zoos, pet shops, and markets. Record whether the samples came from a museum collection in **MusSpec** [= **basisOfRecord**]: “Yes” [PreservedSpecimen] if they were, “No” or blank [LivingSpecimen] if they were not. Repeat for all sites reported. Go to step 15.
   2. NO – Can sampling results reported for species be reconnected indirectly to site coordinates?
      1. YES – Sometimes this is relatively straightforward, but in other cases, this process may require a lot of prep work, involving tables of location data, tables of species data that don’t match up one-to-one, intermediate variables that tie the two together, or consulting the publication map or graphs of test results to put species results and sites back together. It may be possible to contact the author and ask for help with this process, but sometimes you just have to decide if it’s worth the often-intense effort. If yes, go back to step 14a and on to 15; if no, go to step 16 Location + Species Records to create location records and associated species records.
      2. NO – Go to step 16 Location + Species Records to create location records and associated species records.
      3. **Note**: some sources reported locations for species (or individuals) that tested positive but did not report the locations for negative tests; this results in a combination of full records and location/species records for the study.
2. For each species sampled, were data reported for more than one occasion and/or for more than one sex or life stage for at the same site?
   1. YES – Copy full line to yield an individual record for each species/sex/life stage at each site on each sampling occasion (can be single date or sampling period). Populate the Sex and Life Stage fields, then populate the date fields with the span of each sampling occasion, when sampling started (**StartDay, StartMonth, StartYear**) and ended (**EndDay, EndMonth, EndYear**). (If sampling occurred on one day only, Start and End day/month/year will be the same.) Go to step 18 Test Results
   2. NO – Populate the date fields with the span of each sampling occasion, **Day, Month**, and **Year** sampling started and ended. (If sampling occurred on one day only, Start and End day/month/year will be the same.) Go to step 18 Test Results
3. Location + Species Records. Create location records and a parallel set of species records, beginning with the location records. Were results reported separately for more than one sampling occasion at one or more sites?
   1. YES – Copy location information for each sampling site to sufficient data lines to account for all sampling occasions reported (can be single date or sampling period), plus one extra record to serve as a template for the companion species records. Populate the date fields as in step 15.

Are results reported by species?

- - 1. YES ­– To create species records, copy the extra template record to create a data line for each species sampled, removing the location information including **Location** [= **Locality**], Latitude [= **decimalLatitude**], **Longitude** [= **decimalLongitude**], **Elevation** [= **Elevation**] in meters, and the three Coordinate fields [or their equivalent]. In the associated location records, populate any taxa fields that are not shared across all records with <multiple>. (Example: if the study sampled only frogs, **Order** could be Anura, but **Family**, **Genus**, and **Species** would be <multiple>.) Carefully crosscheck data to discover if one or a few species’ test results can be assigned to a particular location and make full records for these, as in 14a. If none, or once this has been done, go to step 17 Species Records.
    2. NO – Delete the extra record; there will be no species recorded for these locations. Populate empty taxa fields with <unknown>, leaving **Synonyms** blank. Go to step 18 Test Results.
  1. NO – Copy one extra record to serve as a template for the companion species records. For each location record, populate the date fields as in step 15. Go to step 18 Test Results.

1. Species Records. Create generalized coordinates for species records based on the locations of the sites in the associated location records. This can be done in several ways, depending on the locality information and the number of sites. If a place name is available for the general area of the study (such as a park or reserve name or a political division), coordinates can be geocoded from the center of the area. Alternatively, the known site coordinates can be plotted in Google Earth, and a weighted centroid of the polygon can become the generalized coordinate. A similar process could be used by eye if a map of the sites is included in the publication. Once this coordinate is established, fill in the georeferencing-related fields as in steps 10–12, with **Coordinate Source** = GEOCD and **Coordinate Accuracy** = ‘SPECIES’. In the **Location** [= **Locality**] field, devise a name for the generalized coordinate that reflects the process used to derive it, followed by the warning “species record NOT FOR GEOSPATIAL ANALYSIS”, which should also be entered in the **Notes** field in the old format, or the **georeferenceRemarks** field in the new format. Then make a copy of the template record for each sampled species and populate the taxa fields as in step 14. If there was only one sampling occasion, populate the date fields with a generalized set of dates that covers the whole sampling period; if results are reported for more than one sampling occasion per species, copy each species record as many times as needed, and populate the date fields appropriately. If more than one sex/life stage is reported, create additional records as needed. Go to step 18 Test Results.
2. Test Results. Depending on how data are reported, test results may be broken down by site, species, sex, life stage, and/or occasion, reported by site separate from species and occasion, or by site and occasion separate from species, or some permutation of the last two. Via the above steps, the correct number of records have been created to reflect all the reported data. In the original data format, the results of the test are summarized in the **Test P/N/U** [= **diseaseDetected**] field: **P** if *Bd* was detected in some or all of sampled animals; **N** if *Bd* was not detected in any of the sampled animals represented by this record; **U** if test result was equivocal or there is reason to doubt the result. Four numeric fields follow—**BdDet, BdUnc, BdNeg, Tested**—that record the number of samples that tested positive, had an equivocal or uncertain result, or tested negative, plus the total samples tested. Some of these fields may be blank if results were not reported by species for a set of sample sites in a published map or table; if a species is reported as positive or negative at a site but no number of positives/tested is given; or if swabs were pooled and the numbers sampled and positive cannot be determined.

In the new data format, these fields became **BdDet** = **diseaseTestedPositiveCount, BdUnc = diseaseTestedQuestionable,** and **Tested** = **individualCount.** The **BdNeg** field was dropped on the new interface, as it can be derived from the other three. A new field, **diseaseTested**, was added for the AmphibiaWeb upload; for *Bd*-Maps data this will always = *Bd*. The **Pooled** [= **measurementRemarks**] field indicates whether swabs were pooled for PCR testing, and sometimes the size of each pool (see **Section IV. Data Field Definitions** for details). A number of optional fields have been little used and were most often left blank: **Morbidity**, **Mortality**, **MortType**, and **Abnorm**, meant to record disease status and symptoms. Morbidity was never used and was dropped in the new format; the other three fields were combined to become **occurrenceRemarks**. **SpecID** [= **catalogNumber**] is an identifier for individual specimens if reported. See **Section IV. Data Field Definitions** for details.

Record the test results as reported, using the above fields as appropriate.

Go to step 19 Record Numbers.

1. Record Numbers. Add record numbers to full, location, and species records. Consult existing data to determine the last-used record number. Populate the **Record Number** [= **materialSampleID**] field beginning with the next unused number. Are the records full records, including both location and species information?
   1. YES – go to step 21 Location Number.
   2. NO – go to step 20 Linking Location and Species Records
2. Linking Location and Species Records. Now that both the location records and their associated species records have record numbers, they must be linked by reciprocal notes in the **Notes** (old format) or **georeferenceRemarks** (new format) fields. The note for the location records will read something like “species sampled may occur at any of the 62 sites represented in RN05882–05998”, whereas the note for the parallel species record will look something like “species record NOT FOR GEOSPATIAL ANALYSIS; sampled sites in RN15137–15141.” Once these sets have been cross-referenced, continue to step 21 Location Numbers.
3. Location Numbers. Each unique combination of locality info and coordinates is assigned an individual **Location Number** [= **locationID**]. This is best done after all data for a source have been entered and formatted, and record numbers have been assigned. First, sort the data by latitude, longitude, and if necessary. **Location** [= **Locality**]. **Location Numbers** are assigned in series based on Continent and Coordinate Accuracy. Series are assigned to full and location records as follows: Africa, 100000s; Asia, 200000s, Australasia, 300000s; Caribbean Islands, 400000s; Europe, 500000s; North America, 600000s; Pacific/Indian Islands, 700000s; South America, 800000s. Country Centroid and Species records are assigned numbers beginning with 9Cxxxx, where C is the lead value of the series for the appropriate continent (e.g., African country centroid records begin with 91xxxx). A set of location records plus species records will thus have location numbers from two series. To assign numbers, check existing data for the last number(s) used in the appropriate series and carry on from there, allocating one location number to each unique set of coordinates. QA/QC: Also search existing data to determine if any new Locality has an existing **Location Number** with matching coordinates; if one is discovered, the old and new records for the site can be tied together by retaining the existing **Location Number** for the new data. Once Location Numbers have been assigned, go to step 22 Taxonomy.
4. Taxonomy. Check and standardize taxonomy (see **Section III.** **Taxonomic Notes**, below). This is another QA/QC step to ensure that taxonomy is consistent with the most recent taxonomic reference, so that tested species are not accidentally double counted. First, check the reported **Genus** [= **genus**] and **Species** [= **specificEpithet**] against taxonomic references (Frost 2020, IUCN 2021, see references under **Section III. Taxonomic Notes**, below), including checking for the correct spelling. Do they match?
   1. YES – retain name as reported. If not already populated and correct, record **Order** and **Family** as given in taxonomic references. Is the full species binomial given?
      1. YES – Go to step 23 IUCN Status
      2. NO – For samples identified only to genus, IUCN status cannot be determined and the **IUCN status** field will be left blank. However, check the taxonomic references to make sure the genus has not been reclassified and enter the revised genus if so, with the original as a **Synonym**. Go to step 24 Site-Level Metrics.
   2. NO – Correct the binomial and/or spelling. Record the binomial as reported in the **Synonyms** field. If not already populated and correct, record **Order** and **Family** as given in taxonomic references. Go to step 23 IUCN Status.
5. IUCN Status. Check conservation status (IUCN 2021) for corrected binomial. Has the species been evaluated as of the latest round of IUCN Red List revisions?
   1. YES – Record 2-letter code for conservation status for each species. DD – Data Deficient; LC – Least Concern; NT – Near Threatened; VU – Vulnerable; EN – Endangered; CR – Critically Endangered; EW – Extinct in the Wild; EX – Extinct. Go to step 24 Site-Level Metrics.
   2. NO – Record status as “NE” for Not Evaluated. Go to step 24 Site-Level Metrics.
6. Site-Level Metrics. Site-level summary metrics are used to distill the record data down to site-level data for use in mapping and geospatial analysis. To calculate site-level *Bd*-detection metrics, first sort the record data by Location Number, *Bd* P/N/U, and species. For each Location Number, tally the species tested and with detections. The results are captured in three fields in the original data, **Site D/ND/U, Spp w/D**, and **Spp Tested** (these are combined into the **Fieldnotes** field separated by pipes in the new data format). **Site D/ND/U** records D if *Bd* was detected for the location in any sampled year or species, ND if not detected, or U if the detection was for some reason uncertain. The other two fields count species with detections at the location, and total species tested at the location. Note that because **Location Numbers** [= **locationID**] can carry across between publications, it is necessary to make sure tallies from all data are combined. In cases where the number of species detected or tested are not recorded, values for these two fields may be <unk> for unknown. For Species records, site-level summary metrics are not calculated, because the ‘site’ the associate with is represented by location records elsewhere; in this case all three fields should read <NA> for Not Applicable. Once site-level metrics have been calculated for the record data, go to step 25 Site-Level Mapping Data.
7. Site-Level Mapping Data. Up to this point in the process, all these steps have been performed on a single data file, the records database, which retains all species and sampling information. However, mapping works better with data reduced to the site level, especially if any geospatial analysis is planned. To derive a new, site-level database, begin with the file sorted as for generating site-level summary metrics, as in step 24 above. Save the file under a new name! Remove all records with duplicate **Location Numbers** [= **locationID**], so that only one record remains to represent each site. Save the file again! At this point, the information on species and individual test results by species, etc., is no longer relevant, so only the fields relating to location information, permissions, and site-level metrics need to be retained. The taxonomic, IUCN status, and individual test results fields can be cautiously deleted, as can the publication-related fields (optional—some locations were reported in only one publication, so this *might* still match up in most cases).

**References**

Olson DH, Aanensen DM, Ronnenberg KL, Powell CI, Walker SF, Bielby J, et al. (2013) Mapping the global emergence of *Batrachochytrium dendrobatidis*, the amphibian chytrid fungus. PLoS ONE 8(2): e56802.

1. **Taxonomic Notes**

Amphibian taxonomy has undergone many and drastic changes since the Olson et al. (2013) PLoS ONE paper was published. In order to make accurate tabulations of species sampled and with *Bd* detected and connect them to conservation status, we standardized (and later updated) taxonomy to the most recent IUCN Red List taxonomy (IUCN 2021), which appears to be based on Frost’s (2020) *Amphibian Species of the World* online resource. Generally, we followed the same process outlined in the Taxonomic Notes (File S1 from Olson et al. 2013), with the updated taxonomic references (see Taxonomy References below). Further taxonomic changes have occurred since that compilation: Craugastoridae now includes the former Strabomantidae and the short-lived Ceuthomantidae; Pelodryadidae and Phyllomedusidae split off from the Hylidae; Rhinodermatiae and Odontophrynidae split from the Cycloramphidae, and more recently the genus *Proceratophrys* moved from that family to Odontophrynidae; and in the Cryptobranchidae we now count the Ozark Hellbender, *Cryptobranchus alleganiensis* ssp. *bishopi* as one with *C. a. alleganiensis* (although the family still has 4 species, having added a new species of *Andrias* in China). In addition, as taxonomic work has progressed apace with new genetics tools, many new species have been identified, adding to the number of species assigned to each family, and others have hopped genera like…well, like frogs.

These changes are reflected in our tally tables S2 and S3, affecting the comparison to those in the earlier publication. Maintaining a consistent taxonomy helps us ensure that we do not double-count species.

Our taxonomy differs somewhat from that maintained by AmphibiaWeb, but only in a few cases, chief among them that our sources recognize the genus *Lithobates* for many North American ranids, whereas AmphibiaWeb retains *Rana* for those species. The database does record synonyms where applicable for these and other species that have been reported under more than one name over time. In the database and our summary tables, species are reported under their current binomial (however ephemeral that may eventually turn out to be). Recent changes in classification are noted in footnotes to Table S2.

**Taxonomy References**

Note: included here are all the references consulted over the life of the project, even if not cited above.

Crother BI (2008) Scientific and standard English names of amphibians and reptiles of North America north of Mexico, with comments regarding confidence in our understanding. Society for the Study of Amphibians and Reptiles Herpetological Circular 37 (2008).

Frost DR (2008) Amphibian species of the world: an online reference. Version 5.2 (15 July 2008). Electronic database accessible at http://research.amnh.org/herpetology/amphibia/index.php American Museum of Natural History, New York, USA.

Frost DR (2009) Amphibian species of the world: an online reference. Version 5.3 (12 February 2009). Electronic database accessible at http://research.amnh.org/herpetology/amphibia/index.php American Museum of Natural History, New York, USA.

Frost DR (2010) Amphibian species of the world: an online reference. Version 5.4 (8 April 2010). Electronic database accessible at http://research.amnh.org/vz/herpetology/amphibia/ American Museum of Natural History, New York, USA.

Frost DR (2011) Amphibian species of the world: an online reference. Version 5.5 (31 January 2011). Electronic database accessible at http://research.amnh.org/vz/herpetology/amphibia/ American Museum of Natural History, New York, USA.

Frost, D.R. (2020) Amphibian Species of the World: An Online Reference. Version 6.1 American Museum of Natural History, New York, USA. doi.org/10.5531/db.vz.0001 https://amphibiansoftheworld.amnh.org/index.php; accessed. November–December 2020.

Frost DR, Grant T, Faivovich J, Bain RH, Haas A et al. (2006) The amphibian tree of life. Bulletin of the American Museum of Natural History 297.

Grant T, Frost DR, Caldwell JP, Gagliardo R, Haddad CFB et al. (2006) Phylogenetic systematics of dartpoison frogs and their relatives (Amphibia: Athesphatanura: Dendrobatidae). Bull Amer Mus Nat Hist 299.

Hedges SB, Duellman WE, Heinicke MP (2008) New World direct-developing frogs (Anura: Terrarana): Molecular phylogeny, classification, biogeography, and conservation. Zootaxa 1737: 1–182.

Heinicke MP, Duellman WE, Hedges SB (2007) Major Caribbean and Central American frog faunas originated by ancient oceanic dispersal. Proceedings of the National Academy of Sciences USA 104: 10092–10097.

IUCN (2010) IUCN Red List of Threatened Species. Version 2010.1. Available at: http//www.iucnredlist.org. Downloaded on 15 April 2010.

IUCN [International Union for the Conservation of Nature]. (2021) IUCN Red List of Threatened Species. Version 2020-3*.* https://www.iucnredlist.org; accessed 1 February 2021.

1. **Environmental Associations with *Bd* Occurrence**

Elevation (m) data were downloaded from the enhanced global elevation model, the Global Multi-resolution Terrain Elevation Data (GMTED2010), developed by the Unites States Geological Survey and the National Intelligence Agency (data description: <https://www.usgs.gov/core-science-systems/eros/coastal-changes-and-impacts/gmted2010?qt-science_support_page_related_con=0#qt-science_support_page_related_con>); data downloaded from: <https://edcintl.cr.usgs.gov/downloads/sciweb1/shared/topo/downloads/GMTED/>). An ESRI-grid (Environmental Systems Research Institute, Redlands, California USA) format was downloaded with the elevation data.

Climate data were derived from the high-resolution gridded datasets provided by the Climate Research Unit (University of East Anglia) and the National Centre for Atmospheric Science (NCAS), United Kingdom (<https://crudata.uea.ac.uk/cru/data/hrg/>) over the 10-year interval 2010 to 2019. These data were at the resolution of 0.5-degree (~55 km) latitude and longitude. Climate data processing entailed 1) downloading zipped ASCII data files; 2) import of ASCII data to a Python array (Python script written for this task: <https://www.python.org/>); 3) generation of necessary statistical grids per climate metric (geotiffs; Python scripts were written for this task; i.e., for mean annual precipitation, low/mean/high average monthly precipitation, temperature range, low/mean/high average monthly minimum temperature, low/mean/high average monthly maximum temperature). All output grids were divided by 10 (10-year time interval of data; ArcGIS software, spatial analyst extension [[www.esri.com](http://www.esri.com)] to convert to appropriate units [precipitation: mm; temperature: degrees C]). For statistical analyses, site-scale *Bd* occurrence and environmental data in an ASCII grid format were read into the R package software *MIAmaxent* (Vollering, Halvorsen & Mazzoni 2019), then consolidated to single *Bd* occurrences (detection, no detection) per 0.5-degree latitude/longitude grid cell, for consistency with climate data.

**References**

Vollering, J, Halvorsen, R, Mazzoni, S. (2019) The MIAmaxent R package: Variable transformation and model selection for species distribution models. Ecol. Evol. 9, 12051– 12068. <https://doi.org/10.1002/ece3.5654>

1. **Data-field Definitions for *Bd*-Maps Data**

**Note:** original field names that are retained as Darwin Core fields in the AmphibiaWeb incarnation of the data will not show an alternate field name.

**Record Number [Darwin Core = materialSampleID]:** Unique numerical tag for database record. Owing to corrections and emendations to the database over time, these numbers are not necessarily continuous, and gaps in numbering may exist.

**Day, Start or End**

**Month, Start or End**

**Year, Start or End:** The date the sample was collected from the wild or captive animal; for museum specimens, the date the specimen was collected in the field. Split into separate columns to avoid confusion between date formats that have month and day in different orders. For records of data collected all on the same day, **Start** and **End** date fields should match. For records including samples collected over a span of time, **Start** and **End** date fields will differ.

# Location-related Fields

**Continent [Darwin Core = continentOcean]:** Continent or greater geographic region. Allowed values given below.

| Continent | Defined as: | Location Number series |
| --- | --- | --- |
| Africa | including Madagascar, Cape Verde, Canary Islands, and Madeira | 100000s |
| Asia | including Russia east of the Urals, the Arabian Peninsula, the Middle East (see exception for Turkey, below), Japan, the Philippines, Indonesia, and Papua New Guinea | 200000s |
| Australasia | comprising Australia and New Zealand | 300000s |
| Europe | including the portion of Russia west of the Urals, plus the Azores; Turkey also overlaps Europe and Asia but is assigned location numbers in the Europe series | 500000s |
| North America | Canada, United States, Mexico, and the countries of Central America south of Mexico, but not including Caribbean island nations | 600000s |
| South America | including the Galapagos Islands | 800000s |
| Caribbean Islands | island nations from Bermuda to Trinidad and Tobago | 400000s |
| Pacific/Indian Islands | island nations of the Pacific and Indian oceans, not including Indonesia, the Philippines, Japan, or Papua New Guinea, which are counted as part of Asia. The Hawaiian Islands are counted as part of the United States, but have location numbers from the Pacific Islands series, 700000s | 700000s |

**Country:** The name for the country in which the sample was taken.

**Region [Darwin Core = stateProvince]:** The name of the state, province, territory, or department (1^st^-level administrative region) in which the sample was taken. Blank if data not available.

**Location Number [Darwin Core = locationID]:** A numeric identifier used to help aggregate records taken at the same location across different sampling dates, species, or studies. Location numbers run in series by **Continent** (see Continent table, above). Country-level centroid and Species records are assigned a location number in the 900000 series, with the second digit designating the continent (e.g., a record for Armenia gets 950002, as the 3^rd^ country centroid for a European nation).

**Location [Darwin Core = locality]:** The name of the place in which this sample was taken. The name should be descriptive and unique to that place. Should be specific enough that data QC could determine if there is a significant error in coordinate entry. If several different coordinates have the same place name in a data set, a numerical indicator or letter may be added to differentiate among sites (“Smith Pond A” or “Great Smoky Mountains site 3”).

**Latitude [Darwin Core = decimalLatitude]:** The latitude of the place at which the sample was taken, in decimal degrees, ranging from 90.000000 (North) to −90.000000 (South). A required field; *Bd*-Maps data do not include records that cannot be assigned location coordinates.

**Longitude [Darwin Core = decimalLongitude]:** The longitude of the place at which the sample was taken, in decimal degrees, ranging from −180.000000 (West) to 180.000000 (East). A required field; *Bd*-Maps data do not include records that cannot be assigned location coordinates.

**Elevation:** The altitude of the place at which the sample was taken, in metres. Must be above the lowest recorded point of earth −422 m (Dead Sea shore, Jordan) and below the highest recorded pace on Earth, 8850 m (summit of Mount Everest). Highest and lowest points in Europe are 5642 m (Mount Elbrus, Russia) and -28 m (Caspian Sea shore, Russia).

In some cases, elevation was uploaded with data. In others, coordinates uploaded without an elevation, and elevation was calculated within the *Bd*-Maps database and appeared in the download. For update data that were never held in the *Bd*-Maps database proper, elevation may be blank if it was not available from the source of the coordinates. Given this variety of sources, **it may be best for the purposes of geographic modeling to re-derive elevations from a consistent digital elevation model based on coordinates rather than relying on elevations listed in the database**, though they could serve for roughly classifying data.

**Coordinate Source [Darwin Core =** part of **georeferenceProtocol]:** The original format of coordinates from the data source, before they were converted into decimal degrees latitude and longitude.

**DDLL:** decimal degrees latitude and longitude (unconverted)

**DMS:** Converted from degrees, minutes, and seconds

**UTM:** Converted Universal Transverse Mercator coordinates as Easting and Northing, with zone and datum.

**TRS:** Converted from U.S. Public Land Survey, Township, Range, Section coordinates.

**GEOCD: The following four categories have been subsumed under one category, designating coordinates geocoded based on various sources and references:**

LNGM: Derived from a location name using reverse geocoding.

LNTZ: Derived from a location name using TopoZone.

MAP: Derived from a location name manually using a map published with the data, sometimes finding coordinate data from Google Earth.

Coordinates were geocoded based on maps from the relevant publication, location names or codes, and references such as Google Earth, Google Maps, Topo Zone, Mapcarta, MapQuest, topographic maps including national topographic map servers, gazetteers, and online sources such as other published research at the same site, and websites for national, state, and regional parks, wildlife, and conservation areas.

**Coordinate Accuracy [Darwin Core = georeferenceRemarks]:** A categorical assessment of how accurate the Latitude and Longitude of the sample are.

**Exact:** The position is taken from the exact point of the amphibian sampling site, though not necessarily of the individual animal sampled.

**Approximate:** The latitude and longitude represent a position close to where the samples were collected, but not the exact site. Generally, error should be less than 2 kilometers.

**Vague:** The latitude and longitude denote the general vicinity of the sample, but are estimated and may be up to several kilometers off. This could include centroids of a county or municipality.

**Region:** The latitude and longitude represent the first-level administrative region where the sample was taken, or can be traced only to an area larger than could be classified as “Vague”. These records should not be used for geographic modeling.

**Country Centroid:** The latitude and longitude represent the country where the sample was taken. These records primarily help to locate species that otherwise have no location data, and should not be used for geographic modeling.

**Fuzzy:** The latitude and longitude have been deliberately changed (usually just made much less precise) to mask a sensitive location.

**Species:** Record is present to document testing of a particular species that can’t be tied to a specific geographic coordinate—location records may exist for the same study that did not break down results by species and show <unknown> or <multiple> in taxonomic fields. See **Notes** field for these records to find the location records that correspond to them. Records designated as “Species” in this field should not be used for geographic modeling.

**Geographic modeling should probably include only data that fall within the precision of the modeling—most often only Exact and Approximate points, but perhaps also Vague points, likely not Region, never Country Centroid or Species records.**

**Coordinate Error [Darwin Core = coordinateUncertaintyInMeters]:** Estimated coordinate spatial error in meters.

Data sources did not provide this information. At the time data were collated and uploaded, a categorical value designated Coordinate Accuracy was assigned, based on precision of provided coordinates, study design and sampling method, and accuracy of location name or published maps. Coordinate Error values in meters were added in 2020, usually many years after records were created, based on these coordinate accuracy classes, and may not reflect estimates made at the time of either collecting GPS or calculating map coordinates, or while geocoding based on maps, location names, and references such as Google Earth, Topo Zone, topographic maps, USGS Geographic Names Information System, gazetteers, and additional online sources.

Exact – if coordinates to only 2 decimal places assigned 800 m error; 3 decimal places 110 m; or based on some other indication of precision in place name

Approximate – coordinates given to only degrees and minutes in DMS are assigned 1100 m error

Vague – assigned based on any indication of precision in place name

Fuzzy – assigned 10,000-m error, as coordinates are deliberately made less accurate to protect sensitive sites.

# Taxonomic Fields

**Order:** Taxonomic order of species sampled. Allowed values are Anura (frogs and toads), Caudata (salamanders), and Gymniophiona (caecilians).

**Family:** Taxonomic family of species sampled, as of 2018, using the IUCN Red List (and Amphibian Species of the World) as references.

**Genus:** Taxonomic genus of species sampled as of 2018, using the IUCN Red List (and Amphibian Species of the World) as references.

**Species [Darwin Core = specificEpithet]:** Taxonomic species (and in some cases, subspecies) of animals sampled as of 2018, using the IUCN Red List (and Amphibian Species of the World) as references. If sampled animal(s) could not be identified to species, may be designated as “sp.” (for animals of only one species), “spp.” (for animals of the same genus, but potentially different species), or with a designator such as “sp. 1” if more than one different unidentified species sampled came from the same genus.

If species was not given for samples, some or all of the above four fields may read <unknown>; if more than one species is represented in a record, some or all of these fields may read <multiple>.

**Cf/Aff/Kl [not included in AmphibiaWeb version]:** indicator of a species that was difficult to identify for some reason (cf.), perhaps due to life stage, color morph, or indeterminate characteristics, or that could be identified as related to, but not the identical with, a similar species (aff. = affinis). Value of 1 for cf used in Latin binomial, e.g., “*Leptodactylodon* cf. *boulengeri*”. Value of 2 for aff. used in Latin binomial, e.g., “*Ptychadena* aff. *perreti*”.

Dropped from data on AmphibiaWeb

**Synonyms [not included in AmphibiaWeb version]:** Alternate binomials under which users might search for a particular species. Because it’s lined up with IUCN Red List taxonomy, *Bd*-Maps taxonomy differs slightly from AmphibiaWeb’s established taxonomy. If the AmphibiaWeb binomial for a species doesn’t agree with the IUCN taxonomy, the synonym AmphibiaWeb name will be given in this field. In addition, because amphibian taxonomy is always in flux, recently superseded names for species are included here**. Note: In the new data portal, this function is automatically taken care of in the program itself, so this field is absent.**

**Status [not included in AmphibiaWeb data]:** Conservation status of the species from the IUCN Red List, as of October 2018 or July 2020. For records of sampled animals identified only to genus (e.g., *Amnirana* sp.), this field will be blank. Definitions:

NE – Not Evaluated

DD – Data Deficient

LC – Least Concern

NT – Near Threatened

VU – Vulnerable

EN – Endangered

CR – Critically Endangered

EW – Extinct in the Wild

EX – Extinct

**Because status changes over time and needs to be updated, this will become part of the automatic updates of taxonomy and status on the AmphbiaWeb portal, so this field will no longer be included.**

**NatInt** **[Darwin Core = establishmentMeans]:** Native or Introduced. “Native” if the species sampled was native to the country or region in which it was found; “Introduced” if it was not.

**WildCapt [Darwin Core = originalSource]:** Was the sample taken from animals found or collected in the wild, or from captive animals?

**Wild:** The animals were sampled or collected in the wild, or in an outdoor setting where they could have easy contact with wild animals (such as outdoor holding facilities on farms).

**Captive:** The animals tested were indoor captives with no easy contact with wild animals, but which could potentially have contact with other animals of the same or different species in a captive setting. This includes animals from zoos, pet shops, and markets.

**Geographic modeling should not include records of captive animals.**

**MusSpec [Darwin Core = basisOfRecord]:** Were the sampled animals preserved specimens from a museum or research collection? “Yes” if they were; “No” or blank if they were not. Darwin Core: “Yes” = PreservedSpecimen; “No” or blank = LivingSpecimen

# *Bd* Sampling Fields

**diseaseTested:** added field for Amphibia Web upload; controlled vocabulary; for *Bd*-Maps data, will always = *Bd*

**Test P/N/U [Darwin Core = diseaseDetected]:** Results of *Bd* testing. P if *Bd* was detected in some or all of sampled animals; N if *Bd* was not detected in any of the sampled animals represented by this record; U if test result was equivocal or there is reason to doubt the result. This variable was added to make it easier to sort and identify positive records. [Darwin Core: P = True; N = False; U = NoConfidence]

*Note: the following numeric fields may be blank in three cases:*

1. *where* *results were not reported by species for a set of sample sites in a published map or table;*
2. *where* *a species is reported as positive or negative at a site but no number of positives/tested is given; or*
3. *where* *swabs were pooled and the numbers sampled and positive cannot be determined.*

**BdDet [New AW field = diseaseTestedPositiveCount]:** Number of amphibians in the sample that **tested positive** for *Bd*. An integer greater than or equal to 0 and less than or equal to the number tested. Should be at least 1 for a positive detection record.

**BdUnc [New AW field = diseaseTestedResultQuestionable]:** Number of amphibians for which results were uncertain, either because of very low zoospore count or for other reasons (Madagascar debate). An integer greater than or equal to 0 and less than or equal to the number tested. Many cells will be blank owing to no data.

**BdNeg:** Number of amphibians in the sample that tested negative for *Bd*. An integer greater than or equal to 0 and less than or equal to the number tested.

*This field is omitted in the Amphibia Web platform format, as it can be derived from the numbers tested and positive or questionable.*

**Tested [Darwin Core = individualCount]:** Number of amphibians that were tested for the sample in this record. If there is only one animal in this record, this field should be 1. An integer greater than zero; should be at least 1, unless there are no data regarding the number tested.

**Pooled [Darwin Core = measurementRemarks]:** Indicates whether swabs were pooled for PCR testing, and in some cases the size of each pool. Values can include “No”, “Yes”, or numbers pooled. In pooled testing, the **Det** field may have a value of 1 and the **Neg** field may be blank if the pooled sample tested positive, as there is no way to determine in exactly how many of the individual animals represented in the pool *Bd* would have been detected if they had been tested separately. If **Pooled** is left blank, the default value is “No”.

In AmphibiaWeb data, values will be “NotPooled” or “Pooled” followed by description of pooling system used.

**Optional fields that may be left blank:**

**Morbidity:** How many amphibians in the sample showed disease symptoms. An integer greater than or equal to 0 and less than or equal to the number tested.

Because this field was never used, it was omitted in AmphibiaWeb data.

**Mortality:** How many amphibians in the sample were dead. An integer greater than or equal to 0 and less than or equal to the number tested.

**MortType:** Type of Mortality. Allowed values: Zero, Single/Few, Mass mortality, Extirpation.

**Abnorm:** Abnormalities. Was there anything abnormal about the animals in this sample, e.g., missing/deformed body parts? This is a free-text field.

**Life Stage [Darwin Core = lifeStage]:** The life stage of the amphibian(s). Allowed values: Larva, Metamorph, Juvenile, Adult, Neotene, Eggs. If life stage was an important variable being sampled, it may be best to break down results by entering a separate record and detections/no. tested for each life stage.

**Sex:** The sex of the biological individual(s) represented in the Occurrence. Allowed values Male or Female.

# Sample Information Fields

**SpecID [Darwin Core = catalogNumber]:** The Id of the swab taken from the amphibian, for records that reflect only a single animal sampled. Any alphanumeric string. Often left blank; will usually be blank for records including multiple sampled animals.

**Method [Darwin Core = testMethod]:** Technique used to test for the presence/absence of *Bd* in this/these amphibians. Allowed values: Histology, PCR, qPCR, rtPCR, Isolation, Visual, or combinations of these methods.

**Laboratory [Darwin Core = diagnosticLab]:** The name of the lab where the *Bd* testing was carried out. Often left blank.

# Record Information Fields

**Permission [not included in AW data]:** Who has permission to see and download this record?

**Public:** visible to all users

**Private:** Visible only to the person in the Contact field

**RACE:** Visible to any member of the RACE group

AmphibiaWeb’s policy is to make all data publicly available; however, some of the *Bd*-Maps data were assembled under a different set of agreements that allowed data to remain private, and we must continue to respect the wishes of the data owners.

Private data will be maintained in a separate, private project until such time as permission has been given.

**Data Source Type:** origin of data—Journal Publication, Thesis or Dissertation, Report (gray literature), Email communication (sent to us directly, usually unpublished data), Fieldwork (input by data owner), Map, Newspaper.

In the AmphibiaWeb data, information from this field is incorporated into **associatedReferences** for records that have no published source.

**Citation:** The short reference to the publication wherein these data are published, e.g., Smith et al. 2008. Full references can be found in the accompanying *Bd*-Maps data bibliography, which also contains the URL or DOI of the publication, if available.

In AmphibiaWeb project, concatenated into **associatedReferences** with the full reference.

**Contact [Darwin Core = principalInvestigator]:** The name of the person who 'owns' these data. Usually includes the email address given in the accompanying publication.

**Reference [Darwin Core = associatedReferences]:** The citation concatenated with the full reference to the publication from which the data originated.

Where citation, contact, and reference are absent, data may have been directly input by the originator without this information.

# Site-Level Summary Fields

**Site D/ND/U:** Site detected/not detected. A summary value for all records from the same site (same set of geographic coordinates, as denoted by **Location Number**), across species and sampling dates.

If one or more records from the aggregate for a location number were positive (P in the P/N field), the site is designated D for Detected; if none of the aggregate records for a location number were positive, the site is designated ND for Not Detected. If all of the possibly positive results were uncertain, the site is designated U. An individual record that was negative or questionable (N or U in P/N/U field) may still be D in Site D/ND/U if *Bd* was detected in other species at that site on the same sampling occasion, or in any species on other sampling occasions.

**This variable is most useful for geographic modeling, when it is preferable to aggregate all records for a site to avoid autocorrelation.**

**Spp w/D:** Number of species in which *Bd* was detected at a site (defined as having the same coordinates and location number). Zero or positive integer, unless some records for site have <unknown> or <multiple> species designators, in which case the value should be <unk> for unknown.

**Spp Tested:** Total number of species with samples tested from a site. Zero or positive integer, unless some records for site have <unknown> or <multiple> species designators, in which case the value should be <unk> for unknown.

In the AmphibiaWeb data, these three fields are folded into the **Fieldnotes** field.
