# Supplementary material for: Global Patterns of the Fungal Pathogen Batrachochytrium dendrobatidis Support Conservation Urgency
Source: Front Vet Sci. 2021 Jul 16;8:685877. doi: 10.3389/fvets.2021.685877 (PMC8322974; doi:10.3389/fvets.2021.685877)
Supplement: Supplementary Appendix 1 — Detailed methods. [file Data_Sheet_1.zip › Data Sheet 1/Appendix S2.docx]

**Frontiers in Veterinary Science, section Zoological Medicine**

**Special Issue: Emerging Infections and Diseases of Herpetofauna**

**Hosted by Amanda L.J. Duffus; Rachel E. Marschang**

**Global Patterns of Fungal Pathogen *Batrachochytrium dendrobatidis* Support Conservation Urgency**

Deanna H. Olson^1^*, Kathryn L. Ronnenberg^1^, Caroline K. Glidden^2^, Kelly R. Christiansen^1^, Andrew R. Blaustein^3^

^1^U.S. Department of Agriculture, Forest Service, Pacific Northwest Research Station, Corvallis, Oregon, USA

^2^Stanford University, Department of Biology, Stanford, California, USA

^3^Oregon State University, Department of Integrative Biology, Corvallis, Oregon, USA

**Appendix S2: Comparing the current study with results from Castro Monzon et al. 2020**

**Country Comparison of *Bd* Occurrences**

Including countries with only country-level *Bd* locations, Castro Monzon et al. (2020: hereafter CM) reported 86 *Bd*-positive of 119 tested countries; we reported 124 tested countries, with 88 *Bd* detections. We reported *Bd* sampling in 13 countries that they did not include in their report: Bosnia and Herzegovina, Botswana, Brunei, Central African Republic, Dominican Republic, Gambia, Hong Kong, Ireland, Kazakhstan, Luxembourg, Mongolia, Pakistan, Poland, and Taiwan. In contrast, Castro Monson et al. (2020) reported *Bd* sampling in 9 countries that we did not include in our compilation: Angola, Burundi (*Bd* detection), Congo, Equatorial Guinea (*Bd* detection), Eritrea, Guinea-Bissau, Israel (*Bd* detection), Sudan, and Zimbabwe. In their online data file, Castro Monzon et al (2020) had recorded data from Brunei as being from Indonesia, and apparently did not count Brunei as a separate country as we have done herein. In addition, Bd status reports differed for two countries, Madagascar (we designated as *Bd*-uncertain, they counted as *Bd*-detected; to reconcile our different designations, Madagascar is provisionally included as *Bd*-detected [from a wild sample] in our grand total) and Thailand (they counted as *Bd* not detected, but we had compiled *Bd*-detections from a different source; Thailand is included as *Bd*-detected below).

Below are the sources that resulted in differences in species/positive counts when comparing our Bd dataset with Castro Monzon et al. (2020) ; they do not represent all sources we or they consulted. Cap = captive *Bd* sample. Wild = field Bd sample. Pos = *Bd*-positive (detection). Neg = *Bd*-negative (no detection).

**We had site-level data from the following 12 countries (4 with detections), CM did not:**

Note: (**pos [in a wild sample]**) or (neg) state whether we counted the country as *Bd*-detected or not detected.

Bosnia and Herzegovina (neg): Sunje et al. 2018 (unique source)

**Botswana** (**pos**): Weldon 2005 (thesis) (**pos**); Vredenburg et al. 2013 (neg)

Brunei (neg): Kaiser and Grafe 2011; CM listed what appear to be the same data as being from Indonesia

**Dominican Republic** (**pos**): Joglar et al. 2007 (unique source)

Hong Kong (neg): Kolby et al. 2014 (pos cap), Rowley et al. 2007b cap and wild neg; CM did not cite either of these sources. Note: we do not count a country as *Bd* detected based only on a positive captive sample.

Ireland (neg): Gandola and Hendry 2013 (unique source)

Kazakhstan (neg): Swei et al. 2011— although CM cited this paper, the data for Kazakhstan are not present in their data file

**Luxembourg** (**pos**): Wood et al. 2009 (unique source)

Mongolia (neg): Swei et al. 2011—although CM cited this paper, the data for Mongolia are not present in their data file

Pakistan (neg): Swei et al. 2011—although CM cited this paper, the data for Pakistan are not present in their data file; also, unpublished data from the Bagrot Valley uploaded without contact information

**Poland** (**pos**): Czeczuga et al. 2011 (**pos**), Kolenda et al. 2017 (**pos**) (unique sources)

Taiwan (neg): Lehtinen et al. 2008 (unique source)

Note: In our data, Eswatini appears as Swaziland.

**Country-level *Bd*-negatives in our data**:

We had, CM did not:

Central African Republic (country-level neg): Marc-Oliver Rödel, unpub. data, 3 spp. sampled

Gambia (country-level neg): Marc-Oliver Rödel, unpub. data, 2 spp. sampled

Both we and CM had:

Armenia (country-level neg): Ouellet et al. 2005, 2 spp. sampled

Barbados (country-level neg): Ouellet et al. 2005, 1 spp. sampled

Iran (country-level neg): Ouellet et al. 2005, 1 sp. sampled

Latvia (country-level neg): Ouellet et al. 2005, 2 spp. sampled

Of these 6 countries, CM had Armenia, Barbados, Iran, and Latvia, from Ouellet et al. 2005, but not Central African Republic and Gambia.

**CM had data for these countries, we did not (*note 3 of 11 countries below had *Bd*-detection that we did not have in our data compilation):**

Angola (neg): from Zhu et al. 2014 (Africa—different ref from the other Zhu et al. 2014 China we both used)

**Burundi** (**pos**): from Byrne et al. 2019

Congo (neg): Zhu et al. 2014

**Equatorial Guinea** (**pos**): Byrne et al. 2019, Zhu et al. 2014 Africa

Eritrea (neg): Zhu et al. 2014 Africa

Guinea-Bissau (neg): Zhu et al. 2014 Africa

**Israel** (**pos**): Perl et al. 2017

Sudan (neg): Zhu et al. 2014 Africa

Zimbabwe (neg): Zhu et al. 2014 Africa

All of these countries’ data came from sources that were unique to Castro Monzon et al. 2020, which we did not consult.

***Bd* status differed:**

Madagascar (us neg, CM **pos**): CM counted uncertain positives as detections, but we did not. In addition, they consulted a newer source that we did not include, which had additional positive samples (Byrne et al. 2019). In our grand total tally by countries, we reconciled these differences by considering *Bd* as detected in the wild in Madagascar, for consistency with the CM report.

Thailand (us **pos**, CM neg): we counted wild and captive positives from Techangamsuwan et al. 2017, which they did not cite.

**Other differences:**

CM counted points that appeared to be in South Korea as being in North Korea, but ironically may also have counted the one point in North Korea (Fong et al. 2015) as being in South Korea.

**Species Comparison**

**One major difference in species reported is due to the fact that we reported captive samples, but they did not.** This also accounts for a number of the peer-reviewed references that we included in our study, but CM did not.

**Handling of samples identified only to genus**

There is no way to tell how our two methods compare for counting the species identified only to genus. We counted a Genus sp. if it was the only member of that genus sampled anywhere, or if it was the only positive sample for a country or the only sample of the genus for a country some distance from the nearest other member of the genus sampled. If other named members of the genus had been sampled for the country, we did not count it. We counted all Genus sp. nov.

Likewise, we cannot distinguish whether both studies handled hybrid species the same way. We did not count a hybrid if both halves of the cross were already counted, unless it was a new *Bd*-positive for one of the species.

**Disagreements with original sources**

In a few cases, for sources used by both papers, our interpretation of the results reported differed. In some cases, the source itself held contradictions, with a table in the main text giving one result, but the accompanying data apparently yielding a different answer.

One example:

Seven species from Holmes et al. 2012 were counted by CM as sampled/negative, but according to the table in the original source, species that theoretically could have been present in the amphibian assemblage for the sampled area were listed, with 0/0 values if the species wasn’t found. This accounts for *Eleutherodactylus grabhami, E. jamaicensis, E. junoni, E. orcutti, E. sisyphodemus, Osteopilus crucialis, O. marianae*. However, this didn’t affect the country count.

**Source Comparison**

The chief differences between the two studies were twofold: we cut off our data compilation at the end of 2019, whereas Castro Monzon et al. carried on into 2020; and our database contained directly contributed, sometimes unpublished data, plus results from non-peer-reviewed publications such as agency reports, theses and dissertations, and other sources. Most of the differences in species and country counts are directly traceable to these differences in source materials. References in **bold face** made a difference to the species or country tallies (country is specified).

**Refs we used that they didn’t use**

*Reports/gray literature*

Alminas et al. 2010; Clemann et al. 2009; Cunningham and Minting 2008; Gaertner et al. 2007 chytrid meeting abstracts; Howard et al. 2010, 2012; Hunter et al. 2009; Mitchell and Green 2002; **Moyer and Weldon 2006**; Obendorf 2005; Pauza and Dreissen 2008; Rogers and Banulis 2004; Rosen and Schwalbe 2002; **Sredl et al. 2002**; **Steens et al. 2012**; **Stevens et al. 2012; Weldon and du Preez 2004**

*Theses/dissertations*

Arai 2008; Bakland 2018; **Bartkus 2009**; Blackburn 2001; Blackley 2016; Brocco 2017; Charbonneau 2006; DiLeo 2010; Firkins 2015; **Ghirardi 2011; Nicolas 2007**; **Schrenker 2017**; **Weldon 2005 (spp. + Botswana)**

*Peer-reviewed*

**Acevedo et al. 2016**; **Adams MJ et al.** 2007, **2008**; Addis et al. 2015; Agostini et al. 2015; Alemu et al. 2013; Allain and Goodman 2017; **Amorim et al. 2019**; Augustine and Neff 2016; **Baláž et al. 2013**; Barrasso et al. 2009; **Barrionuevo and Ponssa 2008**; Bauer et al. 2018; Becker and Harris 2010; Bell et al. 2004; **Berger et al. 1999**; Bettaso and Rachowicz 2006; Blackburn et al. 2010; Brannelly et al. 2012, 2016, 2018 Australia; Briggler et al. 2008; Briggs and Burgin 2004; Brodman and Briggler 2008; Brown et al. 2019; Burkart et al. 2017; Burrowes et al. 2020; Canessa et al. 2013; Carey and Livo 2009; Carnaval et al. 2005; Carvalho et al. 2017; Catenazzi et al. 2010; Chajma and Vojar 2016; **Chinnadurai et al. 2009**; Churgin et al. 2013; Civiš et al. 2013; **Clemann et al. 2009**; **Coutinho et al. 2015**; Cunningham et al. 2005; **Czezuga et al. 2011 (Poland)**; De Paula et al. 2012; Deguise and Richardson 2009; Di Rosa et al. 2007; Duncan Pullen et al. 2010; Ecoclub Amphibian Group et al. 2016; Enciso et al. 2008; Federici et al. 2008; Felger et al. 2007; **Fenolio et al. 2013 (Rept Amph)**; Gabor et al. 2017; Gaertner et al. 2009a (EcoHealth), 2009b (Herp Rev), 2010; Gal et al. 2012; **Galindo-Bustos et al. 2014 (captive)**; **Gandola and Hendry 2013 (Ireland)**; Garcia et al. 2007, 2009; **Garner et al. 2005**, 2006; Ghirardi et al. 2011; **Gilbert et al. 2012**; Gillespie et al. 2015; Glenney et al. 2010; Goodman and Ararso 2012; Govindarajulu et al. 2006; (Campbell) Grant et al. 2008; **Grasselli et al. 2019**; Green and Dodd 2007; Green and Muths 2005; **Goka et al. 2009 (captives)**; Hagman and Alford 2015; **Hale et al. 2005**; Harner et al. 2013; Hasken et al. 2009; **Hauselberger and Alford 2012**; **Havlíková et al. 2015**; Hayes et al. 2009; **Hertz et al. 2018**; Hunter et al. 2010; Hyman and Collins 2012; Isidoro-Ayza et al. 2019; **Joglar et al. 2007** (**Dominican Republic pos)**; Julian et al. 2019; Karvemo et al. 2019; Keitzer et al. 2009; Kilburn et al. 2010; **Kik et al. 2012**; Klop-Toker et al. 2016; Köhler et al. 2016; **Kolby 2014 (captive)**; Kolby and Padgett-Flohr 2009; **Kolby et al.** **2014** **(Hong Kong cap pos)**, 2015a (Honduras), **2015b (Madagascar); Kolenda et al. 2017 (Poland); Lambertini et al. 2017**; Lampo and Señaris 2006; Lastra Gonzalez et al. 2019; Lauer et al. 2007; Laufer et al. 2018**; Lehtinen et al. 2008 (Taiwan)**; Letoof et al. 2013; Longo and Zamudio 2017; **Lötters et al. 2005, 2012**, 2018; Lovich et al. 2008; Lowe 2009; **Makange et al. 2014**; **Malhotra et al. 2007;** Marhanka et al. 2017; Marquez et al. 2010; Marshall et al. 2019; **Mathie et al. 2018 (Guyana)**; May et al. 2011; McMillan et al. 2019; **Mendelson et al. 2005 (2004)**; **Michaels et al. 2018** **(captives)**; Montanucci 2009; Morehouse et al. 2003; Morell 1999; Morgan et al. 2007; Mosher et al. 2018; **Murphy et al. 2015**; **Murray et al. 2010**; Mutnale et al. 2018; Mutschmann et al. 2000; Obendorf and Dalton 2006; **Obon et al. 2013**; Olori et al. 2018; Padgett-Flohr and Longcore 2005, 2007; Parker et al. 2002; **Pasmans et al. 2004**; Patel et al. 2012; Pearl and Green 2005; Pessier et al. 1999; Polasik et al. 2016; Puschendorf 2003; **Puschendorf et al.** 2009, **2013**; Rabemananjara et al. 2011; Raffel et al. 2010; **Rasmussen et al. 2012**; **Reeder et al. 2011**; Reeves 2008; Reeves and Green 2006; Regester et al. 2016; Rittmann et al. 2003; Rizkalla 2009; **Rodriguez et al. 2014** (Brazil—CM did not include many of the species from this paper); **Ron and Merino-Viteri 2000**; Roth et al. 2013; Rovito et al. 2009; **Rowley et al. 2007b (Hong Kong neg)**, 2013; Ruano-Fajardo et al. 2016; **Ruggeri et al. 2018 DAO, 2018 Hydrobiologia**; **Russell et al. 2019**; **Sabino-Pinto et al. 2017**; **Schloegel et al.** 2009, 2010, **2012**; Shaw et al. 2013; Shin et al. 2014; Simoncelli et al. 2005; Slough 2009; **Soorae et al. 2012**; Soto-Azat et al. 2009; **Spitzen-van der Sluijs et al. 2011 (captives)**; Stagni et al. 2002, 2004; **Šunje et al. 2018 (Bosnia and Herzegovina)**; **Sura et al. 2010 (Poland)**; Sztatecsny and Hold 2009; Talbott et al. 2018; **Tamukai et al. 2014 (captives); Tarvin et al. 2014; Techangamsuwan et al. 2017 (Thailand; most but not all captives)**; Thien et al. 2013; **Timpe et al. 2008**; Tobler and Schmidt 2010; Todd-Thompson et al. 2009; **Toledo et al. 2006a S Amer J Herp,** 2006b Amph Rept Cons; Tominaga et al. 2013; **Une et al. 2008 (captives)**; Urbina et al. 2018; **Vasquez-Ochoa et al. 2012**; **Velasquez-E et al. 2008; Velo-Anton et al. 2012; Venegas et al. 2008**; Venesky and Brem 2008; Voordouw et al. 2010; Vörös et al. 2011, 2012; Vredenburg et al. 2012; Waldman et al. 2001; Wei et al. 2010; **Weinstein 2009**; Weldon and Du Preez 2004; **Weldon et al. 2008**, 2014; Wimsatt et al. 2014; Wixson and Rogers 2009; **Wombwell et al. 2016**; **Wood et al. 2009 (Luxembourg)**; Young et al. 2007; Zancolli et al. 2013; Zevallos et al. 2016; Zhu et al. 2016; Zimkus and Larson 2013; Zippel and Tabaka 2008

*Unpublished data*

multiple sources; CM didn’t use any unpublished data; listed are only sources that made a difference to country tallies

Mark-Oliver Rödel, unpub.—Central African Republic, CLO, 3 spp.; Gambia, CLO, 2 spp.)

**Refs they used that we didn’t use**

(these refs do not appear in the reference list following Supplemental Table S2)

Ayres et al. 2020; Backlin et al. 2015; Bates et al. 2018; **Becker et al. 2016, 2019**; **Benacio et al. 2019**; Berger et al. 2004; Berger et al. 2005; Bishop et al. 2009; Bletz et al. 2015b, 2017; **Bosch et al. 2017 (Cuba)**, 2018 (Spain); **Brannelly et al. 2018 Peru** (we had two other Brannelly et al. 2018s, but not this one); Bull 2006; Burrowes et al. 2004, 2011, 2017; **Byrne et al. 2019 (Burundi, Equatorial Guinea)**; Carey et al. 2009; Cashins et al. 2015; Clare et al. 2016; Cook et al. 2018; Crespi et al. 2015; **Cusi et al. 2017**; **de Oliveira Ramalho et al. 2013**; **DiRenzo et al. 2017, 2018; Erdmann et al. 2018**; **Familiar López et al. 2017**; **Fenolio et al. 2011**; **Forti et al. 2017**; Garcia et al. 2019; Gómez et al. 2015; Goodman et al. 2012 (unless this is really Goodman and Ararso 2012); Graham et al. 2013; **Greenhawk et al. 2017**; Grogan et al. 2018; **Guayasamin et al. 2014**; Hammond et al. 2020; Hernández-Gómez et al. 2020; Hite et al. 2016; Hughey et al. 2019; Hyne et al. 2009; **Jacinto-Maldonado et al. 2020**; Jaeger et al. 2017 (Peru); Jani et al. 2017; Jiménez et al. 2019; Joseph and Knapp 2018; Knapp et al. 2011; Kriger et al. 2006a, 2006b; **Krynak et al. 2018**; **Laking et al. 2017;** Lambert et al. 2016; Langhammer et al. 2014; Lennon et al. 2014; Lisboa et al. 2013; Longo et al. 2015, 2017 (unless this is Longo and Zamudio 2017); Lovich et al. 2010; McMillan et al. 2020; Medina et al. 2019; Moreno et al. 2010; **Nava-González et al. 2019**; Nieto et al. 2007; Padgett-Flohr and Goble 2007; Perez et al. 2014; **Perl et al. 2017 (Israel)**; Pope et al. 2016; **Preuss et al. 2016**; Rachowicz and Briggs 2007; **Rebollar et al. 2016; Ribiero et al. 2020**; Richter et al. 2013; **Rodriguez and Catenazzi 2017**; Rollins-Smith et al. 2015; Sainsbury et al. 2016; **Salla et al. 2018**; Savage and Zamudio 2016; Savage et al. 2015; Scheele et al. 2016 (Central America); Skerratt et al. 2011; Spitzen-van der Sluijs et al. 2017; **Thorpe et al. 2018**; Voyles et al. 2012; Vredenburg and Summers 2001; Walke et al. 2017; **Wang et al. 2017, 2018**; Weldon et al. 2004 (unless this is Weldon and Du Preez); Woodhams et al. 2012; **Yap et al. 2016** (excluded from our data to avoid duplication with their data on AmphibianDisease.org); **Zhu et al. 2014 pipids (Angola, Congo, Equatorial Guinea, Eritrea, Guinea-Bissau, Sudan, Zimbabwe)** (both had Zhu et al. 2014 China)
