# Supplementary material for: Global Patterns of the Fungal Pathogen Batrachochytrium dendrobatidis Support Conservation Urgency
Source: Front Vet Sci. 2021 Jul 16;8:685877. doi: 10.3389/fvets.2021.685877 (PMC8322974; doi:10.3389/fvets.2021.685877)
Supplement: Supplementary Appendix 1 — Detailed methods. [file Data_Sheet_1.zip › Data Sheet 1/Table S1.docx]

**Table S1.** Summary of *Batrachochytrium dendrobatidis* (*Bd*) detection (+) and no-detection (-) records and sites in the Global Bd Mapping Project (*Bd*-Maps) database through December 2019. Record^1^ = *Bd* sampling of a species on a date at a site for a project. Site^2^ = Unique latitude and longitude coordinate. Records with Country Centroid or Region coordinate accuracy lacked precise location information. Species-only records track the species that were sampled in an associated group of location records. Wild = *Bd* sample taken from wild-caught amphibian (in field for from museum specimen). Captive = *Bd* sample taken from captive amphibian. U^3^ = *Bd* sampling result uncertain.

| **Record and Site Type** | **Wild** | | | |  | **Captive** | | | | **Grand Total** |
| --- | --- | --- | --- | --- | --- | --- | --- | --- | --- | --- |
|  | ***Bd*+** | ***Bd*-** | **U** | **Total** |  | ***Bd*+** | ***Bd*-** | **U** | **Total** |  |
| Records: |  |  |  |  |  |  |  |  |  |  |
| Full records (location, species, dates(s), plus location records (species not stated) | 9,144 | 22,150 | 35 | 31,329 |  | 288 | 1,260 | 3 | 1,551 | 32,880 |
| Country Centroid | 5 | 109 | 0 | 114 |  | 47 | 137 | 0 | 184 | 298 |
| Species records | 190 | 350 | 0 | 540 |  | 15 | 20 | 0 | 35 | 575 |
|  |  |  |  |  |  |  |  |  |  | **33,753** |
| Sites: |  |  |  |  |  |  |  |  |  |  |
| Full site location | 5,552 | 8,861 | 21 | 14,434 |  | 138 | 74 | 1 | 213 | **14,647** |
| Centroid or Region | 8 | 47 | 0 | 55 |  | 7 | 8 | 0 | 12 | 67 |

^1^Principal investigators varied in how they reported *Bd* sampling data. We designated record types to cover various contingencies in the Global Bd Mapping Project (*Bd*-Maps) database. A *full record* contained *Bd* sampling results for a species at a particular location on a particular sampling date or range of dates. *Species records* documented *Bd* test results for species that may have been sampled at any of a set of location records; they had generalized, less-precise geographic coordinates that were not meant to be mapped. *Location records* were *Bd* test results that included site coordinates but may either have given no information about species tested, or may have been tied, along with other locations tested in a particular survey, to a set of species records or samples for pooled analyses. If location was known only to the level of the country sampled (Country Centroid coordinate accuracy) or a major region (Region), it was a subcategory of a species record. Species records and country centroid records were designated with a different series of location numbers in the database beginning with 9xxxxx, to make it easier to sort them out of the data. Full records and location records were included in data reduced to the site-level for *Bd* occurrence mapping and geospatial analysis; species records and any full records with ‘Country Centroid’ or ‘Region’ accuracy were not used for geospatial analysis, nor were full records documenting test results for captive animals.

^2^Sites with full site location data were resolved to unique latitude and longitude coordinates. These site-level data were used in analyses of environmental associations. Imprecise locations identifiable to general region or a country were tallied as Country Centroids and were not used in further analyses.

^3^Some Bd sample results were categorized as “uncertain” because they have been contested in the literature. Specifically, surveys for *Bd* among Madagascar’s amphibians has been primarily *Bd*-negative (*Bd* no-detection results: Weldon et al. 2008a,b; Crottini et al. 2011; Vredenburg et al. 2012; Weldon et al. 2013; Crottini et al. 2014). Rabemanajara et al. (2011) reported an unconfirmed finding of *Bd* from Makay Massif, Madagascar, providing the impetus for the development of a *Bd* National Monitoring Plan (Weldon et al. 2013). First detections of *Bd* in Madagascar were in amphibians in-transit for trade, tested in captivity (Kolby 2014), hence it was not certain if those animals had been infected in the wild. A rapid follow-up survey of captive and wild amphibians (Kolby et al. 2014) did not detect *Bd* in field-sampled animals. However, Bletz et al. (2015a) reported Bd across the island. These findings were disputed by Kolby and Skerratt (2015) who reported that “Detection of *Bd* at “positive” locations in Madagascar has been inconsistent for unknown reasons.” Bletz et al. (2015b) then reported that the very low *Bd* prevalence (0.0043, 0.0062) at two *Bd*-positive locations during their sampling complements the no-detection data that Kolby et al. (2014) had reported due to confidence intervals of sampling data. As Bd occurrence appears to be under continued investigation in Madagascar at this writing, using an abundance of caution here, we acknowledge these data as uncertain as work in the area continues.

**References**

Bletz MC, Rosa GM, Andreone F, Courtois EA, Schmeller DS, Rabibisoa NHC, Rabemananjara FCE, Raharivololoniaina L, Vences M, Weldon C, Edmonds D, Raxworthy CJ, Harris RN, Fisher MC, Crottini A (2015a) Widespread presence of the pathogenic fungus *Batrachochytrium dendrobatidis* in wild amphibian communities in Madagascar. Scientific Reports 5: 8633 doi: 10.1038/srep08633.

Bletz MC, Rosa GM, Andreone F, Courtois EA, Schmeller DS, Rabibisoa NHC, Rabemananjara FCE, Raharivololoniaina L, Vences M, Weldon C, Edmonds D, Raxworthy CJ, Harris RN, Fisher MC, Crottini A (2015b) Consistency of published results on the pathogen *Batrachochytrium dendrobatidis* in Madagascar: Formal comment on Kolby et al. Rapid response to evaluate the presence of amphibian chytrid fungus (*Batrachochytrium dendrobatidis*) and Ranavirus in wild amphibian populations in Madagascar. PLoSONE 10(10):e0135900. https://doi.org/10.1371/journal.pone.0135900

Crottini A, Barbuto M, Casiraghi M, Andreone F (2011) A rapid amphibian survey at Itremo-Ambatofinandrahana, central Madagascar, with confirmed absence of chytrid fungus and recommendations for future monitoring activities. North-western Journal of Zoology 7(2): 346–351.

Crottini A, Bollen A, Weldon C, Dalton DL, Kotzé A, Noël J, Iambana B, Andreone F (2014) Amphibian survey and current absence of *Batrachochytrium dendrobatidis* in Ivoloina Park, Toamasina (eastern Madagascar). African Journal of Herpetology 63(1): 70–78. doi: 10.1080/21564574.2013.833994

Kolby JE (2014) Presence of the amphibian chytrid fungus *Batrachochytrium dendrobatidis* in native amphibians exported from Madagascar. PLoS ONE 9(3): e89660.

Kolby JE, Skerratt LF (2015) Amphibian chytrid fungus in Madagascar neither shows widespread presence nor signs of certain establishment. PLoS ONE 10(10): e0139172. doi: 10.1371/journal.pone.0139172

Kolby JE, Smith KM, Ramirez SD, Rabemananjara F, Pessier AP, Brunner JL, Goldberg CS, Berger L, Skerratt LF. (2015) Rapid response to evaluate the presence of the amphibian chytrid fungus (*Batrachochytrium dendrobatidis*) and ranavirus in wild amphibian populations in Madagascar. PLoS ONE 10(6): e0125330. doi:10.1371/journal.pone.0125330.

Rabemananjara FCE, Andreone F, Rabibisoa N (2011) Madagascar and chytrid news: needed an urgent action and close collaboration between stakeholders. FrogLog 97: 33.

Vredenburg VT, du Preez L, Raharivololoniaina L, Vieites DR, Vences M, Weldon C (2012) A molecular survey across Madagascar does not yield positive records of the amphibian chytrid fungus *Batrachochytrium dendrobatidis*. Herpetology Notes 5: 507–517.

Weldon C, Du Preez L, Vences M. (2008a) Lack of detection of the amphibian chytrid fungus (*Batrachochytrium dendrobatidis*) in Madagascar. Mon Museo Regionale Scienze Naturali di Torino XLV: 95–106.

Weldon C, Du Preez L, Vences M (2008b) Lack of detection of the amphibian chytrid fungus (*Batrachochytrium dendrobatidis*) in Madagascar. In: Andreone F, ed. A conservation strategy for the amphibians of Madagascar. Monografie del Museo Regionale di Scienze Naturali di Torino, XLV (2008): 95–106.

Weldon C, Crottini A, Bollen A, Rabemananjara FCE, Copsey J, Garcia G, and Andreone F. (2013) Pre-emptive National Monitoring Plan for detecting the amphibian chytrid fungus in Madagascar. EcoHealth 10(3): 234–240. doi: 10.1007/s10393-013-0869-8.
