# Supplementary material for: Global Patterns of the Fungal Pathogen Batrachochytrium dendrobatidis Support Conservation Urgency
Source: Front Vet Sci. 2021 Jul 16;8:685877. doi: 10.3389/fvets.2021.685877 (PMC8322974; doi:10.3389/fvets.2021.685877)
Supplement: Supplementary Appendix 1 — Detailed methods. [file Data_Sheet_1.zip › Data Sheet 1/Table S2_rev03June2021.docx]

**Table S2:** *Batrachochytrium dendrobatidis* detections by species, with references and countries of detection. Family-level taxonomy is shown according to Frost et al. (2020), Grant et al. (2006), and Hedges et al. (2008). Where assignments to genus or species have changed since the species was reported as being found with *Bd*, the older name is given in parentheses. To ensure that all species counted as positive are included, novel (as yet unidentified) species (sp. nov.) are shown; occurrences identified only to genus are shown if they are the only representative of that genus sampled anywhere, or in a particular country, and if several unnamed species with detections were designated as differing from each other by the source, assuming that there were not multiple other named species for that genus already counted from the same or other sources. Uncertain positives are not reported in this table. For further information on taxonomy, see Taxonomic Notes, Appendix S1. The abbreviation (cap.) after a species name designates an infected captive animal or animals. Status is Conservation Status according to the IUCN Red List (IUCN 2020). Categories are defined as: EX = extinct; EW = Extinct in the Wild; CR = Critically Endangered; EN = Endangered; VU = Vulnerable (the previous three categories are considered Threatened by the IUCN); NT = Near Threatened; LC = Least Concern; DD = Data Deficient; NE = Not Evaluated (newly discovered or newly recognized species). Last updated from published literature through December 2019; references follow the table.

**Part 1. Anura**

| **Family** | **Species** | **Status** | **Country** | **Reference** |
| --- | --- | --- | --- | --- |
| **Alsodidae** | *Alsodes nodosus* | NT | Chile | Soto-Azat et al. 2013; Bacigalupe et al. 2017 |
| Alsodidae | *Alsodes pehuenche* | CR | Argentina | Ghirardi 2011; Ghirardi et al. 2014 |
| Alsodidae | *Alsodes tumultuosus* | VU | Chile | Ghirardi et al. 2014 |
| Alsodidae | *Eupsophus calcaratus* | LC | Chile | Bacigalupe et al. 2017 |
| Alsodidae | *Eupsophus contulmoensis* | NE | Chile | Bacigalupe et al. 2017 |
| Alsodidae | *Eupsophus nahuelbutensis* | NE | Chile | Soto-Azat et al. 2013; Bacigalupe et al. 2017 |
| Alsodidae | *Eupsophus roseus* | LC | Chile | Bacigalupe et al. 2017 |
| Alsodidae | *Eupsophus septentrionalis* | NE | Chile | Bacigalupe et al. 2017 |
| **Alytidae^[[1]](#footnote-1)^** | *Alytes dickhilleni* | VU | Spain | Bosch et al. 2013 |
| Alytidae1 | *Alytes muletensis* | VU | Spain | Walker et al. 2008; Gabor et al. 2013; S. Walker, unpub. |
| Alytidae^1^ | *Alytes obstetricans* | LC | Belgium, France, Germany, The Netherlands, Portugal, Spain, Switzerland | Bosch et al. 2001; Garner et al. 2005; Pasmans et al. 2010; Tobler et al. 2012; Böll et al. 2012; Ohst et al. 2013; Rosa et al. 2013; Spitzen-van der Sluijs et al. 2014; Tobler and Schmidt 2010; Tobler et al. 2012; Gabor et al. 2013; Fernández-Beaskoetxea et al. 2015; Rosa et al. 2017; S. Walker, unpub. |
| Alytidae1 | *Discoglossus galganoi* | LC | Spain | Hidalgo-Vila et al. 2012 |
| Alytidae^1^ | *Discoglossus jeannae* | NT | Spain | S. Walker, unpub. |
| Alytidae^1^ | *Discoglossus sardus* | LC | Italy | Bielby et al. 2009 |
| Alytidae^1^ | *Discoglossus scovazzi* | LC | Morocco | El Mouden et al. 2011; El Cadi et al. 2019 |
| **Aromobatidae** | *Allobates conspicuus* | DD | Peru | Russell et al. 2019 |
| **Aromobatidae** | *Allobates femoralis* | LC | French Guiana | Courtois et al. 2015 |
| Aromobatidae | *Allobates marchesianus* | LC | Peru | Kosch et al. 2012 |
| Aromobatidae | *Allobates mcdiarmidi* | NT | Bolivia | Burrowes and de la Riva 2017 |
| Aromobatidae | *Allobates olfersioides* | VU | Brazil | Carnaval et al. 2006 |
| Aromobatidae | *Allobates* sp. | — | Colombia | Acevedo et al. 2016; Flechas et al. 2017 |
| Aromobatidae | *Allobates (Colostethus) talamancae* | LC | Panama | Lips et al. 2006 |
| Aromobatidae | *Allobates trilineatus* | LC | Peru | Russell et al. 2019 |
| Aromobatidae | *Anomaloglossus baeobatrachus* | DD | French Guiana | Courtois et al. 2015 |
| Aromobatidae | *Aromobates meridensis* | CR | Venezuela | Lampo et al. 2008 |
| Aromobatidae | *Aromobates saltuensis* | EN | Colombia | Acevedo et al. 2016; Flechas et al. 2017 |
| Aromobatidae | *Mannophryne collaris* | EN | Venezuela | Sánchez et al. 2008 |
| Aromobatidae | *Mannophryne cordilleriana* | CR | Venezuela | Lampo et al. 2006a |
| Aromobatidae | *Mannophryne herminae* | NT | Venezuela | Nicolás 2007 |
| Aromobatidae | *Mannophryne olmonae* | VU | Trinidad & Tobago | Alemu et al. 2008 |
| Aromobatidae | *Mannophryne trinitatis* | VU | Trinidad & Tobago | Alemu et al. 2013 |
| Aromobatidae | *Rheobates palmatus* | LC | Colombia | Flechas et al. 2013, 2017 |
| **Arthroleptidae** | *Arthroleptis adelphus* | LC | Cameroon | Miller et al. 2018 |
| Arthroleptidae | *Arthroleptis perreti* | EN | Cameroon | Doherty-Bone et al. 2013; Hirschfeld et al. 2016 |
| Arthroleptidae | *Arthroleptis poecilonotus* | LC | Cameroon, Gabon | Doherty-Bone et al. 2013; Hirschfeld et al. 2016; Jongsma et al. 2016; Miller et al. 2018 |
| Arthroleptidae | *Arthroleptis* sp. | — | Rwanda, Uganda | Seimon et al. 2015 |
| Arthroleptidae | *Arthroleptis* sp. Kabobo Plateau | — | Democratic Republic of Congo | Seimon et al. 2015 |
| Arthroleptidae | *Arthroleptis stenodactylus* | LC | Democratic Republic of Congo | Greenbaum et al. 2014 |
| Arthroleptidae | *Arthroleptis sylvaticus* | LC | Cameroon, Gabon | Jongsma et al. 2016; Miller et al. 2018 |
| Arthroleptidae | *Arthroleptis tuberosus* | DD | Cameroon | Doherty-Bone et al. 2013; Hirschfeld et al. 2016 |
| Arthroleptidae | *Arthroleptis variabilis* | LC | Cameroon, Gabon | Doherty-Bone et al. 2013; Jongsma et al. 2016 |
| Arthroleptidae | *Arthroleptis xenodactyloides* | LC | Malawi | Conradie et al. 2011 |
| Arthroleptidae | *Astylosternus batesi* | LC | Cameroon | Hirschfeld et al. 2016 |
| Arthroleptidae | *Astylosternus diadematus* | LC | Cameroon | Hirschfeld et al. 2016 |
| Arthroleptidae | *Astylosternus fallax* | VU | Cameroon | Hirschfeld et al. 2016 |
| Arthroleptidae | *Astylosternus montanus* | LC | Cameroon | Hirschfeld et al. 2016 |
| Arthroleptidae | *Astylosternus perreti* | LC | Cameroon | Hirschfeld et al. 2016 |
| Arthroleptidae | *Astylosternus ranoides* | EN | Cameroon | Doherty-Bone et al. 2013; Hirschfeld et al. 2016 |
| Arthroleptidae | *Astylosternus* sp.^[[2]](#footnote-2)^ | — | Nigeria | Reeder et al. 2011 |
| Arthroleptidae | *Cardioglossa elegans* | LC | Cameroon, Gabon | Bell et al. 2011; Hirschfeld et al. 2016 |
| Arthroleptidae | *Cardioglossa escalerae* | LC | Cameroon | Hirschfeld et al. 2016 |
| Arthroleptidae | *Cardioglossa gracilis* | LC | Cameroon | Doherty-Bone et al. 2013; Hirschfeld et al. 2016 |
| Arthroleptidae | *Cardioglossa leucomystax* | LC | Cameroon | Doherty-Bone et al. 2013; Miller et al. 2018 |
| Arthroleptidae | *Cardioglossa melanogaster* | VU | Cameroon | Doherty-Bone et al. 2013; Hirschfeld et al. 2016 |
| Arthroleptidae | *Leptodactylodon erythrogaster* | CR | Cameroon | Doherty-Bone et al. 2013; Hirschfeld et al. 2016 |
| Arthroleptidae | *Leptodactylodon mertensi* | EN | Cameroon | Doherty-Bone et al. 2013; Hirschfeld et al. 2016 |
| Arthroleptidae | *Leptopelis aubryi* | LC | Cameroon, Gabon | Bell et al. 2011; Hirschfeld et al. 2016; Jongsma et al. 2016; Miller et al. 2018 |
| Arthroleptidae | *Leptopelis aubryioides (omissus)* | LC | Cameroon | Miller et al. 2018 |
| Arthroleptidae | *Leptopelis boulengeri* | LC | Cameroon | Hirschfeld et al. 2016 |
| Arthroleptidae | *Leptopelis brevirostris* | LC | Cameroon | Bell et al. 2011; Doherty-Bone et al. 2013; Hirschfeld et al. 2016; Miller et al. 2018 |
| Arthroleptidae | *Leptopelis calcaratus* | LC | Cameroon | Doherty-Bone et al. 2013; Greenbaum et al. 2015; Hirschfeld et al. 2016; Miller et al. 2018 |
| Arthroleptidae | *Leptopelis christyi* | LC | Uganda | Goldberg et al. 2007 |
| Arthroleptidae | *Leptopelis crystallinoron* | DD | Gabon | Bell et al. 2011 |
| Arthroleptidae | *Leptopelis gramineus* | LC | Ethiopia | Gower et al. 2012 |
| Arthroleptidae | *Leptopelis karissimbensis* | VU | DR Congo | Greenbaum et al. 2015; Seimon et al. 2015 |
| Arthroleptidae | *Leptopelis karissimbensis/kivuensis* | — | Rwanda | van der Hoek et al. 2019 |
| Arthroleptidae | *Leptopelis kivuensis* | LC | DR Congo, Uganda | Goldberg et al. 2007; Greenbaum et al. 2015; Seimon et al. 2015 |
| Arthroleptidae | *Leptopelis millsoni* | LC | Cameroon, Gabon | Bell et al. 2011; Hirschfeld et al. 2016; Jongsma et al. 2016 |
| Arthroleptidae | *Leptopelis modestus* | LC | Cameroon | Doherty-Bone et al. 2013; Hirschfeld et al. 2016 |
| Arthroleptidae | *Leptopelis mtoewaate* | NE | DR Congo | Greenbaum et al. 2015 |
| Arthroleptidae | *Leptopelis nordequatorialis* | LC | Cameroon | Hirschfeld et al. 2016 |
| Arthroleptidae | *Leptopelis notatus* | LC | Cameroon | Doherty-Bone et al. 2013; Hirschfeld et al. 2016; Miller et al. 2018 |
| Arthroleptidae | *Leptopelis ocellatus* | LC | Cameroon, Gabon | Jongsma et al. 2016; Miller et al. 2018 |
| Arthroleptidae | *Leptopelis parbocagii* | LC | DR Congo | Greenbaum et al. 2014 |
| Arthroleptidae | *Leptopelis ragazzii* | VU | Ethiopia | Gower et al. 2012 |
| Arthroleptidae | *Leptopelis rufus* | LC | Cameroon, Gabon | Hirschfeld et al. 2016; Jongsma et al. 2016 |
| Arthroleptidae | *Leptopelis spiritusnoctis* | LC | Nigeria | Imasuen et al. 2011 |
| Arthroleptidae | *Leptopelis uluguruensis* | NT | Japan (cap.) | Tamukai et al. 2014 (cap.) |
| Arthroleptidae | *Leptopelis vannutellii* | LC | Ethiopia | Gower et al. 2012 |
| Arthroleptidae | *Leptopelis vermiculatus* | EN | The Netherlands (cap.) | Spitzen-van der Sluijs et al. 2011 (cap.) |
| Arthroleptidae | *Leptopelis viridis (hyloides)* | LC | Cameroon, Nigeria | Imasuen et al. 2011; Miller et al. 2018 |
| Arthroleptidae | *Leptopelis xenodactylus* | EN | South Africa | Tarrant et al. 2013 |
| Arthroleptidae | *Nyctibates corrugatus* | LC | Cameroon | Hirschfeld et al. 2016; Miller et al. 2018 |
| Arthroleptidae | *Scotobleps gabonicus* | LC | Cameroon, Gabon | Bell et al. 2011; Hirschfeld et al. 2016; Jongsma et al. 2016; Miller et al. 2018 |
| Arthroleptidae | *Trichobatrachus robustus* | LC | Cameroon | Hirschfeld et al. 2016 |
| **Ascaphidae^[[3]](#footnote-3)^** | *Ascaphus truei* | LC | United States | Hossack et al. 2010 |
| **Batrachylidae** | *Atelognathus patagonicus* | CR | Argentina, Chile | Fox et al. 2006; Ghirardi 2011; Ghirardi et al. 2014 |
| Batrachylidae | *Atelognathus reverberii* | VU | Argentina | Arellano et al. 2015 |
| Batrachylidae | *Batrachyla antartandica* | LC | Chile | Bacigalupe et al. 2017 |
| Batrachylidae | *Batrachyla leptopus* | LC | Chile | Bourke et al. 2011 |
| Batrachylidae | *Batrachyla taeniata* | LC | Chile | Bacigalupe et al. 2017 |
| Batrachylidae | *Hylorina sylvatica* | LC | Chile | Bacigalupe et al. 2017 |
| **Bombinatoridae** | *Bombina bombina* | LC | Austria, Czech Republic, Germany, Hungary, Sweden | Sztatecsny and Glaser 2011; Civiš et al. 2012; Baláž et al. 2014 Cons Biol, 2014 Dis Aquat Org; Ohst et al. 2013; Kärvemo et al. 2018; Vörös et al. 2018 |
| Bombinatoridae | *Bombina orientalis* | LC | South Korea; Hong Kong (cap.), United States (cap.) | Swei et al. 2011; Bataille et al. 2013; Shin et al. 2014; Kolby et al. 2014 (cap.); Pessier and Mendelson unpub (cap.) |
| Bombinatoridae | *Bombina pachypus* | EN | Italy | Stagni et al. 2002, 2004; Canestrelli et al. 2013 |
| Bombinatoridae | *Bombina variegata* | LC | Albania, Austria, Belgium, Czeck Republic, Germany, Hungary, The Netherlands, Poland, Romania | Sztatecsny and Glaser 2011; Spitzen-van der Sluijs et al. 2011 (cap.); Civiš et al. 2012; Gál et al. 2012; Vörös et al. 2013; Ohst et al. 2013; Baláž et al. 2014 Cons Biol, 2014 Dis Aquat Org; Spitzen-van der Sluijs et al. 2014; Scheele et al. 2015 Anim Cons; Kolenda et al. 2017; Vojar et al. 2017; Wagner et al. 2017; Vörös et al. 2018; Vörös et al. unpub. |
| **Brachycephalidae** | *Brachycephalus didactylus* | LC | Brazil | Rodriguez et al. 2014 |
| Brachycephalidae | *Brachycephalus epihippium* | LC | Brazil | Ruggeri et al. 2018 Dis Aquat Org |
| Brachycephalidae | *Ischnocnema erythromera* | DD | Brazil | Ruggeri et al. 2018 Dis Aquat Org |
| Brachycephalidae | *Ischnocnema guentheri* | LC | Brazil | Gründler et al. 2012; Rodriguez et al. 2014; Coutinho et al. 2015; Ruggeri et al. 2018 Dis Aquat Org |
| Brachycephalidae | *Ischnocnema henselii* | LC | Brazil | Rodriguez et al. 2014 |
| Brachycephalidae | *Ischnocnema holti* | DD | Brazil | Ruggeri et al. 2018 Dis Aquat Org |
| Brachycephalidae | *Ischnocnema nasuta* | LC | Brazil | Rodriguez et al. 2014 |
| Brachycephalidae | *Ischnocnema parva* | LC | Brazil | Rodriguez et al. 2014; Ruggeri et al. 2018 Dis Aquat Org |
| Brachycephalidae | *Ischnocnema randorum* | DD | Brazil | Coutinho et al. 2015 |
| **Brevicepitidae** | *Balebreviceps hillmani* | CR | Ethiopia | Gower et al. 2012 |
| **Bufonidae** | *Altiphrynoides malcolmi* | EN | Ethiopia | Gower et al. 2012 |
| Bufonidae | *Amazophrynella minuta* | LC | Peru | Russell et al. 2019 |
| Bufonidae | *Anaxyrus (Bufo) americanus* | LC | Canada, United States | Ouellet et al. 2005; Longcore et al. 2007; Zippel and Tabaka 2008; Rodriguez et al. 2009; Davidson and Chambers 2011; Lanoo et al. 2011; Krynak et al. 2012; Wolff et al. 2012; Richards-Hrdlicka et al. 2013; Muelleman and Montgomery 2013; Phillips et al. 2014; Petersen et al. 2016; Marhanka et al. 2017; Tupper et al. 2017; Watters et al. 2018, 2019; A. Pessier and J. Mendelson, unpub. |
| Bufonidae | *Anaxyrus (Bufo) baxteri* | EW | United States | A. Pessier and J. Mendelson, unpub.; J. Polasik, US Fish and Wildife Service unpub. report |
| Bufonidae | *Anaxyrus (Bufo) boreas* | NT | Canada, Mexico, United States | Raverty and Reynolds 2001; Morehouse et al. 2003; Muths et al. 2003; Rogers and Banulis 2004; Thompson et al. 2004; Green and Muths 2005; Adams et al. 2007, 2010; Pearl et al. 2007; Young et al. 2007; Muths et al. 2003, 2008; Deguise and Richardson 2009; Hasken et al. 2009; Murphy et al. 2009; Schock et al. 2009; Slough 2009; MJ Adams et al. 2010; Pilliod et al. 2010; Fellers et al. 2011; Piovia-Scott et al. 2011; Stevens et al. 2012; Richardson et al. 2014; Addis et al. 2015; Ecoclub Amphibian Group et al. 2016; AJ Adams et al. 2017 Ecol Evol, 2017 Ecosphere;Stutz et al. 2017; Mosher et al. 2018; Peralta-García et al. 2018; Unpub. data from Canadian Cooperative Wildlife Health Centre; E. Bull; D.E. Green; M. Hahr; P. Johnson; K. Kendell; B. Maxell; J. Moore; D. Pilliod and E. Muths; D. Sumerlin; C. Tait; S. Wagner and J. Johnson |
| Bufonidae | *Anaxyrus (Bufo) californicus* | EN | Mexico, United States | AJ Adams et al. 2017 Ecol Evol; Peralta-García et al. 2018; R. Fisher, unpub.; D.E. Green, unpub. |
| Bufonidae | *Anaxyrus (Bufo) canorus* | EN | United States | Carey et al. 1999; Green and Kagarise Sherman 2001; Fellers et al. 2011; Unpub. data from: C. Brown; A. Lind and R. Grasso; G. Padgett-Flohr; U.S. Forest Service Sierra Nevada Amphibian Monitoring Program |
| Bufonidae | *Anaxyrus cognatus* | LC | Mexico, United States | Brown and Kerby 2013; Watters et al. 2016; Hernández-Martínez et al. 2019; Suriyamongkol et al. 2019; Todd et al. 2019; A. Pessier and J. Mendelson, unpub. (cap.) |
| Bufonidae | *Anaxyrus debilis* | LC | Mexico, United States | Hernández-Martínez et al. 2019; Suriyamongkol et al. 2019 |
| Bufonidae | *Anaxyrus fowleri* | LC | United States | Davis et al. 2012; Lannoo et al. 2011; A. Pessier and J. Mendelson, unpub. (cap.) |
| Bufonidae | *Anaxyrus hemiophrys* | LC | Canada | Stevens et al. 2012 |
| Bufonidae | *Anaxyrus (Bufo) houstonensis* | EN | United States | Gaertner et al. 2007, 2010 |
| Bufonidae | *Anaxyrus microscaphus* | LC | United States | Ryan et al. 2014 |
| Bufonidae | *Anaxyrus nelsoni* | EN | United States | Forrest et al. 2015 |
| Bufonidae | *Anaxyrus (Bufo) punctatus* | LC | Mexico, United States | Sredl et al. 2002; Hernández-Martínez et al. 2019; Todd et al. 2019; R. Fisher, unpub. |
| Bufonidae | *Anaxyrus speciosus* | LC | United States | Suriyamongkol et al. 2019 |
| Bufonidae | *Anaxyrus (Bufo) terrestris* | LC | United States | Rizkalla 2010; Love et al. 2016; Chiari et al. 2017; A. Pessier and J. Mendelson, unpub. (cap.) |
| Bufonidae | *Anaxyrus (Bufo) woodhousii* | LC | United States | Carey and Livo 2009; Saenz et al. 2010; Lannoo et al. 2011; Brown and Kerby 2013; Harner et al. 2013; McTaggart et al. 2014; Peterson and McKenzie 2014; Watters et al. 2016, 2019; Marhanka et al. 2017; Suriyamongkol et al. 2019; B. Maxell, unpub. |
| Bufonidae | *Atelopus bomolochos* | CR | Ecuador | Ron and Merino-Viteri 2000 |
| Bufonidae | *Atelopus carbonerensis* | CR | Venezuela | Lampo et al. 2006a |
| Bufonidae | *Atelopus chiriquiensis* | CR | Costa Rica, Panama | Lips et al. 2003; Berger et al. 1998 (cap.); Puschendorf et al. 2009 |
| Bufonidae | *Atelopus cruciger* | CR | Venezuela | Bonaccorso et al. 2003; Rodríguez-Contreras et al. 2008; Lampo et al. 2017 |
| Bufonidae | *Atelopus elegans* | EN | Colombia | Flechas et al. 2012, 2015, 2017 |
| Bufonidae | *Atelopus flavescens* | VU | French Guiana | Courtois et al. 2015 |
| Bufonidae | *Atelopus ignescens* | EX | Ecuador | Ron and Merino-Viteri 2000 |
| Bufonidae | *Atelopus laetissimus* | EN | Colombia | Flechas et al. 2017 |
| Bufonidae | *Atelopus mittermeieri* | EN | Colombia | Ruiz and Rueda-Almonacid 2008 |
| Bufonidae | *Atelopus mucabajiensis* | CR | Venezuela | Lampo et al. 2006a, 2006b |
| Bufonidae | *Atelopus patazensis* | CR | Peru | Venegas et al. 2008 |
| Bufonidae | *Atelopus pulcher* | CR | Peru | Lötters et al. 2005 |
| Bufonidae | *Atelopus sorianoi* | CR | Venezuela | Lampo et al. 2006a |
| Bufonidae | *Atelopus spurrelli* | NT | Colombia | Flechas et al. 2015, 2017 |
| Bufonidae | *Atelopus tricolor* | VU | Bolivia | Burrowes and de la Riva 2017 |
| Bufonidae | *Atelopus varius* | CR | Costa Rica, Panama | Berger et al. 1998; Lips et al. 2003; Puschendorf 2003; Puschendorf et al. 2006a, 2009 |
| Bufonidae | *Atelopus zeteki* | CR | Panama | Lips et al. 2006; Richards-Zawacki 2010; Velo-Antón 2012 |
| Bufonidae | *Bufo bufo* | LC | Belgium, Czech Republic, Germany, The Netherlands, Spain, Russian Federation, Sweden, United Kingdom; United States (cap.) | Garner et al. 2005, 2009; Bosch and Martinez-Solano 2006; Arai 2008; Cunningham and Minting 2008; Martel et al. 2012; Rasmussen et al. 2012; Spitzen-van der Sluijs et al. 2014; Ohst et al. 2013; Baláž et al. 2014; Reshetnikov et al. 2014; Tinsley et al. 2015; Kärvemo et al. 2019; Unpub. data from: J. Bosch; TWJ Garner; S. Walker; A. Pessier and J. Mendelson (cap.) |
| Bufonidae | *Bufo gargarizans* | LC | China, South Korea | Yang et al. 2009; Bai et al. 2012; Bataille et al. 2013; Zhu et al. 2014, 2016 |
| Bufonidae | *Bufo japonicus* | LC | Japan (cap.) | S. Okada, unpub. |
| Bufonidae | *Bufo spinosus* | NE | Portugal | Rosa et al. 2017 |
| Bufonidae | *Bufo stejnegeri* | LC | South Korea | Bataille et al. 2013 |
| Bufonidae | *Bufotes pewzowi* | LC | China, Kyrgyzstan | Swei et al. 2011; Zhu et al. 2016 |
| Bufonidae | *Bufotes turanensis* | LC | Kyrgyzstan | Swei et al. 2011 |
| Bufonidae | *Bufotes variabilis* | DD | Sweden, Turkey | Erismis et al. 2014; Kärvemo et al. 2019 |
| Bufonidae | *Bufotes viridis* | LC | Czech Republic, Germany, Greece, Hungary | Baláž et al. 2013; Ohst et al. 2013; Azmanis et al. 2016; Vörös et al. 2018 |
| Bufonidae | *Duttaphrynus melanostictus* | LC | Cambodia, Singapore | Gaertner et al. 2011; Mendoza et al. 2011; Chong et al. 2018 |
| Bufonidae | *Epidalea (Bufo) calamita* | LC | Czech Republic, Germany, The Netherlands, Spain, Sweden, United Kingdom | Arai 2008; Cunningham and Minting 2008; Baláž et al. 2013; Ohst et al. 2013; Spitzen-van der Sluijs et al. 2014; Kärvemo et al. 2018; T. Garner, unpub.; S. Walker, unpub. |
| Bufonidae | *Frostius pernambucensis* | LC | Brazil | Valencia-Aguilar et al. 2015 |
| Bufonidae | *Incilius alvarius* | LC | Japan (cap.) | Tamukai et al. 2014 (cap.) |
| Bufonidae | *Incilius campbelli* | NT | Belize | Kaiser and Pollinger 2012 |
| Bufonidae | *Incilius coniferus* | LC | Panama | Brem and Lips 2008 |
| Bufonidae | *Incilius luetkenii* | LC | Honduras | Gutsche et al. 2015 |
| Bufonidae | *Incilius melanochlorus* | LC | Costa Rica | Zumbado-Ulate et al. 2019 |
| Bufonidae | *Incilius (Bufo) nebulifer* | LC | United States | Gaertner et al. 2010; Brannelly et al. 2018 BMC Ecol |
| Bufonidae | *Incilius (Bufo) occidentalis* | LC | Mexico | D.E. Green, unpub. |
| Bufonidae | *Incilius (Bufo) periglenes* | EX | Costa Rica | Lips et al. 2006 |
| Bufonidae | *Incilius valliceps* | LC | Honduras, Nicaragua, Mexico | García-Roa et al. 2014; Gutsche et al. 2015; Köhler et al. 2019 Mexico |
| Bufonidae | *Ingerophrynus celebensis* | LC | Indonesia | Swei et al. 2011 |
| Bufonidae | *Ingerophrynus macrotis* | LC | Cambodia | Gaertner et al. 2011 |
| Bufonidae | *Ingerophrynus parvus* | LC | Thailand | Vörös et al. 2012 |
| Bufonidae | *Leptophryne cruentata* | CR | Indonesia | Kusrini et al. 2008 |
| Bufonidae | *Melanophryniscus dorsalis* | VU | Brazil | Rodriguez et al. 2014 |
| Bufonidae | *Melanophryniscus montevidensis* | VU | Argentina | Agostini et al. 2015 |
| Bufonidae | *Melanophryniscus moreirae* | NT | Brazil | Van Sluys et al. 2007; Ferriera et al. 2008; Rodriguez et al. 2014 |
| Bufonidae | *Melanophryniscus montevidensis* | VU | Argentina | Agostini et al. 2015 |
| Bufonidae | *Nectophryne afra* | LC | Cameroon | Miller et al. 2018 |
| Bufonidae | *Nectophryne batesii* | LC | DR Congo | Greenbaum et al. 2015 |
| Bufonidae | *Nectophrynoides asperginis* | EW | Tanzania | Weldon and du Preez 2004; Makange et al. 2014; Weldon et al. 2020 |
| Bufonidae | *Nectophrynoides* spp.^[[4]](#footnote-4)^ | NE | Tanzania | Moyer and Weldon 2006 |
| Bufonidae | *Peltophryne lemur* | CR | United States (cap.) | A. Pessier and J. Mendelson, unpub. |
| Bufonidae | *Peltophryne (Bufo) longinasus, P. l. longinasus*, or *P. l. dunni* | EN | Cuba | Díaz et al. 2007; Sabino-Pinto et al. 2017; Cádiz et al. 2018 |
| Bufonidae | *Peltophryne (Bufo) taladai* | VU | Cuba | A. Rodriguez, unpub. |
| Bufonidae | *Rhaebo (Bufo) haematiticus* | LC | Costa Rica, Panama | Berger et al. 1998; Lips et al. 2006; Whitfield et al. 2012; Zumbado-Ulate et al. 2019 |
| Bufonidae | *Rhinella achalensis* | NT | Argentina | Lescano et al. 2013 |
| Bufonidae | *Rhinella arenarum* | LC | Argentina | Lescano et al. 2013 |
| Bufonidae | *Rhinella castaneotica* | LC | French Guiana | Courtois et al. 2015 |
| Bufonidae | *Rhinella crucifer* | LC | Brazil | Valencia-Aguilar et al. 2015; Ruggeri et al. 2018 Dis Aquat Org |
| Bufonidae | *Rhinella fernandezae* | LC | Argentina | Agostini and Burrowes 2015 |
| Bufonidae | *Rhinella humboldti* | NE | Colombia | Acevado et al. 2016; Flechas et al. 2017 |
| Bufonidae | *Rhinella icterica* | LC | Brazil | Ruggeri et al. 2018 Dis Aquat Org |
| Bufonidae | *Rhinella inca* | LC | Peru | Catenazzi et al. 2011 |
| Bufonidae | *Rhinella justinianoi* | VU | Bolivia | Burrowes and de la Riva 2017 |
| Bufonidae | *Rhinella manu* | LC | Peru | Warne et al. 2016 |
| Bufonidae | *Rhinella margaritifera* | LC | Peru | Russell et al. 2019 |
| Bufonidae | *Rhinella marina (Bufo marinus)* | LC | Australia, Colombia, Honduras, Nicaragua, Panama, Trinidad and Tobago, United States, Venezuela | Berger et al. 1998 (cap.); Brem and Lips 2008; Lips et al. 2006; Sánchez et al. 2008; Murray et al. 2010; Patel et al. 2012; Lettoof et al. 2013; García-Roa et al. 2014; Gutsche et al. 2015; Acevedo et al. 2016; Flechas et al. 2017; Goodman et al. 2019; Russell et al. 2019; K. Murray, unpub. |
| Bufonidae | *Rhinella ornata* | LC | Brazil | Coutinho et al. 2015 |
| Bufonidae | *Rhinella quechua* | VU | Bolivia | Barrionuevo et al. 2008; de la Riva and Burrowes 2011; Burrowes and de la Riva 2017 |
| Bufonidae | *Rhinella rubescens* | LC | Brazil | Amorim et al. 2019 |
| Bufonidae | *Rhinella (Bufo) spinulosa, R. s. papillosa, R. s. spinulosa* | LC | Argentina, Bolivia, Chile, Peru | Ghirardi 2011; Solís et al. 2015; Burrowes and de la Riva 2017; Ghirardi et al. 2017 Herp Rev; Seimon et al. 2017; R. Solís, unpub. |
| Bufonidae | *Rhinella sternosignata* | NT | Colombia | Flechas et al. 2017 |
| Bufonidae | *Rhinella veraguensis* | LC | Bolivia | Burrowes and de la Riva 2017 |
| Bufonidae | *Sclerophrys capensis* | LC | South Africa | C. Weldon, unpub. |
| Bufonidae | *Sclerophrys funerea (Amietophrynus, Bufo funereus)* | LC | Uganda | Goldberg et al. 2007 |
| Bufonidae | *Sclerophrys gracilipes* | LC | Cameroon, Gabon | Hirschfeld et al. 2016; Jongsma et al. 2016 |
| Bufonidae | *Sclerophrys (Amietophrynus) gutturalis* | LC | Kenya | Kielgast et al. 2009 |
| Bufonidae | *Sclerophrys maculata (Amietophrynus maculatus)* | LC | Cameroon | Doherty-Bone et al. 2013 |
| Bufonidae | *Sclerophrys mauritanica* | LC | Morocco | El Cadi et al. 2019 |
| Bufonidae | *Sclerophrys (Amietophrynus) pantherinus* | EN | South Africa | Tarrant et al. 2013 |
| Bufonidae | *Sclerophrys (Amietophrynus) rangeri* | LC | South Africa | C. Weldon, unpub. |
| Bufonidae | *Sclerophrys (Amietophrynus) regularis* | LC | Cameroon, Gabon, Kenya | Kielgast et al. 2009; Hirschfeld et al. 2016; Jongsma et al. 2016 |
| Bufonidae | *Vandijkophrynus (Bufo) robinsoni* | LC | South Africa | Weldon 2005 |
| Bufonidae | *Wolterstoffina mirei* | EN | Cameroon | Dohertery-Bone et al. 2013; Hirschfeld et al. 2016 |
| Bufonidae | *Wolterstorffina parvipalmata* | CR | Cameroon | Miller et al. 2018 |
| **Calyptocephalellidae** | *Calyptocephalella gayi* | VU | Chile | Soto-Azat and Cunningham 2010, 2016; Bacigalupe et al. 2017 |
| Calyptocephalellidae | *Telmatobufo bullocki* | EN | Chile | Fenolio et al. 2013 |
| **Centrolenidae** | *Centrolene buckleyi* | VU | Colombia | Velásquez-E. et al. 2008 |
| Centrolenidae | *Centrolene daidaleum* | VU | Colombia | Acevedo et al. 2016; Flechas et al. 2017 |
| Centrolenidae | *Cochranella euknemos* | LC | Panama | Lips et al. 2006 |
| Centrolenidae | *Cochranella granulosa* | LC | Costa Rica | Zumbado-Ulate et al. 2019 |
| Centrolenidae | *Espadarana andina* | LC | Colombia | Acevedo et al. 2016; Flechas et al. 2017 |
| Centrolenidae | *Espadarana (Centrolene, Centrolenella, Cochranella) prosoblepon* | LC | Costa Rica, Panama | Berger et al. 1998; Lips et al. 2006; Picco and Collins 2007; Whitfield et al. 2013 |
| Centrolenidae | *Hyalinobatrachium bergeri* | LC | Peru | Catenazzi et al. 2011 |
| Centrolenidae | *Hyalinobatrachium colymbiphyllum* | LC | Panama | Lips et al. 2006 |
| Centrolenidae | *Hyalinobatrachium fleischmanni* | LC | Belize | Kaiser and Pollinger 2012 |
| Centrolenidae | *Hyalinobatrachium valerioi* | LC | Costa Rica | Goldberg et al. 2009 |
| Centrolenidae | *Nymphargus bejaranoi* | LC | Bolivia | Burrowes and de la Riva 2017 |
| Centrolenidae | *Nymphargus (Cochranella) griffithsi* | VU | Colombia | Velásquez-E. et al. 2008 |
| Centrolenidae | *Sachatamia (Cochranella) albomaculata* | LC | Panama | Berger et al. 1998; Lips et al. 2006 |
| Centrolenidae | *Sachatamia (Centrolene) ilex* | LC | Panama | Lips et al. 2006 |
| Centrolenidae | *Teratohyla pulverata* | LC | Costa Rica | Zumbado-Ulate et al. 2019 |
| Centrolenidae | *Teratohyla spinosa* | LC | Costa Rica | Whitfield et al. 2013; Zumbado-Ulate et al. 2019 |
| Centrolenidae | *Vitreorana eurygnatha* | LC | Brazil | Rodriguez et al. 2013 |
| Centrolenidae | *Vitreorana uranoscopa* | LC | Brazil | Rodriguez et al. 2013 |
| **Ceratobatrachidae** | *Cornufer guentheri* | LC | United States (cap.) | A. Pessier and J. Mendelson, unpub. |
| Ceratobatrachidae | *Platymantis corrugatus* | LC | Philippines | Smith et al. 2019 |
| **Ceratophryidae** | *Ceratophrys aurita* | LC | Brazil | Rodriguez et al. 2013 |
| Ceratophryidae | *Ceratophrys calcarata* | LC | Japan (cap.), Peru | Une et al. 2008 (cap.); Tamukai et al. 2014 (cap.); Russell et al. 2019 |
| Ceratophryidae | *Ceratophrys cornuta* | LC | Japan (cap.) | Une et al. 2008; Goka et al. 2009 |
| Ceratophryidae | *Ceratophrys cranwelli* | LC | Japan (cap.), United States (cap.) | Une et al. 2008 (cap.); Goka et al. 2009 (cap.); Tamukai et al. 2014 (cap.); A. Pessier and J. Mendelson, unpub |
| Ceratophryidae | *Ceratophrys ornata* | NT | Japan (cap.), United States (cap.) | Goka et al. 2009 (cap.); Churgin et al. 2013 (cap.); Tamukai et al. 2014 (cap.); A. Pessier and J. Mendelson, unpub. (cap.) |
| Ceratophryidae | *Chacophrys pierottii* | LC | Japan (cap.) | Une et al. 2008 (cap.); Tamukai et al. 2014 (cap.) |
| Ceratophryidae | *Lepidobatrachus laevis* | LC | Japan (cap.) | Une et al. 2008 (cap.); Goka et al. 2009 (cap.); Tamukai et al. 2014 (cap.) |
| **Conrauidae** | *Conraua crassipes* | LC | Gabon | Bell et al. 2011; Jongsma et al. 2016 |
| Conrauidae | *Conraua derooi* | CR | United States (cap.) | A. Pessier and J. Mendelson unpub. |
| **Craugastoridae**  (Strabomantidae)^[[5]](#footnote-5)^ | *Bryophryne cophites* | EN | Peru | Catenazzi et al. 2011, 2014 |
| Craugastoridae | *Bryophryne zonalis* | DD | Peru | Catenazzi et al. 2011 |
| Craugastoridae | *Craugastor andi* | CR | Costa Rica | Cheng et al. 2011 |
| Craugastoridae | *Craugastor angelicus* | CR | Costa Rica | Cheng et al. 2011 |
| Craugastoridae^[[6]](#footnote-6)^ | *Craugastor (Eleutherodactylus) aurilegulus* | EN | Honduras | Puschendorf et al. 2006b |
| Craugastoridae | *Craugastor azueroensis* | EN | Panama | Lips et al. 2006 |
| Craugastoridae | *Craugastor bransfordii* | LC | Costa Rica, Panama | Lips et al. 2006; Puschendorf et al. 2006a, 2009; Saenz et al. 2009; Whitfield et al. 2012; Zumbado-Ulate et al. 2019 |
| Craugastoridae | *Craugastor chac* | NT | Belize | Kaiser and Pollinger 2012 |
| Craugastoridae | *Craugastor crassidigitus* | LC | Costa Rica, Panama | Lips et al. 2006; Puschendorf et al. 2006a, 2009; Saenz et al. 2009; Zumbado-Ulate et al. 2019 |
| Craugastoridae | *Craugastor (Eleutherodactylus) emcelae* | CR | Panama | Berger et al. 1998 |
| Craugastoridae | *Craugastor fitzingeri* | LC | Costa Rica, Nicaragua | Puschendorf et al. 2006a, 2009; Goldberg et al. 2009; García-Roa et al. 2014; Rebollar et al. 2014; Zumbado-Ulate et al. 2019; C. Goldberg, T. Hawley, and L. Watts, unpub. |
| Craugastoridae | *Craugastor gollmeri* | LC | Panama | Lips et al. 2006 |
| Craugastoridae | *Craugastor greggi* | CR | Guatemala | Cheng et al. 2011 |
| Craugastoridae | *Craugastor laevissimus* | EN | Honduras | Gutsche et al. 2015 |
| Craugastoridae | *Craugastor lauraster* | LC | Honduras | Gutsche et al. 2015 |
| Craugastoridae | *Craugastor loki* | LC | Mexico | Murrieta-Galindo et al. 2014 |
| Craugastoridae | *Craugastor longirostris* | LC | Colombia | Flechas et al. 2017 |
| Craugastoridae | *Craugastor megacephalus* | LC | Costa Rica, Panama | Lips et al. 2006; Whitfield et al. 2013; Zumbado-Ulate et al. 2019 |
| Craugastoridae | *Craugastor (Eleutherodactylus) melanostictus* | LC | Costa Rica | Puschendorf et al. 2009 |
| Craugastoridae | *Craugasor mexicanus* | LC | Mexico | Velo-Antón et al. 2012 |
| Craugastoridae | *Craugastor mimus* | LC | Costa Rica | Whitfield et al. 2013; Zumbado-Ulate et al. 2019 |
| Craugastoridae | *Craugastor noblei* | LC | Costa Rica, Panama | Lips et al. 2006; Saenz et al. 2009 |
| Craugastoridae | *Craugastor podiciferus* | NT | Costa Rica, Panama | Lips et al. 2006; Puschendorf et al. 2006a, 2009; Cheng et al. 2011 |
| Craugastoridae | *Craugastor punctariolus* | EN | Panama | Lips et al. 2006 |
| Craugastoridae | *Craugastor ranoides* | CR | Costa Rica | Zumbado-Ulate et al. 2014; Whitfield et al. 2017 |
| Craugastoridae | *Craugastor rhodopis* | VU | Mexico | Murrieta-Galindo et al. 2014 |
| Craugastoridae | *Craugastor rostralis* | NT | Honduras | Kolby et al. 2010 |
| Craugastoridae | *Craugastor sabrinus* | NT | Belize | Kaiser and Pollinger 2012 |
| Craugastoridae | *Craugastor (Eleutherodactylus) saltator (mexicanus)* | LC | Mexico | Lips et al. 2004 |
| Craugastoridae | *Craugastor stejnegerianus* | LC | Costa Rica | Zumbado-Ulate et al. 2019 |
| Craugastoridae | *Craugastor tabasarae* | CR | Panama | Lips et al. 2006 |
| Craugastoridae | *Craugastor talamancae* | LC | Costa Rica, Panama | Lips et al. 2006; Puschendorf et al. 2006a, 2009 |
| Craugastoridae | *Craugastor taurus* | CR | Costa Rica | Whitfield et al. 2017; Zumbado-Ulate et al. 2019 |
| Craugastoridae | *Craugastor (Eleutherodactylus) underwoodi* | LC | Costa Rica | Picco and Collins 2007 |
| Craugastoridae | *Haddadus binotatus* | LC | Brazil | Gründler et al. 2012; Ruggeri et al. 2018 Dis Aquat Org |
| Craugastoridae | *Holoaden luederwaldti* | DD | Brazil | Rodriguez et al. 2014 |
| Craugastoridae | *Microkayla teqta* | NE | Bolivia | Burrowes and de la Riva 2017 |
| Craugastoridae | *Microkayla wettsteini* | VU | Bolivia | Burrowes and de la Riva 2017 |
| Craugastoridae  (Strabomantidae) | *Noblella* sp.^[[7]](#footnote-7)^ | — | Peru | Catenazzi et al. 2011 |
| Craugastoridae | *Oreobates amarakaeri* | NE | Peru | Russell et al. 2019 |
| Craugastoridae | *Oreobates cruralis* | LC | Bolivia | Burrowes and de la Riva 2017 |
| Craugastoridae | *Oreobates quixensis* | LC | Peru | Russell et al. 2019 |
| Craugastoridae | *Pristimantis achatinus* | LC | Colombia | Flechas et al. 2012, 2017 |
| Craugastoridae | *Pristimantis anolirex* | NT | Colombia | Acevedo et al. 2016 |
| Craugastoridae (Strabomantidae) | *Pristimantis aureolineatus* | LC | Ecuador | McCracken et al. 2009 |
| Craugastoridae | *Pristimantis brevifrons* | LC | Colombia | Flechas et al. 2017 |
| Craugastoridae | *Pristimantis buccinator* | LC | Peru | Russell et al. 2019 |
| Craugastoridae | *Pristimantis carvalhoi* | LC | Peru | Russell et al. 2019 |
| Craugastoridae | *Pristimantis (Eleutherodactylus) caryophyllaceus* | NT | Panama | Morehouse et al. 2003; Lips et al. 2006 |
| Craugastoridae | *Pristimantis cerasinus (*was *Craugastor)* | LC | Costa Rica, Panama | Lips et al. 2006; Zumbado-Ulate et al. 2019 |
| Craugastoridae | *Pristimantis (Eleutherodactylus) chrysops* | EN | Colombia | Velásquez-E. et al. 2008 |
| Craugastoridae | *Pristimantis (Eleutherodactylus) cruentus* | LC | Panama | Berger et al. 1998; Lips et al. 2006 |
| Craugastoridae | *Pristimantis danae* | LC | Peru | Catenazzi et al. 2011, 2014; Warne et al. 2016 |
| Craugastoridae | *Pristimantis (Eleutherodactylus) elegans* | VU | Colombia | Ruiz and Rueda-Almonacid 2008; Flechas et al. 2017 |
| Craugastoridae | *Pristimantis (Eleutherodactylus) erythropleura* | LC | Colombia | Velásquez-E. et al. 2008; Flechas et al. 2017 |
| Craugastoridae | *Pristimantis (Eleutherodactylus) fenestratus* | LC | Peru | Warne et al. 2016; I. De la Riva, unpub. |
| Craugastoridae | *Pristimantis frater* | LC | Colombia | Acevedo et al. 2016; Flechas et al. 2017 |
| Craugastoridae | *Pristimantis (Eleutherodactylus) gracilis* | VU | Colombia | Velásquez-E. et al. 2008 |
| Craugastoridae | *Pristimantis gryllus* | NE | Colombia | Acevedo et al. 2016; Flechas et al. 2017 |
| Craugastoridae | *Pristimantis lanthanites* | LC | Ecuador | McCracken et al. 2009 |
| Craugastoridae | *Pristimantis lindae* | LC | Peru | Warne et al. 2016 |
| Craugastoridae | *Pristimantis lutitus* | EN | Colombia | Flechas et al. 2017 |
| Craugastoridae | *Pristimantis melanoproctus* | DD | Colombia | Acevedo et al. 2016; Flechas et al. 2017 |
| Craugastoridae | *Pristimantis miyatai* | LC | Colombia | Flechas et al. 2017 |
| Craugastoridae | *Pristimantis mondolfii* | DD | Colombia | Acevedo et al. 2016; Flechas et al. 2017 |
| Craugastoridae | *Pristimantis (Eleutherodactylus) museosus* | EN | Panama | Lips et al. 2006 |
| Craugastoridae | *Pristimantis ockendeni* | LC | Ecuador, Peru | McCracken et al. 2009; Russell et al. 2019 |
| Craugastoridae | *Pristimantis (Eleutherodactylus) palmeri* | LC | Colombia | Velásquez-E. et al. 2008; Flechas et al. 2017 |
| Craugastoridae | *Pristimantis paulodutrai* | LC | Brazil | Amorim et al. 2019 |
| Craugastoridae | *Pristimantis pharangobates (rhabdolaemus)* | LC | Bolivia, Peru | Catenazzi et al. 2011, 2014; Warne et al. 2016; Burrowes and de la Riva 2017 |
| Craugastoridae | *Pristimantis piceus* | LC | Colombia | Urbina and Galleano 2011 |
| Craugastoridae | *Pristimantis platydactylus* | LC | Bolivia, Peru | Catenazzi et al. 2011, 2014; Warne et al. 2016; Burrowes and de la Riva 2017 |
| Craugastoridae | *Pristimantis ramagii* | LC | Brazil | Valencia-Aguilar et al. 2015 |
| Craugastoridae | *Pristimantis reichlei* | NE | Peru | Warne et al. 2016; Russell et al. 2019 |
| Craugastoridae | *Pristimantis (Eleutherodactylus) ridens* | LC | Costa Rica, Panama | Lips et al. 2006; Whitfield et al. 2013; Zumbado-Ulate et al. 2019 |
| Craugastoridae | *Pristimantis salaputium* | LC | Peru | Catenazzi et al. 2011, 2014 |
| Craugastoridae | *Pristimantis savagei* | LC | Colombia | Flechas et al. 2017 |
| Craugastoridae | *Pristimantis shrevei* | EN | St. Vincent and the Grenadines | Sweeney 2016 |
| Craugastoridae | *Pristimantis (Eleutherodactylus) silverstonei* | NT | Colombia | Velásquez-E. et al. 2008 |
| Craugastoridae | *Pristimantis (Eleutherodactylus) thectopternus* | LC | Colombia | Velásquez-E. et al. 2008 |
| Craugastoridae | *Pristimantis toftae* | LC | Peru | Catenazzi et al. 2011, 2014; Warne et al. 2016 |
| Craugastoridae | *Pristimantis unistrigatus* | LC | Ecuador | Bresciano et al. 2015 |
| Craugastoridae | *Pristimantis uranobates* | LC | Colombia | Urbina and Galleano 2011 |
| Craugastoridae | *Pristimantis vinhai* | LC | Brazil | Valencia-Aguilar et al. 2015 |
| Craugastoridae | *Pristimantis (Eleutherodactylus) w-nigrum* | LC | Colombia | Velásquez-E. et al. 2008 |
| Craugastoridae | *Pristimantis waoranii* | DD | Ecuador | McCracken et al. 2009 |
| Craugastoridae | *Psychrophrynella adenopleura* | VU | Bolivia | Burrowes and de la Riva 2017 |
| Craugastoridae | *Psychrophrynella illimani* | CR | Bolivia | Burrowes and de la Riva 2017 |
| Craugastoridae | *Psychrophrynella quimsacruzis* | VU | Bolivia | Burrowes and de la Riva 2017 |
| Craugastoridae | *Psychrophrynella* sp. nov.^[[8]](#footnote-8)^ | NE | Peru | Catenazzi et al. 2011 |
| Craugastoridae | *Psychrophrynella usurpator* | NT | Peru | Catenazzi et al. 2011, 2014 |
| Craugastoridae | *Strabomantis (Craugastor) bufoniformis* | LC | Panama | Lips et al. 2006 |
| Craugastoridae | *Strabomantis (Eleutherodactylus) ruizi* | EN | Colombia | Velásquez-E. et al. 2008 |
| Craugastoridae | *Tachiramantis douglasi* | VU | Colombia | Acevedo et al. 2016; Flechas et al. 2017 |
| Craugastoridae | *Tachiramantis lentiginosus* | DD | Colombia | Acevedo et al. 2016; Flechas et al. 2017 |
| Craugastoridae | *Yunganastes fraudator* | LC | Bolivia | Burrowes and de la Riva 2017 |
| Craugastoridae | *Yunganastes pluvicanorus* | LC | Bolivia | Burrowes and de la Riva 2017 |
| **Cycloramphidae^[[9]](#footnote-9)^** | *Cycloramphus asper* | DD | Brazil | Rodriguez et al. 2014 |
| Cycloramphidae | *Cycloramphus boraceiensis* | LC | Brazil | Gründler et al. 2012; Rodriguez et al. 2014; Ruggeri et al. 2015 |
| Cycloramphidae | *Cycloramphus brasiliensis* | NT | Brazil | Rodriguez et al. 2014 |
| Cycloramphidae | *Cycloramphus eleutherodactylus* | DD | Brazil | Rodriguez et al. 2014; Ruggeri et al. 2018 Dis Aquat Org |
| Cycloramphidae | *Cycloramphus fuliginosus* | LC | Brazil | Rodriguez et al. 2014 |
| Cycloramphidae | *Cycloramphus izecksohni* | DD | Brazil | Rodriguez et al. 2014 |
| Cycloramphidae | *Cycloramphus ohausi* | DD | Brazil | Rodriguez et al. 2014 |
| Cycloramphidae | *Cycloramphus semipalmatus* | NT | Brazil | Rodriguez et al. 2014 |
| Cycloramphidae | *Cycloramphus stejnegeri* | DD | Brazil | Rodriguez et al. 2014 |
| Cycloramphidae | *Cycloramphus valae* | DD | Brazil | Rodriguez et al. 2014 |
| Cycloramphidae | *Thoropa miliaris* | LC | Brazil | Rodriguez et al. 2014; Ruggeri et al. 2018 Dis Aquat Org |
| Cycloramphidae | *Thoropa petropolitana* | VU | Brazil | Rodriguez et al. 2014 |
| Cycloramphidae | *Thoropa taophora* | NE | Brazil | Toledo et al. 2006a; Gründler et al. 2012; Rodriguez et al. 2014 |
| **Dendrobatidae** | *Adelphobates galactonotus* | LC | Brazil | Valencia-Aguilar et al. 2015 |
| Dendrobatidae | *Ameerega hahneli* | LC | Peru | Russell et al. 2019 |
| Dendrobatidae | *Ameerega trivittata* | LC | Peru | Russell et al. 2019 |
| Dendrobatidae | *Colostethus inguinalis* | LC | Panama | K Lips, unpub. |
| Dendrobatidae | *Colostethus panamansis (panamensis)* | LC | Panama | Lips et al. 2006 |
| Dendrobatidae | *Colostethus pratti* | LC | Panama | Lips et al. 2006 |
| Dendrobatidae | *Dendrobates auratus* | LC | Costa Rica, The Netherlands (cap.), Thailand (cap.), United States (cap.) | Pessier et al. 1999 (cap.); Saenz et al. 2009; Kik et al. 2012; Techangamsuwan et al. 2017 |
| Dendrobatidae | *Dendrobates leucomelas* | LC | Europe (cap.), United States (cap.) | Spitzen-van der Sluijs et al. 2011; A. Pessier and J. Mendelson, unpub. (cap.) |
| Dendrobatidae | *Dendrobates tinctorius (azureus)* | LC | Brazil, Czech Republic (cap.), Europe (cap.), French Guiana, The Netherlands (cap.), United States (cap.) | Pessier et al. 1999 (cap.); Spitzen-van der Sluijs et al. 2011 (cap.); De Paula et al. 2012 (cap.); Courtois et al. 2015; Havlíková et al. 2015 (cap.); A. Pessier and J. Mendelson, unpub. (cap.) |
| Dendrobatidae | *Epipedobates boulengeri* | LC | Colombia | Flechas et al. 2012, 2017 |
| Dendrobatidae | *Epipedobates tricolor* | VU | The Netherlands (cap.) | Spitzen-van der Sluijs et al. 2011 (cap.) |
| Dendrobatidae | *^[[10]](#footnote-10)^Hyloxalus abditaurantius* | LC | Colombia | Velásquez-E. et al. 2008 |
| Dendrobatidae | *Hyloxalus fascianigrus^[[11]](#footnote-11)^ (fascianiger)* | NT | Colombia | Velásquez-E. et al. 2008 |
| Dendrobatidae | *Hyloxalus ibague* | NE | Colombia | Flechas et al. 2017 |
| Dendrobatidae | *Hyloxalus lehmanni* | NT | Colombia | Velásquez-E. et al. 2008 |
| Dendrobatidae | *Hyloxalus shuar* | NT | Peru | Kosch et al. 2012 |
| Dendrobatidae | *Leucostethus fraterdanieli* | LC | Colombia | Velásquez-E. et al. 2008; Flechas et al. 2012, 2017 |
| Dendrobatidae | *Oophaga granulifera* | VU | Costa Rica | Zumbado-Ulate et al. 2019 |
| Dendrobatidae | *Oophaga histrionica* | CR | Colombia | Flechas et al. 2017 |
| Dendrobatidae | *Oophaga (Dendrobates) pumilio* | LC | Costa Rica, Europe (cap.), The Netherlands (cap.) | Puschendorf et al. 2006a, 2009; Saenz et al. 2009; Whitfield et al. 2012, 2013; Spitzen-van der Sluijs et al. 2011 (cap.); Zumbado-Ulate et al. 2019 |
| Dendrobatidae | *Phyllobates bicolor* | EN | The Netherlands (cap.) | Kik et al. 2012 |
| Dendrobatidae | *Phyllobates lugubris* | LC | Belgium (cap.), Costa Rica (wild), Germany (cap.) | Mutschmann et al. 2000 (cap.); Saenz et al. 2009 |
| Dendrobatidae | *Phyllobates terribilis* | EN | Europe (cap.) | Spitzen-van der Sluijs et al. 2011 (cap.) |
| Dendrobatidae | *Phyllobates vittatus* | EN | Germany (cap.), The Netherlands (cap.) | Mutschmann et al. 2000 (cap.); Kik et al. 2012 (cap.) |
| Dendrobatidae | *Ranitomeya amazonica* | DD | French Guiana | Courtois et al. 2015 |
| Dendrobatidae | *Ranitomeya benedicta* | VU | Japan (cap.) | Tamukai et al. 2014 (cap.) |
| Dendrobatidae | *Ranitomeya fantastica* | NT | Europe (cap.) | Spitzen-van der Sluijs et al. 2011 (cap.) |
| Dendrobatidae | *Ranitomeya imitator* | LC | Japan (cap.) | Tamukai et al. 2014 (cap.) |
| Dendrobatidae | *Ranitomeya reticulata* | LC | Japan (cap.) | Tamukai et al. 2014 (cap.) |
| Dendrobatidae | *Ranitomeya vanzolinii* | LC | Japan (cap.) | Tamukai et al. 2014 (cap.) |
| Dendrobatidae | *Silverstoneia (Colostethus) flotator* | LC | Panama | Lips et al. 2006 |
| Dendrobatidae | *Silverstoneia (Colostethus) nubicola* | LC | Panama | Lips et al. 2006; Velo-Antón et al. 2012 |
| **Dicroglossidae** | *Fejervarya cancrivora* | LC | Singapore | Gilbert et al. 2012 |
| Dicroglossidae | *Fejervarya limnocharis* | LC | Cambodia, China, Japan, Sri Lanka | Goka et al. 2009; Gaertner et al. 2011; Mendoza et al. 2011; Swei et al. 2011; Bai et al. 2012; Zhu et al. 2014 |
| Dicroglossidae | *Hoplobatrachus rugulosus* | LC | Cambodia (cap.) | Gaertner et al. 2011; Gilbert et al. 2012 |
| Dicroglossidae | *Limnonectes blythi* | NT | Singapore | Gilbert et al. 2012 |
| Dicroglossidae | *Limnonectes gyldenstolpei* | LC | Cambodia | Mendoza et al. 2011 |
| Dicroglossidae | *Limnonectes kuhlii* | LC | Malaysia | Swei et al. 2011 |
| Dicroglossidae | *Limnonectes laticeps* | LC | Malaysia | Savage et al. 2011 |
| Dicroglossidae | *Limnonectes macrocephalus* | NT | Philippines | Smith et al. 2019; A. Diesmos and R. Brown, unpub.^[[12]](#footnote-12)^ |
| Dicroglossidae | *Limnonectes microdiscus* | LC | Indonesia | Kusrini et al. 2008 |
| Dicroglossidae | *Limnonectes visayanus* | NT | Philippines | Smith et al. 2019 |
| Dicroglossidae | *Limnonectes woodworthi* | LC | Philippines | Swei et al. 2011; A. Diesmos and R. Brown, unpub.^6^ |
| Dicroglossidae | *Nanorana quadranus* | NT | China | Zhu et al. 2014 |
| Dicroglossidae | *Nanorana unculuanus* | EN | China | Zhu et al. 2014 |
| Dicroglossidae | *Nanorana yunnanensis* | EN | China | Zhu et al. 2014 |
| Dicroglossidae | *Occidozyga laevis* | LC | Philippines | Swei et al. 2011; Smith et al. 2019; A. Diesmos and R. Brown, unpub. ^6^ |
| Dicroglossidae | *Occidozyga lima* | LC | Cambodia, Japan (cap.) | Une et al. 2008 (cap.); Gaertner et al. 2011 |
| Dicroglossidae | *Quasipaa boulengeri* | EN | China | Zhu et al. 2014 |
| **Eleutherodactylidae^[[13]](#footnote-13)^** | *Diasporus^[[14]](#footnote-14)^ diastema* | LC | Costa Rica, Panama | Lips et al. 2006; Brem and Lips 2008; Saenz et al. 2009; Zumbado-Ulate et al. 2019 |
| Eleutherodactylidae | *Diasporus gularis* | LC | Colombia | Flechas et al. 2012, 2017 |
| Eleutherodactylidae | *Diasporus vocator* | LC | Panama | Lips et al. 2006 |
| Eleutherodactylidae | *Eleutherodactylus andrewsi* | EN | Jamaica | Holmes et al. 2012, 2014 |
| Eleutherodactylidae | *Eleutherodactylus antillensis* | LC | Puerto Rico | Burrowes et al. 2008; Barber 2012 |
| Eleutherodactylidae | *Eleutherodactylus atkinsi* | LC | Cuba | Cádiz et al. 2018 |
| Eleutherodactylidae | *Eleutherodactylus auriculatus* | LC | Cuba | Cádiz et al. 2018 |
| Eleutherodactylidae | *Eleutherodactylus brittoni* | LC | Puerto Rico | Burrowes et al. 2008 |
| Eleutherodactylidae | *Eleutherodactylus casparii* | EN | Cuba | Sabino-Pinto et al. 2017; Cádiz et al. 2018 |
| Eleutherodactylidae | *Eleutherodactylus coqui* | LC | Puerto Rico, United States | Beard and O'Neill 2005; Burrowes et al. 2008; Longo and Burrowes 2010; Longo et al. 2010; Longo and Zamudio 2017; Unpub. data from: P. Burrowes, A. Longo and R. Joglar; D.E. Green; A. Pessier and J. Mendelson (cap.) |
| Eleutherodactylidae | *Eleutherodactylus cundalli* | NT | Jamaica | Holmes et al. 2012, 2014 |
| Eleutherodactylidae | *Eleutherodactylus dimidiatus* | NT | Cuba | Cádiz et al. 2018 |
| Eleutherodactylidae | *Eleutherodactylus fuscus* | CR | Jamaica | Holmes et al. 2012, 2014 |
| Eleutherodactylidae | *Eleutherodactylus glamyrus* | EN | Cuba | Cádiz et al. 2018 |
| Eleutherodactylidae | *Eleutherodactylus glaucoreius* | NT | Jamaica | Holmes et al. 2012, 2014 |
| Eleutherodactylidae | *Eleutherodactylus gossei* | LC | Jamaica | Holmes et al. 2012, 2014 |
| Eleutherodactylidae | *Eleutherodactylus gundlachi* | EN | Cuba | Cádiz et al. 2018 |
| Eleutherodactylidae | *Eleutherodactylus ionthus* | EN | Cuba | Cádiz et al. 2018 |
| Eleutherodactylidae | *Eleutherodactylus johnstonei* | LC | Jamaica, Montserrat, St. Vincent and the Grenadines | Holmes et al. 2012, 2014; Sweeney et al. 2016; Hudson et al. 2019 |
| Eleutherodactylidae | *Eleutherodactylus karlschmidti* | CR | Puerto Rico | Burrowes et al. 2008; D.E. Green, unpub. |
| Eleutherodactylidae | *Eleutherodactylus locustus* | CR | Puerto Rico | Burrowes et al. 2008; P. Burrowes, A. Longo, & R. Joglar, unpub. |
| Eleutherodactylidae | *Eleutherodactylus luteolus* | EN | Jamaica | Holmes et al. 2012, 2014 |
| Eleutherodactylidae | *Eleutherodactylus martinicensis* | NT | Dominica | Hudson et al. 2019 |
| Eleutherodactylidae | *Eleutherodactylus melacara* | EN | Costa Rica | Lips et al. 2003 |
| Eleutherodactylidae | *Eleutherodactylus nubicola* | EN | Jamaica | Holmes et al. 2012, 2014 |
| Eleutherodactylidae | *Eleutherodactylus pantoni* | NT | Jamaica | Holmes et al. 2012, 2014 |
| Eleutherodactylidae | *Eleutherodactylus patriciae* | EN | Dominican Republic | Joglar et al. 2007 |
| Eleutherodactylidae | *Eleutherodactylus pentasyringos* | VU | Jamaica | Holmes et al. 2012, 2014 |
| Eleutherodactylidae | *Eleutherodactylus pituinus* | EN | Dominican Republic | Joglar et al. 2007 |
| Eleutherodactylidae | *Eleutherodactylus planirostris* | LC | Cuba, Jamaica, United States | Rizkalla 2010; Holmes et al. 2012, 2014; Goodman et al. 2019; Rivera et al. 2019 |
| Eleutherodactylidae | *Eleutherodactylus podi-noblei* | NE | Panama | K. Lips, unpub. |
| Eleutherodactylidae | *Eleutherodactylus portoricensis* | EN | Puerto Rico | Burrowes et al. 2008; Longo and Burrowes 2010; Longo et al. 2010 |
| Eleutherodactylidae | *Eleutherodactylus richmondi* | CR | Puerto Rico | Burrowes et al. 2008; P. Burrowes, A. Longo, & R. Joglar, unpub. |
| Eleutherodactylidae | *Eleutherodactylus riparius* | LC | Cuba | Cádiz et al. 2018 |
| Eleutherodactylidae | *Eleutherodactylus unicolor* | VU | Puerto Rico | Burrowes et al. 2008 |
| Eleutherodactylidae | *Eleutherodactylus wightmanae* | EN | Puerto Rico | Burrowes et al. 2008 |
| **Heleophrynidae** | *Hadromophryne (Heleophryne) natalensis* | LC | Lesotho, South Africa | Smith et al. 2007; C. Weldon, unpub. |
| Heleophrynidae | *Heleophryne hewitti* | EN | South Africa | Tarrant et al. 2013 |
| Heleophrynidae | *Heleophryne purcelli* | LC | South Africa | Weldon 2005 |
| Heleophrynidae | *Heleophryne regis* | LC | South Africa | Weldon 2005 |
| Heleophrynidae | *Heleophryne rosei* | CR | South Africa | Tarrant et al. 2013 |
| **Hemiphractidae** | *Cryptobatrachus boulengeri* | VU | Colombia | Flechas et al. 2017 |
| Hemiphractidae | *Flectonotus pygmaeus* | LC | Colombia | Acevedo et al. 2016; Flechas et al. 2017 |
| Hemiphractidae | *Fritziana fissilis* | LC | Brazil | Rodriguez et al. 2014 |
| Hemiphractidae | *Fritziana goeldii* | LC | Brazil | Rodriguez et al. 2014; Ruggeri et al. 2018 Dis Aquat Org |
| Hemiphractidae | *Fritziana ohausi* | LC | Brazil | Gründler et al. 2012; Rodriguez et al. 2014; Ruggeri et al. 2018 Dis Aquat Org |
| Hemiphractidae | *Gastrotheca (Hyla) antoniiochoai* | DD | Peru | Catenazzi et al. 2011 |
| Hemiphractidae | *Gastrotheca cornuta* | EN | Panama | Lips et al. 2006 |
| Hemiphractidae | *Gastrotheca dendronastes* | VU | Colombia | Velásquez-E. et al. 2008 |
| Hemiphractidae | *Gastrotheca excubitor* | VU | Peru | Catenazzi et al. 2011; Kosch et al. 2012; Burkart et al. 2017 |
| Hemiphractidae | *Gastrotheca helenae* | DD | Colombia | Acevedo et al. 2016; Flechas et al. 2017 |
| Hemiphractidae | *Gastrotheca marsupiata* | LC | Bolivia | Burrowes and de la Riva 2017 |
| Hemiphractidae | *Gastrotheca megacephala* | NE | Brazil | Amorim et al. 2019 |
| Hemiphractidae | *Gastrotheca nebulanastes* | EN | Peru | Burkart et al. 2017 |
| Hemiphractidae | *Gastrotheca nicefori* | LC | Venezuela | Sánchez et al. 2008 |
| Hemiphractidae | *Gastrotheca ochoai* | DD | Peru | Catenazzi et al. 2011 |
| Hemiphractidae | *Gastrotheca piperata* | LC | Bolivia | Burrowes and de la Riva 2017 |
| Hemiphractidae | *Gastrotheca plumbea* | VU | Ecuador | Bresciano et al. 2015 |
| Hemiphractidae | *Gastrotheca pseustes* | EN | Ecuador | Ron and Merino-Viteri 2000 |
| Hemiphractidae | *Gastrotheca riobambae* | EN | Ecuador | Bresciano et al. 2015 |
| Hemiphractidae | *Gastrotheca splendens* | EN | Bolivia | Burrowes and de la Riva 2017 |
| **Hylidae^[[15]](#footnote-15)^** | *Acris (crepitans) blanchardi* (listed as Threatened in several states, Endangered in Canada) | NE | United States | Pessier et al. 1999; Rothermel et al. 2008; Steiner and Lehtinen 2008; Zippel and Tabaka 2008; Gaertner et al. 2009 (Herp Rev), 2012; Saenz et al. 2010; Lanoo et al. 2011; McTaggart et al. 2014; Phillips et al. 2014; Beyer et al. 2015; Talley et al. 2015; Watters et al. 2016, 2018, 2019; Marhanka et al. 2017; Suriyamongkol et al. 2019; J. Krebs, unpub. |
| Hylidae | *Acris crepitans* | LC | United States | Pessier et al. 1999; Rothermel et al. 2008; Saenz et al. 2010; Lannoo et al. 2011; Brannelly et al. 2012, 2018 BMC Ecol; Goodman and Ararso 2012; Brown and Kerby 2013; Muellman and Montgomery 2013; Hanlon et al. 2014 (Arkansas), 2014 (Tennessee); Petersen et al. 2016; Sonn et al. 2019; Unpub. data from: J. Krebs; J. Ware and K. Duncan; A. Pessier and J. Mendelson (cap.) |
| Hylidae | *Acris gryllus* | LC | United States | Rizkalla 2009, 2010; Brannelly et al. 2012; Hill and Levy 2014; Petersen et al. 2016; Chiari et al. 2017 |
| Hylidae | *Aparasphenodon brunoi* | LC | Brazil | Rodriguez et al. 2014 |
| Hylidae | *Aplastodiscus albosignatus* | LC | Brazil | Rodriguez et al. 2014; Coutinho et al. 2015; Navarro-Lozano et al. 2018 |
| Hylidae | *Aplastodiscus arildae* | LC | Brazil | Coutinho et al. 2015 |
| Hylidae | *Aplastodiscus callipygius* | LC | Brazil | Toledo et al. 2006a; Gründler et al. 2012 |
| Hylidae | *Aplastodiscus ibirapitanga* | LC | Brazil | Amorim et al. 2019 |
| Hylidae | *Aplastodiscus leucopygius* | LC | Brazil | Toledo et al. 2006a; Gründler et al. 2012; Coutinho et al. 2015 |
| Hylidae | *Aplastodiscus perviridis* | LC | Brazil | Gründler et al. 2012; Rodriguez et al. 2014 |
| Hylidae | *Aplastodiscus sibilatus* | DD | Brazil | Valencia-Aguilar et al. 2016; Amorim et al. 2019 |
| Hylidae | *Boana albomarginata (Hypsiboas albomarginatus)* | LC | Brazil | Rodriguez et al. 2014; Valencia-Aguilar et al. 2016 |
| Hylidae | *Boana albonigra* | NT | Bolivia | Burrowes and de la Riva 2017 |
| Hylidae | *Boana albopunctata (Hypsiboas albopunctatus)* | LC | Brazil | Toledo et al. 2006a; Jenkinson et al. 2016; Navarro-Lozano et al. 2018 |
| Hylidae | *Boana (Hypsiboas) balzani* | LC | Bolivia, Peru | Catenazzi et al. 2011; Burrowes and de la Riva 2017; Burrowes et al. 2020 |
| Hylidae | *Boana (Hypsiboas) bischoffi* | LC | Brazil | Rodriguez et al. 2014; Coutinho et al. 2015 |
| Hylidae | *Boana calcarata* | LC | Peru | Russell et al. 2019 |
| Hylidae | *Boana callipleura* | LC | Bolivia | Lindquist et al. 2016; Burrowes and de la Riva 2017 |
| Hylidae | *Boana cinerascens* | LC | Peru | Russell et al. 2019 |
| Hylidae | *Boana (Hypsiboas) cordobae* | DD | Argentina | Gutierrez et al. 2010; Lescano et al. 2013 |
| Hylidae | *Boana (Hypsiboas) crepitans* | LC | Brazil, Colombia, Venezuela | Sánchez et al. 2008; Valencia-Aguilar et al. 2015; Acevedo et al. 2016; Flecha et al. 2017 |
| Hylidae | *Boana exastis* | DD | Brazil | Valencia-Agilar et al. 2015 |
| Hylidae | *Boana (Hypsiboas) faber* | LC | Brazil | Gründler et al. 2012; Vieira et al. 2012; Rodriguez et al. 2014; Coutinho et al. 2015; Jenkinson et al. 2016; Navarro-Lozano et al. 2018; Ruggeri et al. 2018 Dis Aquat Org |
| Hylidae | *Boana (Hypsiboas) freicanecae* | DD | Brazil | Carnaval et al. 2006; Valencia-Aguilar et al. 2016 |
| Hylidae | *Boana geographica (Hypsiboas geographicus, Hyla geographica)* | LC | Peru | Russell et al. 2019 |
| Hylidae | *Boana gladiator* | VU | Peru | Warne et al. 2016 |
| Hylidae | *Boana lanciformis* | LC | Colombia, Peru | Acevedo et al. 2016; Flechas et al. 2017; Russell et al. 2019 |
| Hylidae | *Boana latistriata (Hypsiboas latistriatus)* | DD | Brazil | Carnaval et al. 2006; Gründler et al. 2012 |
| Hylidae | *Boana maculateralis* | NE | Peru | Russell et al. 2019 |
| Hylidae | *Boana marianitae* | LC | Bolivia | Burrowes and de la Riva 2017 |
| Hylidae | *Boana (Hypsiboas) melanopleura* | DD | Peru | Kosch et al. 2012 |
| Hylidae | *Boana (Hypsiboas) pardalis* | LC | Brazil | Gründler et al. 2012; Rodriguez et al. 2014 |
| Hylidae | *Boana polytaenia (Hypsiboas polytaenius)* | LC | Brazil | Rodriguez et al. 2014; Couthinho et al. 2015; Ruggeri et al. 2018 Dis Aquat Org |
| Hylidae | *Boana prasina (Hypsiboas prasinus)* | LC | Brazil | Gründler et al. 2012; Rodriguez et al. 2014 |
| Hylidae | *Boana pulchella (Hypsiboas pulchellus)* | LC | Argentina, Brazil, Uruguay | Borteiro et al. 2009; Ghirardi 2011; Rodriguez et al. 2014; Agostini and Burrowes 2015 |
| Hylidae | *Boana punctata* | LC | Colombia, Peru | Flechas et al. 2017; Russell et al. 2019 |
| Hylidae | *Boana riojana* | DD | Bolivia | Lindquist et al. 2016; Burrowes and de la Riva 2017 |
| Hylidae | *Boana rufitela* | LC | Costa Rica | Zumbado-Ulate et al. 2019 |
| Hylidae | *Boana semilineata (Hypsiboas semilineatus)* | LC | Brazil | Toledo et al. 2006a; Coutiho et al. 2015 |
| Hylidae | *Boana (Hypsiboas) tetete* | VU | Peru | Russell et al. 2019 |
| Hylidae | *Bokermannohyla astartea* | LC | Brazil | Coutinho et al. 2015 |
| Hylidae | *Bokermannohyla capra* | NE | Brazil | Amorim et al. 2019 |
| Hylidae | *Bokermannohyla carvalhoi* | LC | Brazil | Ruggeri et al. 2018 Dis Aquat Org |
| Hylidae | *Bokermannohyla circumdata* | LC | Brazil | Toledo et al. 2006a; Gründler et al. 2012; Rodriguez et al. 2014; Coutinho et al. 2015; Ruggeri et al. 2018 Dis Aquat Org |
| Hylidae | *Bokermannohyla gouveai* | DD | Brazil | Carnaval et al. 2006 |
| Hylidae | *Bokermannohyla hylax* | LC | Brazil | Toledo et al. 2006a; Gründler et al. 2012; Coutinho et al. 2015; Jenkinson et al. 2016 |
| Hylidae | *Bokermannohyla luctuosa* | LC | Brazil | Gründler et al. 2012; Rodriguez et al. 2014 |
| Hylidae | *Bromeliohyla bromeliacia* | LC | Honduras | Blooi et al. 2017 |
| Hylidae | *Charadrahyla juanitae* | NT | Mexico | Köhler et al. 2016 |
| Hylidae | *Charadrahyla nephila* | EN | Mexico | Lips et al. 2004 |
| Hylidae | *Corythomantis greeningi* | LC | Brazil | Amorim et al. 2019 |
| Hylidae | *Dendropsophus anceps* | LC | Brazil | Rodriguez et al. 2014 |
| Hylidae | *Dendropsophus brevifrons* | LC | Peru | Russell et al. 2019 |
| Hylidae | *Dendropsophus colombianus* | LC | Colombia | Flechas et al. 2017 |
| Hylidae | *Dendropsophus ebraccatus (Hyla ebraccata)* | LC | Belize, Costa Rica, Panama | Picco and Collins 2007; Kaiser and Pollinger 2012; Rebollar et al. 2014; Zumbado-Ulate et al. 2019 |
| Hylidae | *Dendropsophus leucophyllatus* | LC | Colombia, Peru | Flechas et al. 2017; Russell et al. 2019 |
| Hylidae | *Dendropsophus mathiassoni* | LC | Colombia | Vásquez-Ochoa et al. 2012 |
| Hylidae | *Dendropsophus meridensis* | EN | Venezuela | Sánchez et al. 2008 |
| Hylidae | *Dendropsophus microcephalus (Hyla microcephala)* | LC | Colombia, Honduras, Nicaragua, Panama | Lips et al. 2006; Brem and Lips 2008; Garcia-Roa et al. 2014; Gutsche et al. 2015; Acevedo et al. 2016; Flechas et al. 2017 |
| Hylidae | *Dendropsophus microps* | LC | Brazil | Gründler et al. 2012; Rodriguez et al. 2014 |
| Hylidae | *Dendropsophus minutus* | LC | Brazil, French Guiana, Peru | Gründler et al. 2012; Rodriguez et al. 2014; Courtois et al. 2015; Valencia-Aguilar et al. 2015; Jenkinson et al. 2016; Ruggeri et al. 2018 Dis Aquat Org; Russell et al. 2019 |
| Hylidae | *Dendropsophus molitor* | LC | Colombia | Vásquez-Ochoa et al. 2012; Acevedo et al. 2016; Flechas et al. 2017 |
| Hylidae | *Dendropsophus parviceps* | LC | Peru | Russell et al. 2019; I. De la Riva, unpub. |
| Hylidae | *Dendropsophus pelidna* | LC | Colombia | Acevedo et al. 2016; Flechas et al. 2017 |
| Hylidae | *Dendropsophus rhodopeplus* | LC | Peru | Russell et al. 2019 |
| Hylidae | *Dendropsophus riveroi* | LC | Peru | Russell et al. 2019 |
| Hylidae | *Dendropsophus seniculus* | LC | Brazil | Rodriguez et al. 2014 |
| Hylidae | *Dendropsophus subocularis* | LC | Colombia | Flechas et al. 2017 |
| Hylidae | *Dendropsophus triangulum* | LC | Peru | Russell et al. 2019 |
| Hylidae | *Dryophytes (Hyla) arenicolor* | LC | United States | Bradley et al. 2002; Speare and Berger 2005^[[16]](#footnote-16)^; Arizona Game & Fish Dept, unpub. |
| Hylidae | *Dryophytes bocourti* | CR | Guatemala | Mendelson et al. 2014 |
| Hylidae | *Dryophytes (Hyla) chrysoscelis* | LC | United States | Rothermel et al. 2008, 2016; Davidson and Chambers 2011; Lannoo et al. 2011; Tupper et al. 2017 |
| Hylidae | *Dryophytes (Hyla) chrysoscelis/versicolor* or *versicolor/chrysoscelis* | LC | United States | Beyer et al. 2015; Talley et al. 2015; Watters et al. 2016, 2018, 2019; Marhanka et al. 2017; Talbott et al. 2018 |
| Hylidae | *Dryophytes cinereus (Hyla cinereal)* | LC | United States | Longcore et al. 2007; Watters et al. 2016; Chiari et al. 2017; Marhanka et al. 2017; Tupper et al. 2017; Rivera et al. 2019; A. Pessier and J. Mendelson, unpub. (cap.) |
| Hylidae | *Dryophytes euphorbiaceus*  *(Hyla euphorbiacea)* | NT | Mexico | Frías-Alvarez et al. 2008 |
| Hylidae | *Dryophytes eximius (Hyla eximia)* | LC | Mexico | Frías-Alvarez et al. 2008; Velo-Antón et al. 2012; Galindo-Bustos et al. 2014 |
| Hylidae | *Dryophytes gratiosus* | LC | United States | Hill and Levy 2014; A. Pessier and J. Mendelson, unpub. (cap.) |
| Hylidae | *Dryophytes immaculatus* | LC | China | Zhu et al. 2014 |
| Hylidae | *Dryophytes japonicus (Hyla japonica)* | LC | Japan, South Korea | Goka et al. 2009; Yang et al. 2009; Swei et al. 2011; Bataille et al. 2013; An and Waldman 2016; Borzée et al. 2017; Rios-Sotelo et al. 2018; S. Okada, unpub. |
| Hylidae | *Dryophytes (Hyla) squirellus* | LC | United States | Horner et al. 2017; Rivera et al. 2019 |
| Hylidae | *Dryophytes suweonensis* | EN | South Korea | Borzée et al. 2017; |
| Hylidae | *Dryophytes (Hyla) versicolor* | LC | Canada, United States | Ouellet et al. 2005; Rothermel et al. 2008; Muelleman and Montgomery 2013; Richards-Hrdlicka et al. 2013; Lenker et al. 2014; Villamizar-Gómez et al. 2016; Watters et al. 2016; Marhanka et al. 2017; Olori et al 2018; A. Pessier and J. Mendelson, unpub. (cap.) |
| Hylidae | *Dryophytes (Hyla) wrightorum* | LC | United States | Sredl et al. 2002 |
| Hylidae | *Duellmanohyla ignicolor* | NT | Mexico | Lips et al. 2004 |
| Hylidae | *Duellmanohyla rufioculis* | LC | Costa Rica | Picco and Collins 2007; Zumbado-Ulate et al. 2019 |
| Hylidae | *Duellmanohyla soralia* | EN | Honduras | Kolby et al. 2010, 2015 Honduras; Blooi et al. 2017 |
| Hylidae | *Duellmanohyla uranochroa* | EN | Costa Rica | Puschendorf et al. 2006a, 2009 |
| Hylidae | *Exerodonta melanomma* | VU | Mexico | Lips et al. 2004; Frías-Alvarez et al. 2008 |
| Hylidae | *Hyla annectans* | LC | China | Bai et al. 2012; Zhu et al. 2014 |
| Hylidae | *Hyla arborea* | LC | Germany, Japan (cap.), Montenegro, The Netherlands, Spain | Ohst et al. 2013; Spitzen-van der Sluijs et al. 2014; Tamukai et al. 2014 (cap.); Vojar et al. 2017; S. Walker, unpub. |
| Hylidae | *Hyla chinensis* | LC | China | Bai et al. 2012 |
| Hylidae | *Hyla meridionalis* | LC | Morocco, Spain | El Mouden et al. 2011; Hidalgo-Vila et al. 2012; El Cadi et al. 2019; S. Walker, unpub. |
| Hylidae | *Hyla molleri* | NE | Portugal | Rosa et al. 2017 |
| Hylidae | *Hyla orientalis* | NE | Turkey | Erismis et al. 2014 |
| Hylidae | *Hyla sarda* | LC | Italy | J. Bielby, TWJ Garner, & S. Bovero, unpub. |
| Hylidae | *Hyloscirtus alytolylax* | NT | Colombia | Velásquez-E. et al. 2008 |
| Hylidae | *Hyloscirtus armatus* | LC | Bolivia, Peru | Catenazzi et al. 2011; Burrowes and de la Riva 2017 |
| Hylidae | *Hyloscirtus bogotensis* | NT | Colombia | Ruiz and Rueda-Almonacid 2008 |
| Hylidae | *Hyloscirtus colymba* | CR | Panama | Lips et al. 2006 |
| Hylidae | *Hyloscirtus jahni* | NT | Venezuela | Sánchez et al. 2008 |
| Hylidae | *Hyloscirtus larinopygion* | NT | Colombia | Urbina and Galleano 2011 |
| Hylidae | *Hyloscirtus palmeri* | LC | Costa Rica, Panama | Lips et al. 2006; Brem and Lips 2008; Zumbado-Ulate et al. 2019 |
| Hylidae | *Hyloscirtus* cf. *phyllognathus* | LC | Peru | Kosch et al. 2012 |
| Hylidae | *Hyloscirtus platydactylus* | VU | Colombia, Venezuela | Sánchez et al. 2008; Acevedo et al. 2016 |
| Hylidae | *Hyloscirtus psarolaimus (Hyla psarolaima)* | EN | Ecuador | Ron and Merino-Viteri 2000 |
| Hylidae | *Isthmohyla picadoi* | NT | Panama | Lindquist et al. 2011 |
| Hylidae | *Isthmohyla pseudopuma* | LC | Costa Rica | Puschendorf et al. 2006a, 2009; Picco and Collins 2007; Cheng et al. 2011 |
| Hylidae | *Isthmohyla rivularis* | CR | Costa Rica | Cheng et al. 2011 |
| Hylidae | *Itapotihyla langsdorfii* | LC | Brazil | Rodriguez et al. 2014 |
| Hylidae | *Ololygon albicans* | LC | Brazil | Ruggeri et al. 2018 Dis Aquat Org |
| Hylidae | *Ololygon flavoguttata* | LC | Brazil | Ruggeri et al. 2018 Dis Aquat Org |
| Hylidae | *Ololygon v-signata* | LC | Brazil | Ruggeri et al. 2018 Dis Aquat Org |
| Hylidae | *Osteocephalus buckleyi* | LC | Peru | Kosch et al. 2012; Russell et al. 2019 |
| Hylidae | *Osteocephalus castaneicola* | LC | Peru | Russell et al. 2019 |
| Hylidae | *Osteocephalus leprieurii* | LC | Peru | Russell et al. 2019 |
| Hylidae | *Osteocephalus planiceps* | LC | Peru | Russell et al. 2019 |
| Hylidae | *Osteocephalus taurinus* | LC | Peru | Russell et al. 2019 |
| Hylidae | *Osteocephalus yasuni* | LC | Peru | Russell et al. 2019 |
| Hylidae | *Osteopilus ocellatus* | LC | Jamaica | Holmes et al. 2012, 2014 |
| Hylidae | *Osteopilus septentrionalis* | LC | Cuba, Czech Republic (cap.), United States | Rizkalla 2010; Havlíková et al. 2015; Sabino-Pinto et al. 2017; A. Pessier and J. Mendelson, unpub. (cap.) |
| Hylidae | *Osteopilus vastus* | EN | Dominican Republic | Joglar et al. 2007 |
| Hylidae | *Phyllodytes acuminatus* | LC | Brazil | Valencia-Aguilar et al. 2015 |
| Hylidae | *Phyllodytes edelmoi* | DD | Brazil | Valencia-Aguilar et al. 2015; Ruano-Fajardo et al. 2016 |
| Hylidae | *Phyllodytes gyrinaethes* | DD | Brazil | Valencia-Aguilar et al. 2015 |
| Hylidae | *Phyllodytes tuberculosus* | DD | Brazil | Valencia-Aguilar et al. 2015 |
| Hylidae | *Plectrohyla arborescandens* | EN | Mexico | Luría-Manzano et al. 2011 |
| Hylidae | *Plectrohyla dasypus* | CR | Honduras | Kolby and Padgett-Flohr 2009; Kolby et al. 2010, 2015 Honduras; Blooi et al. 2017 |
| Hylidae | *Plectrohyla exquisita* | CR | Honduras | Kolby et al. 2015 Honduras; Blooi et al. 2017 |
| Hylidae | *Plectrohyla matudai* | LC | Mexico | Lips et al. 2004 |
| Hylidae | *Plectrohyla sagorum* | VU | Mexico | Lips et al. 2004 |
| Hylidae | *Pseudacris cadaverina* | LC | United States | Lannoo et al. 2011; Adams et al. 2017 Ecol Evol; Peralta-García et al. 2018; A. Pessier and J. Mendelson, unpub. (cap.) |
| Hylidae | *Pseudacris clarkii* | LC | United States | Watters et al. 2016, 2019 |
| Hylidae | *Pseudacris crucifer* | LC | United States | Rothermel et al. 2008; Saenz et al. 2010; Lannoo et al. 2011; Goodman and Ararso 2012; Krynak et al. 2012; Muelleman and Montgomery 2013; Hill and Levy 2014; Hughey et al. 2014; Lenker et al. 2014; Phillips et al. 2014; Windstam and Olori 2014; Beyer et al. 2015; Saenz et al. 2015; Rothermel et al. 2016; Watters et al. 2016, 2018; Chiari et al. 2017; Tupper et al. 2017; Brannelly et al. 2018 BMC Ecol; Olori et al. 2018; D.E. Green, unpub.; A. Pessier and J. Mendelson, unpub. (cap.) |
| Hylidae | *Pseudacris fouquettei* | LC | United States | Rothermel et al. 2008; Saenz et al. 2010; Watters et al. 2016, 2019; Marhanka et al. 2017; Brannelly et al. 2012, 2018 BMC Ecol |
| Hylidae | *Pseudacris hypochondriaca* (and *P. h. curta*) | LC | Mexico, United States | Lannoo et al. 2011; Luja et al. 2012 ; Reeder et al. 2012 |
| Hylidae | *Pseudacris maculata* | LC | Canada, United States | Muths et al. 2008; Rittmann et al. 2003; Rogers and Banulis 2004; Rodriguez et al. 2009; Schock et al. 2009; Wixson and Rogers 2009; Hyman and Collins 2012; Stevens et al. 2012; Brown and Kerby 2013; Phillips et al. 2014; Beyer et al. 2015; Araos et al. 2017; Reeves et al. 2017; Stutz et al. 2017; Watters et al. 2018; Unpub. data from: D.E. Green; B. Maxell; J. Nachtmann |
| Hylidae | *Pseudacris maculata/triseriata* | LC | United States | Talbott et al. 2018 |
| Hylidae | *Pseudacris ocularis* | LC | United States | Rizkalla 2010 |
| Hylidae | *Pseudacris ornata* | LC | United States | Hill and Levy 2014; Love et al. 2016; Petersen et al. 2016; Horner et al. 2017 |
| Hylidae | *Pseudacris regilla* | LC | United States | Pearl et al. 2007; Lowe 2009; Gaulke et al. 2011; Piovia-Scott et al. 2011; Richardson et al. 2014; Battaglin et al. 2016; Petersen et al. 2016; Ecoclub Amphibian Group et al. 2016; Adams et al. 2017 Ecol Evol; 2017 Ecosphere; Jaeger et al. 2017; Peralta-García et al. 2018; Unpub. data from: D.E. Green; V. Hemingway; P. Johnson; G. Padgett-Flohr; S. Wagner and J. Johnson |
| Hylidae | *Pseudacris sierra* | NE | United States | Fellers et al. 2011; Petersen et al. 2016 |
| Hylidae | *Pseudacris streckeri* | LC | United States | Watters et al. 2016; Marhanka et al. 2017 |
| Hylidae | *Pseudacris triseriata* | LC | United States | Sredl et al. 2002; Green and Muths 2005; Ouellet et al. 2005; Young et al. 2007; Carey and Livo 2009; Peterson and McKenzie 2014; Battaglin et al. 2016; D.E. Green, unpub. |
| Hylidae | *Pseudis minuta* | LC | Argentina | Agostini and Burrowes 2015 |
| Hylidae | *Pseudis paradoxa* | LC | Venezuela | Sánchez et al. 2008 |
| Hylidae | *Ptychohyla hypomykter* | CR | Guatemala, Honduras | Mendelson et al. 2004; Kolby et al. 2010, 2015 Honduras; Blooi et al. 2017 |
| Hylidae | *Ptychohyla legleri* | EN | Costa Rica | Whitfield et al. 2017 |
| Hylidae | *Ptychohyla leonhardschultzei* | EN | Mexico | Lips et al. 2004; Köhler et al. 2016 |
| Hylidae | *Ptychohyla zophodes* | VU | Mexico | Lips et al. 20014 |
| Hylidae | *Quilticohyla (Ptychohyla) erythromma* | EN | Mexico | Lips et al. 2004 |
| Hylidae | *Rheohyla miotympanum* | NT | Mexico | Murrieta-Galindo et al. 2014 |
| Hylidae | *Sarcohyla bistincta* | LC | Mexico | Köhler et al. 2019 Mexico |
| Hylidae | *Sarcohyla (Hyla) cyclada* | VU | Mexico | Lips et al. 20014 |
| Hylidae | *Sarcohyla pentheter* | EN | Mexico | Köhler et al. 2016 |
| Hylidae | *Sarcohyla (Hyla) sabrina* | CR | Mexico | Lips et al. 20014 |
| Hylidae | *Scarthyla goinorum* | LC | Peru | Russell et al. 2019 |
| Hylidae | *Scarthyla vigilans* | LC | Venezuela | Sánchez et al. 2008 |
| Hylidae | *Scinax albicans* | LC | Brazil | Toledo et al. 2006a S Amer J Herp |
| Hylidae | *Scinax alter* | LC | Brazil | Coutinho et al. 2015 |
| Hylidae | *Scinax ariadne* | DD | Brazil | Rodriguez et al. 2014 |
| Hylidae | *Scinax boulengeri* | LC | Costa Rica | Zumbado-Ulate et al. 2019 |
| Hylidae | *Scinax brieni* | LC | Brazil | Coutinho et al. 2015 |
| Hylidae | *Scinax crospedospilus* | LC | Brazil | Coutinho et al. 2015 |
| Hylidae | *Scinax elaeochroa (elaeochrous)* | LC | Costa Rica | Whitfield et al. 2013; Zumbado-Ulate et al. 2019 |
| Hylidae | *Scinax eurydice* | LC | Brazil | Valencia-Aguilar et al. 2015 ; Amorim et al. 2019 |
| Hylidae | *Scinax funereus (*sp. 1*)* | LC | Peru | Russell et al. 2019 |
| Hylidae | *Scinax fuscovarius* | LC | Brazil | Rodriguez et al. 2014; Coutinho et al. 2015; Jenkinson et al. 2016 |
| Hylidae | *Scinax garbei* | LC | Peru | Kosch et al. 2012 |
| Hylidae | *Scinax granulatus* | LC | Brazil | Rodriguez et al. 2014 |
| Hylidae | *Scinax hayii* | LC | Brazil | Gründler et al. 2012; Rodriguez et al. 2014; Coutinho et al. 2015; Jenkinson et al. 2016; Navarro-Lozano et al. 2018; Ruggeri et al. 2018 Dis Aquat Org |
| Hylidae | *Scinax machadoi* | LC | Brazil | Rodriguez et al. 2014 |
| Hylidae | *Scinax manriquei* | LC | Colombia | Acevedo et al. 2016; Flechas et al. 2017 |
| Hylidae | *Scinax nebulosus* | LC | Brazil | Valencia-Aguilar et al. 2015 |
| Hylidae | *Scinax pachycrus* | LC | Brazil | Valencia-Aguilar et al. 2015 |
| Hylidae | *Scinax perpusillus* | LC | Brazil | Rodriguez et al. 2014 |
| Hylidae | *Scinax ranki* | DD | Brazil | Rodriguez et al. 2014 |
| Hylidae | *Scinax rostratus* | LC | Colombia | Acevedo et al. 2016 |
| Hylidae | *Scinax ruber* | LC | Colombia | Flechas et al. 2017 |
| Hylidae | *Scinax squalirostris* | LC | Uruguay | Borteiro et al. 2009 |
| Hylidae | *Scinax trapicheiroi* | NT | Brazil | Rodriguez et al. 2014 |
| Hylidae | *Scinax wandae* | LC | Colombia | Flechas et al. 2017 |
| Hylidae | *Smilisca baudinii* | LC | Honduras, Nicaragua | García-Roa et al. 2014; Gutsche et al. 2015 |
| Hylidae | *Smilisca cyanosticta* | NT | Belize | Kaiser and Pollinger 2012 |
| Hylidae | *Smilisca phaeota* | LC | Costa Rica, Nicaragua, Panama | Morehouse et al. 2003; Lips et al. 2006; Brem and Lips 2008; García-Roa et al. 2014; Zumbado-Ulate et al. 2019 |
| Hylidae | *Smilisca sila* | LC | Panama | Hertz et al. 2018 |
| Hylidae | *Smilisca sordida* | LC | Costa Rica | Goldberg et al. 2009; Saenz et al. 2009; Zumbado-Ulate et al. 2019 |
| Hylidae | *Sphaenorhynchus carneus* | LC | Peru | Russell et al. 2019 |
| Hylidae | *Sphaenorhynchus lacteus* | LC | Peru | Russell et al. 2019 |
| Hylidae | *Tlalocohyla loquax* | LC | Costa Rica | Zumbado-Ulate et al. 2019 |
| Hylidae | *Trachycephalus mesophaeus* | LC | Brazil | Rodriguez et al. 2014, Coutinho et al. 2015 |
| Hylidae | *Trachycephalus resinifictrix* | LC | Japan (cap.) | Tamukai et al. 2014 (cap.) |
| Hylidae | *Trachycephalus typhonius venulosa* | LC | Peru | Russell et al. 2019 |
| Hylidae | *Triprion spinosus* | LC | Costa Rica | Zumbado-Ulate et al. 2019 |
| **Hylodidae** | *Crossodactylus bokermanni* | DD | Brazil | Rodriguez et al. 2014 |
| Hylodidae | *Crossodactylus dantei* | DD | Brazil | Valencia-Aguilar 2015, 2016 |
| Hylodidae | *Crossodactylus caramaschii* | LC | Brazil | Rodriguez et al. 2014; Navarro-Lozano et al. 2018 |
| Hylodidae | *Crossodactylus gaudichaudii* | LC | Brazil | Rodriguez et al. 2014 |
| Hylodidae | *Crossodactylus schmidti* | NT | Brazil | Jenkinson et al. 2016 |
| Hylodidae | *Hylodes amnicola* | DD | Brazil | Rodriguez et al. 2014 |
| Hylodidae | *Hylodes asper* | LC | Brazil | Gründler et al. 2012; Rodriguez et al. 2014; Coutinho et al. 2015; Ruggeri et al. 2015 |
| Hylodidae | *Hylodes cardosoi* | LC | Brazil | Vieira et al. 2012; Jenkinson et al. 2016 |
| Hylodidae | *Hylodes dactylocinus* | DD | Brazil | Toledo et al. 2006a |
| Hylodidae | *Hylodes glaber* | DD | Brazil | Rodriguez et al. 2014 |
| Hylodidae | *Hylodes heyeri* | DD | Brazil | Rodriguez et al. 2014 |
| Hylodidae | *Hylodes japi* | NE | Brazil | Jenkinson et al. 2016 |
| Hylodidae | *Hylodes lateristrigatus* | LC | Brazil | Rodriguez et al. 2014 |
| Hylodidae | *Hylodes magalhaesi* | DD | Brazil | Carnaval et al. 2005; Toledo et al. 2006b; Gründler et al. 2012 |
| Hylodidae | *Hylodes meridionalis* | LC | Brazil | Toledo et al. 2006a; Rodriguez et al. 2014; Jenkinson et al. 2016 |
| Hylodidae | *Hylodes nasus* | LC | Brazil | Rodriguez et al. 2014 |
| Hylodidae | *Hylodes ornatus* | LC | Brazil | Rodriguez et al. 2014 |
| Hylodidae | *Hylodes perere* | NE | Brazil | Rodriguez et al. 2014 |
| Hylodidae | *Hylodes perplicatus* | LC | Brazil | Toledo et al. 2006a; Rodriguez et al. 2014 |
| Hylodidae | *Hylodes phyllodes* | LC | Brazil | Toledo et al. 2006a; Gründler et al. 2012; Coutinho et al. 2015; Ruggeri et al. 2015; Jenkinson et al. 2016; Lambertini et al. 2016 |
| Hylodidae | *Hylodes* sp. *(*aff. *sazimai)* | DD | Brazil | Toledo et al. 2006a |
| Hylodidae | *Megaelosia* cf*. boticariana* | DD | Brazil | Toledo et al. 2006a |
| Hylodidae | *Megaelosia massarti* | DD | Brazil | Toledo et al. 2006a |
| **Hyperoliidae** | *Afrixalus clarkei* | EN | Ethiopia | Gower et al. 2012 |
| Hyperoliidae | *Afrixalus crotalus* | LC | Malawi | Conradie et al. 2011 Herp Rev |
| Hyperoliidae | *Afrixalus dorsalis* | LC | Cameroon, Gabon, Nigeria | Imasuen et al. 2011; Hirschfeld et al. 2016; Jongsma et al. 2016 |
| Hyperoliidae | *Afrixalus fornasini* | LC | Kenya, Japan (cap.) | Goka et al. 2009; Kielgast et al. 009 |
| Hyperoliidae | *Afrixalus fulvovittatus* | LC | Cameroon, Gabon | Bell et al. 2011; Hirschfeld et al. 2016 |
| Hyperoliidae | *Afrixalus lacteus* | EN | Cameroon | Hirschfeld et al. 2016 |
| Hyperoliidae | *Afrixalus laevis* | LC | Cameroon, DR Congo | Greenbaum et al. 2015; Seimon et al. 2015; Hirschfeld et al. 2016 |
| Hyperoliidae | *Afrixalus nigeriensis* | NT | Nigeria | Imasuen et al. 2011 |
| Hyperoliidae | *Afrixalus osorioi* | LC | DR Congo | Seimon et al. 2015 |
| Hyperoliidae | *Afrixalus paradorsalis* | LC | Cameroon, Gabon, Nigeria | Bell et al. 2011; Imasuen et al. 2011; Hirschfeld et al. 2016; Miller et al. 2018 |
| Hyperoliidae | *Afrixalus quadrivittatus, A. q. 1,* and *A. q. 2* | LC | Cameroon, DR Congo | Greenbaum et al. 2015; Seimon et al. 2015; Miller et al. 2018 |
| Hyperoliidae | *Afrixalus* sp. nov. | NE | Tanzania | Moyer and Weldon 2006 |
| Hyperoliidae | *Afrixalus spinifrons* | VU | South Africa | Tarrant et al. 2013 |
| Hyperoliidae | *Afrixalus sylvaticus* | EN | Kenya | Kielgast et al. 2009 |
| Hyperoliidae | *Afrixalus uluguruensis* | EN | Tanzania | Moyer and Weldon 2006 |
| Hyperoliidae | *Afrixalus wittei* | LC | DR Congo | Seimon et al. 2015 |
| Hyperoliidae | *Alexteroon hypsiphonus* | LC | Cameroon | Miller et al. 2018 |
| Hyperoliidae | *Alexteroon obstetricans* | LC | Cameroon | Miller et al. 2018 |
| Hyperoliidae | *Callixalus pictus* | VU | DR Congo | Greenbaum et al. 2015 |
| Hyperoliidae | *Chrysobatrachus cupreonitens* | EN | DR Congo | Greenbaum et al. 2015 |
| Hyperoliidae | *Cryptothylax greshoffii* | LC | Gabon | Jongsma et al. 2016 |
| Hyperoliidae | *Hyperolius acuticeps* | LC | Kenya, Tanzania | Moyer and Weldon 2006; Kielgast et al. 2009 |
| Hyperoliidae | *Hyperolius admetzi* | EN | Cameroon | Hirschfeld et al. 2016 |
| Hyperoliidae | *Hyperolius adspersus* | LC | Gabon | Jongsma et al. 2016 |
| Hyperoliidae | *Hyperolius balfouri* | LC | Cameroon | Hirschfeld et al. 2016 |
| Hyperoliidae | *Hyperolius bolifambae* | LC | Cameroon | Hirschfeld et al. 2016 |
| Hyperoliidae | *Hyperolius camerunensis* | LC | Cameroon | Hirschfeld et al. 2016 |
| Hyperoliidae | *Hyperolius castaneus* | LC | DR Congo, Rwanda, Uganda | Greenbaum et al. 2015; Seimon et al. 2015; van der Hoek et al. 2019 |
| Hyperoliidae | *Hyperolius cinnamomeoventris* | LC | Cameroon, Gabon, Rwanda, Uganda | Seimon et al. 2015; Hirschfeld et al. 2016; Jongsma et al. 2016; van der Hoek et al. 2019 |
| Hyperoliidae | *Hyperolius concolor* | LC | Cameroon, Gabon, Nigeria | Imasuen et al. 2011; Hirschfeld et al. 2016; Jongsma et al. 2016 |
| Hyperoliidae | *Hyperolius constellatus* | VU | DR Congo | Greenbaum et al. 2015; Seimon et al. 2015 |
| Hyperoliidae | *Hyperolius discodactylus* | LC | DR Congo, Rwanda | Seimon et al. 2015 |
| Hyperoliidae | *Hyperolius frontalis* | LC | DR Congo | Seimon et al. 2015 |
| Hyperoliidae | *Hyperolius fusciventris burtoni* | LC | Nigeria | Imasuen et al. 2011 |
| Hyperoliidae | *Hyperolius glandicolor* | LC | Kenya | Kielgast et al. 2009 |
| Hyperoliidae | *Hyperolius kivuensis* | LC | DR Congo, Ethiopia, Kenya, Rwanda, Uganda | Goldberg et al. 2007; Greenbaum et al. 2008, 2015; Kielgast et al. 2009; Gower et al. 2012; Seimon et al. 2015 |
| Hyperoliidae | *Hyperolius koehleri* | LC | Cameroon | Doherty-Bone et al. 2013 |
| Hyperoliidae | *Hyperolius kuligae* | LC | DR Congo | Greenbaum et al. 2008 |
| Hyperoliidae | *Hyperolius langi* | LC | DR Congo | Greenbaum et al. 2015 |
| Hyperoliidae | *Hyperolius lateralis* | LC | Kenya | Kielgast et al. 2009 |
| Hyperoliidae | *Hyperolius mitchelli* | LC | The Netherlands (cap.) | Spitzen-van der Sluijs et al. 2011 (cap.) |
| Hyperoliidae | *Hyperolius molleri* | LC | São Tomé and Princípe | Hydeman et al. 2013 |
| Hyperoliidae | *Hyperolius nasicus* | LC | DR Congo | Greenbaum et al. 2014 |
| Hyperoliidae | *Hyperolius nasutus* | LC | Cameroon, DR Congo | Seimon et al. 2015; Hirschfeld et al. 2016 |
| Hyperoliidae | *Hyperolius nitidulus* | LC | Cameroon | Hirschfeld et al. 2016 |
| Hyperoliidae | *Hyperolius ocellatus* | LC | Cameroon, Gabon | Bell et al. 2011; Hirschfeld et al. 2016; Jongsma et al. 2016 |
| Hyperoliidae | *Hyperolius pardalis* | LC | Gabon | Jongsma et al. 2016 |
| Hyperoliidae | *Hyperolius pickersgilli* | CR | South Africa | Tarrant et al. 2013 |
| Hyperoliidae | *Hyperolius picturatus* | LC | Nigeria | Imasuen et al. 2011 |
| Hyperoliidae | *Hyperolius pictus* | LC | Tanzania | Moyer and Weldon 2006 |
| Hyperoliidae | *Hyperolius pseudargus* | LC | Tanzania | Moyer and Weldon 2006 |
| Hyperoliidae | *Hyperolius puncticulatus* | EN | Tanzania | Moyer and Weldon 2006 |
| Hyperoliidae | *Hyperolius quinquevittatus* | LC | DR Congo | Greenbaum et al. 2014 |
| Hyperoliidae | *Hyperolius riggenbachi* and *H. r. hyeiroglyphica* | VU | Cameroon | Doherty-Bone et al. 2013; Hirschfeld et al. 2016 |
| Hyperoliidae | *Hyperolius robustus* | DD | DR Congo | Seimon et al. 2015 |
| Hyperoliidae | *Hyperolius spinigularis* | LC | Tanzania | Moyer and Weldon 2006 |
| Hyperoliidae | *Hyperolius sylvaticus* | LC | Nigeria | Imasuen et al. 2011 |
| Hyperoliidae | *Hyperolius thomensis* | EN | São Tomé and Princípe | Hydeman et al. 2013 |
| Hyperoliidae | *Hyperolius tuberculatus* | LC | DR Congo, Gabon | Bell et al. 2011; Greenbaum et al. 2015; Jongsma et al. 2016 |
| Hyperoliidae | *Hyperolius tuberilinguis* | LC | Kenya | Kielgast et al. 2009 |
| Hyperoliidae | *Hyperolius viridiflavus* and *H. v. pitmani* | LC | Ethiopia, Kenya, Rwanda, Uganda | Kielgast et al. 2009; Gower et al. 2012; Seimon et al. 2015; van der Hoek et al. 2019 |
| Hyperoliidae | *Kassina decorata* | VU | Cameroon | Hirschfeld et al. 2016 |
| Hyperoliidae | *Kassina maculosa* | LC | Cameroon | Hirschfeld et al. 2016 |
| Hyperoliidae | *Kassina senegalensis* | LC | DR Congo, Kenya, Tanzania, South Africa | Weldon 2005; Moyer and Weldon 2006; Kielgast et al. 2009; Greenbaum et al. 2014 |
| Hyperoliidae | *Paracassina obscura* | LC | Ethiopia | Gower et al. 2012 |
| Hyperoliidae | *Phlyctimantis leonardi* | LC | Cameroon, Gabon | Baláž et al. 2012; Jongsma et al. 2016 |
| Hyperoliidae | *Phlyictimantis verrucosus* | LC | DR Congo | Seimon et al. 2015 |
| **Leiopelmatidae** | *Leiopelma archeyi* | CR | New Zealand | Bell et al. 2004; Shaw et al. 2013; A. Haigh, unpub. |
| **Leptodactylidae** | *Adenomera (Leptodactylus* cf.*) andreae* | LC | Peru | Kosch et al. 2012; Russell et al. 2019 |
| Leptodactylidae | *Adenomera marmorata* | LC | Brazil | Gründler et al. 2012; Rodriguez et al. 2014; Coutinho et al. 2015 |
| Leptodactylidae | *Edalorhina perezi* | LC | Peru | Russell et al. 2019 |
| Leptodactylidae | *Engystomops freibergi* | LC | Peru | Russell et al. 2019 |
| Leptodactylidae | *Engystomops (Physalaemus) petersi* | LC | Ecuador, Peru | McCracken et al. 2009; Kosch et al. 2012; Tarvin et al. 2014 |
| Leptodactylidae | *Engystomops (Physalaemus) pustulosus* | LC | Colombia, Panama, Venezuela | Lips et al. 2006; Sánchez et al. 2008; Zumbado-Ulate et al. 2014; Acevedo et al. 2016; Rodríguez-Brenes et al. 2016; Flechas et al. 2017 |
| Leptodactylidae | *Leptodactylus albilabris* | LC | Puerto Rico | Burrowes et al. 2008; P Burrowes, A. Longo, & R. Joglar, unpub. |
| Leptodactylidae | *Leptodactylus bolivianus (ocellatus)* | LC | Argentina | Herrera et al. 2005; Ghirardi et al. 2009 |
| Leptodactylidae | *Leptodactylus chaquensis* | LC | Argentina, Brazil | Ghirardi 2011; Amorim et al. 2019 |
| Leptodactylidae | *Leptodactylus colombiensis* | LC | Colombia | Vásquez-Ochoa et al. 2012; Acevedo et al. 2016; Flechas et al. 2017 |
| Leptodactylidae | *Leptodactylus discodactylus* | LC | Ecuador | McCracken et al. 2009 |
| Leptodactyidae | *Leptodactylus elenae* | LC | Brazil | Becker et al. 2016 |
| Leptodactylidae | *Leptodactylus fallax* | CR | Dominica, Montserrat | Malhotra et al. 2007; Garcia et al. 2009; Hudson et al. 2016; M.C. Fisher, unpub. |
| Leptodactylidae | *Leptodactylus fuscus* | LC | Colombia | Flechas et al. 2017 |
| Leptodactylidae | *Leptodactylus gracilis* | LC | Argentina | Ghirardi et al. 2009 |
| Leptodactylidae | *Leptodactylus labyrinthicus* | LC | Brazil | Rodriguez et al. 2014 |
| Leptodactylidae | *Leptodactylus latrans* | LC | Argentina, Brazil | Ghirardi 2011; Agostino and Burrowes 2015; Coutinho et al. 2015; Ghirardi et al. 2018 |
| Leptodactylidae | *Leptodactylus leptodactyloides* | LC | Peru | Russell et al. 2019 |
| Leptodactyidae | *Leptodactylus knudseni* | LC | Brazil | Becker et al. 2016 |
| Leptodactylidae | *Leptodactylus mystaceus* | LC | Bolivia, Brazil | Valencia-Aguilar et al. 2015; Burrowes and de la Riva 2017 |
| Leptodactylidae | *Leptodactyus notoaktites* | LC | Brazil | Rodriguez et al. 2014 |
| Leptodactylidae | *Leptodactylus pentadactylus* | LC | Japan (cap.), Panama, Ecuador | Lips et al. 2006; Une et al. 2008; McCracken et al. 2009 |
| Leptodactylidae | *Leptodactylus petersii* | LC | Peru | Kosch et al. 2012; Russell et al. 2019 |
| Leptodactylidae | *Leptodactylus podicipinus* | LC | Brazil | Rodriguez et al. 2014 |
| Leptodactylidae | *Leptodactylus poecilochilus* | LC | Colombia, Costa Rica | Zumbado-Ulate et al. 2014; Acevedo et al. 2016; Flechas et al. 2017 |
| Leptodactyidae | *Leptodactylus pustulatus* | LC | Brazil | Becker et al. 2016 |
| Leptodactylidae | *Leptodactylus rhodomystax* | LC | Ecuador | McCracken et al. 2009 |
| Leptodactylidae | *Leptodactylus savagei* | LC | Costa Rica | Whitfield et al. 2013 |
| Leptodactylidae | *Leptodactylus* spp.^[[17]](#footnote-17)^ | — | Venezuela | Lampo et al. 2006 |
| Leptodactyidae | *Leptodactylus stenodema* | LC | Brazil | Becker et al. 2016 |
| Leptodactylidae | *Leptodactylus syphax* | LC | Bolivia | Burrowes and de la Riva 2017 |
| Leptodactylidae | *Leptodactylus wagneri* | LC | Ecuador | Tarvin et al. 2014 |
| Leptodactylidae | *Lithodytes lineatus* | LC | Colombia, Peru | Acevedo et al. 2016; Russell et al. 2019 |
| Leptodactylidae | *Paratelmatobius lutzii* | DD | Brazil | Rodriguez et al. 2014 |
| Leptodactylidae | *Physalaemus albifrons* | LC | Brazil | Amorim et al. 2019 |
| Leptodactylidae | *Physalaemus cuvieri* | LC | Brazil | Coutinho et al. 2015; Valencia-Aguilar et al. 2015; Navarro-Lozano et al. 2018; Amorim et al. 2019 |
| Leptodactylidae | *Physalaemus fernandezae* | LC | Argentina | Delgado et al. 2012 |
| Leptodactylidae | *Physalaemus henselii* | LC | Uruguay | Borteiro et al. 2009 |
| Leptodactylidae | *Physalaemus olfersii* | LC | Brazil | Rodriguez et al. 2014 |
| Leptodactylidae | *Physalaemus signifer* | LC | Brazil | Ruggeri et al. 2018 Dis Aquat Org |
| Leptodactylidae | *Pleurodema bibroni* | NT | Uruguay | Bardier et al. 2011 |
| Leptodactylidae | *Pleruodema bufoninum* | LC | Argentina | Ghirardi 2011; Ghirardi et al. 2014 |
| Leptodactylidae | *Pleurodema cinereum* | LC | Bolivia | de la Riva and Burrowes 2011; Burrowes and de la Riva 2017 |
| Leptodactylidae | *Pleurodema diplolister* | LC | Brazil | Amorim et al. 2019 |
| Leptodactylidae | *Pleurodema kriegi* | NT | Argentina | Lescano et al. 2013 |
| Leptodactylidae | *Pleurodema marmoratum (marmorata)* | LC | Bolivia, Chile, Peru | Seimon et al. 2007; Catenazzi et al. 2011; Solís et al. 2015; Burrowes and de la Riva 2017; Seimon et al. 2017; R. Solís, unpub. |
| Leptodactylidae | *Pleurodema somuncurense* | CR | Argentina | Arellano et al. 2017 |
| Leptodactylidae | *Pleurodema thaul* | LC | Argentina, Chile | Soto-Azat and Cunningham 2010; Bourke et al. 2011; Ghirardi 2011; Soto-Azat et al. 2013, 2016; Ghirardi et al. 2014; Bacigalupe et al. 2017 |
| **Limnodynastidae** | *Adelotus brevis* | NT | Australia | Berger et al. 1999; Speare and Berger 2005; Kriger and Hero 2007a; Murray et al. 2010 |
| Limnodynastidae | *Heleioporus australiacus* | VU | Australia | Berger et al. 1999 |
| Limnodynastidae | *Heleioporus barycragus* | LC | Australia | Murray et al. 2010 |
| Limnodynastidae | *Heleioporus eyrei* | LC | Australia | Berger et al. 1999 (cap.); Speare and Berger 2005; Murray et al. 2010 |
| Limnodynastidae | *Lechriodus fletcheri* | LC | Australia | Berger et al. 1999 |
| Limnodynastidae | *Limnodynastes dorsalis* | LC | Australia | Berger et al. 1999; Murray et al. 2010 |
| Limnodynastidae | *Limnodynastes dumerilii* and *L.d. fryi* | LC | Australia | Berger et al. 1998, 1999; Clemann et al. 2009; Murray et al. 2010 |
| Limnodynastidae | *Limnodynastes fletcheri* | LC | Australia | Ocock et al. 2013 |
| Limnodynastidae | *Limnodynastes peronii* | LC | Australia | Briggs and Burgin 2004; Kriger and Hero 2007a; Murray et al. 2010; Lettoof et al. 2013; Stockwell et al. 2016 |
| Limnodynastidae | *Limnodynastes tasmaniensis* | LC | Australia | Berger et al. 1998, 1999; Howard et al. 2010; Murray et al. 2010; Simpkins et al. 2010; Aziz et al. 2011; Ocock et al. 2013 |
| Limnodynastidae | *Limnodynastes terraereginae* | LC | Australia | Murray et al. 2010 |
| Limnodynastidae | *Neobatrachus kunapalari* | LC | Australia | Berger et al. 1999 |
| Limnodynastidae | *Neobatrachus pelobatoides* | LC | Australia | Murray et al. 2010 |
| Limnodynastidae | *Neobatrachus sudelli* | LC | Australia | Ocock et al. 2013 |
| **Mantellidae** | *Mantella* sp. | — | United States (cap.) | A Pessier, unpub. (cap.) |
| **Megophryidae** | *Leptobrachium hasseltii* | LC | Indonesia | Kusrini et al. 2008 |
| Megophryidae | *Leptobrachium hendricksoni* | LC | Malaysia | Savage et al. 2011 EcoHealth; Leblanc et al. 2014 |
| Megophryidae | *Leptolalax* sp.^[[18]](#footnote-18)^ | — | Laos | Swei et al. 2011 |
| Megophryidae | *Megophrys nasuta* | LC | United States (cap.) | A Pessier and J Mendelson, unpub. (cap.) |
| Megophryidae | *Ophryophryne* sp.^[[19]](#footnote-19)^ | — | Vietnam | Swei et al. 2011 |
| **Micrixalidae** | *Micrixalus saxicola* | VU | India | Molur et al. 2015 |
| **Microhylidae** | *Arcovomer* sp.^[[20]](#footnote-20)^ | — | Brazil | Rodriguez et al. 2014 |
| Microhylidae | *Chiasmocleis bassleri* | LC | Peru | Russell et al. 2019 |
| Microhylidae | *Chiasmocleis shudikarensis* | LC | French Guiana | Courtois et al. 2015 |
| Microhylidae | *Chiasmocleis tridactyla* | LC | Peru | Russell et al. 2019 |
| Microhylidae | *Chiasmocleis ventrimaculata* | LC | Peru | Russell et al. 2019 |
| Microhylidae | *Cophixalus australis* | NE | Australia | Edwards et al. 2019 |
| Microhylidae | *Cophixalus ornatus* | LC | Australia | Kriger and Hero 2007b |
| Microhylidae | *Ctenophryne (Nelsonophryne) aterrima* | LC | Panama | Lips et al. 2006 PNAS |
| Microhylidae | *Dyscophus antongilii* | LC | Thailand (cap.), United States (cap.) | Tehangamsuwan et al. 2017 (cap.); A Pessier and J Mendelson, unpub. (cap.) |
| Microhylidae | *Elachistocleis bicolor* | LC | Argentina | Arellano et al. 2009 |
| Microhylidae | *Gastrophryne carolinensis* | LC | United States | Phillips et al. 2014; Love et al. 2016; Watters et al. 2016, 2018; Marhanka et al. 2017 |
| Microhylidae | *Gastrophryne olivacea* | LC | Mexico, United States | Watters et al. 2016, 2019; Marhanka et al. 2017; Hernández-Martínez et al. 2019 |
| Microhylidae | *Hamptophryne boliviana* | LC | Peru | Russell et al. 2019 |
| Microhylidae | *Kaloula pulchra* | LC | Cambodia, Malaysia, Singapore | Gaertner et al. 2011; Mendoza et al. 2011; Savage et al. 2011 EcoHealth; Chong et al. 2018 |
| Microhylidae | *Metaphrynella pollicaris* | LC | Malaysia | Savage et al. 2011 EcoHealth |
| Microhylidae | *Microhyla annectens* | DD | Malaysia | Savage et al. 2011 EcoHealth |
| Microhylidae | *Microhyla fissipes* | LC | Cambodia | Gaertner et al. 2011 |
| Microhylidae | *Microhyla heymonsi* | LC | Cambodia, Malaysia | Gaertner et al. 2011; Savage et al. 2011 EcoHealth |
| Microhylidae | *Microhyla mantheyi* | LC | Singapore | Chong et al. 2018 |
| Microhylidae | *Microhyla mixtura* | LC | China | Zhu et al. 2014 |
| Microhylidae | *Microhyla pulchra* | LC | Cambodia | Gaertner et al. 2011; Mendoza et al. 2011 |
| Microhylidae | *Phrynomantis bifasciatus* | LC | Tanzania | Moyer and Weldon 2006 |
| Microhylidae | *Plethodontohyla tuberata* | VU | Japan (cap.) | Une et al. 2008 |
| Microhylidae | *Scaphiophryne boribory* | VU | Japan (cap.) | Tamukai et al. 2014 (cap.) |
| Microhylidae | *Scaphiophryne gottlebei* | EN | United States (cap.) | A Pessier and J Mendelson, unpub. (cap.) |
| Microhylidae | *Stereocyclops incrassatus* | LC | Brazil | Rodriguez et al. 2014 |
| **Myobatrachidae** | *Assa darlingtoni* | LC | Australia | Kriger and Hero 2007a |
| Myobatrachidae | *Crinia georgiana* | LC | Australia | Speare and Berger 2005; Murray et al. 2010; Riley et al. 2013 |
| Myobatrachidae | *Crinia glauerti* | LC | Australia | Murray et al. 2010 |
| Myobatrachidae | *Crinia insignifera* | LC | Australia | Murray et al. 2010 |
| Myobatrachidae | *Crinia pseudinsignifera* | LC | Australia | Berger et al. 1999; Murray et al. 2010 |
| Myobatrachidae | *Crinia signifera* | LC | Australia | Clemann et al. 2009; Hunter et al. 2009; Howard et al. 2010, 2012; Pauza and Driessen 2008; Pauza et al. 2010; Brannelly et al. 2015, 2018 Anim Cons; Stockwell et al. 2016; Scheele et al. 2017 |
| Myobatrachidae | *Crinia subinsignifera* | LC | Australia | Murray et al. 2010 |
| Myobatrachidae | *Crinia tasmaniensis* | LC | Australia | Pauza and Dreissen 2008; Pauza et al. 2010; Aziz et al. 2011 |
| Myobatrachidae | *Geocrinia rosea* | LC | Australia | Speare and Berger 2005; Murray et al. 2010 |
| Myobatrachidae | *Geocrinia vitellina* | VU | Australia | Murray et al. 2010 |
| Myobatrachidae | *Mixophyes carbinensis* | LC | Australia | Puschendorf et al. 2013 |
| Myobatrachidae | *Mixophyes fasciolatus* | LC | Australia | Berger et al. 1998 (cap.), 1999; Symonds et al. 2007; Murray et al. 2010, 2013; Narayan et al. 2014 |
| Myobatrachidae | *Mixophyes fleayi* | EN | Australia | Berger et al. 1998; Symonds et al. 2007; Murray et al. 2010 |
| Myobatrachidae | *Mixophyes iteratus* | EN | Australia | Murray et al. 2010 |
| Myobatrachidae | *Pseudophryne corroboree* | CR | Australia | Hunter et al. 2010; Murray et al. 2010 |
| Myobatrachidae | *Pseudophryne pengilleyi* | EN | Australia | Berger et al. 1999 (cap.); Hunter et al. 2010; Murray et al. 2010; Scheele et al. 2017 |
| Myobatrachidae | *Taudactylus acutirostris* | CR | Australia | Berger et al. 1998, 1999 |
| Myobatrachidae | *Taudactylus eungellensis* | CR | Australia | Berger et al. 1998; Retallick et al. 2004; Murray et al. 2010 |
| Myobatrachidae | *Uperoleia fusca* | LC | Australia | Kriger and Hero 2007a |
| Myobatrachidae | *Uperoleia laevigata* | LC | Australia | Murray et al. 2010 |
| **Nyctibatrachidae** | *Lankanectes corrugatus* | LC | Sri Lanka | Swei et al. 2011 |
| Nyctibatrachidae | *Nyctibatrachus humayuni* | VU | India | Dahanukar et al. 2013 |
| **Odontophrynidae** | *Macrogenioglottus alipioi* | LC | Brazil | Rodriguez et al. 2014 |
| Odontophrynidae | *Odontophrynus achalensis* | VU | Argentina | Lescano et al. 2013 |
| Odontophrynidae | *Odontophrynus maisuma* | NE | Uruguay | Borteiro et al. 2009 |
| Odontophrynidae | *Odontophrynus occidentalis* | LC | Argentina | Gutierrez et al. 2010; Ghirardi 2011 |
| Odontophrynidae | *Proceratophrys appendiculata* | LC | Brazil | Ruggeri et al. 2018 Dis Aquat Org |
| Odontophrynidae | *Proceratophrys boiei* | LC | Brazil | Ruggeri et al. 2018 Dis Aquat Org |
| Odontophrynidae | *Proceratophrys melanopogon* | LC | Brazil | Rodriguez et al. 2014 |
| Odontophrynidae | *Proceratophrys renalis* | NE | Brazil | Valencia-Aguilar et al. 2015, 2016 |
| **Pelobatidae** | *Pelobates cultripes* | NT | Spain | Hidalgo-Vila et al. 2012; S. Walker, unpub. |
| Pelobatidae | *Pelobates fuscus* | LC | Germany | Ohst et al. 2013 |
| Pelobatidae | *Pelobates varaldii* | EN | Morocco | El Mouden et al. 2011 |
| **Pelodryadidae^[[21]](#footnote-21)^** | *Litoria adelaidensis* | LC | Australia | Berger et al. 1999; Speare and Berger 2005; Murray et al. 2010 |
| Pelodryadidae | *Litoria aurea* | VU | Australia, New Zealand | Speare and Berger 2005; Murray et al. 2010; Shaw et al. 2013; Stockwell et al. 2015, 2016; Klop-Toker et al. 2016; A. Haigh, unpub. |
| Pelodryadidae | *Litoria burrowsi (burrowsae)* | LC | Australia | Pauza and Driessen 2008; Pauza et al. 2010 |
| Pelodryadidae | *Litoria caerulea* | LC | Australia, Japan (cap.), United States (cap.) | Berger et al. 1998, 1999; Pessier et al. 1999; Goka et al. 2009; Murray et al. 2010; A. Pessier and J. Mendelson, unpub. (cap.) |
| Pelodryadidae | *Litoria chloris* | LC | Australia | Berger et al. 1999; Kriger and Hero 2007a, 2008; Murray et al. 2010 |
| Pelodryadidae | *Litoria citropa* | LC | Australia (cap.) | Berger et al. 1999 (cap.) |
| Pelodryadidae | *Litoria cooloolensis* | EN | Australia | Simpkins et al. 2017 |
| Pelodryadidae | *Litoria (Nyctimystes) dayi* | EN | Australia | Berger et al. 1999; Murray et al. 2010 |
| Pelodryadidae | *Litoria ewingii* | LC | Australia, New Zealand | Berger et al. 1999; Obendorf 2005; Obendorf and Dalton 2006; Pauza and Driessen 2008; Murray et al. 2010; Shaw et al. 2013; Pauza et al. 2010; Aziz et al. 2011; A. Haigh, unpub. |
| Pelodryadidae | *Litoria fallax* | LC | Australia | Kriger and Hero 2007a; Murray et al. 2010; Stockwell et al. 2016 |
| Pelodryadidae | *Litoria freycineti* | VU | Australia | Simpkins et al. 2017 |
| Pelodryadidae | *Litoria genimaculata* | LC | Australia | Berger et al. 1999; Murray et al. 2010; Woodhams et al. 2010 Div Dist |
| Pelodryadidae | *Litoria gracilenta* | LC | Australia | Murray et al. 2010 |
| Pelodryadidae | *Litoria jungguy^[[22]](#footnote-22)^* | NT | Australia | Kriger et al. 2007 Cons Biol; Retallick et al. 2004; Murray et al. 2010; Puschendorf et al. 2013 |
| Pelodryadidae | *Litoria latopalmata* | LC | Australia | Kriger and Hero 2007a Div Dist; Murray et al. 2010; Ocock et al. 2013; Simpkins et al. 2010 |
| Pelodryadidae | *Litoria lesueurii* | LC | Australia | Berger et al. 1998, 1999; Morehouse et al. 2003; Kriger et al. 2007 Cons Biol; Murray et al. 2010; Skerratt et al. 2010 |
| Pelodryadidae | *Litoria lorica* | CR | Australia | Murray et al. 2010; Puschendorf et al. 2011 |
| Pelodryadidae | *Litoria moorei* | LC | Australia | Berger et al. 1999; Murray et al. 2010 |
| Pelodryadidae | *Litoria nannotis* | EN | Australia | Berger et al. 1998; Murray et al. 2010; Puschendorf et al. 2011, 2013 |
| Pelodryadidae | *Litoria nasuta* | LC | Australia | Murray et al. 2010 |
| Pelodryadidae | *Litoria olongburensis* | VU | Australia | Simpkins et al. 2017 |
| Pelodryadidae | *Litoria pallida* | LC | Australia | Catullo et al. 2018 |
| Pelodryadidae | *Litoria paraewingii* | LC | Australia | Clemann et al. 2009 |
| Pelodryadidae | *Litoria pearsoniana* | NT | Australia | Berger et al. 1999; Kriger and Hero 2007a (Div Dist), 2008; Murray et al. 2010 |
| Pelodryadidae | *Litoria peronii* | LC | Australia | Briggs and Burgin 2004; Murray et al. 2010; Ocock et al. 2013; Stockwell et al. 2016 |
| Pelodryadidae | *Litoria raniformis* | EN | Australia, New Zealand | Waldman et al. 2001; Murray et al. 2010; Vörös et al. 2011; Heard et al. 2014 Shaw et al. 2013; A. Haigh, unpub. |
| Pelodryadidae | *Litoria rheocola* | EN | Australia | Berger et al. 1998, 1999; Murray et al. 2010; Phillott et al. 2013; Sapsford et al. 2013; Roznik et al. 2015; Sapsford et al. 2015; Grogan et al. 2016 |
| Pelodryadidae | *Litoria rubella* | LC | Australia | Ocock et al. 2013 |
| Pelodryadidae | *Litoria serrata* | NE | Australia | Puschendorf et al. 2013; Hagman and Alford 2015 |
| Pelodryadidae | *Litoria spenceri* | CR | Australia | Berger et al. 1998, 1999; Murray et al. 2010; Gillespie et al. 2015 |
| Pelodryadidae | *Litoria tyleri* | LC | Australia | Kriger and Hero 2007a |
| Pelodryadidae | *Litoria verreauxii* and  *L. v. alpina* | LC | Australia | Clemann et al. 2009; Hunter et al. 2009; Scheele et al. 2014, 2015 Biol Cons, 2016 JAE; Brannelly et al. 2015, 2016 |
| Pelodryadidae | *Litoria wilcoxii^[[23]](#footnote-23)^* | LC | Australia | Berger et al. 1998, 1999; Retallick et al. 2004; Kriger and Hero 2006, 2007 (Div Dist), 2008; Van Sluys and Hero 2009; Murray et al. 2010; Simpkins et al. 2010; Kindermann et al. 2012, 2017 |
| Pelodryadidae | *Litoria wilcoxii/jungguy* | LC | Australia | Kriger et al. 2007a Cons Biol; Rowley et al. 2007 Australia |
| Pelodryadidae | *Litoria xanthomera* | LC | Australia | Murray et al. 2010 |
| Pelodryadidae | *Nyctimystes infrafrenatus* | LC | Australia | Berger et al. 1999 (cap.); Murray et al. 2010 |
| **Petropedetidae** | *Arthroleptides (Petropedetes) yakusini* | EN | Tanzania | Weldon and du Preez 2004; Moyer & Weldon 2006; Weldon et al. 2020 |
| Petropedetidae | *Ericabatrachus baleensis* | CR | Ethiopia | Gower et al. 2012 |
| Petropedetidae | *Petropedetes euskircheni* | NE | Cameroon | Hirschfeld et al. 2016 |
| Petropedetidae | *Petropedetes johnstoni* | NT | Gabon | Bell et al. 2011 |
| Petropedetidae | *Petropedetes palmipes* | EN | Gabon | Bell et al. 2011 |
| Petropedetidae | *Petropedetes* sp. nov. | NE | Nigeria | Reeder et al. 2011 |
| **Phrynobatrachidae^[[24]](#footnote-24)^** | *Phrynobatrachus acridoides* | LC | Kenya | Kielgast et al. 2009 |
| Phrynobatrachidae | *Phrynobatrachus acutirostris* | NT | DR Congo | Greenbaum et al. 2015 |
| Phrynobatrachidae | *Phrynobatrachus africanus* | LC | Cameroon | Doherty-Bone et al. 2013; Hirschfeld et al. 2016 |
| Phrynobatrachidae | *Phrynobatrachus asper* | VU | DR Congo | Greenbaum et al. 2015 |
| Phrynobatrachidae | *Phrynobatrachus auritus* | LC | Cameroon, Gabon | Bell et al. 2011; Hirschfeld et al. 2016; Miller et al. 2018 |
| Phrynobatrachidae | *Phrynobatrachus calcaratus* | LC | Cameroon, Nigeria | Imasuen et al. 2011; Doherty-Bone et al. 2013 |
| Phrynobatrachidae | *Phrynobatrachus cornutus* | LC | Cameroon | Hirschfeld et al. 2016 |
| Phrynobatrachidae | *Phrynobatrachus cricogaster* | NT | Cameroon | Doherty-Bone et al. 2013; Hirschfeld et al. 2016 |
| Phrynobatrachidae | *Phrynobatrachus dendrobates* | LC | DR Congo | Greenbaum et al. 2015 |
| Phrynobatrachidae | *Phrynobatrachus graueri* | LC | DR Congo | Greenbaum et al. 2015 |
| Phrynobatrachidae | *Phrynobatrachus graueri/parvulus* | — | Rwanda | van der Hoek et al. 2019 |
| Phrynobatrachidae | *Phrynobatrachus krefftii* | EN | DR Congo | Seimon et al. 2015 |
| Phrynobatrachidae | *Phrynobatrachus liberiensis* | NT | Nigeria | Imasuen et al. 2011 |
| Phrynobatrachidae | *Phrynobatrachus mababiensis* | LC | Tanzania | Makange et al. 2014 |
| Phrynobatrachidae | *Phrynobatrachus minutus* | LC | Ethiopia | Gower et al. 2012 |
| Phrynobatrachidae | *Phrynobatrachus natalensis* | LC | DR Congo, Malawi, South Africa | Conradie et al. 2011; Greenbaum et al. 2014, 2015; C. Weldon, unpub. |
| Phrynobatrachidae | *Phrynobatrachus perpalmatus* | LC | DR Congo | Greenbaum et al. 2015 |
| Phrynobatrachidae | *Phrynobatrachus plicatus* | LC | Nigeria | Imasuen et al. 2011 |
| Phrynobatrachidae | *Phrynobatrachus steindachneri* | CR | Cameroon | Doherty-Bone et al. 2013; Hirschfeld et al. 2016 |
| Phrynobatrachidae | *Phrynobatrachus werneri* | LC | Cameroon | Doherty-Bone et al. 2013; Hirschfeld et al. 2016 |
| **Phyllomedusidae^[[25]](#footnote-25)^** | *Agalychnis annae* | EN | Costa Rica | Whitfield et al. 2017 |
| Phyllomedusidae | *Agalychnis callidryas* | LC | Costa Rica, Nicaragua, Panama; United States (cap.) | Lips et al. 2006; Brem and Lips 2008; García-Roa et al. 2014; Rebollar et al. 2014; Zumbado-Ulate et al. 2019A Pessier and J Mendelson, unpub. (cap.) |
| Phyllomedusidae | *Agalychnis granulosa* | LC | Brazil | Valencia-Aguilar et al. 2016 |
| Phyllomedusidae | *Agalychnis (Phyllomedusa) lemur* | CR | Costa Rica, Panama | Lips et al. 2006; Whitfield et al. 2017 |
| Phyllomedusidae | *Agalychnis moreletii* | CR | Mexico, El Salvador, Belize | Felger et al. 2007; Frías-Alvarez et al. 2008; Lawson et al. 2011; Kaiser and Pollinger 2012 |
| Phyllomedusidae | *Agalychnis spurrelli* | LC | Costa Rica | Zumbado-Ulate et al. 2019 |
| Phyllomedusidae | *Cruziohyla calcarifer* | LC | Costa Rica | Zumbado-Ulate et al. 2019 |
| Phyllomedusidae | *Phrynomedusa* cf. *marginata* | LC | Brazil | Toledo et al. 2006a S Amer J Herpetol |
| Phyllomedusidae | *Phyllomedusa bahiana* | DD | Brazil | Amorim et al. 2019 |
| Phyllomedusidae | *Phyllomedusa burmeisteri* | LC | Brazil | Coutinho et al. 2015 |
| Phyllomedusidae | *Phyllomedusa camba* | LC | Peru | Russell et al. 2019 |
| Phyllomedusidae | *Phyllomedusa distincta* | LC | Brazil | Rodriguez et al. 2014 |
| Phyllomedusidae | *Phyllomedusa tetraploidea* | LC | Brazil | Rodriguez et al. 2014 |
| Phyllomedusidae | *Phyllomedusa venusta* | LC | Colombia | Acevedo et al. 2016 |
| Phyllomedusidae | *Pithecopus hypochondrialis* | LC | Colombia, The Netherlands (cap.) | Spitzen-van der Sluijs et al. 2011 (cap.); Flechas et al. 2017 |
| Phyllomedusidae | *Pithecopus nordestius* | DD | Brazil | Valencia-Aguilar et al. 2015 |
| Phyllomedusidae | *Pithecopus palliatus* | LC | Peru | Russell et al. 2019 |
| Phyllomedusidae | *Pithecopus rohdei* | LC | Brazil | Coutinho et al. 2015 |
| **Pipidae** | *Hymenochirus boettgeri* | LC | Canada | Raverty and Reynolds 2001 |
| Pipidae | *Hymenochirus curtipes* | LC | United States (cap.) | Murphy et al. 2015 |
| Pipidae | *Pipa pipa* | LC | Peru | Russell et al. 2019 |
| Pipidae | *Xenopus amieti* | VU | Cameroon, Czech Republic (cap.), Gabon | Doherty-Bone et al. 2013; Havlíková et al. 2015; Jongsma et al. 2016 |
| Pipidae | *Xenopus andrei* | LC | Cameroon | Miller et al. 2018 |
| Pipidae | *Xenopus borealis* | LC | Kenya | Kielgast et al. 2009; Vredenburg et al. 2013 |
| Pipidae | *Xenopus clivii* | LC | Ethiopia | Gower et al. 2012 |
| Pipidae | *Xenopus (Silurana) epitropicalis* | LC | Cameroon | Miller et al. 2018 |
| Pipidae | *Xenopus eysoole* | DD | Cameroon | Hirschfeld et al. 2016 |
| Pipidae | *Xenopus fraseri* | LC | Cameroon | Soto-Azat et al. 2009; Doherty-Bone et al. 2013; Hirschfeld et al. 2016 |
| Pipidae | *Xenopus gilli* | EN | South Africa | Weldon 2005; Soto-Azat et al. 2009; Tarrant et al. 2013 |
| Pipidae | *Xenopus laevis* | LC | Botswana, Chile, China (cap.), DR Congo, Ghana, Hong Kong (cap.), Japan, Kenya, Malawi, South Africa, Uganda, United Kingdom, United States (cap.) | Weldon 2005; Speare and Berger 2005; Goka et al. 2009; Soto-Azat et al. 2009, 2016; Solís et al. 2010; Bai et al. 2012 (cap.); Vredenburg et al. 2013; Greenbaum et al. 2014; Kolby et al. 2014; Tamukai et al. 2014 (cap.); Tinsley et al. 2015; Zhu et al. 2016 (cap.); Bacigalupe et al. 2017; Wilson et al. 2018; Unpub. data from: J. Longcore; R. Solís; A. Pessier and J. Mendelson,. (cap.); C. Weldon |
| Pipidae | *Xenopus longipes* | CR | Cameroon | Hirschfeld et al. 2016 |
| Pipidae | *Xenopus mellotropicalis* | NE | Gabon | Jongsma et al. 2016 |
| Pipidae | *Xenopus muelleri* | LC | Mozambique, Swaziland | Weldon 2005; Conradie et al. 2011 Herp Rev, 2016 |
| Pipidae | *Xenopus parafraseri* | LC | Cameroon | Hirschfeld et al. 2016 |
| Pipidae | *Xenopus petersii* | LC | Botswana | Weldon 2005 |
| Pipidae | *Xenopus* sp. nov. |  | Cameroon | Hirschfeld et al. 2016 |
| Pipidae | *Xenopus (Silurana) tropicalis* | LC | Nigeria, United States (cap.) | Parker et al. 2002 (cap.); Imasuen et al. 2011 |
| Pipidae | *Xenopus victorianus* | LC | Kenya | Kielgast et al. 2009 |
| Pipidae | *Xenopus wittei* | LC | DR Congo, Uganda | Goldberg et al. 2007; Seimon et al. 2015 |
| **Ptychadenidae** | *Ptychadena aequiplicata* | LC | Tanzania | Weldon and du Preez 2004 |
| Ptychadenidae | *Ptychadena anchietae* | LC | Kenya, Tanzania, Uganda | Berger and Speare 2000; Weldon and du Preez 2004; Speare and Berger 2005; Kielgast et al. 2009; Seimon et al. 2015; Weldon et al. 2020 |
| Ptychadenidae | *Ptychadena chrysogaster* | LC | DR Congo, Uganda | Seimon et al. 2015 |
| Ptychadenidae | *Ptychadena erlangeri* | NT | Ethiopia | Gower et al. 2012 |
| Ptychadenidae | *Ptychadena longirostris* | LC | Nigeria | Imasuen et al. 2011 |
| Ptychadenidae | *Ptychadena mascareniensis* | LC | Kenya, Uganda | Goldberg et al. 2007; Rödder et al. 2009 |
| Ptychadenidae | *Ptychadena neumanni* | LC | Ethiopia | Gower et al. 2012 |
| Ptychadenidae | *Ptychadena newtoni* | EN | São Tomé and Princípe | Hydeman et al. 2013 |
| Ptychadenidae | *Ptychadena perreti* | LC | Gabon | Jongsma et al. 2016 |
| Ptychadenidae | *Ptychadena pumilio* | LC | Nigeria | Imasuen et al. 2011 |
| Ptychadenidae | *Ptychadena* sp.^[[26]](#footnote-26)^ |  | Cameroon | Doherty-Bone et al. 2013; Hirschfeld et al. 2016 |
| Ptychadenidae | *Ptychacena uzungwensis* | LC | DR Congo | Greenbaum et al. 2014 |
| **Pyxicephalidae** | *Amietia (Afrana) angolensis* | LC | Kenya, Malawi, South Africa, Tanzania, Uganda | Weldon 2005; Kielgast et al. 2009; Conradie et al. 2011 Afr Zool, 2011 Herp Rev; Zancolli et al. 2013; Seimon et al. 2015; C. Weldon, unpub. |
| Pyxicephalidae | *Amietia (Afrana) delandii (dracomontana)* | NE | Lesotho, South Africa | Weldon 2005 |
| Pyxicephalidae | *Amietia (Afrana) fuscigula* | LC | South Africa | Hopkins and Channing 2003; Lane et al. 2003; Weldon 2005; C. Weldon, unpub. |
| Pyxicephalidae | *Amietia johnstoni* | EN | Malawi | Conradie et al. 2011 Herp Rev |
| Pyxicephalidae | *Amietia ruwenzorica* | DD | Uganda | Viertel et al. 2012 |
| Pyxicephalidae | *Amietia* sp. Force Bendera | — | Democratic Republic of Congo | Greenbaum et al. 2015 |
| Pyxicephalidae | *Amietia* sp. Marungu Plateau | — | Democratic Republic of Congo | Greenbaum et al. 2014 |
| Pyxicephalidae | *Amietia* sp. Itombwe | — | Democratic Republic of Congo | Greenbaum et al. 2015 |
| Pyxicephalidae | *Amietia* sp. Mayola River | — | Democratic Republic of Congo | Greenbaum et al. 2014 |
| Pyxicephalidae | *Amietia vertebralis* | LC | Lesotho | Weldon 2005 |
| Pyxicephalidae | *Amietia wittei* | DD | Kenya, Tanzania | Kielgast et al. 2009; Zancolli et al. 2013 |
| Pyxicephalidae^[[27]](#footnote-27)^ | *Cacosternum boettgeri* | LC | South Africa | Weldon 2005; C. Weldon, unpub. |
| Pyxicephalidae | *Microbatrachella capensis* | CR | South Africa | Tarrant et al. 2013 |
| Pyxicephalidae | *Nothophryne broadleyi* | EN | Malawi | Conradie et al. 2011 Herp Rev |
| Pyxicephalidae | *Pyxicephalus adspersus* | LC | United States (cap.) | Churgin et al. 2013 (cap.); A Pessier and J Mendelson, unpub. (cap.) |
| Pyxicephalidae | *Strongylopus fasciatus* | LC | South Africa | Weldon 2005 |
| Pyxicephalidae | *Strongylopus fuelleborni* | LC | Malawi | Conradie et al. 2011 Herp Rev |
| Pyxicephalidae | *Strongylopus grayii* | LC | South Africa | Hopkins and Channing 2003; C. Weldon, unpub. |
| Pyxicephalidae | *Strongylopus hymenopus* | LC | Lesotho, South Africa | Smith et al. 2007; M.-O. Rödel, unpub. |
| Pyxicephalidae | *Tomopterna cryptotis* | LC | South Africa | Weldon 2005 |
| Pyxicephalidae | *Tomopterna natalensis* | LC | South Africa | Weldon 2005; C. Weldon, unpub. |
| **Ranidae** | *Amnirana (Hylarana) albolabris* | LC | Cameroon, Gabon, Nigeria | Imasuen et al. 2011; Hirschfeld et al. 2016; Jongsma et al. 2016 |
| Ranidae | *Amnirana lepus* | LC | Cameroon, Gabon | Hirschfeld et al. 2016; Jongsma et al. 2016 |
| Ranidae | *Babina adenopleura* | LC | China | Zhu et al. 2014 |
| Ranidae | *Babina daunchina* | LC | China | Zhu et al. 2014 |
| Ranidae | *Babina (Rana) pleuraden* | LC | China | Bai et al. 2010; Zhu et al. 2014 |
| Ranidae | *Chalcorana (Hylarana) chalconota* | LC | Indonesia | Kusrini et al. 2008 ; Swei et al. 2011 |
| Ranidae | *Chalcorana labialis* | NE | Singapore | Chong et al. 2018 |
| Ranidae | *Chalcorana (Hylarana) macrops* | NT | Indonesia | Swei et al. 2011 |
| Ranidae | *Glandirana emeljanovi* | LC | North Korea | Fong et al. 2015 |
| Ranidae | *Glandirana (Rana) rugosa* | LC | Japan, South Korea | Goka et al. 2009; Bataille et al. 2013; Rios-Sotelo et al. 2018 |
| Ranidae | *Huia masonii (javana)* | VU | Indonesia | Swei et al. 2011 |
| Ranidae | *Hylarana (Rana) erythraea* | LC | Cambodia | Gaertner et al. 2011 |
| Ranidae | *Indosylvirana (Hylarana) temporalis* | NT | India, Sri Lanka | Swei et al. 2011; Molur et al. 2015 |
| Ranidae | *Lithobates areolatus* | NT | United States | Kinney et al. 2011 ; Terrell et al. 2014 |
| Ranidae | *Lithobates (Rana) berlandieri* | LC | Belize, Mexico, United States, | Sredl and Caldwell 2000; Lovich et al. 2008; Kaiser and Pollinger 2012; Murrieta-Galindo et al. 2014; Hernández-Martínez et al. 2019; Marshall et al. 2019; Arizona Game & Fish Dept, unpub. |
| Ranidae | *Lithobates (Rana) blairi* | LC | United States | Sredl and Caldwell 2000; Carey and Livo 2009; Harner et al. 2013; McTaggart et al. 2014; Beyer et al. 2015; Watters et al. 2016; Marhanka et al. 2017; Arizona Game & Fish Dept, unpub.; A Pessier and J Mendelson, unpub. (cap.) |
| Ranidae | *Lithobates brownorum* | NE | Nicaragua | García-Roa et al. 2014 |
| Ranidae | *Lithobates catesbeianus*  *(Rana catesbeiana)* | LC | Argentina, Belgium, Brazil, Canada, China, France, Italy, Japan, Mexico, The Netherlands, Singapore (cap.), South Korea, United Kingdom, United States, Uruguay, Venezuela | Rosen and Schwalbe 2002; Sredl et al. 2002; Mazzoni et al. 2003; Morehouse et al. 2003; Hanselmann et al. 2004; Cunningham et al. 2005; Daszak et al. 2005; Ouellet et al. 2005, 2012; Pearl and Green 2005; Bettaso and Rachowicz 2006; Charbonneau 2006; Garner et al. 2005, 2006; Govindarajulu et al. 2006; Green and Dodd 2007; Longcore et al. 2007; Morgan et al. 2007; Pearl et al. 2007; Peterson et al. 2007; Schlaepfer et al. 2007; MJ Adams et al. 2008, 2010; Campbell Grant et al. 2008; Rothermel et al. 2008, 2016; Sánchez et al. 2008; Goka et al. 2009; Rizkalla 2009; Schloegel et al. 2009 (cap.); Yang et al. 2009; Alminas et al. 2010; Bai et al. 2010, 2012; Gaudreau et al. 2010; Rimer and Briggler 2010; Rizkalla 2010; Sadinski et al. 2010; Saenz et al. 2010; Fellers et al. 2011; Forrest and Schlaepfer 2011; Ghirardi 2011; Ghirardi et al. 2011, 2017 Belg J Zool; Harner et al. 2011, 2013; Lannoo et al. 2011; Tupper et al. 2011; Gilbert et al. 2012 (cap.); Krynak et al. 2012; Bataille et al. 2013; Huss et al. 2013; Martel et al. 2013; Muelleman and Montgomery 2013; Richards-Hrdlicka et al. 2013; Vieira et al. 2013; Galindo-Bustos et al. 2014; Hughey et al. 2014; Lenker et al. 2014; McTaggart et al. 2014; Peterson and McKenzie et al. 2014; Phillips et al. 2014; Richardson et al. 2014; Rodriguez et al. 2014; Spitzen-van der Sluijs et al. 2014; Windstam and Olori 2014; Beyer et al. 2015; Forrest et al. 2015; Wilson et al. 2015, 2018; Battaglin et al. 2016; Jenkinson et al. 2016; Kadekaru et al. 2016; Love et al. 2016; Petersen et al. 2016; Sacerdote-Velat et al. 2016; Watters et al. 2016, 2018, 2019; Zhu et al. 2016 (cap.); AJ Adams et al. 2017 Ecol Evol, 2017 Ecosphere; Jaeger et al. 2017; Borzée et al. 2017; Marhanka et al. 2017; Stutz et al. 2017; Tupper et al. 2017; Bakland 2018; Laufer et al. 2018; Olori et al. 2018; Peralta-García et al. 2018; Rios-Sotelo et al. 2018; Robinson et al. 2018; Urbina et al. 2018; Goodman et al. 2019; Hernández-Martínez et al. 2019; Jongsma et al. 2019; Unpub. data from: Arizona Game & Fish dept.; M. Byrne; Canadian Cooperative Wildlife Health Centre; R. Fisher; TWJ Garner; Gibbons et al.; D.E. Green; M. Hayes; V. Hemingway; J Longcore; J. Kolby; J. Krebs; J. Mendelson.; G Padgett-Flohr; A. Pessier and J. Mendelson (cap.); P. Rosen and D. Caldwell.; J. Ware and K. Duncan; http://www.promedmail.org (Y. Ume) |
| Ranidae | *Lithobates (Rana) chiricahuensis* | VU | United States | Morell 1999; Bradley et al. 2002; Rosen and Schwalbe 2002; Sredl et al. 2002; Unpub. data from: Arizona Game & Fish Dept.; D.E. Green; A. Pessier and J. Mendelson (cap.); P.C. Rosen and D. Caldwell |
| Ranidae | *Lithobates (Rana) clamitans* and *L.cl. melanota* | LC | Canada, United States | Ouellet et al. 2005; Longcore et al. 2007; Byrne et al. 2008; Campbell Grant et al. 2008; Rothermel et al. 2008, 2016; St-Amour et al. 2008, 2010; Timpe et al. 2008; Chinnadurai et al. 2009; Forzán et al. 2010; Gaudreau et al. 2010; Groner and Relyea 2010; Monsen-Collar et al. 2010; Sadinski et al. 2010; Hill et al. 2011; Lannoo et al. 2011; Tupper et al. 2011, 2017; Becker et al. 2012; Boivin 2012; Brannelly et al. 2012; Klemish et al. 2012; Krynak et al. 2012; Richards-Hrdlicka et al. 2013; Hanlon et al. 2014 (Tennessee); Igleski and Nicholson 2014; Korfel and Hetherington 2014; Lenker et al. 2014; Phillips et al. 2014; Richardson et al. 2014; Windstam and Olori 2014; Wolff et al. 2014; Beyer et al. 2015; Wilson et al. 2015; Battaglin et al. 2016; Julian et al. 2016, 2019; Love et al. 2016; Petersen et al. 2016; Sacerdote-Velat et al. 2016; Chiari et al. 2017; Marhanka et al. 2017; Bakland 2018; Olori et al. 2018; Robinson et al. 2018; Watters et al. 2018; Jongsma et al. 2019; Unpub. data from: D.E. Green; J. Ware and K. Duncan; A. Pessier and J. Mendelson (cap.); M. Whitney; V. St-Amour |
| Ranidae | *Lithobates forreri* | LC | Costa Rica, Honduras, Mexico | Zumbado-Ulate et al. 2014; Gutsche et al. 2015; Köhler et al. 2016 |
| Ranidae | *Lithobates (Rana) heckscheri* | LC | United States | D.E. Green, unpub. |
| Ranidae | *Lithobates juliani* | NT | Belize | Kaiser and Pollinger 2012 |
| Ranidae | *Lithobates macroglossa* | VU | Guatemala | Mendelson et al. 2014 |
| Ranidae | *Lithobates maculatus (Rana maculata)* | LC | El Salvador, Honduras, Nicaragua | Puschendorf et al. 2006b EcoHealth; Felger et al. 2007; Kolby et al. 2010; García-Roa et al. 2014; Gutsche et al. 2015 |
| Ranidae | *Lithobates (Rana) magnaocularis* | LC | Mexico | Hale et al. 2005 |
| Ranidae | *Lithobates (Rana) megapoda* | VU | Mexico | Frías-Alvarez et al. 2008 |
| Ranidae | *Lithobates (Rana) montezumae* | LC | Mexico | Frías-Alvarez et al. 2008; Galindo-Bustos et al. 2014 |
| Ranidae | *Lithobates neovolcanicus (Rana neovolcanica)* | NT | Mexico | Frías-Alvarez et al. 2008 |
| Ranidae | *Lithobates onca* | EN | United States | Jaeger et al. 2017 |
| Ranidae | *Lithobates (Rana) palustris* | LC | Canada, United States | Ouellet et al. 2005; Longcore et al. 2007; Campbell Grant et al. 2008; Rothermel et al. 2008; Timpe et al. 2008; Todd-Thompson et al. 2009; Gaudreau et al. 2010; Rimer and Briggler 2010; Davidson and Chambers 2011; Goodman and Ararso 2012; Krynak et al. 2012; Richards-Hrdlicka et al. 2013; Petersen et al. 2016; Robinson et al. 2018; A. Pessier and J. Mendelson, unpub. (cap.) |
| Ranidae | *Lithobates (Rana) pipiens* and *L. p. sp. ‘E’* | LC | Canada, Panama, United States | Carey et al. 1999; Muths et al. 2003; Ouellet et al. 2005; Lips et al. 2006; Longcore et al. 2007; Young et al. 2007; Brem and Lips 2008; Woodhams et al. 2008 (cap.); Loda and Otis 2009; Rodriguez et al. 2009; Forzán et al. 2010; Sadinski et al. 2010; Voordouw et al. 2010; Stevens et al. 2012; Brown and Kerby 2013; Richards-Hrdlicka et al. 2013; Lenker et al. 2014; Firkins 2015; Hyman and Collins 2015; Talley et al. 2015; Petersen et al. 2016; Sacerdote-Velat et al. 2016; Araos et al. 2017; Olori et al. 2018; McMillan et al. 2019; Unpub. data from: Canadian Cooperative Wildlife Health Centre; D.E. Green; K. Kendell; J. Loda; J. Longcore; B. Maxell; Northern Leopard Frog Recovery Team; A. Pessier; A. Pessier and J. Mendelson, unpub. (cap.); S. Wagner, J. Johnson, and S. Germaine |
| Ranidae | *Lithobates* cf. *pueblae* | CR | Mexico | Köhler et al. 2019 Mexico |
| Ranidae | *Lithobates pustulosus (Rana pustulosa)* | LC | Mexico | Hale et al. 2005 |
| Ranidae | *Lithobates (Rana) septentrionalis* | LC | Canada, United States | Ouellet et al. 2005; Longcore et al. 2007; Rodriguez et al. 2009; Sadinski et al. 2010; Lenker et al. 2014; Battaglin et al. 2016; Petersen et al. 2016; D.E. Green, unpub. |
| Ranidae | *Lithobates sevosus (Rana sevosa)* | CR | United States | Drake et al. 2007 |
| Ranidae | *Lithobates sierramadrensis* | VU | Mexico | Lips et al. 2004; Köhler et al. 2016 |
| Ranidae | *Lithobates (Rana) spectabilis* | LC | Mexico | Frías-Alvarez et al. 2008; D.E. Green, unpub. |
| Ranidae | *Lithobates sphenocephalus (Rana sphenocephala)* | LC | United States | Mitchell and Green 2002; Daszak et al. 2005; Drake et al. 2007; Timpe et al. 2008; Venesky and Brem 2008; Rothermel et al. 2008; Hill et al. 2011; Lannoo et al. 2011; Muelleman and Montgomery 2013; Hanlon et al. 2014 Tennessee; Talley et al. 2015; Hill and Levy 2014; Phillips et al. 2014; Wimsatt et al. 2014; Battaglin et al. 2016; Love et al. 2016; Petersen et al. 2016; Watters et al. 2016, 2018, 2019; Chiari et al. 2017; Marhanka et al. 2017; Tupper et al. 2017; Bakland 2018; Brannelly et al. 2018 BMC Ecol; Marshall et al. 2019; Rivera et al. 2019; Unpub. data from: D.E. Green; Tulsa Zoo, (cap.); J. Ware and K. Duncan |
| Ranidae | *Lithobates (Rana) subaquavocalis^[[28]](#footnote-28)^* | CR | United States | Sredl et al. 2002; Arizona Game & Fish Dept, unpub. |
| Ranidae | *Lithobates sylvaticus (Rana sylvatica)* | LC | Canada, United States | Rittmann et al. 2003; Muths et al. 2008; Green and Muths 2005; Ouellet et al. 2005; Longcore et al. 2007; Reeves and Green 2006; Young et al. 2007; Reeves 2008; Rothermel et al. 2008, 2016; Zellmer et al. 2008; Chatfield et al. 2009; Rodriguez et al. 2009; Schock et al. 2009; Slough 2009; Forzán et al. 2010; Gaudreau et al. 2010; Sadinski et al. 2010; Davidson and Chambers 2011; Krynak et al. 2012; Stevens et al. 2012; Wolff et al. 2012; Richards-Hrdlicka et al. 2013; Rollins et al. 2013; Lenker et al. 2014; Richardson et al. 2014; Ross et al. 2014; D’Aoust-Messier et al. 2015; Petersen et al. 2016; Olori et al. 2018; Robinson et al. 2018; Talbott et al. 2018; Jongsma et al. 2019; Unpub. data from: D.E. Green; J. Longcore; A. Pessier and J. Mendelson (cap.) |
| Ranidae | *Lithobates (Rana) tarahumarae* | VU | Mexico, United States | Hale et al. 2005; Arizona Game & Fish Dept, unpub.; T. Jones, unpub. |
| Ranidae | *Lithobates vaillanti* | LC | Belize, Colombia, Honduras | Kaiser and Pollinger 2012; Gutsche et al. 2015; Flechas et al. 2017 |
| Ranidae | *Lithobates vibicarius (Rana vibicaria)* | VU | Costa Rica | Puschendorf et al. 2006a, 2009; Whitfield et al. 2017 |
| Ranidae | *Lithobates (Rana) warszewitschii* | LC | Costa Rica, Panama | Lips et al. 2006; Brem and Lips 2008; Zumbado-Ulate et al. 2019 |
| Ranidae | *Lithobates (Rana) yavapaiensis* | LC | Mexico, United States | Bradley et al. 2002; Rosen and Schwalbe 2002; Sredl et al. 2002; Morehouse et al. 2003; Hale et al. 2005; Schlaepfer et al. 2007; Forrest and Schlaepfer 2011; Savage et al. 2011 Biol Cons; Velo-Antón et al. 2012; Unpub. data from: Arizona Game & Fish Dept.; D.E. Green; T. Jones |
| Ranidae | *Odorrana andersonii* | LC | China | Bai et al. 2010 |
| Ranidae | *Odorrana chloronota* | LC | Laos | Swei et al. 2011 |
| Ranidae | *Odorrana grahami* | NT | China | Zhu et al. 2014 |
| Ranidae | *Odorrana hosii* | LC | Indonesia | Swei et al. 2011 |
| Ranidae | *Odorrana margaretae* | LC | China | Zhu et al. 2014 |
| Ranidae | *Odorrana (Rana) narina (*briefly *Eburana)* | EN | Japan | Goka et al. 2009 |
| Ranidae | *Odorrana schmakeri* | LC | China | Zhu et al. 2014 |
| Ranidae | *Pelophylax bedriagae* | LC | Turkey | Göçmen et al. 2013 |
| Ranidae | *Pelophylax caralitanus* | NT | Turkey | Erismis et al. 2014 |
| Ranidae | *Pelophylax chosenicus* | VU | South Korea | Bataille et al. 2013 |
| Ranidae | *Pelophylax epeiroticus* | VU | Greece | Azmanis et al. 2016 |
| Ranidae | *Pelophylax* kl. *esculentus (Rana esculenta)* | LC | Austria, Czech Republic, Denmark, Germany, Hungary, Italy, Luxembourg, The Netherlands, Poland, Serbia, Switzerland | Adams et al. 2008; Federici et al. 2008; Scalera et al. 2008; Wood et al. 2009; Czeczuga et al. 2011; Baláž et al. 2014 Cons Biol; Spitzen-van der Sluijs et al. 2014; Kolenda et al. 2017; Mali et al. 2017; Vörös et al. 2018; A. Hettyey, unpub; U. Reyer, unpub. |
| Ranidae | *Pelophylax (Rana) lessonae* | LC | Austria, Italy, The Netherlands, Poland, Serbia, Switzerland, United Kingdom | Garner et al. 2005; Simoncelli et al. 2005; Di Rosa et al. 2007; Cunningham and Minting 2008; Czeczuga et al. 2011; Sztatecsny and Glaser 2011; Spitzen-van der Sluijs et al. 2014; Kolenda et al. 2017, Mali et al. 2017 |
| Ranidae | *Pelophylax nigromaculatus (Rana nigromaculata)* | NT | China, Japan, South Korea | Goka et al. 2009; Bai et al. 2012; Bataille et al. 2013; Zhu et al. 2014, 2016 |
| Ranidae | *Pelophylax (Rana) perezi* | LC | Portugal, Spain | Hidalgo-Vila et al. 2012; Rosa et al. 2017; Oficialdegui et al. 2017; S. Walker, unpub. |
| Ranidae | *Pelophylax porosus porosus (Rana porosa porosa)* | LC | Japan | Goka et al. 2009 |
| Ranidae | *Pelophylax ridibundus (Rana ridibunda)* | LC | Greece, Hungary, Serbia, Switzerland, Turkey | Garner et al. 2005; Erismis et al. 2014; Azmanis et al. 2016; Mali et al. 2017; Vörös et al. 2018 |
| Ranidae | *Pelophylax saharicus* | LC | Morocco | El Cadi et al. 2019 |
| Ranidae | *Pelophylax* sp.^[[29]](#footnote-29)^ | — | North Macedonia | Vojar et al. 2017 |
| Ranidae | *Pulchrana glandulosa* | LC | Malaysia | Savage et al. 2011 EcoHealth |
| Ranidae | *Pulchrana laterimaculata* | LC | Singapore | Chong et al. 2018 |
| Ranidae | *Puchrana piturata* | LC | Malaysia | Savage et al. 2011 EcoHealth |
| Ranidae | *Pulchrana (Hylarana, Rana) similis* | LC | Philippines | Swei et al. 2011; Smith et al. 2019; A. Diesmos and R. Brown, unpub.^6^ |
| Ranidae | *Rana arvalis* | LC | Germany, Sweden | Mutschmann et al. 2000; Ohst et al. 2013; Kärvemo et al. 2018 |
| Ranidae | *Rana aurora* and *R.a. aurora* | LC | Canada, United States | Pearl et al. 2007; Adams et al. 2007, 2010; Richardson et al. 2014; Ecoclub Amphibian Group et al. 2016; Petersen et al. 2016; D.E. Green, unpub.; N. Nieto, unpub. |
| Ranidae | *Rana boylii* | NT | United States | Lowe 2009; MJ Adams et al. 2010; Gaulke et al. 2011; Ecoclub Amphibian Group et al. 2016; AJ Adams et al. 2017 Ecol Evol, 2017 Ecosphere; Unpub. data from: T. James; P. Johnson; S. Kupferberg; N. Nieto; G. Padgett-Flohr; K. Pope; S. Wagner and J. Johnson |
| Ranidae | *Rana cascadae* | NT | United States | Adams et al. 2010; Piovia-Scott et al. 2011; Roth et al. 2013; De Léon et al. 2017; K. Pope, unpub.; S. Wagner and J. Johnson, unpub. |
| Ranidae | *Rana chaochiaoensis* | LC | China | Bai et al. 2010 |
| Ranidae | *Rana chensinensis* | LC | China | Zhu et al. 2014 |
| Ranidae | *Rana coreana* | LC | South Korea | Bataille et al. 2013 |
| Ranidae | *Rana dalmatina* | LC | Germany | Rasmussen et al. 2012 |
| Ranidae | *Rana draytonii* | VU | Mexico, United States  (US Threatened) | Morgan et al. 2007; MJ Adams et al. 2010; Tatarian and Tatarian 2010; Fellers et al. 2011; AJ Adams et al. 2017 Ecol Evol, 2017 Ecosphere; Peralta-García et al. 2018; Santos Barrera and Peralta-García 2018; Unpub. data from: R. Fisher; V. Hemingway; T. James; P. Johnson; G. Padgett-Flohr |
| Ranidae | *Rana dybowskii* | LC | South Korea | Bataille et al. 2013 |
| Ranidae | *Rana huanrenesis* | LC | South Korea | Bataille et al. 2013 |
| Ranidae | *Rana iberica* | NT | Spain | S. Walker, unpub. |
| Ranidae | *Rana italica* | LC | Italy | Zampiglia et al. 2013 |
| Ranidae | *Rana latastei* | VU | Italy | Garner et al. 2005 |
| Ranidae | *Rana luteiventris* | LC | Canada, United States | Muths et al. 2003, 2008; Pearl et al. 2007; Adams et al. 2010; Russell et al. 2010; Gaulke et al. 2011; Stevens et al. 2012; Richardson et al. 2014; Araos et al. 2017; Unpub. data from: E Bull; C. Goldberg and L. Watts; J. Engle; D.E. Green; J. Lowe; B. Maxell; D. Pilliod and E. Muths |
| Ranidae | *Rana muscosa* | EN | United States | Fellers et al. 2001; Morehouse et al. 2003; Rachowicz et al. 2006; Morgan et al. 2007; Woodhams et al. 2007; Vredenburg et al. 2010; Smith et al. 2017; Unpub. data from: R. Fisher; D.E. Green; R. Knapp; D. McGriff; S. Muskopf; G. Padgett-Flohr; A. Pessier and J. Mendelson (cap.) |
| Ranidae | *Rana omeimontis* | LC | China | Zhu et al. 2014 |
| Ranidae | *Rana ornativentris* | LC | Japan (cap.) | Goka et al. 2009 |
| Ranidae | *Rana pretiosa* | VU | Canada, United States | Pearl et al. 2007, 2009; Hayes et al. 2009; Adams et al. 2010; Richardson et al. 2014; D.E. Green, unpub.; J. Lowe, unpub. |
| Ranidae | *Rana pyrenaica* | EN | Spain | S. Walker, unpub. |
| Ranidae | *Rana sierrae* | EN | United States | Briggs et al. 2010; Morgan et al. 2007; Vredenburg et al. 2010; Fellers et al. 2011; Smith et al. 2017; Brown et al. 2019; R. Knapp, unpub. |
| Ranidae | *Rana tagoi* | LC | Japan (cap.) | S. Okada, unpub. |
| Ranidae | *Rana temporaria* | LC | Austria, Denmark, France, Germany, Hungary, The Netherlands, Poland, Romania, Spain, Sweden, United Kingdom | Garner et al. 2005; Arai 2008; Cunningham and Minting 2008; Scalera et al. 2008; Czeczuga et al. 2011; Sztatecsny and Glaser 2011; Rasmussen et al. 2012; Ohst et al. 2013; Vörös et al. 2013; Baláž et al. 2014; Spitzen-van der Sluijs et al. 2014; Kärvemo et al. 2018, 2019; S. Walker, unpub. |
| Ranidae | *Rana zhenhaiensis* | LC | China | Bai et al. 2012 |
| Ranidae | *Sanguirana (Hylarana, Rana) luzonensis* | NT | Philippines | Swei et al. 2011; A. Diesmos and R. Brown, unpub.^6^ |
| Ranidae | *Sylvirana (Hylarana, Rana) faber* | LC | Cambodia | Mendoza et al. 2011 |
| Ranidae | *Sylvirana guentheri* | LC | Singapore | Chong et al. 2018 |
| **Ranixalidae** | *Indirana brachytarsus* | EN | India | Nair et al. 2011 |
| Ranixalidae | *Indirana leithii* | VU | India | Nair et al. 2011; Molur et al. 2015 |
| **Rhacophoridae** | *Buergeria buergeri* | LC | Japan (cap.) | S. Okada, unpub. (cap.) |
| Rhacophoridae | *Buergeria japonica* | LC | Japan | Goka et al. 2009 |
| Rhacophoridae | *Chiromantis rufescens* | LC | Cameroon, Gabon, Nigeria | Bell et al. 2011; Imasuen et al. 2011; Hirschfeld et al. 2016; Jongsma et al. 2016 |
| Rhacophoridae | *Kurixalus (Rhacophorus) bisacculus* | LC | Cambodia | Mendoza et al. 2011 |
| Rhacophoridae | *Kurixalus verrucosus* | LC | Japan (cap.) | Tamukai et al. 2014 (cap.) |
| Rhacophoridae | *Nyctixalus pictus* | NT | Japan (cap.) | Tamukai et al. 2014 (cap.) |
| Rhacophoridae | *Philautus cardamonus* | DD | Cambodia | Mendoza et al. 2011 |
| Rhacophoridae | *Philautus petersi* | LC | Malaysia | Savage et al. 2011 EcoHealth |
| Rhacophoridae | *Philautus* sp. 1 (Laos)^[[30]](#footnote-30)^ | — | Laos | Swei et al. 2011 |
| Rhacophoridae | *Philautus vermiculatus* | LC | Malaysia | Savage et al. 2011 EcoHealth |
| Rhacophoridae | *Polypedates leucomystax* | LC | Cambodia, Singapore | Mendoza et al. 2011; Gaertner et al. 2011; Gilbert et al. 2013 |
| Rhacophoridae | *Pseudophilautus alto* | EN | Sri Lanka | Swei et al. 2011 |
| Rhacophoridae | *Pseudophilautus amboli* | CR | India | Molur et al. 2015 |
| Rhacophoridae | *Pseudophilautus silus* | EN | Sri Lanka | Swei et al. 2011 |
| Rhacophoridae | *Pseudophilautus sordidus* | NT | Sri Lanka | Swei et al. 2011 |
| Rhacophoridae | *Raorchestes beddomii* | NT | India | Molur et al. 2015 |
| Rhacophoridae | *Raorchestes bombayensis* | VU | India | Dahanukar et al. 2013 |
| Rhacophoridae | *Raorchestes ghatei* | NE | India | Molur et al. 2015 |
| Rhacophoridae | *Raorchestes gryllus* | DD | Vietnam | Swei et al. 2011 |
| Rhacophoridae | *Rhacophorus bipunctatus* | LC | Cambodia | Mendoza et al. 2011 |
| Rhacophoridae | *Rhacophorus margaritifer (javanus)* | LC | Indonesia | Kusrini et al. 2008 |
| Rhacophoridae | *Taruga eques* | EN | Sri Lanka | Swei et al. 2011 |
| Rhacophoridae | *Theloderma asperum* | LC | Japan (cap.) | Tamukai et al. 2014 (cap.) |
| Rhacophoridae | *Theloderma bicolor* | EN | Czech Republic (cap.), Japan (cap.) | Tamukai et al. 2014 (cap.); Havlíková et al. 2015 (cap.) |
| Rhacophoridae | *Theloderma stellatum* | LC | Czech Republic (cap.) | Havlíková et al. 2015 (cap.) |
| Rhacophoridae | *Zhangixalus dennysi* | LC | China | Zhu et al. 2014 |
| Rhacophoridae | *Zhangixalus (Rhacophorus) viridis viridis* | LC | Japan (cap.) | S. Okada, unpub. (cap.) |
| **Rhinodermatidae** | *Rhinoderma darwinii* | EN | Chile, Uruguay | Bourke et al. 2010, 2011; Bardier et al. 2011; Soto-Azat et al. 2013 |
| Rhinodermatidae | *Rhinoderma rufum* | CR | Chile | Soto-Azat et al. 2013 |
| **Scaphiopodidae** | *Scaphiopus couchii* | LC | United States | Suriyamongkol et al. 2019 |
| Scaphiopodidae | *Scaphiopus holbrookii* | LC | United States | Tupper et al. 2011, 2014; A. Pessier and J. Mendelson, unpub. (cap.) |
| Scaphiopodidae | *Spea bombifrons* | LC | United States | Suriyamongkol et al. 2019 |
| Scaphiopodidae | *Spea intermontana* | LC | Canada | Richardson et al. 2014 |
| **Telmatobiidae** | *Telmatobius atacamensis* | CR | Argentina | Barrionuevo and Mangione 2006 |
| Telmatobiidae | *Telmatobius bolivianus* | NT | Bolivia | Burrowes and de la Riva 2017 |
| Telmatobiidae | *Telmatobius culeus* | EN | Bolivia, Peru | Berenguel et al. 2016; Burrowes and de la Riva 2017 ; Burrowes et al. 2020 |
| Telmatobiidae | *Telmatobius edaphonastes* | EN | Bolivia | Burrowes and de la Riva 2017 |
| Telmatobiidae | *Telmatobius espadai* | CR | Bolivia | Burrowes and de la Riva 2017 |
| Telmatobiidae | *Telmatobius gigas* | CR | Bolivia | de la Riva and Burrowes 2011; Burrowes and de la Riva 2017 |
| Telmatobiidae | *Telmatobius hintoni* | VU | Bolivia | Burrowes and de la Riva 2017 |
| Telmatobiidae | *Telmatobius huayra* | VU | Bolivia | Burrowes and de la Riva 2017 |
| Telmatobiidae | *Telmatobius intermedius* | EN | Peru | Rubio et al. 2018 |
| Telmatobiidae | *Telmatobius jelskii* | NT | Peru | Catenazzi et al. 2013 |
| Telmatobiidae | *Telmatobius marmoratus* | VU | Argentina, Bolivia, Chile, Peru | Seimon et al. 2007; Catenazzi et al. 2010, 2011; Ghirardi 2011; Kosch et al. 2012; Cossell et al. 2014; Solís et al. 2015; Warne et al. 2016; Burrowes and de la Riva 2017; Seimon et al. 2017; Rubio et al. 2018; I. De la Riva, unpub.; R. Solís, unpub. |
| Telmatobiidae | *Telmatobius niger* | CR | Ecuador | Berger et al. 1999; Ron and Merino-Viteri 2000 |
| Telmatobiidae | *Telmatobius peruvianus* | VU | Bolivia | Solís et al. 2015 |
| Telmatobiidae | *Telmatobius pisanoi* | EN | Argentina | Barrionuevo and Mangione 2006 |
| Telmatobiidae | *Telmatobius rubigo* | VU | Bolivia | Burrowes and de la Riva 2017 |
| Telmatobiidae | *Telmatobius sanborni* | VU | Bolivia | Burrowes and de la Riva 2017 |
| Telmatobiidae | *Telmatobius sibiricus* | EN | Bolivia | Burrowes and de la Riva 2017 |
| Telmatobiidae | *Telmatobius simonsi* | NT | Bolivia | Burrowes and de la Riva 2017 |
| Telmatobiidae | *Telmatobius timens* | CR | Bolivia |  |
| Telmatobiidae | *Telmatobius verrucosus* | VU | Bolivia |  |
| Telmatobiidae | *Telmatobius yuracare* | VU | Bolivia |  |

**Part 2. Caudata**

| **Family** | **Species** | **Status** | **Country** | **Reference** |
| --- | --- | --- | --- | --- |
| **Ambystomatidae** | *Ambystoma altamirani* | EN | Mexico | Frías-Alvarez et al. 2008 |
| Ambystomatidae | *Ambystoma andersoni* | CR | Mexico, United Kingdom (cap.) | Michaels et al. 2018 (cap.); Basanta et al. 2019 |
| Ambystomatidae | *Ambystoma annulatum* | LC | United States | Watters et al. 2018 |
| Ambystomatidae | *Ambystoma californiense* | VU | United States | Padgett-Flohr and Longcore 2005 |
| Ambystomatidae | *Ambystoma dumerilii* | CR | France (cap.), Mexico (cap.), United Kingdom (cap.) | Michaels et al. 2018 (cap.); Basanta et al. 2019 (cap.) |
| Ambystomatidae | *Ambystoma flavipiperatum* | EN | Mexico | Basanta et al. 2019 |
| Ambystomatidae | *Ambystoma gracile* | LC | United States | S. Wagner and J. Johnson, unpub. |
| Ambystomatidae | *Ambystoma granulosum* | CR | Mexico | Frías-Alvarez et al. 2008 |
| Ambystomatidae | *Ambystoma jeffersonianum* | LC | United States | Brodman and Briggler 2008; Krynak et al. 2012; Lenker et al. 2014 |
| Ambystomatidae | *Ambystoma macrodactylum* | LC | Canada, United States | Stevens et al. 2012; Unpub. data from: S. Wagner and J. Johnson; D.E. Green; C. Goldberg and L. Watts |
| Ambystomatidae | *Ambystoma maculatum* | LC | Canada, United States | Ouellet et al. 2005; Krynak et al. 2012; Richards-Hrdlicka et al. 2013; Lenker et al. 2014; Moffitt et al. 2015; Olori et al. 2018; Robinson et al. 2018; A. Pessier and J. Mendelson, unpub. (cap.) |
| Ambystomatidae | *Ambystoma mexicanum* (cap.) | CR | Australia (cap.), Mexico (cap.), United Kingdom (cap.) | Berger et al. 1999 (cap.); Velo-Antón et al. 2012 (cap.); Galindo-Bustos et al. 2014 (cap.); Michaels et al. 2018 (cap.) |
| Ambystomatidae | *Ambystoma opacum* | LC | Japan (cap.), United States | Kinney et al. 2011; Tamukai et al. (cap.); Love et al. 2016; A. Pessier and J. Mendelson, unpub. (cap.) |
| Ambystomatidae | *Ambystoma rivulare* | EN | Mexico | Frías-Alvarez et al. 2008; Basanta et al. 2019 |
| Ambystomatidae | *Ambystoma talpoideum* | LC | United States | Love et al. 2016; A. Pessier and J. Mendelson, unpub. (cap.) |
| Ambystomatidae | *Ambystoma texanum* | LC | United States | Phillips et al. 2014; Marhanka et al. 2017; Watters et al. 2018 |
| Ambystomatidae | *Ambystoma tigrinum* | LC | Canada, Japan (cap.), United States | Sredl et al. 2002; Davidson et al. 2003; Morehouse et al. 2003; Muths et al. 2008; Tamukai et al. 2014 (cap.); Love et al. 2016; Unpub. data from: Canadian Cooperative Wildlife Health Centre; D.E. Green; B. Maxell |
| Ambystomatidae | *Ambystoma velasci* | LC | Mexico | Frías-Alvarez et al. 2008 |
| Ambystomatidae^[[31]](#footnote-31)^ | *Dicamptodon aterrimus* | LC | United States | Hossack et al. 2010; D.E. Green, unpub. |
| Ambystomatidae^11^ | *Dicamptodon tenebrosus* | LC | United States | S. Kupferberg, unpub. |
| **Amphiumidae** | *Amphiuma means* | LC | United States | Chatfield et al. 2012 |
| Amphiumidae | *Amphiuma tridactylum* | LC | United States | Chatfield et al. 2012 |
| **Cryptobranchidae** | *Andrias davidianus* | CR | China | Zhu et al. 2014, 2016 |
| Cryptobranchidae | *Andrias japonicus* | NT | Japan, United States (cap.) | A. Pessier and J. Mendelson, unpub. (cap.) |
| Cryptobranchidae | *Cryptobranchus alleganiensis alleganiensis* | NT | United States | Briggler et al. 2008; Burgmeier et al. 2011; Gonyor et al. 2011; Regester et al. 2012; Souza et al. 2012; Tominaga et al. 2013; Eskew et al. 2014; Williams and Groves 2014; Bales et al. 2015; Seeley et al. 2016; G. Lipps, unpub.; A. Pessier and J. Mendelson, unpub. (cap.) |
| Cryptobranchidae | *Cryptobranchus alleganiensis bishopi* | NT | United States | Briggler et al. 2008; Bodinof et al. 2011; Tominaga et al. 2013; A. Pessier and J. Mendelson, unpub. (cap.) |
| **Hynobiidae** | *Hynobius leechii* | LC | South Korea | Bataille et al. 2013 |
| Hynobiidae | *Hynobius quelpaertensis* | DD | South Korea | Bataille et al. 2013 |
| Hynobiidae | *Liua shihi* | LC | China | Zhu et al. 2014 |
| **Plethodontidae** | *Aneides aeneas* | NT | United States | Blackburn et al. 2015; Moffitt et al. 2015; Bauer et al. 2018; Newman et al. 2019 |
| Plethodontidae | *Aneides lugubris* | LC | United States (cap.) | A. Pessier and J. Mendelson, unpub. (cap.) |
| Plethodontidae | *Aquiloeurycea (Pseudoeurycea) cephalica* | NT | Mexico | Van Rooij et al. 2011 |
| Plethodontidae | *Batrachoseps attenuatus* | LC | United States | Weinstein 2009; Sette et al. 2015 |
| Plethodontidae | *Batrachoseps gavilanensis* | LC | United States | Weinstein 2009 |
| Plethodontidae | *Batrachoseps nigriventris* | LC | United States | Weinstein 2009; A. Pessier and J. Mendelson, unpub. (cap.) |
| Plethodontidae | *Batrachoseps relictus* | DD | United States | Weinstein 2009 |
| Plethodontidae | *Batrachoseps wrighti* | VU | United States | Weinstein 2009 |
| Plethodontidae | *Bolitoglossa colonnea* | LC | Costa Rica, Panama | Lips et al. 2006; Zumbado-Ulate et al. 2019 |
| Plethodontidae | *Bolitoglossa conanti* | EN | United States (cap.) | A Pessier and J. Mendelson, unpub. |
| Plethodontidae | *Bolitogolossa doffleini* | NT | Belgium (cap.) | Pasmans et al. 2004 (cap.) |
| Plethodontidae | *Bolitoglossa engelhardti* | EN | Guatemala | Cheng et al. 2011 |
| Plethodontidae | *Bolitoglossa flavimembris* | EN | Guatemala | Cheng et al. 2011 |
| Plethodontidae | *Bolitoglossa franklini x lincolni* |  | Guatemala | Rovito et al. 2009; Cheng et al. 2013 |
| Plethodontidae | *Bolitoglossa mombachoensis* | VU | Nicaragua | Stark et al. 2017 |
| Plethodontidae | *Bolitoglossa leandrae* | NE | Colombia | Acevedo et al. 2016; Flechas et al. 2017 |
| Plethodontidae | *Bolitoglossa lincolni* | NT | Guatemala | Rovito et al. 2009 |
| Plethodontidae | *Bolitoglossa mombachoensis* | VU | Nicaragua | Stark et al. 2017 |
| Plethodontidae | *Bolitoglossa occidentalis* | LC | Guatemala | Rovito et al. 2009; Cheng et al. 2013 |
| Plethodontidae | *Bolitoglossa robusta* | LC | Costa Rica | Cheng et al. 2011 |
| Plethodontidae | *Bolitoglossa rufescens* | LC | Mexico; United States (cap.) | Van Rooij et al. 2011; A. Pessier and J. Mendelson unpub. (cap.) |
| Plethodontidae | *Bolitoglossa schizodactyla* | LC | Panama | Lips et al. 2006 |
| Plethodontidae | *Bolitoglossa tamaense* | NE | Colombia | Acevedo et al. 2016; Flechas et al. 2017 |
| Plethodontidae | *Chiropterotriton dimidiatus* | EN | Mexico | Cheng et al. 2011 |
| Plethodontidae | *Chiropterotriton multidentatus* | EN | Mexico | Cheng et al. 2011 |
| Plethodontidae | *Dendrotriton bromeliacius* | CR | Guatemala | Rovito et al. 2009; Cheng et al. 2011 |
| Plethodontidae | *Dendrotriton xolocalcae* | VU | Mexico | Velo-Antón et al. 2012 |
| Plethodontidae | *Desmognathus conanti* | NE | United States | Timpe et al. 2008; Hill et al. 2011; Chiari et al. 2017 |
| Plethodontidae | *Desmognathus fuscus* | LC | Canada, Japan (cap.), United States | Campbell Grant et al. 2008; Hossack et al. 2010; Davidson and Chambers 2011; Lannoo et al. 2011; Krynak et al. 2012; Richards-Hrdlicka et al. 2013; Tamukai et al. 2014 (cap.); Brocco 2017; Spaulding et al. 2018; Jongsma et al. 2019 |
| Plethodontidae | *Desmognathus monticola* | LC | United States | Hossack et al. 2010; Davidson and Chambers 2011; Gratwicke et al. 2011 Virginia; Hill et al. 2011; Spaulding et al. 2018 |
| Plethodontidae | *Desmognathus ochrophaeus* | LC | United States | Davidson and Chambers 2011; Krynak et al. 2012 |
| Plethodontidae | *Desmognathus ocoee* | LC | United States | Kiemnec-Tyburczy et al. 2012; Rothermel et al. 2013 |
| Plethodontidae | *Desmognathus quadramaculatus* | LC | United States | Bartkus 2009; Davidson and Chambers 2011; Rothermel et al. 2013 |
| Plethodontidae | *Desmognathus santeetlah* | LC | United States | Caruso and Lips 2013 |
| Plethodontidae | *Desomognathus* sp. | — | United Kingdom (cap.) | Wombwell et al. 2016 |
| Plethodontidae | *Eurycea bislineata* | LC | United States | Campbell Grant et al. 2008; Krynak et al. 2012; Richards-Hrdlicka et al. 2013; Windstam and Olori 2014; Augustine and Neff 2016; Bauer et al. 2018; Olori et al. 2018; Robinson et al. 2018; D.E. Green, unpub. |
| Plethodontidae | *Eurycea chisholmensis* | VU | United States (cap.) | A Pessier and J Mendelson, unpub. (cap.) |
| Plethodontidae | *Eurycea cirrigera* | LC | United States | Byrne et al. 2008; Davidson and Chambers 2011; Hill et al. 2011; Lannoo et al. 2011; Brocco 2017; Chiari et al. 2017; Spaulding et al. 2018 |
| Plethodontidae | *Eurycea guttolineata* | LC | United States | Chiari et al. 2017 |
| Plethodontidae | *Eurycea longicauda* | LC | United States | Rimer and Briggler 2010; Davidson and Chambers 2011; Lannoo et al. 2011; Muelleman and Montgomery 2013; Bauer et al. 2018 (cap.); Watters et al. 2018 |
| Plethodontidae | *Eurycea lucifuga* | LC | United States | Davidson and Chambers 2011; Mowry et al. 2017; Watters et al. 2018; A Pessier and J Mendelson, unpub. (cap.) |
| Plethodontidae | *Eurycea multiplicata* | LC | United States | Watters et al. 2018 |
| Plethodontidae | *Eurycea nana* | VU | United States | Gaertner et al. 2007, 2009a |
| Plethodontidae | *Eurycea neotenes* | VU | United States | Gaertner et al. 2009a |
| Plethodontidae | *Eurycea pterophila* | DD | United States | Gaertner et al. 2009a |
| Plethodontidae | *Eurycea quadridigitata* | LC | United States | Saenz et al. 2010 |
| Plethodontidae | *Eurycea rathbuni* | VU | United States | Gluesenkamp et al. 2018 |
| Plethodontidae | *Eurycea sosorum* | VU | United States | Gaertner et al. 2007, 2009a; A Pessier and J Mendelson, unpub. (cap.) |
| Plethodontidae | *Eurycea spelaea* | LC | United States | Rimer and Briggler 2010 |
| Plethodontidae | *Eurycea tonkawae* | EN | United States | Gaertner et al. 2007, 2009a; A Pessier and J Mendelson, unpub. (cap.) |
| Plethodontidae | *Eurycea tynerensis* | NT | United States | Watters et al. 2018 |
| Plethodontidae | *Eurycea waterlooensis* | VU | United States (cap.) | A Pessier and J Mendelson, unpub. (cap.) |
| Plethodontidae | *Gyrinophilus porphyriticus* | LC | United States | Richards-Hrdlicka et al. 2013; Robinson et al. 2018 |
| Plethodontidae | *Hemidactylium scutatum* | LC | United States | Richards-Hrdlicka et al. 2013 |
| Plethodontidae | *Karsenia koreana* | LC | South Korea | Bataille et al. 2013 |
| Plethodontidae | *Oedipina collaris* | DD | Panama | Lips et al. 2006 |
| Plethodontidae | *Oedipina grandis* | EN | Costa Rica | Lips et al. 2003 |
| Plethodontidae | *Oedipina poelzi* | EN | Costa Rica | Cheng et al. 2011 |
| Plethodontidae | *Oedipina uniformis* | NT | Costa Rica | Cheng et al. 2011 |
| Plethodontidae | *Plethodon albagula* | LC | United States | Watters et al. 2018 |
| Plethodontidae | *Plethodon augusticlavius* | LC | United States | Watters et al. 2018 |
| Plethodontidae | *Plethodon cinereus* | LC | Canada, United States | Lauer et al. 2007; Davidson and Chambers 2011; Krynak et al. 2012; Richards-Hrdlicka et al. 2013; Lenker et al. 2014; Robinson et al. 2018; Jongsma et al. 2019; Muletz-Wolz et al. 2019 |
| Plethodontidae | *Plethodon glutinosus* | LC | United States | Chinnadurai et al. 2009; Davidson and Chambers 2011 |
| Plethodontidae | *Plethodon neomexicanus* | NT | United States | Cummer et al. 2005 |
| Plethodontidae | *Plethodon shermani* | VU | United States | Kiemnec-Tyburczy et al. 2012 |
| Plethodontidae | *Plethodon yohahlossee* | LC | United States | Chinnadurai et al. 2009 |
| Plethodontidae | *Pseudoeurycea firscheini* | EN | Mexico | Van Rooij et al. 2011 |
| Plethodontidae | *Pseudoeurycea leprosa* | LC | Mexico | Van Rooij et al. 2011; Mendoza-Almeralla et al. 2016 |
| Plethodontidae | *Pseudoeurycea nigromaculata* | EN | Mexico | Cheng et al. 2011 |
| Plethodontidae | *Pseudoeurycea smithi* | CR | Mexico | Cheng et al. 2011 |
| Plethodontidae | *Pseudotriton ruber* | LC | United States | Speare and Berger 2005; Montanucci 2009; Hill et al. 2011; Lannoo et al. 2011; Krynak et al. 2012; Spaulding et al. 2018 |
| Plethodontidae | *Thorius pennatulus* | CR | Mexico | Cheng et al. 2011 |
| **Proteidae** | *Necturus alabamensis* | EN | United States | Chatfield et al. 2012; A. Pessier and J. Mendelson, unpub. (cap.) |
| Proteidae | *Necturus beyeri* | LC | United States | Brannelly et al. 2012; Chatfield et al. 2012; Glorioso et al. 2017 |
| Proteidae | *Necturus maculosus* and *N.m. maculosus* | LC | Japan (cap.), United States | Tamukai et al. 2014 (cap.); Regester et al. 2016; D.E. Green, unpub.; A. Pessier and J. Mendelson, unpub. (cap.) |
| Proteidae | *Necturus punctatus* | LC | United States (cap.) | A. Pessier and J. Mendelson, unpub. (cap.) |
| **Salamandridae** | *Calotriton (Euproctus) asper^[[32]](#footnote-32)^* | NT | Spain | S. Walker, unpub. |
| Salamandridae | *Cynops ensicauda* | EN | Czech Republic (cap.), Japan | Goka et al. 2009; Lastra González et al. 2019 (cap.); S. Okada, unpub. |
| Salamandridae | *Cynops pyrrhogaster* | LC | Japan | S. Okada, unpub. |
| Salamandridae | *Euproctus platycephalus* | EN | Italy | Bovero et al. 2008; Bielby et al. 2013; M.C. Fisher, unpub. |
| Salamandridae | *Hypselotriton orientalis* | LC | China (cap.) | Bai et al. 2012 (cap.) |
| Salamandridae | *Ichthyosaura (Triturus, Mesotriton) alpestris^[[33]](#footnote-33)^* and *I.a. apuanus* | LC | Austria, Belgium, Czech Republic, Germany, Hungary, Italy, Luxembourg, The Netherlands, Spain, Switzerland, United Kingdom | Garner et al. 2005; Cunningham and Minting 2008; Wood et al. 2009; Sztatecsny and Glaser 2011; Civiš et al. 2012; Rasmussen et al. 2012; Tobler et al. 2012; Ohst et al. 2013; Zampiglia et al. 2013; Spitzen-van der Sluijs et al. 2014; Vörös et al. 2018; S. Walker, unpub. |
| Salamandridae | *Lissotriton boscai* | LC | Portugal, Spain | Hidalgo-Vila et al. 2012; Rosa et al. 2017 |
| Salamandridae | *Lissotriton (Triturus) helveticus^[[34]](#footnote-34)^* | LC | France, Germany, Luxembourg, Spain, Switzerland, United Kingdom | Cunningham and Minting 2008; Wood et al. 2009; Tobler et al. 2012; Ohst et al. 2013; Lastra González et al. 2019; S. Walker, unpub. |
| Salamandridae | *Lissotriton (Triturus) vulgaris^[[35]](#footnote-35)^ (*and *L. vulgaris amplensis)* | LC | Austria, Germany, The Netherlands, Montenegro, Romania, United Kingdom | Garner et al. 2005; Cunningham and Minting 2008; Sztatecsny and Glaser 2011; Rasmussen et al. 2012;.Ohst et al. 2013; Vörös et al. 2013; Spitzen-van der Sluijs et al. 2014; Vojar et al. 2017; Lastra González et al. 2019 |
| Salamandridae | *Neurergus kaiseri* | VU | Czech Republic (cap.), Europe (cap.), United States (cap.) | Spitzen-van der Sluijs et al. 2011 (cap.); Havlíková et al. 2015 (cap.); A Pessier and J Mendelson, unpub. (cap.) |
| Salamandridae | *Notophthalmus peristriatus* | NT | United States | Hill and Levy 2014 |
| Salamandridae | *Notophthalmus viridescens*  *(N. v. viridescens; N. v. dorsalis; N. v. louisianensis)* | LC | Canada, United States | Ouellet et al. 2005; Rothermel et al. 2008, 2016; Zippel and Tabaka 2008; Chatfield et al. 2009; Bakkegard and Pessier 2010; Groner and Relyea 2010; Raffel et al. 2010; Davidson and Chambers 2011; Hill et al. 2011; Krynak et al. 2012; Wunder et al. 2012; Bletz and Harris 2013; Richards-Hrdlicka et al. 2013; Hughey et al. 2014; Lenker et al. 2014; Wimsatt et al. 2014; Patillo and Parris 2016; Marhanka et al. 2017; Olori et al. 2018; Robinson et al. 2018; Watters et al. 2018; Jongsma et al. 2019; Longo et al. 2019; Unpub. data from: D.E. Green; A. Pessier and J. Mendelson (cap.); Tulsa Zoo; M. Whitney |
| Salamandridae | *Pleurodeles waltl* | NT | Japan (cap.), Morocco, Portugal, Spain | Hidalgo-Vila et al. 2012; Tamukai et al. 2014 (cap.) Oficialdegui et al. 2019; Unpub. data from: J. Bosch; M.C. Fisher; S. Walker |
| Salamandridae | *Salamandra salamandra* and *S.s. gigliolii* | LC | France, Germany, Italy, Portugal, Spain | Bosch and Martinez-Solano 2006; Ohst et al. 2013; Zampiglia et al. 2013; Medina et al. 2015; Gabor et al. 2017; Rosa et al. 2017; Lötters et al. 2018; S. Walker, unpub. |
| Salamandridae | *Taricha granulosa* | LC | Canada, United States | Piovia-Scott et al. 2011; Richardson et al. 2011, 2014; Stutz et al. 2017; Johnson et al. 2018; S. Kupferberg, unpub. |
| Salamandridae | *Taricha torosa* | LC | United States | Padgett-Flohr and Longcore 2007; AJ Adams et al. 2017 Ecol Evol; Stutz et al. 2017; Johnson et al. 2018; R. Knapp, unpub. |
| Salamandridae | *Triturus carnifex* | LC | Austria, Italy | Sztatecsny and Glaser 2011, Grasselli et al. 2019 |
| Salamandridae | *Triturus cristatus* | LC | Austria, Germany, Montenegro | Sztatecsny and Glaser 2011; Ohst et al. 2013; Lastra González et al. 2019 |
| Salamandridae | *Triturus dobrogicus* | NT | Austria | Sztatecsny and Glaser 2011 |
| Salamandridae | *Triturus macedonicus* | NE | Albania | Vojar et al. 2017 |
| Salamandridae | *Triturus marmoratus* | LC | Portugal, Spain | Rosa et al. 2017; S. Walker, unpub. |
| Salamandridae | *Triturus pygmaeus* | NT | Spain | Hidalgo-Vila et al. 2012; Oficialdegui et al. 2019; S. Walker, unpub. |
| **Sirenidae** | *Pseudobranchus axanthus* | LC | United States | Chatfield et al. 2012 |
| Sirenidae | *Siren intermedia* | LC | United States | Talley et al. 2011; Brannelly et al. 2012; Chatfield et al. 2012 |
| Sirenidae | *Siren lacertina* | LC | Japan (cap.), United States | Chatfield et al. 2012; Tamukai et al. 2014 (cap.); A Pessier and J Mendelson, unpub. (cap.) |

**Part 3. Gymniophiona**

| **Family** | **Species** | **Status** | **Country** | **Reference** |
| --- | --- | --- | --- | --- |
| Dermophiidae | *Geotrypetes seraphini* | LC | Cameroon, United Kingdom (cap.) | Doherty-Bone et al. 2013; Gower et al. 2013; Rendle et al. 2015 |
| Dermophiidae | *Schistometopum thomense* | LC | São Tomé and Princípe | Hydeman et al. 2013 |
| Herpelidae | *Boulengerula uluguruensis* | LC | Tanzania | Gower et al. 2013 |
| Herpelidae | *Herpele squalostoma* | LC | Cameroon | Doherty-Bone et al. 2013; Gower et al. 2013 |
| Indotyphlidae | *Idiocranium* cf. *russeli* | DD | Cameroon | Gower et al. 2013 |
| Scolecomorphidae | *Crotaphatrema lamottei* | CR | Cameroon | Doherty-Bone et al. 2013; Gower et al. 2013; Hirschfeld et al. 2016 |
| Scolecomorphidae | *Scolecomorphus kirkii* | LC | Tanzania | Gower et al. 2013 |
| Siphonopidae | *Luetkenotyphlus brasiliensis* | DD | Brazil | Lambertini et al. 2017 |
| Siphonopidae | *Siphonops annulatus* | LC | Brazil | Lambertini et al. 2017 |
| Siphonopidae | *Siphonops paulensis* | LC | Brazil | Lambertini et al. 2017 |
| Typhlonectidae | *Chthonerpeton indistinctum* | LC | Uruguay | Lambertini et al. 2017 |
| Typhlonectidae | *Potamotyphlus kaupii* | LC | Brazil, French Guiana | Rendle et al. 2015; Lambertini et al. 2017 |
| Typhlonectidae | *Typhlonectes compressicauda* | LC | Brazil | Lambertini et al. 2017 |
| Typhlonectidae | *Typhlonectes natans* | LC | United States (cap.) | Churgin et al. 2013; A Pessier and J Mendelson, unpub. (cap.) |

# *Bd*-maps Data References

Acevedo AA, Martínez MP, Armesto LO, Florez LS, Pérez KS, Lizcano DJ (2016) Detection of *Batrachochytrium dendrobatidis* from northeastern Colombia. Herpetological Review 47(2): 220–226.

Adams AJ, Kupferberg SJ, Wilber MQ, Pessier AP, Grefsrud M, Bobzien S, Vredenburg VT, Briggs CJ (2017) Extreme drought, host density, sex, and bullfrogs influence fungal pathogen infection in a declining lotic amphibian. Ecosphere 8(3): e01740. doi: 10.1002/ecs2.1740

Adams AJ, Pessier AP, Briggs CJ (2017) Rapid extirpation of a North American frog coincides with an increase in fungal pathogen prevalence: Historical analysis and implications for reintroduction. Ecology and Evolution 7: 10216–10232. doi: 10.1002/ece3.3468

Adams MJ, Galvan S, Reinitz D, Cole RA, Pyare S, Hahr M, Govindarajulu P (2007) Incidence of the fungus *Batrachochytrium dendrobatidis* in amphibian populations along the northwest coast of North America. Herpetological Review 38: 430–431.

Adams MJ, Galvan S, Scalera R, Grieco C, Sindaco R (2008) *Batrachochytrium dendrobatidis* in amphibian populations in Italy. Herpetological Review 39: 324–326.

Adams MJ, Chelgren ND, Reinitz D, Cole RA, Rachowicz LJ, Galvan S, McCreary B, Pearl CA, Bailey LL, Bettaso J, Bull EL, Leu M (2010) Using occupancy models to understand the distribution of an amphibian pathogen, *Batrachochytrium dendrobatidis*. Ecological Applications 20: 289–302. https://doi.org/10.1890/08-2319.1

Addis BR, Lowe WH, Hossack BR, Allendorf FW (2015) Population genetic structure and disease in montane boreal toads: more heterozygous individuals are more likely to be infected with amphibian chytrid. Conservation Genetics 16: 833–844. doi: 10.1007/s10592-015-0704-6

Agostini MT, Burrowes PA (2015) Infection patterns of the chytrid fungus, *Batrachochytrium dendrobatidis*, on anuran assemblages in agro-ecosystems from Buenos Aires Province, Argentina. Phyllomedusa 14(2): 113–126. doi: http://dx.doi.org/10.11606/issn.2316-9079.v14i2p113-126.

Agostini MG, Cortelezzi A, Berkunsky I, Soler G, Burrowes P (2015) First record of *Batrachochytrium dendrobatidis* infecting threatened populations of Tandilean Red-belly Toad (*Melanophryniscus* aff. *montevidensis*) in Argentina. Revista Mexicana de Biodiversidad 86: 826–828. http://dx.doi.org/10.1016/j.rmb.2015.07.007

Alemu I JB, Cazabon MNE, Dempewolf L, Hailey A, Lehtinen RM, Mannette RP, Naranjit KT, Roach ACJ (2008) Presence of the chytrid fungus *Batrachochytrium dendrobatidis* in populations of the critically endangered frog *Mannophryne olmonae* in Tobago, West Indies. EcoHealth 5: 34–39.

Alemu I JB, Cazabon-Mannette MNE, Cunningham AA, Dempewolf L, Hailey A, Mannette RP, Naranjit KT, Perkins MW, Schmidt-Roach ACJ (2013) Presence of the chytrid fungus *Batrachochytrium dendrobatidis* in a Vulnerable frog in Trinidad, West Indies. Endangered Species Research 20: 131–136. doi: 10.3354/esr00485

Allain SJR, Goodman MJ (2017) Absence of chytrid fungus (*Batrachochytrium dendrobatidis*) in an introduced population of the common midwife toad (*Alytes obstetricans*) in Cambridge, UK. Herpetological Bulletin 142: 40–41.

Alminas OSV, Cook AA, Engilis A Jr (2010) Herpetofauna of the Lower Snake River habitat management units: results from the 2009 field season. A report to the US Army Corps of Engineers, Walla Walla District. Davis, CA: University of California, Davis, Museum of Wildlife and Fish Biology, Department of Wildlife, Fish, and Conservation Biology. https://www.researchgate.net/profile/Andrew_Jr/publication/277711744_Herpetofauna_of_the_Lower_Snake_River_Habitat_Management_Units_-_Results_from_the_2009_Field_Season_A_Report_to_the_US_Army_Corps_of_Engineers_-_Walla_Walla_District/links/5570bda208ae2f213c223856.pdf

Amorim FO, Pimentel LA, Machado LF, Cavalcanti ADC, Napoli MF, Juncá FA (2019) New records of *Batrachochytrium dendrobatidis* in the state of Bahia, Brazil: histological analysis in anuran amphibian collections. Diseases of Aquatic Organisms 136: 147–155. https://doi.org/10.3354/dao03402

An D, Waldman B (2016) Enhanced call effort in Japanese tree frogs infected by amphibian chytrid fungus. Biology Letters 12: 20160018. http://dx.doi.org/10.1098/rsbl.2016.0018

Arai S (2008) Investigation on the spread of chytridiomycosis between UK natterjack toads and other inland amphibian populations within Cumbria. MSc thesis. London: Imperial College. https://www.iccs.org.uk/wp-content/thesis/consci/2008/Arai.pdf

Araos HL, Kroft KL, Bogardus RM, Chang Y-M, Donohue KR, Hanley D, Hatch KA, Wilson KW (2017) The Columbia Spotted Frog (*Rana luteiventris*)—another species persisting with *Batrachochytrium dendrobatidis* infection. Herpetological Review 48(4): 782–786.

Arellano ML, Ferraro DP, Steciow MM, Lavilla EO (2009) Infection by the chytrid fungus *Batrachochytrium dendrobatidis* in the yellow belly frog (*Elachistocleis bicolor*) from Argentina. Herpetological Journal 19: 219–220.

Arellano ML, Akmentins MS, Velasco MA, Kass C, Kacoliris FP (2015) First report of *Batrachochytrium dendrobatidis* in *Atelognathus reverberii*, a threatened species in Argentina. Herpetological Review 46(3): 354–356.

Arellano ML, Velasco MA, Kacoliris FP, Belasen AM, James TY (2017) First record of *Batrachochytrium dendrobatidis* in *Pleurodema somuncurensis*, a critically endangered species from Argentina. Herpetological Review 48(1): 68–70.

Augustine L, Neff M (2016) Determining the prevalence of amphibian chytrid fungus (*Batrachochytrium dendrobatidis*) and *Ranavirus* at Long Branch Nature Center in Arlington, Virginia. Catesbeiana (Virginia Herpetological Society) 36(1): 35–42.

Aziz MNBA, Skerratt LF, McCallum H (2011) Dynamics of chytridiomycosis in a Tasmanian frog community. Herpetological Review 42(1): 53–57.

Azmanis PN, Strachinis I, Lymberakis P, Marschang RE (2016) First detection of the amphibian chytrid fungus (*Batrachochytrium dendrobatidis*) in free-living anuran populations in Greece. Journal of the Hellenic Veterinary Medical Society 67(4): 253–258.

Bacigalupe LD, Soto-Azat C, García-Vera C, Barría-Oyarzo I, Rezende EL (2017) Effects of amphibian phylogeny, climate and human impact on the occurrence of the amphibian-killing chytrid fungus. Global Change Biology 23: 3543–3553. doi: 10.1111/gcb.13610

Bai C, Garner TWJ, Li Y (2010) First evidence of *Batrachochytrium dendrobatidis* in China: Discovery of chytridiomycosis in introduced American bullfrogs and native amphibians in the Yunnan Province, China. EcoHealth 7: 127–134. doi: 10.1007/s10393-010-0307-0

Bai C, Liu X, Fisher MC, Garner TWJ, Li Y (2012) Global and endemic Asian lineages of the emerging pathogenic fungus *Batrachochytrium dendrobatidis* widely infect amphibians in China. Diversity and Distributions 18: 307–318. doi: 10.1111/j.1472-4642.2011.00878.x

Bakkegard KA, Pessier AP (2010) *Batrachochytrium dendrobatidis* in adult *Notophthalmus viridescens* in north-central Alabama, USA. Herpetological Review 41: 45–47.

Bakland P-EM (2018) Prevalence assessment of the amphibian chytrid fungus *Batrachochytrium dendrobatidis* across two habitat types in east Tennessee. MS Thesis. Chattanooga, TN: University of Tennessee at Chattanooga.

Baláž V, Kopecký O, Gvoždík V (2012) Presence of the amphibian chytrid pathogen confirmed in Cameroon. Herpetological Journal 22: 191–194. [Balaz V, Kopecky O, Gvozdik V]

Baláž V, Kubečová M, Civiš P, Rozínek R, Vojar J (2013) Fatal chytridiomycosis and infection loss observed in captive toads infected in the wild. Acta Vet Brno 82: 351–355. doi: 10.2754/avb201382040351 [Balaz V, Kubeckova M, Civis P, Rozinek R, Vojar J]

Baláž V, Vojar J, Civiš P, Šandera M, Rozínek R (2014a DAO) Chytridiomycosis risk among Central European amphibians based on surveillance data. Diseases of Aquatic Organisms 112: 1–8. doi: 10.3354/dao02799 [Balaz V, Vojar J, Civis P, Sandera M, Rozinek R]

Baláž V, Vörös J, Civiš P, Vojar J, Hettyey A, Só­s E, Dankovics R, Jehle R, Christiansen DG, Clare F, Fisher MC, Garner TWJ, Bielby J (2014b Cons Biol) Assessing risk and guidance on monitoring of *Batrachochytrium dendrobatidis* in Europe through identification of taxonomic selectivity of infection. Conservation Biology 28: 213–223. doi: 10.1111/cobi.12128 [Balaz V, Voros J, Civis P, Vojar J, Hettyey A, Sos E, Dankovics R, Jehle R, Christiansen DG, Clare F, Fisher MC, Garner TWJ, Bielby J]

Bales EK, Hyman OJ, Loudon AH, Harris RN, Lipps G, Chapman E, Roblee K, Kleopfer JD, Terrell KA (2015) Pathogenic chytrid fungus *Batrachochytrium dendrobatidis*, but not *B. salamandrivorans*, detected on Eastern Hellbenders. PLoS ONE 10(2): e0116405. doi: 10.1371/journal.pone.0116405

Barber DM (2012) Chytrid fungus, *Batrachochytrium dendrobatidis* (*Bd*), detected at lower elevations in Puerto Rico: Implications for conservation of Puerto Rican Crested Toad (*Peltophryne lemur*). Herpetological Review 43(1): 73–75.

Bardier C, Ghirardi R, Levy M, Maneyro R. (2011) First case of chytridiomycosis in an adult specimen of a native anuran from Uruguay. Herpetological Review 42: 65–66.

Barrasso DA, Cajade R, Nenda SJ, Baloriani G, Herrera R (2009) Introduction of the American bullfrog *Lithobates catesbeianus* (Anura: Ranidae) in natural and modified environments: an increasing conservation problem in Argentina. South American Journal of Herpetology 4: 69–75.

Barrionuevo S, Mangione S (2006) Chytridiomycosis in two species of *Telmatobius* (Anura: Leptodactylidae) from Argentina. Diseases of Aquatic Organisms 73: 171–174.

Barrionuevo JS, Ponssa ML (2008) Decline of three species of the genus *Telmatobius* (Anura: Leptodactylidae) from Tucumán Province, Argentina. Herpetologica 64(1): 47–62.

Barrionuevo JS, Aguayo R, Lavilla EO (2008) First record of chytridiomycosis in Bolivia (*Rhinella quechua*; Anura: Bufonidae). Diseases of Aquatic Organisms 82: 161–163.

Bartkus CJ (2009) The occurrence of *Batrachochytrium dendrobatidis* in salamander populations of West Virginia. MS Thesis. Huntington, WV: Marshall University.

Basanta MD, Calzada-Arciniega RA, Jiménez Velásquez G, Arias-Balderas SF, Ibarra Reyes AA, Medina Rangel G, Suazo-Ortuño I, Ochoa-Ochoa LM, Parra-Olea G (2019) Detection of *Batrachochytrium dendrobatidis* in threatened endemic mole salamanders (*Ambystoma*) in Mexico. Herpetological Review 50(3): 493–495.

Bataille A, Fong JJ, Cha M, Wogan GOU, Baek HJ, Lee H, Min M, Waldman B (2013) Genetic evidence for a high diversity and wide distribution of endemic strains of the pathogenic chytrid fungus *Batrachochytrium dendrobatidis* in wild Asian amphibians. Molecular Ecology 22: 4196–4209. doi: 10.1111/mec.12385

Battaglin WA, Smalling KL, Anderson C, Calhoun D, Chestnut T, Muths E (2016) Potential interactions among disease, pesticides, water quality and adjacent land cover in amphibian habitats in the United States. Science of the Total Environment 566–567: 320–332. http://dx.doi.org/10.1016/j.scitoenv.2016.05.062

Bauer KL, Steeil JC, Walsh TF, Evans MJ, Klocke B, Gratwicke B, Siegal-Willott JL, Neiffer DL (2018) *Batrachochytrium dendrobatidis* in a captive collection of green salamanders (*Aneides aeneus*), long-tailed salamanders (*Eurycea longicauda*), and two-lined salamanders (*Eurycea bislineata*). Journal of Zoo and Wildlife Medicine 49(2): 454–459. https://doi.org/10.1638/2017-0174.1

Beard KH, O’Neill EM (2005) Infection of an invasive frog *Eleutherodactylus coqui* by the chytrid fungus *Batrachochytrium dendrobatidis* in Hawaii. Biological Conservation 126: 591–595.

Becker CG, Rodriguez D, Longo AV, Talaba AL, Zamudio KR (2012) Disease risk in temperate amphibian populations is higher at closed-canopy sites. PLoS ONE 7(10): e48205. doi: 10.1371/journal.pone.0048205

Becker CG, Rodriguez D, Lambertini C, Toledo LF, Haddad CFB (2016) Historical dynamics of *Batrachochytrium dendrobatidis* in Amazonia. Ecography 39(10): 954–960. https://doi.org/10.1111/ecog.02055

Becker CG, Rodriguez D, Lambertini C, Toledo LF, Haddad CFB (2015) Data from: Historical dynamics of *Batrachochytrium dendrobatidis* in Amazonia. https://doi.org/10.5061/dryad.h311

Becker MH, Harris RN (2010) Cutaneous bacteria of the Redback Salamander prevent morbidity associated with lethal disease. PLoS ONE 5(6): e10957. doi:10.1371/journal.pone.0010957

Bell BD, Carver S, Mitchell NJ, Pledger S (2004) The recent decline of a New Zealand endemic: how and why did populations of Archey’s frog *Leiopelma archeyi* crash over 1996–2001? Biological Conservation 120: 189–199.

Bell RC, Gata Garcia AV, Stuart BL, Zamudio KR (2011) High prevalence of the amphibian chytrid pathogen in Gabon. EcoHealth 8: 116–120. doi: 10.1007/s10393-010-0364-4

Berenguel RA, Elias RK, Weaver TJ, Reading RP (2016) Chytrid fungus, *Batrachochytrium dendrobatidis*, in wild populations of the Lake Titicaca Frog, *Telmatobius culeus*, in Peru. Journal of Wildlife Disease 52(4): 973–975. doi: 10.7589/2016-01-007

Berger L, Speare R, Daszak P, Green DE, Cunningham AA, Goggin CL, Slocombe R, Ragan MA, Hyatt AD, McDonald KR, Hines HB, Lips KR, Marantelli G, Parkes H (1998) Chytridiomycosis causes amphibian mortality associated with population declines in the rain forests of Australia and Central America. Proceedings of the National Academy of Sciences USA 95: 9031–9036.

Berger L, Speare R, Hyatt AD (1999) Chytrid fungi and amphibian declines: overview, implications and future directions. In: Campbell A (ed.) Declines and disappearances of Australian frogs*.* Environment Australia, Canberra, pp 23–33.

Bettaso JB, Rachowicz LJ (2006) Overwintering larvae of bullfrogs (*Rana catesbeiana*) as biological reservoirs of the pathogen causing chytridiomycosis (*Batrachochytrium dendrobatidis*) in Trinity County, California. [Abstract]. Northwestern Naturalist 87: 162.

Beyer SE, Phillips CA, Schooley RL (2015) Canopy cover and drought influence the landscape epidemiology of an amphibian chytrid fungus. Ecosphere 6(5): art78.

Bielby J, Bovero S, Sotgiu G, Tessa G, Favelli M, Angelini C, Doglio S, Clare FC, Gazzaniga E, Lapietra F, Garner TWJ (2009) Fatal chytridiomycosis in the Tyrrhenian painted frog. EcoHealth 6: 27–32. doi: 10.1007/s10393-009-0232-2

Bielby J, Bovero S, Angelini C, Favelli M, Gazzianiga E, Perkins M, Sotgiu G, Tessa G, Garner TWJ. (2013) Geographic and taxonomic variation in *Batrachochytrium dendrobatidis* infection and transmission within a highly endemic amphibian community. Diversity and Distributions 19:1153-1163. doi: 10.1111/ddi.12085

Blackburn DC, Evans BJ, Pessier AP, Vredenburg VT (2010) An enigmatic mortality event in the only population of the Critically Endangered Cameroonian frog *Xenopus longipes*. African Journal of Herpetology 59: 111–122. doi: 10.1080/04416651.2010.495674

Blackburn LM (2001) Status of Blanchard’s cricket frogs (*Acris crepitans blanchardi*) along their decline front: population parameters, malformation rates and disease. Muncie, Indiana, USA: Ball State University. Master’s thesis.

Blackburn M, Wayland J, Smith WH, McKenna JH, Harry M, Hamed MK, Gray MJ, Miller DL (2015) First report of ranavirus and *Batrachochytrium dendrobatidis* in Green Salamanders (*Aneides aeneas*) from Virginia, USA. Herpetological Review 46(3): 357–361.

Blackley SK IV (2016) The prevalence of chytridiomycosis in the Southern Appalachians. BS Honors Thesis. Boone, NC: Appalachian State University. https://libres.uncg.edu/ir/asu/listing.aspx?id=21408

Bletz MC, Harris RN (2013) Occurrence of *Batrachochytrium dendrobatidis* in *Notophthalmus viridescens* in northwestern Virginia, USA. Herpetological Review 44(2): 257–259.

Bletz MC, Rosa GM, Andreone F, Courtois EA, Schmeller DS, Rabibisoa NHC, Rabemananjara FCE, Raharivololoniaina L, Vences M, Weldon C, Edmonds D, Raxworthy CJ, Harris RN, Fisher MC, Crottini A (2015) Widespread presence of the pathogenic fungus *Batrachochytrium dendrobatidis* in wild amphibian communities in Madagascar. Scientific Reports 5: 8633 doi: 10.1038/srep08633

Blooi M, Laking AE, Martel A, Haesebrouck F, Jocque M, Brown T, Green S, Vences M, Bletz MC, Pasmans F (2017) Host niche may determine disease-driven extinction risk. PLoS ONE 12(7): e0181051. https://doi.org/10.1371/journal.pone.0181051

Bodinof CM, Briggler JT, Duncan MC, Beringer J, Millspaugh JJ (2011) Historic occurrence of the amphibian chytrid fungus *Batrachochytrium dendrobatidis* in hellbender *Cryptobranchus alleganiensis* populations from Missouri. Diseases of Aquatic Organisms 99: 1–7. doi: 10.3354/dao02380

Boivin E (2012) *Batrachochytrium dendrobatidis* in the Adirondacks, New York, USA. Herpetological Review 43(4): 610.

Böll S, Tobler U, Geiger CC, Hansbauer G, Schmidt BR (2012) The amphibian chytrid fungus in Bavarian populations of *Alytes obstetricans*: past absence, current presence, and metamorph mortality. Amphibia-Reptilia 33(3–4):319–326.

Bonaccorso E, Guyasamin JM, Méndez D, Speare R (2003) Chytridiomycosis as a possible cause of population declines in *Atelopus cruciger* (Anura: Bufonidae). Herpetological Review 34: 331–334.

Borteiro C, Cruz JC, Kolenc F, Aramburu A (2009) Chytridiomycosis in frogs from Uruguay. Diseases of Aquatic Organisms 84: 159–162.

Borteiro C, Cruz JC, Kolenc F, Verdes JM, Moraña A, Debat CM, Kun A, Ubilla M, Okada K (2014) Dermocystid-chytrid coinfection in the Neotropical frog *Hypsiboas pulchellus* (Anura: Hylidae). Journal of Wildlife Diseases 50(1): 150–153. doi: 10.7589/2013-06-151

Borzée A, Kosch TA, Kim M, Jang Y (2017) Introduced bullfrogs are associated with increased *Batrachochytrium dendrobatidis* prevalence and reduced occurrence of Korean treefrogs. PLoS ONE 12(5): e0177860. https://doi.org/10.1371/journal.pone.0177860

Bosch J, Martínez-Solano I (2006) Chytrid fungus infection related to unusual mortalities of *Salamandra salamandra* and *Bufo bufo* in the Peñalara Natural Park, Spain. Oryx 40: 84–89.

Bosch J, Martínez-Solano I, García-París M (2001) Evidence of a chytrid fungus infection involved in the decline of the common Midwife Toad (*Alytes obstricans*) in protected areas of central Spain. Biological Conservation 97: 331–337.

Bosch J, García-Alonso D, Fernández-Beaskoetxea S, Fisher MC, Garner TWJ (2013) Evidence for the introduction of lethal chytridiomycosis affecting wild Betic Midwife Toads (*Alytes dickhilleni*). EcoHealth 10: 82-89. doi: 10.1007/s10393-013-0828-4

Bourke J, Mutschmann F, Ohst T, Ulmer P, Gutsche A, et al. (2010) *Batrachochytrium dendrobatidis* in Darwin’s frog *Rhinoderma* spp. in Chile. Diseases of Aquatic Organisms 92: 217–221. doi: 10.3354/dao02239 (2010).

Bourke J, Ohst T, Gräser Y, Böhme W, Plötner J (2011) New records of *Batrachochytrium dendrobatidis* in Chilean frogs. Diseases of Aquatic Organisms 95: 259–261. doi: 10.3354/dao02369

Bovero S, Sotgiu G, Angelini C, Doglio S, Gazzaniga E, et al. (2008) Detection of chytridiomycosis caused by *Batrachochytrium dendrobatidis* in the endangered Sardinian brook newt *Euproctus platycephalus* in southern Sardinia, Italy. Journal of Wildlife Diseases 44: 712–715.

Bradley GA, Rosen PC, Sredl MJ, Jones TR, Longcore JE (2002) Chytridiomycosis in native Arizona frogs. Journal of Wildlife Diseases 38: 206–212.

Brannelly LA, Chatfield MWH, Richards-Zawacki CL (2012) Field and laboratory studies of the susceptibility of the Green Treefrog (*Hyla cinerea*) to *Batrachochytrium dendrobatidis* infection. PLoS ONE 7(6): e38473.

Brannelly LA, Hunter DA, Lenger D, Scheele BC, Skerratt LF, Berger L (2015) Dynamics of chytridiomycosis during the breeding season in an Australian alpine amphibian. PLoS ONE 10(12): e0143629. doi: 10.1371/journal.pone.0143629

Brannelly LA, Hunter DA, Skerratt LF, Scheele BC, Lenger D, McFadden MS, Harlow PS, Berger L (2016) Chytrid infection and post-release fitness in the reintroduction of an endangered alpine tree frog. Animal Conservation 19: 153–162.

Brannelly LA, Chatfield MWH, Sonn J, Robak M, Richards-Zawacki CL (2018 BMC Ecol) Fungal infection has sublethal effects in a lowland subtropical amphibian population. BMC Ecology 18: 34. https://doi.org/10.1186/s12898-018-0189-5

Brannelly LA, Webb RJ, Hunter DA, Clemann N, Howard K, Skerratt LF, Berger L, Scheele BC (2018 Anim Cons) Non-declining amphibians can be important reservoir hosts for amphibian chytrid fungus. Animal Conservation 21: 91–101. doi: 10.1111/acv.12380

Brem FMR, Lips KR (2008) *Batrachochytrium dendrobatidis* infection patterns among Panamanian amphibian species, habitats and elevations during epizootic and enzootic stages. Diseases of Aquatic Organisms 81: 189–202. doi: 10.3354/dao01960

Bresciano JC, Salvador CA, Paz-y-Miño C, Parody-Merino AM, Bosch J, Woodhams DC (2015) Variation in the presence of anti-*Batrachochytrium dendrobatidis* bacteria of amphibians across life stages and elevations in Ecuador. EcoHealth 12: 310–319. doi: 10.1007/s10393-015-1010-y

Briggler JT, Larson KA, Irwin KJ (2008) Presence of the amphibian chytrid fungus (*Batrachochytrium dendrobatidis*) on hellbenders (*Cryptobranchus alleganiensis*) in the Ozark Highlands. Herpetological Review 39: 443–444.

Briggs C, Burgin S (2004) Congo Red, an effective stain for revealing the chytrid fungus, *Batrachochytrium dendrobatidis*, in epidermal skin scrapings from frogs. Mycologist 18: 98–103.

Briggs CJ, Knapp RA, Vredenburg VT (2010) Enzootic and epizootic dynamics of the chytrid fungal pathogen of amphibians. Proceedings of the National Academy of Sciences USA 107: 9695–9700.

Brocco C (2017) An analysis of prevalence of chytrid fungus in an amphibian assemblage in Middle Tennessee. Honors Thesis, University of Tennessee at Chattanooga. https://scholar.utc.edu/honors-theses/117/

Brodman R, Briggler JT (2008) *Batrachochytrium dendrobatidis* in *Ambystoma jeffersonianum* larvae in southern Indiana. Herpetological Review 39: 320–321.

Brown C, Wilkinson LR, Wilkinson KK, Tunstall T, Foote R, Todd BD, Vredenburg VT (2019) Demography, habitat, and movements of the Sierra Nevada Yellow-legged Frog (*Rana sierrae*) in streams. Copeia 107(4): 661–675.

Brown J, Kerby J (2013) *Batrachochytrium dendrobatidis* in South Dakota, USA amphibians. Herpetological Review 44(3): 457–458.

Burgmeier NG, Unger SD, Meyer JL, Sutton TM, Williams RN (2011) Health and habitat quality assessment for the Eastern Hellbender (*Cryptobranchus alleganiensis alleganiensis*) in Indiana, USA. Journal of Wildlife Diseases 47: 836–845.

Burkart D, Flechas SV, Vredenburg VT, Catenazzi A (2017) Cutaneous bacteria, but not peptides, are associated with chytridiomycosis resistance in Peruvian marsupial frogs. Animal Conservation 20: 483–491. doi: 10.1111/acv.12352

Burrowes PA, De la Riva I (2017) Unraveling the historical prevalence of the invasive chytrid fungus in the Bolivian Andes: implications in recent amphibian declines. Biological Invasions 19: 1791–1794. doi: 10.1007/s10530-017-1390-8

Burrowes PA, Longo AV, Joglar RL, Cunningham AA (2008) Geographic distribution of *Batrachochytrium dendrobatidis* in Puerto Rico. Herpetological Review 39: 321–324.

Burrowes PA, James TY, Jenkinson TS, De la Riva I (2020) Genetic analysis of post-epizootic amphibian chytrid strains in Bolivia: adding a piece to the puzzle. Transboundary and Emerging Diseases [Early View] doi: 10.1111/tbed.13568

Byrne MW, Davie EP, Gibbons JW (2008) *Batrachochytrium dendrobatidis* occurrence in *Eurycea cirrigera*. Southeastern Naturalist 7(3): 551–555. http://www.bioone.org/doi/full/10.1656/1528-7092-7.3.551

Cádiz A, Reytor ML, Díaz LM, Chestnut T, Burns JA, Amato G (2019) The chytrid fungus, *Batrachochytrium dendrobatidis*, is widespread among Cuban amphibians. EcoHealth 16(1): 128–140. https://doi.org/10.1007/s10393-018-1383-9 [Cadiz]

Campbell Grant EH, Bailey LL, Ware JL, Duncan KL (2008) Prevalence of the amphibian pathogen *Batrachochytrium dendrobatidis* in stream and wetland amphibians in Maryland, USA. Applied Herpetology 5: 233–241.

Canessa S, Martel A, Pasmans F (2013) No detection of chytrid in first systematic screening of *Bombina variegata pachypus* (Anura: Bombinatoridae) in Liguria, northern Italy. Acta Herpetologica 8(1): 59–63.

Canestrelli D, Zampiglia M, Nascetti G (2013) Widespread occurrence of *Batrachochytrium dendrobatidis* in contemporary and historical samples of the endangered *Bombina pachypus* along the Italian Peninsula. PLoS ONE 8(5): e63349. doi: 10.1371/journal.pone.0063349

Carey C, Livo LJ (2009) Chytridiomycosis in Woodhouse’s Toad (*Anaxyrus woodhousii*) in Colorado. Herpetological Review 40: 50–52.

Carey C, Cohen N, Rollins-Smith LA (1999) Amphibian declines: An immunological perspective. Developmental and Comparative Immunology 23: 459–472.

Carnaval ACOQ, Toledo LF, Haddad CFB, Britto FB (2005) Chytrid fungus infects high-altitude stream-dwelling *Hylodes magalhaesi* (Leptodactylidae) in the Brazilian Atlantic rainforest. FrogLog 70: 3–4.

Carnaval ACOQ, Puschendorf R, Peixoto OL, Verdade VK, Rodrigues MT (2006) Amphibian chytrid fungus broadly distributed in the Brazilian Atlantic Rain Forest. EcoHealth 3: 41–48. doi: 10.1007/s10393-005-0008-2

Caruso NM, Lips KR (2013) Truly enigmatic declines in terrestrial salamander populations in Great Smoky Mountains National Park. Diversity and Distributions 19: 38–48. doi:10.1111/j.1472-4642.2012.00938.x

Carvalho T, Becker CG, Toledo LF (2017) Historical amphibian declines and extinctions in Brazil linked to chytridiomycosis. Proceedings of the Royal Society B 284(1848): 20162254. https://doi.org/10.1098/rspb.2016.2254

Data in:

Carvalho T, Becker CG, Toledo LF (2017) Data from: Historical amphibian declines and extinctions in Brazil linked to chytridiomycosis. Dryad Digital Repository. https://doi.org/10.5061/dryad.4t53n

Catenazzi A, Vredenburg VT, Lehr E (2010) *Batrachochytrium dendrobatidis* in the live frog trade of *Telmatobius* (Anura: Ceratophryidae) in the tropical Andes. Diseases of Aquatic Organisms 92: 187–191. doi:10.3354/dao02250 (2010a).

Catenazzi A, Lehr E, Rodriguez LO, Vredenburg VT (2011) *Batrachochytrium dendrobatidis* and the collapse of anuran species richness in the upper Manu National Park, southeastern Peru. Conservation Biology 25: 382–391. doi: 10.1111/j.1523-1739.2010.01604.x

Catenazzi A, von May R, Vredenburg VT (2013) High prevalence of infection in tadpoles increases vulnerability to fungal pathogen in high-Andean amphibians. Biological Conservation 159: 413-421. doi: 10.1016/j.biocon.2012.11.023

Catenazzi A, Lehr E, Vredenburg VT (2014) Thermal physiology, disease, and amphibian declines on the eastern slopes of the Andes. Conservation Biology 28(2): 509–517. doi: 10.1111/cobi.12194

Catullo RA, Morgan MJ, Piggott MP, Alford RA (2018) *Batrachochytrium dendrobatidis* surveys from the savannah regions of northern and central Queensland, Australia. Herpetological Review 49(1): 41–44.

Chaber A-L, Combreau O, Perkins M, Saegerman C, Cunningham A (2016) Preliminary survey fail to detect *Batrachochytrium dendrobatidis* infection in the United Arab Emirates and Oman. Herpetological Review 47(3): 403–404.

Chajma P, Vojar J (2016) The effect of size-assortative mating on fertilization success of the Common Toad (*Bufo bufo*). Amphibia-Reptilia 37: 389–395. doi: 10.1163/15685381-00003069

Charbonneau M (2006) Amphibian diseases: Pesticide immunotoxicity and chytridiomycosis in larval *Rana catesbeiana* and ranaviral disease in *Rana sylvatica* tadpoles of central Ontario. MSc thesis, Trent University, Peterborough, Ontario, Canada.

Chatfield MWH, Rothermel BB, Brooks CS, Kay JB (2009) Detection of *Batrachochytrium dendrobatidis* in amphibians from the Great Smoky Mountains of North Carolina and Tennessee, USA. Herpetological Review 40: 176–179.

Chatfield MWH, Moler P, Richards-Zawacki CL (2012) The amphibian chytrid fungus, *Batrachochytrium dendrobatidis*, in fully aquatic salamanders from southeastern North America. PLoS ONE 7(9): e44821. doi: 10.1371/journal.pone.0044821

Cheng TL, Rovito SM, Wake DB, Vredenburg VT (2011) Coincident mass extirpation of neotropical amphibians with the emergence of the infectious fungal pathogen *Batrachochytrium dendrobatidis*. Proceedings of the National Academy of Sciences USA 108(23): 9502–9507. doi: 10.1073/pnas.1105538108

Chestnut T, Johnson JE, Wagner RS (2008) Results of amphibian chytrid (*Batrachochytrium dendrobatidis*) sampling in Denali National Park, Alaska, USA. Herpetological Review 39: 202–204.

Chiari Y, van der Meijden A, Mucedda M, Wagner N, Veith M (2013) No detection of the pathogen *Batrachochytrium dendrobatidis* in Sardinian cave salamanders, genus *Hydromantes*. Amphibia-Reptilia 34: 136–141. doi: 10.1163/15685381-00002876

Chiari Y, Moreno N, Elmore J, Hylton A, Ray A, Burkhardt R, Glaberman S (2017) Widespread occurrence of *Batrachochytrium dendrobatidis* in southern Alabama, USA. Herpetological Review 48(2): 356–359.

Chinnadurai SK, Cooper D, Dombrowski DS, Poore MF, Levy MG (2009) Experimental infection of native North Carolina salamanders with *Batrachochytrium dendrobatidis*. Journal of Wildlife Diseases 45(3): 631–636.

Chong SM, Sng W, Yan BTZ, Wong WK, Siow HJ, Fernandez CJ (2018) Prevalence of chytrid fungus *Batrachochytrium dendrobatidis* in wild amphibians, Singapore. Herpetological Review 49(2): 252–254.

Churgin SM, Raphael BL, Pramuk JB, Trupkiewicz JG, West G (2013) *Batrachochytrium dendrobatidis* in aquatic caecilians (*Typhlonectes natans*): a series of cases from two institutions. Journal of Zoo and Wildlife Medicine 44(4): 1002–1009.

Cisneros-Heredia DF, McDiarmid RW (2007) Revision of the characters of Centrolenidae (Amphibia: Anura: Athesphatanura), with comments on its taxonomy and the description of new taxa of glassfrogs. Zootaxa 1572. [taxonomy reference]

Civiš P, Vojar J, Literák I, Baláž V (2012) Current state of *Bd* occurrence in the Czech Republic. Herpetological Review 43(1): 75–78. [Civis, Literak, Balaz]

Civiš P, Vojar J, Baláž V, Kohutka A, Ulbrichová I, Dvořák V (2013) Sampling for *Batrachochytrium dendrobatidis* in Russia. Herpetological Journal 23: 55–58. [Civis, Balaz, Ulbrichova, Dvorak]

Clemann N, Hunter D, Scroggie M, Pietsch R, Hollis G (2009) Vanishing frogs: prevalence of the amphibian chytrid fungus (*Batrachochytrium dendrobatidis*) in populations of key frog species in the Australian Alps. Arthur Rylah Institute for Environmental Research, Department of Sustainability and Environment, Heidelberg, Victoria, Australia.

Conradie W, Weldon C, Smith KG, du Preez LH (2011) Seasonal patterns of chytridiomycosis in Common River Frog (*Amietia angolensis*) in the South African grassland biome. African Zoology 46(1): 95–102. doi: http://dx.doi.org/10.3377/004.046.0122. URL: http://www.bioone.org/doi/full/10.3377/004.046.0122

Conradie W, Harvey J, Kotzé A, Dalton DL, Cunningham MJ (2011) Confirmed amphibian chytrid in Mount Mulanjie area, Malawi. Herpetological Review 42(3): 369–371.

Conradie W, Bittencourt-Silva GB, Loader SP, Menegon M, Nanvonamuquitxo C, Kotzé A, Dalton DL, Engelbrecht HM, Tolley KA (2016) *Batrachochytrium dendrobatidis* survey of amphibians in the northern Mozambique “Sky Islands” and low-lying areas. Herpetological Review 47(1): 42–46.

Cossel J Jr, Lindquist E, Craig H, Luthman K (2014) Pathogenic fungus *Batrachochytrium dendrobatidis* in marbled water frog *Telmatobius marmoratus*: first record from Lake Titicaca, Bolivia. Diseases of Aquatic Organisms 112: 83–87. doi: 10.3354/dao02778

Courtois EA, Gaucher P, Chave J, Schmeller DS (2015) Widespread occurrence of *Bd* in French Guiana, South America. PLoS ONE 10(4): e0125128. doi: 10.1371/journal.pone.0125128

Coutinho SD, Burke JC, de Paula CD, Rodrigues MT, Catão-Dias JL (2015) The use of singleplex and nested PCR to detect *Batrachochytrium dendrobatidis* in free-living frogs. Brazilian Journal of Microbiology 46(2): 551–555. doi: http://dx.doi.org/10.1590/S1517-838246246220140110

Crother BI (2008) Scientific and standard English names of amphibians and reptiles of North America north of Mexico, with comments regarding confidence in our understanding. Society for the Study of Amphibians and Reptiles Herpetological Circular 37 (2008).

Crottini A, Barbuto M, Casiraghi M, Andreone F (2011) A rapid amphibian survey at Itremo-Ambatofinandrahana, central Madagascar, with confirmed absence of chytrid fungus and recommendations for future monitoring activities. North-western Journal of Zoology 7(2): 346–351.

Crottini A, Bollen A, Weldon C, Dalton DL, Kotzé A, Noël J, Iambana B, Andreone F (2014) Amphibian survey and current absence of *Batrachochytrium dendrobatidis* in Ivoloina Park, Toamasina (eastern Madagascar). African Journal of Herpetology 63(1): 70–78. doi: 10.1080/21564574.2013.833994

Cummer MR, Green DE, O’Neill EM (2005) Aquatic chytrid pathogen detected in a terrestrial Plethodontid salamander. Herpetological Review 36: 248–249.

Cunningham AA, Minting P (2008) National survey of *Batrachochytrium dendrobatidis* infection in UK amphibians, 2008. Final report to Natural England. London: Institute of Zoology, Zoological Society of London. https://www.arguk.org/downloads-in-pages/resources/survey-and-monitoring/27-national-survey-of-batrachochytrium-dendrobatidis-infection-in-uk-amphibians/file

Cunningham AA, Garner TWJ, Aguilar-Sanchez V, Banks B, Foster J, Sainsbury AW, Perkins M, Walker SF, Hyatt A, Fisher M (2005) Emergence of amphibian chytridiomycosis in Britain. Veterinary Record 157: 386–387.

Czeczuga B, Semeniuk A, Czeczuga-Semeniuk E (2011) Chytridiomycosis in three species of *Rana* genus from northeastern Poland. Current Trends in Microbiology 7: 15–20.

Dahanukar N, Krutha K, Paingankar MS, Padhye AD, Modak N, Molur S (2013) Endemic Asian chytrid strain infection in threatened and endemic anurans of the northern Western Ghats, India. PLoS ONE 8(10): e77528. doi: 10.137/journal.pone.0077528

Dahl C, Kiatik I, Baisen I, Bronikowski E, Fleischer RC, Rotzel NC, Lock J, Novotny V, Narayan E, Hero J-M (2012) *Batrachochytrium dendrobatidis* not found in rainforest frogs along an altitudinal gradient of Papua New Guinea. Herpetological Journal 22: 182–186.

D’Aoust-Messier A-M, Echaubard P, Billy V, Lesbarrères D (2015) Amphibian pathogens at northern latitudes: presence of chytrid fungus and ranavirus in northeastern Canada. Diseases of Aquatic Organisms 113: 149–155. doi: 10.3354/dao02837

Daszak P, Strieby A, Cunningham AA, Longcore JE, Brown CC, et al. (2005) Amphibian population declines at Savannah River site are linked to climate, not chytridiomycosis. Ecology 86: 3232–3237.

Daversa D, Bosch J, Jeffery K (2011) First survey of the chytrid fungus, *Batrachochytrium dendrobatidis*, in amphibian populations of Gabon, Africa. Herpetological Review 42: 67–69.

Davidson EW, Parris M, Collins JP, Longcore JE, Pessier AP et al. (2003) Pathogenicity and transmission of chytridiomycosis in Tiger Salamanders (*Ambystoma tigrinum*). Copeia 2003: 601–607.

Davidson SRA, Chambers DL (2011) Occurrence of *Batrachochytrium dendrobatidis* in amphibians of Wise County, VA. Herpetological Review 42(2): 214–215.

Davis JR, Eastlack DT, Kouba AJ, Vance CK (2012) *Batrachochytrium dendrobatidis* detected in Fowler’s Toad (*Anaxyrus fowleri*) populations in Memphis, Tennessee, USA. Herpetological Review 43(1): 81–83.

Deguise I, Richardson JS (2009) Prevalence of the chytrid fungus (*Batrachochytrium dendrobatidis*) in Western Toads in southwestern British Columbia, Canada. Northwestern Naturalist 90: 35–38.

De la Riva I, Burrowes PA (2011) Rapid assessment of the presence of *Batrachochytrium dendrobatidis* in Bolivian Andean frogs. Herpetological Review 42(3): 372–375.

De Léon ME, Vredenburg VT, Piovia-Scott J (2017) Recent emergence of a chytrid fungal pathogen in California Cascades Frogs (*Rana cascadae*). EcoHealth 14: 155–161. doi: 10.1007/s10393-016-1201-1

De Paula CD, Pacífico-Assis EC, Catão-Dias JL (2012) *Batrachochytrium dendrobatidis* in amphibians confiscated from illegal wildlife trade and used in an *ex situ* breeding program in Brazil. Diseases of Aquatic Organisms 98: 171–175. doi: 10.3354/dao02426

Delgado CS, Natale GS, Herrera RA, Barrasso DA (2012) First record of *Batrachochytrium* *dendrobatidis* in *Physalaemus fernandezae* (Anura: Leiuperidae) for Buenos Aires Province, Argentina. Herpetological Review 43(1):84–85.

Díaz L, Cádiz A, Chong A, Silva A (2007) First report of chytridiomycosis in a dying toad (Anura: Bufonidae) from Cuba: A new conservation challenge for the island. EcoHealth 4: 172–175.

Di Leo K (2010) An assessment of the correlation between amphibian populations, chytridiomycete communities, and the ecological integrity of the habitat. Masters thesis, Ecology and Evolution. Rutgers University, Camden, NJ.

Di Rosa I, Simoncelli F, Fagotti A, Pascolini R (2007) The proximate cause of frog declines? Nature 447: E4–E5.

Doherty-Bone TM, Bielby J, Gonwouo NL, LeBreton M, Cunningham AA (2008) In a vulnerable position? Preliminary survey work fails to detect the amphibian chytrid pathogen in the highlands of Cameroon, an amphibian hotspot. Herpetological Journal 18: 115–118.

Doherty-Bone TM, Gonwouo NL, Hirschfeld M, Ohst T, Weldon C, Perkins M, Kouete MT, Browne RK, Loader SP, Gower DJ, Wilkinson MW, Rödel MO, Penner J, Barej MF, Schmitz A, Plötner J, Cunningham AA (2013) *Batrachochytrium dendrobatidis* in amphibians of Cameroon, including first records for caecilians. Diseases of Aquatic Organisms 102: 187–194. doi: 10.3354/dao02557

Drake DL, Altig R, Grace JB, Walls SG (2007) Occurrence of oral deformities in larval anurans. Copeia 2007: 449–458.

Drake MC, Zieger U, Groszkowski A, Gallardo B, Sages P, Reavis R, Faircloth L, Jacobson K, Lonce N, Pinckney R, Cole RC (2014) Survey of helminths, ectoparasites, and chytrid fungus of an introduced population of cane toads, *Rhinella marina* (Anura: Bufonidae), from Grenada, West Indies. Journal of Parasitology 100(5): 608–615. doi: 10.1645/13-470.1

Duncan Pullen K, Best AM, Ware JL (2010) Amphibian pathogen *Batrachochytrium dendrobatidis* prevalence is correlated with season and not urbanization in central Virginia. Diseases of Aquatic Organisms 91: 9–16. doi: 10.3354/dao02249

Ecoclub Amphibian Group, Pope KL, Wengert GM, Foley JE, Ashton DT, Botzler RG (2016) Citizen scientists monitor a deadly fungus threatening amphibian communities in northern coastal California, USA. Journal of Wildlife Diseases 52(3): 516–523. doi: 10.7589/2015-10-280

Edwards L, Alford RA, Pike DA (2019) *Batrachochytrium dendrobatidis* in Southern Ornate Nursery Frogs, *Cophixalus australis* (Microhylidae). Herpetological Review 50(2): 288–289.

El Cadi RA, Laghzaoui E-M, Crottini A, Slimani T, Bosch J, El Mouden EH (2019) Occurrence of *Batrachochytrium dendrobatidis* in the Tensift region, with comments on its spreading in Morocco. Acta Herpetologica 14(2): 109–115. doi: 10.13128/a_h-7748

El Mouden EH, Slimani T, Donaire D, Fernández-Beaskoetxea S, Fisher MC, Bosch J (2011) First record of the chytrid fungus *Batrachochytrium dendrobatidis* in North Africa. Herpetological Review 42: 71–75.

Enciso MA, Villena M, Mendoza AP, Chávez G (2008) Rapid survey of amphibian skin diseases in a mountain forest at the northern Andes of Peru. FrogLog 87: 4–7.

Erismis UC, Konuk M, Yoldas T, Agyar P, Yumuk D, Korcan SE (2014) Survey of Turkey’s endemic amphibians for chytrid fungus *Batrachochytrium dendrobatidis*. Diseases of Aquatic Organisms 111: 153–157. doi: 10.3354/dao02742.

Eskew EA, Todd BD, Hopkins WA (2014) Extremely low prevalence of *Batrachochytrium dendrobatidi*s infection in Eastern Hellbenders (*Cryptobranchus alleganiensis alleganiensis*) in southwest Virginia, USA. Herpetological Review 45(3): 425–427.

Farrer RA, Weinert LA, Bielby J, Garner TWJ, Balloux F, Clare F, Bosch J, Cunningham AA, Weldon C, du Preez LH, Anderson L, Kosakofsky Pond SL, Shahar-Golan R, Henk DA, Fisher MC (2011) Multiple emergences of genetically diverse amphibian-infecting chytrids include a globalized hypervirulent recombinant lineage. Proceedings of the National Academy of Sciences USA 108(46): 18732-18736. www.pnas.org/cgi/doi/10.1073/pnas.1111915108

Federici S, Clemenzi S, Favelli M, Tessa G, Andreone F, Casiraghi M, Crottini A (2008) Identification of the pathogen *Batrachochytrium dendrobatidis* in amphibian populations of a plain area in the Northwest of Italy. Herpetology Notes 1: 33–37.

Felger J, Enssle J, Mendez D, Speare R (2007) Chytridiomycosis in El Salvador. Salamandra 43: 122–127.

Fellers GM, Green DE, Longcore JE (2001) Oral chytridiomycosis in the Mountain Yellow-legged Frog (*Rana muscosa*). Copeia 2001: 945–953.

Fellers GM, Cole RA, Reinitz DM, Kleeman PM (2011) Amphibian chytrid fungus (*Batrachochytrium dendrobatidis*) in coastal and montane California, USA. Herpetological Conservation and Biology 6(3): 383–394.

Fenolio DB, Moreno-Puig V, Levy MG, Núñez JJ, Lamar WW, Fabry MO, Tirado MS, Crump ML, Charrier A (2013 Herp Rev) Status and conservation of a Gondwana legacy: Bullock’s False Toad, *Telmatobufo bullocki* (Amphibia: Anura: Calyptocephalellidae). Herpetological Review 44(4): 583–590.

Fenolio DB, Niemiller ML, Levy MG, Martinez B (2013 R&A) Conservation status of the Georgia Blind Salamander (*Eurycea wallacei*) from the Floridan Aquifer of Florida and Georgia. Reptiles and Amphibians 20(3): 97–111.

Fernández-Beaskoetxea S, Carrascal LM, Fernández-Loras A, Fisher MC, Bosch JC (2015) Short term minimum water temperatures determine levels of infection by the amphibian chytrid fungus in *Alytes obstetricans* tadpoles. PLoS ONE 10(3): e0120237. doi: 10.1371/journal.pone.0120237 [Fernandez-Beaskoetxea]

Fernández-Loras A, Boyero L, Correa-Araneda F, Tejedo M, Hettyey A, Bosch J (2019) Infection with *Batrachochytrium dendrobatidis* lowers heat tolerance of tadpole hosts and cannot be cleared by brief exposure to CTmax. PLoS ONE 14(4): e0216090. https://doi.org/10.1371/journal.pone.0216090 [Fernandez-Loras]

Ferreira TK, Lamarão FRM, Moraes MO, Van Sluys M (2008) Amphibian chytrid infection in *Melanophryniscus moreirae* (Bufonidae) in the Brazilian Atlantic Rainforest. Herpetological Review 39: 445–446.

Ficetola GF, Valentini A, Miaud C, Noferini A, Mazzotti S, Dejean T (2011) *Batrachochtyrium dendrobatidis* in amphibians from the Po River Delta, northern Italy. Acta Herpetologica 6(2):297–302.

Firkins MP (2015) Prevalence and distribution of ranavirus, chytrid fungus, and helminths in North Dakota amphibians. MS thesis. Grand Forks, ND: University of North Dakota. Theses and Dissertations 1895. https://commons.und.edu/theses/1895

Flechas SV, Sarmiento C, Amézquita A (2012) *Bd* on the beach: high prevalence of *Batrachochytrium dendrobatidis* in the lowland forests of Gorgona Island (Colombia, South America). EcoHealth 9: 298–302. doi: 10.1007/s10393-012-0771-9

Flechas SV, Medina EM, Crawford AJ, Sarmiento C, Cárdenas ME, Amézquita A, Restrepo S (2013) Characterization of the first *Batrachochytrium dendrobatidis* isolate from the Colombian Andes, an amphibian biodiversity hotspot. EcoHealth 10: 72–76. doi: 10.1007/s10393-013-0823-9

Flechas SV, Vredenburg VT, Amézquita A (2015) Infection prevalence in three lowland species of Harlequin Toads from the threatened genus *Atelopus*. Herpetological Review 46(4): 528–532.

Flechas SV, Paz A, Crawford AJ, Sarmiento C, Acevedo AA, Arboleda A, Bolívar-García W, Echeverry-Sandoval CL, Franco R, Mojica C, Muñoz A, Palacios-Rodríguez P, Posso-Terranova AM, Quintero-Marín P, Rueda-Solano LA, Castro-Herrera F, Amézquita A (2017) Current and predicted distribution of the pathogenic fungus *Batrachochytrium dendrobatidis* in Colombia, a hotspot of amphibian biodiversity. Biotropica 49(5): 685–694. doi: 10.1111/btp.12457

Fong JJ, Cheng TJ, Battaile A, Pessier AP, Waldman B, Vredenburg VT (2015) Early 1900s detection of *Batrachochytrium dendrobatidis* in Korean amphibians. PLoS ONE 10(3): e0115656. doi: 10.1371/journal.pone.0115656

Forrest MJ, Schlaepfer MA (2011) Nothing a hot bath won’t cure: infection rates of amphibian chytrid fungus correlate negatively with water temperature under natural field settings. PLoS ONE 6(12): e28444. DOI: 10.1371/journal.pone.0028444

Forrest MJ, Edwards MS, Rivera R, Sjöberg JC, Jaeger JR (2015) High prevalence and seasonal persistence of amphibian chytrid fungus in the desert-dwelling Amargosa Toad, *Anaxyrus nelsoni*. Herpetological Conservation and Biology 10(3): 917–925.

Forzán MJ, Vanderstichel R, Hogan NS, Teather K, Wood J (2010) Prevalence of *Batrachochytrium dendrobatidis* in three species of wild frogs on Prince Edward Island, Canada. Diseases of Aquatic Organisms 91: 91–96. doi: 10.3354/dao02244

Fox SF, Greer AL, Torres-Cervantes R, Collins JP (2006) First case of ranavirus-associated morbidity and mortality in natural populations of a South American frog, *Atelognathus patagonicus*. Diseases of Aquatic Organisms 72: 87–92.

Frías-Alvarez P, Vredenburg VT, Familiar-López M, Longcore JE, González-Bernal E et al. (2008) Chytridiomycosis survey in wild and captive Mexican amphibians. EcoHealth 5: 18–26.

Freitas ES, Rujirawan A, Ampai N, Puanprapai P, Yodthong S, Termprayoon K, Siler CD, Aowphol A (2019) Amphibian surveys reveal no instances of *Batrachochytrium dendrobatidis* and suggest low prevalence of chytrid fungus in Thailand. Herpetological Review 50(2): 290–298.

Fungus threatens to wipe out Philippine frogs (AFP–Agence France Press) – newspaper article citing Arvin Diesmos and Rafe Brown. Available at: https://tech.hindustantimes.com/tech/news/fungus-threatens-to-wipe-out-philippine-frogs-experts-story-qEB8azm8mlrzfRfV97BWTK.html, accessed 25 February 2021.

Gabor C, Fisher MC, Bosch J (2013) A non-invasive stress assay shows that tadpole populations infected with *Batrachochytrium dendrobatidis* have elevated corticosterone levels. PLoS ONE 8(2): e56054. doi: 10.1371/journal.pone.0056054

Gabor C, Forsburg Z, Vörös J, Serrano-Laguna C, Bosch J (2017) Differences in chytridiomycosis infection costs between two amphibian species from Central Europe. Amphibia-Reptilia 38: 250–256. doi: 10.1163/15685381-00003099

Gaertner JP, Hahn D, Forstner MRJ (2007) Detection of *Batrachochytrium dendrobatidis* in four endemic Central Texas amphibians. Abstracts of Nov. 2007 *Bd* Conference, Available at: http://www.parcplace.org/documents/Bd_Program_post-FINAL.pdf.

Gaertner JP, Gaston MA, Spontak D, Forstner MRJ, Hahn D (2009a) Seasonal variation in the detection of *Batrachochytrium dendrobatidis* in a Texas population of Blanchard’s Cricket Frog (*Acris crepitans blanchardi*). Herpetological Review 40: 184–187.

Gaertner JP, Forstner MRJ, O’Donnell L, Hahn D (2009b) Detection of *Batrachochytrium dendrobatidis* in endemic salamander species from Central Texas. EcoHealth 6: 20–26. doi: 10.1007/s10393-009-0229-x

Gaertner JP, McHenry D, Forstner MRJ, Hahn D (2010) Annual variation of *Batrachochytrium dendrobatidis* in the Houston Toad (*Bufo houstonensis*) and a sympatric congener (*Bufo nebulifer*). Herpetological Review 41: 456–459.

Gaertner JP, Mendoza JA II, Neang T, Forstner MR, Hahn D (2011) Detection of *Batrachochytrium dendrobatidis* in frogs from different locations in Cambodia. Herpetological Review 42(4): 546–549.

Gaertner JP, Brown DJ, Mendoza JA, Forstner MRJ, Bonner T, Hahn D (2012) Geographic variation in *Batrachochytrium dendrobatidis* occurrence among populations of *Acris crepitans blanchardi* in Texas, USA. Herpetological Review 43(2): 274–278.

Gál JT, Szabó K, Vörös J (2012) Kitridiomikózis vizsgálata egy magas-bakonyi vizes élőhely kétéltűközösségén. [Investigation of chytridiomycosis in an amphibian community in a high-Bakony wetland.] Állattani Közlemények (Journal of the Zoology Department of the Hungarian Biological Society) 97(1): 47–59. [Gal, Szabo, Voros] [in Hungarian]

Galindo-Bustos MA, Hernandez-Jauregui DMB, Cheng T, Vredenburg V, Parra-Olea G (2014) Presence and prevalence of *Batrachochytrium dendrobatidis* in commercial amphibians in Mexico City. Journal of Zoo and Wildlife Medicine 45(4): 830–835.

Gandola R, Hendry CR (2013) No detection of the chytrid fungus (*Batrachochytrium dendrobatidis*) in a multi-species survey of Ireland’s native amphibians. Herpetological Journal 23: 233–236.

Garcia G, Cunningham AA, Horton DL, Garner TWJ, Hyatt A et al. (2007) Mountain chickens *Leptodactylus fallax* and sympatric amphibians appear to be disease free on Montserrat. Oryx 41: 398–401.

Garcia G, Lopez J, Fa JE, Gray GAL (2009) Chytrid fungus strikes mountain chickens in Montserrat. (subarticle in Conservation news). Oryx 43: 323–324.

García-Roa R, Sunyer J, Fernández-Loras A, Bosch J (2014) First record of *Batrachochytrium dendrobatidis* in Nicaragua. Herpetological Journal 24: 65–68.

Garner TWJ, Walker S, Bosch J, Hyatt AD, Cunningham AA et al. (2005) Chytrid fungus in Europe. Emerging Infectious Diseases 11: 1639–1640.

Garner TWJ, Perkins MW, Govindarajulu P, Seglie D, Walker S et al. (2006) The emerging amphibian pathogen *Batrachochytrium dendrobatidis* globally infects introduced populations of the North American bullfrog, *Rana catesbeiana*. Biology Letters 2: 455–459.

Garner TWJ, Walker S, Bosch J, Leech S, Rowcliffe JM, Cunningham AA, Fisher MC (2009) Life history tradeoffs influence mortality associated with the amphibian pathogen *Batrachochytrium dendrobatidis*. Oikos 118: 783–791. doi: 10.1111/j.1600-0706.2008.17202.x

Gaudreau MM, Thiet RK, Perrotti L (2010) Incidence of *Batrachochtyrium dendrobatidis* (*Bd*) in Rhode Island anuran populations. Archived PowerPoint presentation. Boston, MA: U.S. Environmental Protection Agency, Region 1. https://archive.epa.gov/region1/neaeb2010/web/pdf/1b-incidencebatrachochytriumdendrobatidis.pdf

Gaulke CA, Irwin JT, Wagner RS (2011) Prevalence and distribution of *Batrachochytrium dendrobatidis* at montane sites in central Washington state, USA. Herpetological Review 42(2): 209–211.

Ghirardi R (2011) Estudio de quitridiomicosis por *Batrachochytrium dendrobatidis* en anfibios anuros del Litoral, Cuyo y Patagonia Argentina. Ph.D. thesis. La Plata, Buenos Aires, Argentina: Universidad Nacional de La Plata.

Ghirardi R, Lescano JN, Longo MS, Robledo G, Steciow MM et al. (2009) *Batrachochytrium dendrobatidis* in Argentina: First record in *Leptodactylus gracilis* and another record in *Leptodactylus ocellatus*. Herpetological Review 40: 175–176.

Ghirardi R, López JA, Scarabotti PA, Steciow MM, Perotti MG (2011) First record of the chytrid fungus in *Lithobates catesbeianus* from Argentina: exotic species and conservation. Revista Mexicana de Biodiversidad 82: 1337–1339.

Ghirardi R, Levy MG, López JA, Corbalán V, Steciow MM, Perotti MG (2014) Endangered amphibians infected with the chytrid fungus *Batrachochytrium dendrobatidis* in Austral temperate wetlands from Argentina. Herpetological Journal 24: 129–133.

Ghirardi R, López JA, Sanabria EA, Quiroga LB, Levy MG (2017a) First report of *Batrachochytrium dendrobatidis* in the Warty Toad, *Rhinella spinulosa* (Wiegmann, 1834), from the Argentinean Andean foothills. Herpetological Review 48(1): 64–66.

Ghirardi R, López JA, Sanabria EA, Quiroga LB, Levy MG (2017b) Pathogenic fungus in feral populations of the invasive North American bullfrog in Argentina. Belgian Journal of Zoology 147(2): 81–86. https://doi.org/10.26496/bjz.2017.7

Ghirardi R, López JA, Antoniazzi CE (2018) The chytrid fungus *Batrachochytrium dendrobatidis* infecting the Creole Frog, *Leptodactylus latrans*, in a new region of Argentina. Herpetological Review 49(2): 255–257.

Gibbons et al. (in review, Southeastern Naturalist); data from Mike Byrne, NPS, SECN, Michael_W_Byrne@nps.gov, 11/07/2007

Gilbert M, Bickford D, Clark L, Johnson A, Joyner PH, Ogg Keats L, Khammavong K, Nguyễn Vặn L, Newton A, Seow TPW, Roberton S, Silithammavong S, Singhalath S, Yang A, Seimon TA (2012) Amphibian pathogens in southeast Asian frog trade. EcoHealth 9: 386–398. DOI: 10.1007/s10393-019-0817-7

Gillespie GR, Hunter D, Berger L, Marantelli G (2015) Rapid decline and extinction of a montane frog population in southern Australia follows detection of the amphibian pathogen *Batrachochytrium dendrobatidis*. Animal Conservation 18: 295–302. doi: 10.1111/acv12174

Glenney GW, Julian JT, Quartz WM (2010) Preliminary amphibian health survey in the Delaware Water Gap National Recreation Area. J Aquat Anim Health 22: 102–114. doi: 10.1577/H09-037.1

Glorioso BM, Waddle JH, Richards-Zawacki CL (2017) Prevalence of *Batrachochytrium dendrobatidis* and *B. salamandrivorans* in the Gulf Coast Waterdog, *Necturus beyeri*, from southeast Louisiana, USA. Herpetological Review 48(2): 360–353.

Gluesencamp AG, Muscher-Hodges BJ, Lee MM, Sandoval NM, Fenolio DB (2018) Sampling for *Batrachochytrium dendrobatidis* and *B. salamandrivorans* in the Texas Blind Salamander (*Eurycea rathbuni*). Herpetological Review 49(1): 44–46.

Göçmen B, Veith M, Iğci N, Akman B, Godmann O, Wagner N (2013) No detection of the amphibian pathogen *Batrachochytrium dendrobatidis* in terrestrial Turkish salamanders (*Lyciasalamandra*) despite its occurrence in syntopic frogs (*Pelophylax bedriagae*). Salamandra 49(1): 55–55. [Gocmen, Igci]

Goka K, Yokoyama J, Une Y, Kuroki T, Suzuki K et al. (2009) Amphibian chytridiomycosis in Japan: distribution, haplotypes and possible route of entry into Japan. Molecular Ecology 18: 4757–4774.

Goldberg CS, Hawley TJ, Waits LP (2009) Local and regional patterns of amphibian chytrid prevalence on the Osa Peninsula, Costa Rica. Herpetological Review 40: 309–311.

Goldberg TL, Readel AM, Lee MH (2007) Chytrid fungus in frogs from an equatorial African montane forest in western Uganda. Journal of Wildlife Diseases 43: 521–524.

Gonyor JL, Yabsley MJ, Jensen JB (2011) A preliminary survey of *Batrachochytrium dendrobatidis* exposure in Hellbenders from a stream in Georgia, USA. Herpetological Review 42(1): 58–59.

Goodman RM, Ararso YT (2012) Survey of ranavirus and the fungus *Batrachochytrium dendrobatidis* in frogs of central Virginia, USA. Herpetological Review 43(1): 78–80.

Goodman RM, Tyler JA, Reinartz DM, Wright AN (2019) Survey of ranavirus and *Batrachochytrium dendrobatidis* in introduced frogs in Hawaii, USA. Journal of Wildlife Diseases 55(3): 668–672. doi: 10.7589/2018-05-137

Govindarajulu PP, Garner TWJ, Anholt BR (2006) Assessing prevalence of chytrid fungus (*Batrachochytrium dendrobatidis*) in native amphibians and bullfrogs (*Rana catesbeiana*) on Vancouver Island, British Columbia. Northwestern Naturalist 87: 170 [Abstract].

Gower DJ, Doherty-Bone TM, Aberra RK, Mengistu A, Schwaller S, Menegon M, de Sá R, Saber SA, Cunningham AA, Loader SP (2012) High prevalence of the amphibian chytrid fungus (*Batrachochytrium dendrobatidis*) across multiple taxa and localities in the highlands of Ethiopia. Herpetological Journal 22: 225–233.

Gower DJ, Doherty-Bone T, Loader SP, Wilkinson M, Kouete MT, Tapley B, Orton F, Daniel OZ, Wynne F, Flach E, Müller H, Menegon M, Stephen I, Browne RK, Fisher MC, Cunningham AA, Garner TWJ (2013) *Batrachochytrium dendrobatidis* infection and lethal chytridiomycosis in caecilian amphibians (Gymnophiona). EcoHealth 10: 173–183. doi: 10.1007/s10393-013-0831-9

Grano M, Cattaneo C (2015) A survey on the prevalence of the invasive alien American bullfrog, *Lithobates catesbeianus* (Shaw, 1802) (Amphibia Anura Ranidae) in Latium (Central Italy) with reference to a possible infection of *Batrachochytrium dendrobatidis* on *Bufo bufo*. Hyla 2015 (1): 70–75.

Grasselli E, Bianchi G, Dondero L, Marchiano V, Carafa M, Perrone M, Salvidio S (2019) First screening for *Batrachochytrium salamandrivorans* (Bsal) in wild and captive salamanders from Italy. Salamandra 55(2): 124–126.

Gratwicke B, Alonso A, Elie T, Kolowski J, Lock J, Rotzel N, Sevin J, Fleischer RC (2011) *Batrachochytrium dendrobatidis* not detected in amphibians from two lowland sites in Gabon, Africa. Herpetological Review 42(1): 69–71.

Gratwicke B, Evans M, Campbell Grant EH, Greathouse J, McShea WJ, Rotzel N, Flescher RC. (2011) Low prevalence of *Batrachochytrium dendrobatidis* detected in salamanders from Warren County, Virginia, USA. Herpetological Review 42(2): 217–219.

Green DE, Dodd CK (2007) Presence of amphibian chytrid fungus *Batrachochytrium dendrobatidis* and other amphibian pathogens at warm-water fish hatcheries in southeastern North America. Herpetological Conservation and Biology 2: 43–47.

Green DE, Kagarise Sherman C (2001) Diagnostic histological findings in Yosemite toads (*Bufo canorus*) from a die-off in the 1970’s. Journal of Herpetology 35: 92–103.

Green DE, Muths E (2005) Health evaluation of amphibians in and near Rocky Mountain National Park (Colorado, USA). Alytes 22: 109–129.

Green DE, Converse KA, Schrader AK (2002) Epizootiology of sixty-four amphibian morbidity and mortality events in the USA, 1996–2001. Annals of the New York Academy of Sciences 969: 323–339.

Greenbaum E, Kusamba C, Aristote MM, Reed K (2008) Amphibian chytrid fungus infections in *Hyperolius* (Anura: Hyperoliidae) from eastern Democratic Republic of Congo. Herpetological Review 39: 70–73.

Greenbaum E, Meece J, Reed KD, Kusamba C (2014) Amphibian chytrid infections in non-forested habitats of Katanga, Democratic Republic of Congo. Herpetological Review 45(4): 610–614.

Greenbaum E, Meece J, Reed KD, Kusamba C (2015) Extensive occurrence of the amphibian chytrid fungus in the Albertine Rift, a Central African amphibian hotspot. Herpetological Journal 25: 91–100.

Greener MS, Shepherd R, Hoskisson PA, Asmath H, Downie JR (2017) How many Trinidad stream frogs (*Mannophryne trinitatis*) are there, and should they be regarded as vulnerable to extinction? Herpetological Journal 27(1): 5–11.

Grogan LF, Phillott AD, Scheele BC, Berger L, Cashins SD, Bell SC, Puschendorf R, Skerratt LF (2016) Endemicity of chytridiomycosis features pathogen overdispersion. Journal of Animal Ecology 85: 806–816. doi: 10.1111/1365-2656.12500

Groner ML, Relyea RA (2010) *Batrachochytrium dendrobatidis* is present in northwest Pennsylvania, USA, with high prevalence in *Notophthalmus viridescens*. Herpetological Review 41: 462–465.

Grummer JA, Leaché AD (2016) Survey for *Batrachochytrium dendrobatidis* in the North Cascades National Park Service complex, Washington, USA. Herpetological Review 47(3): 392–394.

Gründler MC, Toledo LF, Parra-Olea G, Haddad CFB, Giasson LOM, Sawaya RJ, Prado CPA, Araujo OGS, Zara FJ, Centeno FC, Zamudio KR (2012) Interaction between breeding habitat and elevation affects prevalence but not infection intensity of *Batrachochytrium dendrobatidis* in Brazilian anuran assemblages. Diseases of Aquatic Organisms 97: 173–187. doi: 10.3354/dao02413

Guthrie A, Sweeney R, Steele K (2017) *Batrachochytrium dendrobatidis* and *Batrachochytrium salamandrivorans* surveillance in salamanders of southeastern Virginia, USA. Herpetological Review 48(2): 363–365.

Gutierrez FR, Arellano ML, Moreno LE, Natale GS (2010) *Batrachochytrium dendrobatidis* in Argentina: First record of infection in *Hypsiboas cordobae* and *Odontophrynus occidentalis* tadpoles, in San Luis Province. Herpetological Review 41: 323–325.

Gutsche A, McCranie JR, Ohst T, Valdés Orellana L (2015) New records of the chytrid fungus *Batrachochytrium dendrobatidis* in Honduran frogs. Herpetological Review 46(2): 202–205.

Hagman M, Alford RA (2015) Patterns of *Batrachochytrium dendrobatidis* transmission between tadpoles in a high-elevation rainforest stream in tropical Australia. Diseases of Aquatic Organisms 115:213–221. doi: 10.3354/dao02898

Hale SF, Rosen PC, Jarchow JL, Bradley GA (2005) Effects of chytrid fungus on the Tarahumara Frog (*Rana tarahumarae*) in Arizona and Sonora, Mexico. In: Gottfried GJ, Gebow BS, Eskew LG, Edminster CB (compilers) Connecting mountain islands and desert seas: biodiversity and management of the Madrean Archipelago II. 2004 May 11–15; Tucson, AZ. Proceedings RMRS-P-36. Fort Collins, CO: U.S. Department of Agriculture, Forest Service, Rocky Mountain Research Station. pp. 407–411.

Hanlon SM, Smith D, Kerby JL, Berg E, Peterson W, Parris MJ, Moore JE (2014 Arkansas) Occurrence of *Batrachochytrium dendrobatidis* in Wapanocca National Wildlife Refuge, Arkansas, USA. Herpetological Review 45(1): 31–32.

Hanlon SM, Smith D, Kerby JL, Parris MJ, Moore JE (2014 Tennessee) Detection of *Batrachochytrium dendrobatidis* infections in amphibian populations at Edward J. Meeman Biological Field Station, Tennessee, USA. Herpetological Review 45(1): 32–35.

Hanselmann R, Rodríguez A, Lampo M, Fajardo-Ramos L, Aguirre AA, et al. (2004) Presence of an emerging pathogen of amphibians in introduced bullfrogs *Rana catesbeiana* in Venezuela. Biological Conservation 120: 115–119.

Harner MJ, Nelson AJ, Geluso K, Simon DM (2011) Chytrid fungus in American Bullfrogs (*Lithobates catesbeianus*) along the Platte River, Nebraska, USA. Herpetological Review 42(4): 549–511.

Harner MJ, Merlino JN, Wright GD (2013) Amphibian chytrid fungus in Woodhouse’s Toads, Plains Leopard Frogs, and American Bullfrogs along the Platte River, Nebraska, USA. Herpetological Review 44(3): 459–461.

Hasken J, Newby JL, Grelle AM, Boling J, Estes J et al. (2009) Evaluation of chytrid infection level in a newly discovered population of *Anaxyrus boreas* in the Rio Grande National Forest, Colorado, USA. Herpetological Review 40: 426–428.

Hauselberger KF, Alford RA (2012) Prevalence of *Batrachochytrium dendrobatidis* infection is extremely low in direct-developing Australian microhylids. Diseases of Aquatic Organisms 100: 191–200. doi: 10.3354/dao02494

Havlíková B, Baláz V, Vojar J (2015) First systematic monitoring of *Batrachochytrium dendrobatidis* in collections of captive amphibians in the Czech Republic. Amphibia-Reptilia 36: 27–35. doi: 10.1163/15685381-00002972

Hayes MP, Rombough CJ, Padgett-Flohr GE, Hallock LA, Johnson JE et al. (2009) Amphibian chytridiomycosis in the Oregon Spotted Frog (*Rana pretiosa*) in Washington state, USA. Northwestern Naturalist 90: 148–151

Heard GW, Scroggie MP, Clemann N, Ramsey DSL (2014) Wetland characteristics influence disease risk for a threatened amphibian. Ecological Applications 24(4): 650–652.

Hedges SB, Duellman WE, Heinicke MP (2008) New World direct-developing frogs (Anura: Terrarana): Molecular phylogeny, classification, biogeography, and conservation. Zootaxa 1737: 1–182. [taxonomy reference]

Heinicke MP, Duellman WE, Hedges SB (2007) Major Caribbean and Central American frog faunas originated by ancient oceanic dispersal. Proceedings of the National Academy of Sciences USA 104: 10092–10097.

Hernández-Martínez LA, Romero-Méndez U, González-Barrios JL, García-De la Peña MC, Amézquita-Torres A (2019) Nuevos registros y prevalencia de *Batrachochytrium dendrobatidis* en anuros de la Cuenca Nazas-Aguanaval en la region norte-centro de México [New records and prevalence of *Batrachochytrium dendrobatidis* in anurans from the Nazas-Aguanaval basin in the north-central region of Mexico]. Revista Mexicana de Biodiversidad 90: e902934. https://doi.org/10.22201/ib.20078706e.2019.90.2934 [in Spanish] [Hernandez-Martinez, Romero-Mendez, Gonzalez-Barrios, Garcia-De la Pena, Amezquita-Torres] [taxonomy reference]

Herrera RA, Seciow MM, Natale GS (2005) Chytrid fungus parasitizing the wild amphibian *Leptodactylus ocellatus* (Anura: Leptodactylidae) in Argentina. Diseases of Aquatic Organisms 64: 247–252.

Hertz A, Ponce M, Madani G, Bland A, Petchey A, Andrén C, Eisenberg T (2018) Low *Batrachochytrium dendrobatidis* prevalence in two persisting post-decline populations of endangered hylid frogs in western Panama. Salamandra 54(1): 83–86.

Hidalgo-Vila J, Díaz-Paniagua C, Marchand MA, Cunningham AA (2012) *Batrachochytrium dendrobatidis* infection of amphibians in the Doñana National Park, Spain. Diseases of Aquatic Organisms 98: 113–119. doi: 10.3354/dao02419

Hill RL, Levy MG (2014) Prevalence of *Batrachochytrium dendrobatidis* in pond-breeding amphibians of the Fall Line Sandhills region of Georgia, USA. Herpetological Review 45(2): 236–238.

Hill RL, Levy MG, Timpe EK, Kaylock JB (2011) Additional reports of the amphibian chytrid fungus *Batrachochytrium dendrobatidis* from Georgia, USA. Herpetological Review 42(3): 376–378.

Hirschfeld M, Blackburn DC, Doherty-Bone TM, Gonwouo LN, Ghose S, Rödel M-O (2016) Dramatic declines of montane frogs in a Central African biodiversity hotspot. PLoS ONE 11(5): e0155129. doi: 10.1371/journal.pone.0155129

Holmes I, McLaren K, Wilson B (2012) Surveys for frog diversity and *Batrachochytrium dendrobatidis* in Jamaica. Herpetological Review 43(2): 278–282.

Holmes I, McLaren, Wilson B (2014) Precipitation constrains amphibian chytrid fungus infection rates in a terrestrial frog assemblage in Jamaica, West Indies. Biotropica 46(2): 219–228. doi: 10.111/btp.12093

Hopkins S, Channing A (2003) Chytrid fungus in Northern and Western Cape frog populations, South Africa. Herpetological Review 34: 334–336.

Horner AA, Hoffman EA, Tye MR, Hether TD, Savage AE (2017) Cryptic chytridiomycosis linked to climate and genetic variation in amphibian populations in the southeastern United States. PLoS ONE 12(4): e0175843. https://doi.org/10.1371/journal.pone.0175843

Hoskin CJ, Hines HB, Webb RJ, Skerratt LF, Berger L (2018) Naïve rainforest frogs on Cape York, Australia, are at risk of the introduction of the amphibian chytridiomycosis disease. Australian Journal of Zoology 66: 174–178. https://doi.org/10.1071/ZO18041

Hossack BR, Adams MJ, Campbell Grant EH, Pearl CA, Bettaso JB, Barichivich WJ, Lowe WH, True K, Ware JL, Corn PS (2010) Low prevalence of chytrid fungus (*Batrachochytrium dendrobatidis*) in amphibians in U.S. headwater streams. Journal of Herpetology 44: 253–260.

Hoverman JT, Mihaljevic JR, Richgels KLD, Kerby JL, Johnson PTJ (2012) Widespread co-occurrence of virulent pathogens within California amphibian communities. EcoHealth 9: 288–292. doi: 10.1007/s10393-012-0778-2

Howard K, Cleeland C, Clemann N (2010) Assessment of the status of the threatened Bibron’s Toadlet and Southern Toadlet in areas affected by the Kilmore East-Murrindindi fires. Black Saturday, Victoria 2009 – Natural values fire recovery program. Heidelberg, Victoria, Australia: Department of Sustainability and Environment.

Howard K, Clemann N, Antrobus J (2012) Assessment of the status of threatened herpetofauna following fire in sub alpine habitat at Lake Mountain and Mount Bullfight, near Marysville, north-east Victoria. Black Saturday Victoria 2009—Natural values fire recovery program. Heidelberg, Victoria, Australia: Department of Sustainability and Environment. https://www.ari.vic.gov.au/__data/assets/pdf_file/0023/34961/VBRRA-P8-web-rev.pdf

http://www.jcu.edu.au/school/phtm/PHTM/frogs/chyglob.htm—see Speare and Berger 2005

Huang R, Wilson LA (2013) *Batrachochytrium dendrobatidis* in amphibians of the Piedmont and Blue Ridge provinces in northern Georgia, USA. Herpetological Review 44(1): 95–98.

Hudson MA, Young RP, Lopez J, Martin L, Fenton C, McCrea R, Griffiths RA, Adams S-L, Gray G, Garcia G, Cunningham AA (2016) In-situ itraconazole treatment improves survival rate during an amphibian chytridiomycosis epidemic. Biological Conservation 195: 37–45. http://dx.doi.org/10.1016/j.biocon.2015.12.041

Hudson MA, Griffiths RA, Martin L, Fenton C, Adams S-L, Blackman A, Sulton M, Perkins MW, Lopez J, Garcia G, Tapley B, Young RP, Cunningham AA (2019) Reservoir frogs: seasonality of *Batrachochytrium dendrobatidis* infection in robber frogs in Dominica and Montserrat. PeerJ 7:e7021. doi: 10.7717/peerj.7021

Hughey MC, Becker MH, Walke JB, Swartwout MC, Belden LK (2014) *Batrachochytrium dendrobatidis* in Virginia

amphibians: within and among site variation in infection. Herpetological Review 45(3): 428–438.

Hunter D, Pietsch R, Clemann N, Scroggie M, Hollis G, Marantelli G (2009) Prevalence of the amphibian chytrid fungus (*Batrachochytrium dendrobatidis*) in the Australian Alps. Report to the Australian Alps Liaison Committee, January 2009. Department of Environment & Climate Change, New South Wales and Department of Sustainability and Environment, Victoria. https://theaustralianalps.files.wordpress.com/2013/12/chytrid-report.pdf

Hunter DA, Speare R, Marantelli G, Mendez D, Pietsch R, Osborne W (2010) Presence of the amphibian chytrid fungus *Batrachochytrium dendrobatidis* in threatened corroboree frog populations in the Australian Alps. Diseases of Aquatic Organisms 92: 209–216. doi: 10.3354/dao02118

Huss M, Huntley L, Vredenburg V, Johns J, Green S (2013) Presence of *Batrachochytrium dendrobatidis* in 120 archived specimens of *Lithobates catesbeianus* (American Bullfrog) collected in California, 1924–2007. EcoHealth 10: 339–343. doi: 10.1007/s10393-013-0895-6

Hydeman ME, Bell RC, Drewes RC, Zamudio KR (2013) Amphibian chytrid fungus confirmed in endemic frogs and caecilians on the island of São Tomé, Africa. Herpetological Review 44(2): 254–257.

Hyman OJ, Collins JP (2012) Evaluation of a filtration-based method for detecting *Batrachochytrium dendrobatidis* in natural bodies of water. Diseases of Aquatic Organisms 97: 185–195. doi: 10.3354/dao02423

Hyman OJ, Collins JP (2015) *Batrachochytrium dendrobatidis* dynamics in an isolated Northern Leopard Frog (*Lithobates pipiens*) population in Arizona. Herpetological Review 46(4): 535–537.

Igleski MJ, Nicholson KE (2014) Spatial pattern of *Batrachochytrium dendrobatidis* infection in Green Frogs (*Lithobates clamitans*) in Michigan, USA. Herpetological Review 45(1): 34–40.

Imasuen AA, Aisien MSO, Weldon C, Dalton DL, Kotze A, du Preez LH (2011) Occurrence of *Batrachochytrium dendrobatidis* in amphibian populations of Okomu National Park, Nigeria. Herpetological Review 42(3): 379–382.

Isidoro-Ayza M, Lorch JM, Ballmann AE, Businga NK (2019) Mass mortality of Green Frog (*Rana clamitans*) tadpoles in Wisconsin, USA, associated with severe infection with the pathogenic Perkinsea clade. Journal of Wildlife Diseases 55(1): 262–265. doi: 10.7589/2018-02-046

IUCN (2010) IUCN Red List of Threatened Species. Version 2010.1. Available at: http//www.iucnredlist.org. Downloaded on 15 April 2010.

Jaeger JR, Waddle AW, Rivera R, Harrison DT, Ellison S, Forrest MJ, Vredenburg VT, van Breukelen F (2017) *Batrachochytrium dendrobatidis* and the decline and survival of the Relict Leopard Frog. EcoHealth 14: 285–295. doi: 10.1007/s10393-017-1240-2

Jenkinson TS, Betancourt Román CM, Lambertini C, Valencia-Aquilar A, Rodriguez D, Nunes-de-Almeida CHL, Ruggeri J, Belasen AM, Da Silva Leite D, Zamudio KR, Longcore JE, Toledo LF, James TY (2016) Amphibian-killing chytrid in Brazil comprises both locally endemic and globally expanding populations. Molecular Ecology 25: 2978–2996. doi: 10.1111/mec.13599

Joglar RL, Álvarez AO, Aide TM, Barber D, Burrowes PA et al. (2007) Conserving the Puerto Rican herpetofauna. Applied Herpetology 4: 327–345.

Johnson PTJ, McKenzie VJ, Peterson AC, Kerby JL, Brown J, Blaustein AR, Jackson T (2011) Regional decline of an iconic amphibian associated with elevation, land-use change, and invasive species. Conservation Biology 25(3): 556–566. doi: 10.1111/j.1523-1739.2010.01645.x

Johnson PTJ, Calhoun DM, Stokes AN, Susbilla CB, McDevitt-Galles T, Briggs CJ, Hoverman JT, Tkach VV, de Roode JC (2018) Of poisons and parasites—the defensive role of tetrodotoxin against infections in newts. Journal of Animal Ecology 87: 1192–1204. doi: 10.1111/1365-2656.12816

Jongsma GFM, Bamba Kaya A, Yoga J-A, Mbega J-D, Mve Beh J-H, Tobi E, Emrich AM, Dixon-MacCallum GP, Davis DR, Kerby JL, Blackburn DC (2016) Widespread presence and high prevalence of *Batrachochytrium dendrobatidis* in Gabon. Herpetological Review 47(2): 227–230.

Jongsma GFM, Empey MA, Smith CM, Bennett AM, McAlpine DF (2019) High prevalence of the amphibian pathogen *Batrachochytrium dendrobatidis* in plethodontid salamanders in protected areas in New Brunswick, Canada. Herpetological Conservation and Biology 14(1): 91–96.

Julian JT, Gould VA, Glenney GW, Brooks RP (2016) Seasonal infection rates of *Batrachochytrium dendrobatidis* in populations of northern green frog *Lithobates clamitans melanota* tadpoles. Diseases of Aquatic Organisms 121: 97–104. doi: 10.3354/dao03046

Julian JT, Brooks RP, Glenney GW, Coll JA (2019) State-wide survey of amphibian pathogens in Green Frog (*Lithobates clamitans melanota*) reveals high chytrid infection intensities in constructed wetlands. Herpetological Conservation and Biology 14(1): 199–211.

Kadekaru S, Tamukai K, Tominaga A, Goka K, Une Y (2016) Spontaneous oral chytridiomycosis in wild bullfrog tadpoles in Japan. Journal of Veterinary Medical Science 78(4): 573–577. doi: 10.1292/jvms.15-0486

Kaiser K, Grafe TU (2011) Chytrid fungus not found in preliminary survey of lowland amphibian populations across northwestern Borneo. Herpetological Review 42: 59–61.

Kaiser K, Pollinger J (2012) *Batrachochytrium dendrobatidis* shows high genetic diversity and ecological niche specificity among haplotypes in the Maya Mountains of Belize. PLoS ONE 7(2): e32113. doi: 10.1371/journal.pone.0032113

Kärvemo S, Meurling S, Berger D, Höglund J, Laurila A (2018) Effects of host species and environmental factors on the prevalence of *Batrachochytrium dendrobatidis* in northern Europe. PLoS ONE 13(10): e0199852. https://doi.org.10.1371/journal.pone.0199852 [Karvemo, Hoglund]

Kärvemo S, Laurila A, Höglund J (2019) Urban environment and reservoir host species are associated with *Batrachochytrium dendrobatidis* infection prevalence in the common toad. Diseases of Aquatic Organisms 134: 33–42. https://doi.org/10.3354/dao03359 [Karvemo, Hoglund]

Keitzer SC, Goforth R, Pessier AP, Johnson AJ (2011) Survey for the pathogenic chytrid fungus *Batrachochytrium dendrobatidis* in southwestern North Carolina salamander populations. Journal of Wildlife Diseases 47: 455–458.

Kerby J (2011) Prevalence of an emerging disease in South Dakota amphibian populations. South Dakota Department of Game, Fish and Parks State Wildlife Grants Final Report. Contact: Jacob Kerby, jacob.kerby@usd.edu

Kielgast J, Rödder D, Veith M, Lötters S (2009) Widespread occurrence of the amphibian chytrid fungus in Kenya. Animal Conserv 13, suppl. 1: 1–8.

Kiemnec-Tyburczy KM, Eddy SL, Chouinard AJ, Houck LD (2012) Low prevalence of *Batrachochytrium dendrobatidis* in two plethodontid salamanders from North Carolina, USA. Herpetological Review 43(1): 85–87.

Kik M, Stege M, Boonyarittichaikij R, van Asten A (2012) Concurrent ranavirus and *Batrachochytrium dendrobatidis* infection in captive frogs (*Phyllobates* and *Dendrobates* species), The Netherlands, 2012: a first report. The Veterinary Journal 194: 247–249. http://dx.doi.org/10.1016/j.tfjl.2012.09.016

Kilburn VL, Ibáñez R, Sanjur O, Bermingham E, Suraci JP, Green DM (2010) Ubiquity of the pathogenic chytrid fungus, *Batrachochytrium dendrobatidis*, in anuran communities in Panamá. EcoHealth 7: 537–548. doi: 10.1007/s10393-010-0634-1

Kilburn VL, Ibáñez R, Green DM (2011) Reptiles as potential vectors and hosts of the amphibian pathogen *Batrachochytrium dendrobatidis* in Panama. Diseases of Aquatic Organisms 97: 127–134. doi: 10.3354/dao02409

Kindermann C, Narayan EJ, Hero J-M (2012) Urinary corticosterone metabolites and chytridiomycosis disease prevalence in a free-living population of male Stony Creek frogs (*Litoria wilcoxii*). Comparative Biochemistry and Physiology, Part A 162: 171–176. doi: 10.1016/j.cbpa.2012.02.018

Kindermann C, Narayan EJ, Hero J-M (2017) Does physiological response to disease incur cost to reproductive ecology in a sexually dichromatic amphibian species? Comparative Biochemistry and Physiology, Part A 203: 220-226. http://dx.doi.org/10.1016/j.cbpa.2016.09.019

Kinney VC, Heemeyer JL, Pessier AP, Lannoo MJ (2011) Seasonal pattern of *Batrachochytrium dendrobatidis* infection and mortality in *Lithobates areolatus*: affirmation of Vredenburg’s “10,000 zoospore rule”. PLoS ONE 6(3): e16708. doi: 10.1371/journal.pone.0016708

Klemish JL, Johnson BL, Siddons SR, Wild ER (2012) Occurrence of *Batrachochytrium dendrobatidis* among populations of *Lithobates clamitans* and *L. pipiens* in Wisconsin, USA. Herpetological Review 43(2): 282–288.

Klop-Toker K, Valdez J, Stockwell M, Fardell L, Clulow S, Clulow J, Mahony M (2016) We made your bed, why won’t you lie in it? Food availability and disease may affect reproductive output of reintroduced frogs. PLoS ONE 11(7): e0159143. doi: 10.1371/journal.pone.0159143

Köhler G, Trejo-Pérez RG, Reuber V, Wehrenberg G, Méndez-de la Cruz F (2016) A survey of tadpoles and adult anurans in the Sierra Madre del Sur of Oaxaca, Mexico (Ampibia: Anura). Mesoamerican Herpetology 3(3): 639–660. [Kohler, Trejo-Perez, Mendez-de la Cruz]

Köhler G, Hantke G, Méndez-de la Cruz FR, Flamm A, Eisenberg T (2019a Mexico) A survey for the amphibian chytrid fungus *Batrachochytrium dendrobatidis* in the Mexican states of México, Morelos, Oaxaca, and Puebla. Herpetological Review 50(2): 303–305. [Kohler, Mendez-de la Cruz]

Köhler G, Than NL, Flamm A, Eisenberg T (2019b Myanmar) A preliminary survey for *Batrachochytrium dendrobatidis* in Myanmar. Herpetological Review 50(2): 298–300. [Kohler]

Kolby JE (2014) Presence of the amphibian chytrid fungus *Batrachochytrium dendrobatidis* in native amphibians exported from Madagascar. PLoS ONE 9(3): e89660.

Kolby JE, Padgett-Flohr GE (2009) Reassessment of the historical timeline for *Batrachochytrium dendrobatidis* presence in Honduras and conservation implications for *Plectrohyla dasypus*. Herpetological Review 40: 307–308.

Kolby JE, Padgett-Flohr GE, Field R (2010) Amphibian chytrid fungus *Batrachochytrium dendrobatidis* in Cusuco National Park, Honduras. Diseases of Aquatic Organisms 92: 245–251. doi: 10.3354/dao02055

Kolby JE, Smith KM, Berger L, Karesh WB, Preston A, Pessier AP, Skerratt LF (2014) First evidence of amphibian chytrid fungus (*Batrachochytrium dendrobatidis*) and ranavirus in Hong Kong amphibian trade. PLoS ONE 9(3): e90750. doi: 10.1371/journal.pone.0090750

Kolby JE, Smith KM, Ramirez SD, Rabemananjara F, Pessier AP, Brunner JL, Goldberg CS, Berger L, Skerratt LF. (2015a) Rapid response to evaluate the presence of the amphibian chytrid fungus (*Batrachochytrium dendrobatidis*) and ranavirus in wild amphibian populations in Madagascar. PLoS ONE 10(6): e0125330. doi:10.1371/journal.pone.0125330.

Kolby JE, Ramirez SD, Berger L, Richards-Hrdlicka KL, Jocque M, Skerratt LF (2015b) Terrestrial dispersal and potential environmental transmission of the amphibian chytrid fungus (*Batrachochytrium dendrobatidis*). PLoS ONE 10(4): e0125386. doi: 10.1371/journal.pone.0125386

Kolenda K, Najbar A, Ogielska M, Baláž V (2017) *Batrachochytrium dendrobatidis* is present in Poland and associated with reduced fitness in wild populations of *Pelophylax lessonae*. Diseases of Aquatic Organisms 124: 241–245. https://doi.org/10.3354/dao03121

Korfel CA, Hetherington TE (2014) Temperature alone does not explain patterns of *Batrachochytrium dendrobatidis* infections in the Green Frog *Lithobates clamitans*. Diseases of Aquatic Organisms 109: 177–185. doi: 10.3354/dao02749.

Kosch TA, Morales V, Summers K (2012) *Batrachochytrium dendrobatidis* in Peru. Herpetological Review 43(2): 288–293.

Kriger KM, Hero J-M (2006) Survivorship in wild frogs infected with chytridiomycosis. EcoHealth 3: 171–177.

Kriger KM, Hero J-M (2007a) The chytrid fungus *Batrachochytrium dendrobatidis* is non-randomly distributed across amphibian breeding habitats. Diversity and Distributions 13: 781–788.

Kriger KM, Hero J-M (2007b) *Cophixalus ornatus* (Ornate Nursery Frog). Chytridiomycosis. Herpetological Review 38: 321.

Kriger KM, Hero J-M (2008) Altitudinal distribution of chytrid (*Batrachochytrium dendrobatidis*) infection in subtropical Australian frogs. Austral Ecology 33: 1022–1032. doi: 10.1111/j.1442-9993.2008.01872.x

Kriger KM, Pereoglou F, Hero J-M (2007) Latitudinal variation in the prevalence and intensity of chytrid (*Batrachochytrium dendrobatidis*) infection in eastern Australia. Conservation Biology 21: 1280–1290.

Krynak TJ, Robison TL, Scott JJ (2012) Detection of *Batrachochytrium dendrobatidis* in amphibian populations of northeast Ohio. Herpetological Review 43(1): 87–89.

Kusrini MD, Skerratt LF, Garland S, Berger L, Endarwin W (2008) Chytridiomycosis in frogs of Mount Gede Pangrango, Indonesia. Diseases of Aquatic Organisms 82: 187–194.

Labisko J, Maddock ST, Taylor ML, Chong-Seng L, Gower DJ, Wynne FJ, Wombwell E, Morel C, French GCA, Bunbury N, Bradfield KS (2015) Chytrid fungus (*Batrachochytrium dendrobatidis*) undetected in the two orders of Seychelles amphibians. Herpetological Review 46(1): 41–45.

Lambertini C, Becker CG, Jenkinson TS, Rodriguez D, da Silva Leite D, James TY, Zamudio KR, Toledo LF (2016) Local phenotypic variation in amphibian-killing fungus predicts infection dynamics. Fungal Ecology 20: 15–21. http://dx.doi.org/10.1016/j.fungeco.2015.09.014

Lambertini C, Becker CG, Bardier C, da Silva Leite D, Toledo LF (2017) Spatial distribution of *Batrachochytrium dendrobatidis* in South American caecilians. Diseases of Aquatic Organisms 124: 109–116. https://doi.org/10.3354/dao03114

Lampo M, Señaris JC (2006) Unexplained amphibian mortalities in the secluded mountains of the Venezuelan Guayana: is there evidence of chytridiomycosis? Herpetological Review 37: 47–49.

Lampo M, Rodríguez A, La Marca E, Daszak PA (2006a) A chytridiomycosis epidemic and a severe dry season precede the disappearance of *Atelopus* species from the Venezuelan Andes. Herpetological Journal 16: 395–402.

Lampo M, Barrio-Amorós CL, Han BA (2006b) *Batrachochytrium dendrobatidis* infection in the recently rediscovered *Atelopus mucubajiensis* (Anura, Bufonidae), a critically endangered frog from the Venezuelan Andes. EcoHealth 3: 299–302.

Lampo M, Sánchez D, Nicolás A, Márquez M, Nava-González F, et al. (2008) *Batrachochytrium dendrobatidis* in Venezuela. Herpetological Review 39: 449–454.

Lampo M, Señaris C, Garcia CZ (2017) Population dynamics of the critically endangered toad *Atelopus cruciger* and the fungal disease chytridiomycosis. PLoS ONE 12(6): e0179007. https://doi.org/10.1371/journal.pone.0179007

Lane EP, Weldon C, Bingham J (2003) Histological evidence of chytridiomycete fungal infection in a free-ranging amphibian, *Afrana fuscigula* (Anura: Ranidae) in South Africa. Journal of the South African Veterinary Association 74: 20–21.

Lannoo MJ, Peterson C, Lovich RE, Nanjappa P, Phillips C, Mitchell JC, Macallister I (2011) Do frogs get their kicks on Route 66? Continental U.S. transect reveals spatial and temporal patterns of *Batrachochytrium dendrobatidis* infection. PLoS ONE 6(7): e22211. doi: 10.1371/journal.pone.0022211

Lastra González L, Baláž V, Solský M, Thumsová B, Kolenda K, Najbar A, Najbar B, Kautman M, Chajma P, Balogová M, Vojar J (2019) Recent findings of potentially lethal salamander fungus *Batrachochytrium salamandrivorans*. Emerging Infectious Diseases 25(7): 1416–1418. doi: https://doi.org/10.3201/eid2507.181001 [Gonzalez, Balaz, Solsky, Thumsova, Balogova]

Lauer A, Simon MA, Banning JL, André E, Duncan K, et al. Common cutaneorus bacteria from the eastern red-backed salamander can inhibit pathogenic fungi. Copeia 2007: 630–640.

Laufer G, Gobel N, Borteiro C, Soutullo A, Martínez-Debat C, de Sá RO (2018) Current status of American bullfrog, *Lithobates catesbeianus*, invasion in Uruguay and exploration of chytrid infection. Biological Invasions 20: 285–291. https://doi.org/10.1007/s10530-017-1540-z

Lawson TD, Jones ML, Komar O, Welch AM (2011) Prevalence of *Batrachochytrium dendrobatidis* in *Agalychnis moreletii* (Hylidae) of El Salvador and association with larval jaw sheath depigmentation. Journal of Wildlife Diseaess 47(3): 544–554.

Leblanc J, Faruk A, Dort E, Govindarajulu P, Quah E, Muin MA, Hintz W (2014) Multi-year surveillance for *Batrachochytrium dendrobatidis* in amphibians of Peninsular Malaysia. Herpetological Review 45(4): 603–608.

Lehtinen RM, Kam Y-C, Richards CL (2008) Preliminary surveys for *Batrachochytrium dendrobatidis* in Taiwan. Herpetological Review 39: 317–318.

Lenker MA, Savage AE, Becker CG, Rodriguez D, Zamudio KR (2014) Batrachochytrium dendrobatidis infection dynamics vary seasonally in upstate New York, USA. Diseases of Aquatic Organisms 111: 51–60. doi: 10.3354/dao02760

Lescano JN, Longo S, Robledo G (2013) Chytridiomycosis in endemic amphibians of the mountain tops of the Cordoba and San Luis ranges, Argentina. Diseases of Aquatic Organisms 102: 249-254. doi: 10.3354/dao02551

Lettoof DC, Greenlees MJ, Stockwell M, Shine R (2013) Do invasive cane toads affect the parasite burdens of native Australian frogs? International Journal for Parasitology: Parasites and Wildlife 2:155–164.http://dx.doi.org/10.1016/j.ijppaw.2013.04.002

Lindquist E, Cossel J Jr, Córdoba KL, Salamanca J, Zambana I, McFarland HR, Luthman K (2016) First record of the pathogenic fungus *Batrachochytrium dendrobatidis* in *Hypsiboas riojanus* and *H. callipleura* from central Bolivia (Anura: Hylidae). Herpetological Review 47(3): 400–402.

Lindquist ED, Shin MJ, Cossel JO Jr, Stuckert AMM, Bletz MC, Trimmer NC (2011) Chytrid in a canopy amphibian: Picado’s Bromeliad Treefrog, *Isthmohyla picadoi* (Hylidae), persists at a site affected by *Batrachochytrium dendrobatidis*. Herpetological Review 42(2): 205–208.

Lips KR, Green DE, Papendick R (2003) Chytridiomycosis in wild frogs from southern Costa Rica. Journal of Herpetology 37: 215–218.

Lips KR, Mendelson JR III, Muñoz-Alonso A, Canseco-Márquez L, Mulcahy DG (2004) Amphibian population declines in montane southern Mexico: resurveys of historical localities. Biological Conservation 119: 555–564. doi: 10.1016/j.biocon.2004.01.017

Lips KR, Brem F, Brenes R, Reeve JD, Alford RA et al. (2006) Emerging infectious disease and the loss of biodiversity in a Neotropical amphibian community. Proceedings of the National Academy of Sciences USA 103: 3165–3170.

Loda JL, Otis DL (2009) Low prevalence of amphibian chytrid fungus (*Batrachochytrium dendrobatidis*) in northern leopard frog (*Rana pipiens*) populations of north-central Iowa, USA. Herpetological Review 40: 428–431.

Longcore JR, Longcore JE, Pessier AP, Halteman WA (2007) Chytridiomycosis widespread in anurans of northeastern United States. Journal of Wildlife Management 71: 435–444.

Longo AV, Burrowes PA (2010) Persistence with chytridiomycosis does not assure survival of direct-developing frogs. EcoHealth 7: 185–195. doi: 10.1007/s10393-010-0327-9

Longo AV, Zamudio KR (2017) Temperature variation, bacterial diversity and fungal infection dynamics in the amphibian skin. Molecular Ecology 26: 4787–4797. doi: 10.1111/mec.14220

Longo AV, Burrowes PA, Joglar RL (2010) Seasonality of *Batrachochytrium dendrobatidis* infection in direct-developing frogs suggests a mechanism for persistence. Diseases of Aquatic Organisms 92: 253–260. doi: 10.3354/dao02054

Longo AV, Ossiboff RJ, Zamudio KR, Burrowes PA (2013) Lability in host defenses: terrestrial frogs die from chytridiomycosis under enzootic conditions. Journal of Wildlife Diseases 49: 197–199. doi: 10.7589/2012-05-129

Longo AV, Fleischer RC, Lips KR (2019) Double trouble: co-infections of chytrid fungi will severely impact widely distributed newts. Biological Invasions 21: 2233–2245. https://doi.org/10.1007/s10530-019-01973-3

Lötters S, Schulte R, Cordova JH, Veith M (2005) Conservation priorities for harlequin frogs (*Atelopus* spp.) of Peru. Oryx 39(3): 343–346.

Lötters S, Kielgast J, Sztatecsny M, Wagner N, Schulte U, Werner P, Rödder D, Dambach J, Reissner T, Hochkirch A, Schmidt B (2012) Absence of infection with the amphibian chytrid fungus in the terrestrial Alpine salamander, *Salamandra atra*. Salamandra 48(1): 58–62.

Lötters S, Wagner N, Kerres A, Vences M, Steinfartz S, Sabino-Pinto J, Seufer L, Preissler K, Schultz V, Veith M (2018) First report of host co-infection of parasitic amphibian chytrid fungi. Salamandra 54(4): 287–290.

Love CN, Winzeler ME, Beasley R, Scott DE, Nunziata SO, Lance SL (2016) Patterns of amphibian infection prevalence across wetlands on the Savannah River Site, South Carolina, USA. Diseases of Aquatic Organisms 121: 1–14. Doi: 10.3354/dao03039.

Lovich R, Ryan MJ, Pessier AJ, Claypool B (2008) Infection with the fungus *Batrachochytrium dendrobatidis* in a non-native *Lithobates berlandieri* below sea level in the Coachella Valley, California, USA. Herpetological Review 39: 315–317.

Lowe J (2009) Amphibian chytrid (*Batrachochytrium dendrobatidis*) in post-metamorphic *Rana boylii* in Inner Coast Ranges of central California. Herpetological Review 40: 180–182.

Luja VH, Rodríguez-Estrella, Ratzlaff K, Parra-Olea G, Ramírez-Bautista A (2012) The chytrid fungus *Batrachochytrium dendrobatidis* in isolated populations of the Baja California Treefrog *Pseudacris hypochondriaca curta* in Baja California Sur, Mexico. The Southwestern Naturalist 57(3): 323–327. doi: 10.1894/0038-4909-57.3.323

Luría-Manzano R, Canseco-Márquez L, Frías-Alvarez P (2011) *Batrachochytrium dendrobatidis* in *Plectrohyla arboriscandens (*Anura: Hylidae) larvae at a montane site in the Sierra Negra, Puebla, Mexico. Herpetological Review 42(4): 552–554.

Makange M, Kulaya N, Biseko E, Kalenga P, Mutagwaba S, Misinzo G (2014) *Batrachochytrium dendrobatidis* detected in Kihansi spray toads at a captive breeding facility (Kihansi, Tanzania). Diseases of Aquatic Organisms 111: 159–164. doi: 10.3354/dao02775

Malhotra A, Thorpe RS, Hypolite E, James A (2007) A report on the status of the herpetofauna of the Commonwealth of Dominica, West Indies. Applied Herpetology 4: 177–194.

Mali I, Villamizar-Gomez A, Krizmanić I, Ajtić R (2017) Evidence of *Batrachochytrium dendrobatidis* infection in amphibians from Serbian lowlands. Journal of Wildlife Diseases 53(3): 686–689. doi: 10.7589/2016-07-172

Marhanka EC, Watters JL, Huron NA, McMillin SL, Winfrey CC, Curtis DJ, Davis DR, Farkas JK, Kerby JL, Siler CD (2017) Detection of high prevalence of *Batrachochytrium dendrobatidis* in amphibians from southern Oklahoma, USA. Herpetological Review 48(1): 70–74.

Márquez M, Nava-González F, Sánchez D, Calcagno M, Lampo M (2010) Immunological clearance of *Batrachochytrium dendrobatidis* infection at a pathogen-optimal temperature in the hylid frog Hypsiboas crepitans. EcoHealth 7: 380–388. doi: 10.1007/s10393-010-0350-x [Marquez, Nava-Gonzalez, Sanchez]

Martel A, Fard MS, Van Rooij P, Jooris R, Boone F, Haesebrouck F, Van Rooij D, Pasmans F (2012) Road-killed Common Toads (*Bufo bufo*) in Flanders (Belgium) reveal low prevalence of ranaviruses and *Batrachochytrium dendrobatidis*. Journal of Wildlife Diseases 48(3): 835–839.

Martel A, Adriaensen C, Sharifian-Fard M, Spitzen-van der Sluijs A, Louette G, Baert K, Crombaghs B, Dewulf J, Pasmans F (2013) The absence of zoonotic agents in invasive bullfrogs (*Lithobates catesbeianus*) in Belgium and the Netherlands. EcoHealth 10: 344–347. doi: 10.1007/s10393-013-0864-0

Marshall TL, Baca CR, Correa DT, Forstner MRJ, Hahn D, Rodriguez D (2019) Genetic characterization of chytrids isolated from larval amphibians collected in central and east Texas. Fungal Ecology 39: 55–62. https://doi.org/10.1016/j.funeco.2018.12.001

Mathie AH, Jarrett C, Jhaveri L, Hoskisson PA, Downie JR (2018) First assessment of *Batrachochytrium dendrobatidis* in amphibian populations in the Kanuku Mountains Protected Area of Guyana. Herpetological Bulletin 146: 18–24.

May S, Zeisset I, Beebee TJC (2011) Larval fitness and immunogenetic diversity in chytrid-infected and uninfected natterjack toad (*Bufo calamita*) populations. Conservation Genetics 12: 805–811. doi: 10.1007/s10592-011-0187-z

Mazzoni R, Cunningham AA, Daszak P, Apolo A, Perdomo E, et al. (2003) Emerging pathogen of wild amphibians in frogs (*Rana catesbeiana*) farmed for international trade. Emerging Infectious Diseases 9: 995–998.

McCracken S, Gaertner JP, Forstner MRJ, Hahn D (2009) Detection of *Batrachochytrium dendrobatidis* in amphibians from the forest floor to the upper canopy of an Ecuadorian Amazon lowland rainforest. Herpetological Review 40: 190–195.

McLelland DJ, Smith I, Olds L, Myers C, Skerratt LF (2013) *Batrachochytrium dendrobatidis* not detected in anurans in the Northern Kimberley region of Western Australia. Herpetological Review 44(1): 98–100.

McLeod DS, Sheridan JA, Jiraungkoorskul W, Khonsue W (2008) A survey for chytrid fungus in Thai amphibians. Raffles Bulletin of Zoology 56: 199–204.

McMahon TA, Brannelly LA, Chatfield MWH, Johnson PTJ, Joseph MB, McKenzie VJ, Richards-Zawacki CL, Venesky MD, Rohr JR (2013) Chytrid fungus *Batrachochytrium dendrobatidis* has nonamphibian hosts and releases chemicals that cause pathology in the absence of infection. Proceedings of the National Academy of Sciences USA 110(1): 210–215. www.pnas.org/cgi/doi/10.1073/pnas.1200592110

McMillan KM, Lesbarrères D, Harrison XA, Garner TWJ (2019) Spatiotemporal heterogeneity decouples infection parameters of amphibian chytridiomycosis. Journal of Animal Ecology 89: 1109–1121. doi: 10.1111/1365-2656.13170

McTaggart AL, Eberl T, Keller O, Jameson ML (2014) First report of *Batrachochytrium dendrobatidis* associated with amphibians in Kansas, USA. Herpetological Review 45(3): 439–441.

Medina D, Garner TWJ, Carrascal LM, Bosch J (2015) Delayed metamorphosis of amphibian larvae facilitates *Batrachochytrium dendrobatidis* transmission and persistence. Diseases of Aquatic Organisms 117: 85–92. doi: 10.3354/dao02934

Mendelson JR III, Brodie ED Jr, Malone JH, Acevedo ME, Baker MA, Smatresk NJ, Campbell JA (2005 “2004”) Factors associated with the catastrophic decline of a cloudforest frog fauna in Guatemala. Revista de Biología Tropical (International Journal of Tropical Biology) 52(4): 991–1000.

Mendelson JR III, Jones MEB, Pessier AP, Toledo G, Kabay EH, Campbell JA (2014) On the timing of an epidemic of amphibian chytridiomycosis in the highlands of Guatemala. South American Journal of Herpetology 9(2): 151–153. doi: 10.2994/SAJH-D-14-00021.1

Mendoza JA II, Gaertner JP, Holden J, Forstner MRJ, Hahn D (2011) Detection of *Batrachochytrium dendrobatidis* on amphibians in Pursat Province, Cambodia. Herpetological Review 42(4): 542–545.

Mendoza-Almeralla C, López-Velásquez A, Longo AV, Parra-Olea G (2016) Temperature treatments boost subclinical infections of *Batrachochytrium dendrobatidis* in a Mexican salamander (*Pseudoeurycea leprosa*). Revista Mexicana de Biodiversidad 87: 171–179. http://dx.doi.org/10.1016/j.rmb.2016.01.020

Miaud C, Dejean T, Savard K, Millery-Vigues A, Valentini A, Curt Grand Gaudin N, Garner TWJ (2016) Invasive North American bullfrogs transmit lethal fungus *Batrachochytrium dendrobatidis* infections to native amphibian host species. Biological Invasions 18: 2299–2308. doi: 10.1007/s10530-016-1161-y

Michaels CJ, Rendle M, Gibault C, Lopez J, Garcia G, Perkins MW, Cameron S, Tapley B (2018) *Batrachochytrium dendrobatidis* infection and treatment in the salamanders *Ambystoma andersoni*, *A. dumerilii*, and *A. mexicanum*. Herpetological Journal 28: 87–92.

Miller CA, Tasse Taboue GC, Ekane MMP, Robak M, Sesink Clee PR, Richards-Zawaki CR, Fokam EB, Fuashi NA, Anthony NM (2018) Distribution modeling and lineage diversity of the chytrid fungus *Batrachochytrium dendrobatidis* (*Bd*) in a central African amphibian hotspot. PLoS ONE 13(6): e0199288. doi: https://doi.org/10.1371/journal.pone.0199288

Mitchell JC, Green DE (2002) Chytridiomycosis in two species of ranid frogs in the southeastern United States. Joint Meeting of the American Society of Ichthyologists and Herpetologists, Herpetologists' League, and Society for the Study of Amphibians and Reptiles, 4–8 July 2002, Kansas City, USA.

Moffitt D, Williams LA, Hastings A, Pugh MW, Gangloff MM, Siefferman L (2015) Low prevalence of the amphibian pathogen *Batrachochytrium dendrobatidis* in the southern Appalachian Mountains. Herpetological Conservation and Biology 10(1): 123–126.

Molur S, Krutha K, Paingankar MS, Dahanukar N (2015) Asian strain of *Batrachochytrium dendrobatidis* is widespread in the Western Ghats, India. Diseases of Aquatic Organisms 112: 251–255. doi: 10.3354/dao02804

Monsen-Collar K, Hazard L, Dussa R (2010) Comparison of PCR and RT-PCR in the first report of *Batrachochytrium dendrobatidis* in amphibians in New Jersey, USA. Herpetological Review 41: 460–462.

Montanucci RR (2009) The chytrid fungus in the Red Salamander, *Pseudotriton ruber*, in South Carolina, USA. Herpetological Review 40: 188.

Morehouse EA, James TY, Ganley ARD, Vilgalys R, Berger L, et al. (2003) Multilocus sequence typing suggests the chytrid pathogen of amphibians is a recently emerged clone. Molecular Ecology 12: 395–403.

Morell V (1999) Are pathogens felling frogs? Science 284: 728–731.

Morgan JAT, Vredenburg VT, Rachowicz LJ, Knapp RA, Stice MJ et al. (2007) Population genetics of the frog killing fungus *Batrachochytrium dendrobatidis*. Proceedings of the National Academy of Sciences USA 104: 13845–13850.

Mosher BA, Bailey LL, Muths E, Huyvaert KP (2018) Host-pathogen metapopulation dynamics suggest high elevation refugia for boreal toads. Ecological Applications 28(4): 926–937.

Mowry CB, Keene CM, Prisland SE, Tyler BD, Montgomery AA, Mowry AP, Martin RA, Stevens S, Ellwanger J, Morgan MB (2017) A survey of *Batrachochytrium dendrobatidis* occurrence in amphibians of Walker and Floyd Counties, Georgia, USA. Herpetological Review 48(4): 777–779.

Moyer DC, Weldon C (2006) Chytrid distribution and pathogenicity among frogs of the Udzungwa Mountains, Tanzania. Report for the Wildlife Conservation Society, Bronx, NY, USA.

Muelleman PJ, Montgomery CE (2013) *Batrachochytrium dendrobatidis* in amphibians of northern Calhoun County, Illinois, USA. Herpetological Review 44(4): 614–615.

Muletz C, Caruso NM, Fleischer RC, McDiarmid RW, Lips KR (2014) Unexpected rarity of the pathogen *Batrachochytrium dendrobatidis* in Appalachian *Plethodon* salamanders: 1957–2011. PLoS ONE 9(8): e103728. doi: 10.1371/journal.pone.0103728

Muletz-Wolz CR, Fleischer RC, Lips KR (2019) Fungal disease and temperature alter skin microbiome structure in an experimental salamander system. Molecular Ecology 28: 2917–2931. doi: 10.1111/mec.15122

Murphy BG, Hillman C, Groff JM (2015) Chytridiomycosis in dwarf African frogs *Hymenochirus curtipes*. Diseases of Aquatic Organisms 114: 69–75. doi: 10.3354/dao02851

Murphy PJ, St-Hilaire S, Bruer S, Corn PS, Peterson CR (2009) Distribution and pathogenicity of *Batrachochytrium dendrobatidis* in boreal toads from the Grand Teton area of western Wyoming. EcoHealth 6: 109–120. doi: 10.1007/s10393-009-0230-4s.

Murray KA, Skerratt LF, Speare R, McCallum H (2009) Impact and dynamics of disease in species threatened by the amphibian chytrid fungus, *Batrachochytrium dendrobatidis*. Conservation Biology 23: 1242–1252.

Murray K, Retallick R, McDonald KR, Mendez D, Aplin K, Kirkpatrick P, Berger L, Hunter D, Hines HB, Campbell R, Pauza M, Driessen M, Speare R, Richards SJ, Mahony M, Freeman A, Phillott AD, Hero J-M, Kriger K, Driscoll D, Felton A, Puschendorf R, Skerratt LF (2010) The distribution and host range of the pandemic disease chytridiomycosis in Australia, spanning surveys from 1956–2007. Ecology 91(5): 1557. Ecological Archives E091–108. http://esapubs.org/archive/ecol/E091/108/

Murray KA, Skerratt LF, Garland S, Kriticos D, McCallum H (2013) Whether the weather drives patterns of endemic amphibian chytridiomycosis: a pathogen proliferation approach. PLoS ONE 8(4): e61061. doi: 10.1371/journal.pone.0061061

Murrieta-Galindo R, Parra-Olea G, González-Romero A, López-Barrera F, Vredenburg VT (2014) Detection of *Batrachochytrium dendrobatidis* in amphibians inhabiting cloud forests and coffee agroecosystems in central Veracruz, Mexico. European Journal of Wildlife Research 60: 431–439. doi: 10.1007/s10344-014-0800-9

Muths E, Corn PS, Pessier AP, Green DE (2003) Evidence for disease-related amphibian decline in Colorado. Biological Conservation 110: 357–365.

Muths E, Pilliod DS, Livo LJ (2008) Distribution and environmental limitations of an amphibian pathogen in the Rocky Mountains, USA. Biological Conservation 141: 1484–1492.

Muths E, Spurre Pedersen B, Spurre Pedersen F (2009) How relevant is opportunistic *Bd* sampling: Are we ready for the big picture? Herpetological Review 40: 183–184.

Mutnale MC, Anand S, Eluvathingal LM, Roy JK, Reddy GS, Vasudevan K (2018) Enzootic frog pathogen *Batrachochytrium dendrobatidis* in Asian tropics reveals high ITS haplotype diversity and low prevalence. Scientific Reports 8: 10125 [article number, not pages]. doi:10.1038/s41598-018-28304-1

Mutschmann F, Berger L, Zwart P, Gaedicke C (2000) [Chytridiomycosis in amphibians—first report in Europe] Berl Munch Tierarztl Wochenschr 113: 380–383. [In German].

Nair A, Daniel O, Gopalan SV, George S, Kumar KS, Merilä J, Teacher AGF (2011) Infectious disease screening of *Indirana* frogs from the Western Ghats biodiversity hotspot. Herpetological Review 42(4): 544–557.

Narayan E, Molinia F, Hero J-M (2011) Absence of invasive chytrid fungus (*Batrachochytrium dendrobatidis*) in native Fijian ground frog (*Platymantis vitiana*) populations on Viwa-Tailevu, Fiji Islands. Acta Herpetologica 6(2): 261–266.

Narayan EJ, Graham C, McCallum H, Hero J-M (2014) Over-wintering tadpoles of Mixophyes fasciolatus act as reservoir host for *Batrachochytrium dendrobatidis*. PLoS ONE 9(3): e92499. doi: 10.1371/journal.pone.0092499

Navarro-Lozano A, Sánchez-Domene D, Rossa-Freres DC, Bosch J, Sawaya RJ (2018) Are oral deformities in tadpoles accurate indicators of anuran chytridiomycosis ? PLoS ONE 13(1): e0190955. https://doi.org/10.1371/journal.pone.0190955

Newman JC, Mota JL, Hardman RH, Dillman JW, Barrett K (2019) Pathogen detection in Green Salamanders (*Aneides aeneus*) in South Carolina, USA. Herpetological Review 50(3): 503–505.

Nicolás A (2007) Tendencia altitudinal de infección en un hongo patógeno en ranas *Mannophryne herminae* del Parque Nacional Henri Pittier. Undergraduate thesis. Universidad Simón Bolívar, Caracas, Venezuela.

North S, Alford RA (2008) Infection intensity and sampling locality affect *Batrachochytrium dendrobatidis* distribution among body regions on green-eyed tree frogs *Litoria genimaculata*. Diseases of Aquatic Organisms 81: 177–188. doi: 10.3354/dao01958

Obendorf DL (2005) Application of field and diagnostic methods for chytridiomycosis in Tasmanian frogs. Central North Field Naturalists, Inc., Tasmania, Australia.

Obendorf DL, Dalton A (2006) A survey for the presence of the amphibian chytrid fungus (*Batrachochytrium dendrobatidis*) in Tasmania. Papers and Proceedings of the Royal Society of Tasmania 140: 25–29.

Obon E, Carbonell F, Valbuena-Ureña E, Alonso M, Larios R, Fernández-Beaskoetxea S, Fisher MC, Bosch J (2013) Chytridiomycosis surveillance in the critically endangered Montseny brook newt, *Calotriton arnoldi*, northeastern Spain. Herpetological Journal 23: 237–240.

Ocock JF, Rowley JJL, Penman TD, Rayner TS, Kingsford RT (2013) Amphibian chytrid prevalence in an amphibian community in arid Australia. EcoHealth 10(1): 77–81. doi: 10.1007/s10393-013-0824-8

Oficialdegui FJ, Sánchez MI, Monsalve-Carcaño C, Boyero L, Bosch J (2019) The invasive red swamp crayfish (*Procambarus clarkii*) increases infection of the amphibian chytrid fungus (*Batrachochytrium dendrobatidis*). Biological Invasions 21: 3221–3231. https://doi.org/10.1007/s10530-019-02041-6

Ohst T, Gräser Y, Mutschmann F, Plötner J (2011) Neue Erkenntnisse zur Gefährdung europäischer Amphibien durch den Hautpilz. Zeitschrift für Feldherpetologie 18: 1–17. [these data reported again in Ohst et al. 2013]

Ohst T, Gräser Y, Plötner J (2013) *Batrachochytrium dendrobatidis* in Germany: distribution, prevalences, and prediction of high risk areas. Diseases of Aquatic Organisms 107: 49–59. doi: 10.3354/dao02662

Olori JC, Netzband R, McKean N, Lowery J, Parsons K, Windstam ST (2018) Multi-year dynamics of ranavirus, chytridiomycosis, and co-infections in a temperate host assemblage of amphibians. Diseases of Aquatic Organisms 130: 187–197. https://doi.org/10.3354/dao03260

Ouellet M, Mikaelian I, Pauli BD, Rodrigue J, Green DE (2005) Historical evidence of wide-spread chytrid infection of North American amphibian populations. Conservation Biology 19: 1431–1440.

Ouellet M, Dejean T, Galois P (2012) Occurrence of the amphibian chytrid fungus *Batrachochytrium dendrobatidis* in introduced and native species from two regions of France. Amphibia-Reptilia 33: 415–422. doi: 10.1163/15685381-00002845

Padgett-Flohr GE, Longcore JE (2005) *Ambystoma californiense* (California Tiger Salamander). Fungal infection. Herpetological Review 36: 50–51.

Padgett-Flohr GE, Longcore JE (2007) *Taricha torosa* (California Newt). Fungal infection. Herpetological Review 38: 176–177.

Padgett-Flohr GE, Hopkins RL III (2009) *Batrachochytrium dendrobatidis*, a novel pathogen approaching endemism in central California. Diseases of Aquatic Organisms 83: 1–9. doi: 10.3354/dao02003

Padgett-Flohr GE, Hopkins RL III (2010) Landscape epidemiology of *Batrachochytrium dendrobatidis* in central California. Ecography 33(4): 688–697. doi: 10.1111/j.1600-0587.2009.05994.x

Paetow LJ, McLaughlin JD, Cue RI, Pauli BD, Marcogliese DJ (2012) Effects of herbicides and the chytrid fungus *Batrachochytrium dendrobatidis* on the health of post-metamorphic northern leopard frogs (*Lithobates pipiens*). Ecotoxicology and Environmental Safety 80: 372–380. http://dx.doi.org/10.1016/j.ecoenv.2012.04.006

Parker JM, Mikaelian I, Hahn N, Diggs HE (2002) Clinical diagnosis and treatment of epidermal chytridiomycosis in African clawed frogs (*Xenopus tropicalis*). Comparative Medicine 52: 265–268.

Pasmans F, Zwart P, Hyatt AD (2004) Chytridiomycosis in the Central American bolitoglossine salamander (*Bolitoglossa dofleini*). Veterinary Record 154: 153. doi: 10.1136/vr.154.5.153

Pasmans F, Muijsers M, Maes S, Van Rooij P, Brutyn M, Ducatelle R, Haesbrouck F, Martel A (2010) Chytridiomycosis related mortality in a midwife toad (*Alytes obstetricans*) in Belgium. Vlaams Diergeneeskundig Tijdschrift 79(6): 460–462. [only abstract available online]

Pasmans F, Van Rooij P, Blooi M, Tessa G, Bogaerts S, Sotgiu G, Garner TWJ, Fisher MC, Schmidt BR, Woeltjes T, Beukema W, Bovero S, Adriaensen C, Oneto F, Ottonello D, Martel A, Salvidio S (2013) Resistance to chytridiomycosis in European plethodontid salamanders of the genus *Speleomantes*. PLoS ONE 8(5): e63639. doi: 10.1371/journal.pone.0063639

Patel YA, Cavin JN, Moore MK (2012) Morphological anomalies as indicators of chytrid infection in *Bufo marinus* from Trinidad, West Indies. Bios 83(3): 75–80. doi: 10.1893/0005-3155-83.3.75

Patillo BE, Parris MJ (2016) High prevalence of *Batrachochytrium dendrobatidis* in *Notophthalmus viridescens* in the Ozark National Forest and Harold E. Alexander Wildlife Management Area, Arkansas, USA. Herpetological Review 47(2): 210–211.

Patrelle C, Miaud C, Cristina N, Kulberg P, Merilä J (2012) Chytrid fungus screening in a population of Common Frogs from northern Finland. Herpetological Review 43(3): 422–425.

Pauza M, Driessen M (2008) Distribution and potential spread of amphibian chytrid fungus *Batrachochytrium dendrobatidis* in the Tasmanian Wilderness World Heritage Area. Report to the Biodiversity Conservation Branch, Department of Primary Industries and Water, Tasmania. February 2008.

Pauza MD, Driessen MM, Skerratt LF (2010) Distribution and risk factors for spread of amphibian chytrid fungus *Batrachochytrium dendrobatidis* in the Tasmanian Wilderness World Heritage Area, Australia. Diseases of Aquatic Organisms 92: 193–199. doi: 10.3354/dao02212

Pearl CA, Green DE (2005) *Rana catesbeiana* (American Bullfrog) Chytridiomycosis. Herpetological Review 36: 305.

Pearl CA, Bull EL, Green DE, Bowerman J, Adams MJ, et al. (2007) Occurrence of the amphibian pathogen *Batrachochytrium dendrobatidis* in the Pacific Northwest. Journal of Herpetology 41: 145–149.

Pearl CA, Bowerman J, Adams MJ, Chelgren ND (2009) Widespread occurrence of the chytrid fungus *Batrachochytrium dendrobatidis* on Oregon Spotted Frogs (*Rana pretiosa*). EcoHealth 6: 209–218. doi: 10.1007/s10393-009-0237-x

Penner J, Adum GB, McElroy MT, Doherty-Bone T, Hirschfeld M, Sandberger L, Weldon C, Cunningham AA, Ohst T, Wombwell E, Portik DM, Reid D, Hillers A, Ofori-Boateng C, Oduro W, Plötner J, Ohler A, Leaché AD, Rödel MO (2013) West Africa—a safe haven for frogs? A sub-continental assessment of the chytrid fungus (*Batrachochytrium dendrobatidis*). PLoS ONE 8(2): e56236. doi: 10.1371/journal.pone.005236

Peralta-García A, Valdez-Villavicencio JH, Galina-Tessaro P (2014) African Clawed Frog (*Xenopus laevis*) in Baja California: a confirmed population and possible ongoing invasion in Mexican watersheds. Southwestern Naturalist 59(3): 431–434.

Peralta-García A, Adams AJ, Briggs CJ, Galina-Tessaro P, Valdez-Villavicencio JH, Hollingsworth BD, Shaffer HB, Fisher RN (2018) Occurrence of *Batracochytrium dendrobatidis* in anurans of the Mediterranean region of Baja California, México. Diseases of Aquatic Organisms 127: 193–200. https://doi.org/10.3354/dao03202.

Pessier AP, Nichols DK, Longcore JE, Fuller MS (1999) Cutaneous chytridiomycosis in poison dart frogs (*Dendrobates* spp.) and White's tree frogs (*Litoria caerulea*). Journal of Veterinary Diagnostic Investigation 11: 194–199.

Petersen CE, Lovich RE, Phillips CA, Dreslik MJ, Lannoo MJ (2016) Prevalence and seasonality of the amphibian chytrid fungus *Batrachochytrium dendrobatidis* along widely separated longitudes across the United States. EcoHealth 13: 368–382. doi: 10.1007/s10393-016-1101-4

Peterson AC, McKenzie VJ (2014) Investigating the differences across host species and scales to explain the distribution of the amphibian pathogen *Batrachochytrium dendrobatidis*. PLoS ONE 9(9): e107441. doi: 10.1371/journal.pone.0107441

Peterson JD, Wood MB, Hopkins WA, Unrine JM, Mendonça MT (2007) Prevalence of *Batrachochytrium dendrobatidis* in American Bullfrog and Southern Leopard Frog larvae from wetlands on the Savannah River Site, South Carolina. Journal of Wildlife Diseases 43(3): 450–460.

Phillips CA, Wesslund NA, MacAllister IE (2014) Occurrence of the chytrid fungus *Batrachochytrium dendrobatidis* in amphibians in Illinois, USA. Herpetological Review 45(2): 238–240.

Phillott AD, Grogan LF, Cashins SD, McDonald KR, Berger L, Skerratt LF (2013) Chytridiomycosis and seasonal mortality of tropical stream-associated frogs 15 years after introduction of *Batrachochytrium dendrobatidis.* Conservation Biology 27: 1058–1068. doi: 10.1111/cobi.12073

Picco AM, Collins JP (2007) Fungal and viral pathogen occurrence in Costa Rican amphibians. Journal of Herpetology 41: 746–749.

Pilliod DS, Muths E, Scherer RD, Bartelt PE, Corn PS, et al. (2010) Effects of amphibian chytrid fungus on individual survival probability in wild boreal toads. Conservation Biology 24: 1259–1267.

Piovia-Scott J, Pope KL, Lawler SP, Cole EM, Foley JE (2011) Factors related to the distribution and prevalence of the fungal pathogen *Batrachochytrium dendrobatidis* in *Rana cascadae* and other amphibians in the Klamath Mountains. Biological Conservation 144: 2913–2921. doi: 10.1016/j.biocon.2011.08.008

Polasik JS, Murphy MA, Abbott T, Vincent K (2016) Factors limiting early life stage survival and growth during endangered Wyoming Toad reintroductions. Journal of Wildlife Management 80(3): 540–552. doi: 10.1002/jwmg.1031

Puschendorf, R (2003) *Atelopus varius* (Harlequin Frog). Fungal infection. Herpetological Review 34: 355.

Puschendorf R, Bolanos F, Chaves G. (2006a) The amphibian chytrid fungus along an altitudinal transect bore the first reported declines in Costa Rica. Biological Conservation 132: 136–142.

Puschendorf R, Castañeda F, McCranie J (2006b) Chytridiomycosis in wild frogs from Pico Bonito National Park, Honduras. EcoHealth 3: 178–181.

Puschendorf R, Carnaval AC, VanDerWal J, Zumbado-Ulate H, Chaves G et al. (2009) Distribution models for the amphibian chytrid *Batrachochytrium dendrobatidis* in Costa Rica: proposing climatic refuges as a conservation tool. Diversity and Distributions 15: 401–408.

Puschendorf R, Hoskin CJ, Cashins SD, McDonald K, Skerratt LF, Vanderwal J, Alford RA. (2011) Environmental refuge from disease-driven amphibian extinction. Conservation Biology 25: 956–964. doi: 10.1111/j.1523-1739.2011.01728.x

Puschendorf R, Hodgson L, Alford RA, Skerratt LF, VanDerWal J (2013) Underestimated ranges and overlooked refuges from amphibian chytridiomycosis. Diversity and Distributions 19: 1313-1321. doi: 10.1111/ddi.12091

Rabemananjara FCE, Andreone F, Rabibisoa N (2011) Madagascar and chytrid news: needed an urgent action and close collaboration between stakeholders. FrogLog 97: 33.

Rachowicz LJ, Knapp RA, Morgan JAT, Stice MJ, Vredenburg VT, Parker JM, Briggs CJ (2006) Emerging infectious disease as a proximate cause of amphibian mass mortality. Ecology 87(7): 1671–1683.

Raffel TR, Michel PJ, Sites EW, Rohr JR (2010) What drives chytrid infections in newt populations? Associations with substrate, temperature, and shade. EcoHealth 7: 526–536. doi: 10.1007/s10393-010-0358-2

Ramesh R, Lord A, Griffis-Kyle K, Perry G, Hamilton D, Silva S (2013) Amphibian populations in Brazos River basin, Texas, show no evidence of *Bd* infection. Herpetological Review 44(3): 461–464.

Rasmussen C, Eisenberg T, Alfermann D, Köhler J (2012) Presence of *Batrachochytrium dendrobatidis* in amphibians from central and southern Hesse, central Germany: results from a preliminary regional screening. Salamandra 48(3): 166–172.

Raverty S, Reynolds T (2001) Cutaneous chytridiomycosis in dwarf aquatic frogs (*Hymenochirus boettgeri*) originating from southeast Asia and in a western toad (*Bufo boreas*) from northeastern British Columbia. Canadian Veterinary Journal 42: 385–386.

Rebollar EA, Hughey MC, Harris RN, Domangue RJ, Medina D, Ibáñez R, Belden LK (2014) The lethal fungus *Batrachochytrium dendrobatidis* is present in lowland tropical forests of far eastern Panama. PLoS ONE 9(4): e95484. doi: 10.1371/journal.pone.0095454

Reeder NMM, Cheng TL, Vredenburg VT, Blackburn DC (2011) Survey of the chytrid fungus *Batrachochytrium dendrobatidis* from montane and lowland frogs in eastern Nigeria. Herpetology Notes 4: 83–86.

Reeder NMM, Pessier AP, Vredenburg VT (2012) A reservoir species for the emerging amphibian pathogen *Batrachochytrium dendrobatidis* thrives in a landscape decimated by disease. PLoS ONE 7(3): e33567. doi: 10.1371/journal.pone.0033567

Reeves MK (2008) *Batrachochytrium dendrobatidis* in wood frogs (*Rana sylvatica*) from three National Wildlife Refuges in Alaska, USA. Herpetological Review 39: 68–70.

Reeves MK, Green DE (2006) *Rana sylvatica* (Wood Frog). Chytridiomycosis. Herpetological Review 37: 450.

Reeves RA, Pierce CL, Vandever MW, Muths E, Smalling KL (2017) Amphibians, pesticides, and the amphibian chytrid fungus in restored wetlands in agricultural landscapes. Herpetological Conservation and Biology 12: 68–77.

Regester KJ, Simpson H, Chapman EJ, Petokas PJ (2012) Occurrence of the fungal pathogen *Batrachochytrium dendrobatidis* among Eastern Hellbender populations (*Cryptobranchus a. alleganiensis*) within the Allegheny-Ohio and Susquehanna River drainages, Pennsylvania, USA. Herpetological Review 43(1): 90–93.

Regester KJ, Eisenman HC, Stragand JL, Brooks CS (2016) First reported detection of the amphibian pathogens *Batrachochytrium dendrobatidis* and Ranavirus in the Common Mudpuppy (*Necturus m. maculosus*). Herpetological Review 47(3): 397–400.

Rendle M, Tapley B, Perkins M, Bittencourt-Silva G, Gower DJ, Wilkinson M (2015) Itraconazole treatment of *Batrachochytrium dendrobatidis* (*Bd*) infection in captive caecilians (Amphibia: Gymniophiona) and the first case of *Bd* in a wild neotropical caecilian. Journal of Zoo and Aquarium Research 3(4): 137–140.

Reshetnikov AN, Chestnut T, Brunner JL, Charles K, Nebergall EE, Olson DH (2013) Detection of the emerging amphibian pathogens *Batrachochytrium dendrobatidis* and *Ranavirus* in Russia. Diseases of Aquatic Organisms 110: 235–240. Doi: 10.3354/dao02757

Retallick RWR, McCallum H, Speare R (2004) Endemic infection of the amphibian chytrid fungus in a frog community post-decline. PLoS Biology 2: 1965–1971.

Richards CL, Zellmer AJ, Martens LM (2008) *Batrachochytrium dendrobatidis* not detected in *Oophaga pumilio* on Bastimentos Island, Panama. Herpetological Review 39: 200–202.

Richards-Hrdlicka KL (2013) Preserved specimens of the extinct Golden Toad of Monteverde (*Cranopsis periglenes*) tested negative for the amphibian chytrid fungus (*Batrachochytrium dendrobatidis*). Journal of Herpetology 47(3): 456–458. doi: 10.1670/11-243

Richards-Hrdlicka KL, Richardson JL, Mohabir L (2013) First survey for the amphibian chytrid fungus *Batrachochytrium dendrobatidis* in Connecticut (USA) finds widespread prevalence. Diseases of Aquatic Organisms 102: 169–180. doi: 10.3354/dao02552

Richards-Zawacki CL (2010) Thermoregulatory behavior affects prevalence of chytrid fungal infection in a wild population of Panamanian golden frogs. Proceedings of the Royal Society B – Biological Sciences 277: 519–528. doi: 10.1098/rspb.2009.1656.

Richardson JML, Govindarajulu P, Anholt BR (2014) Distribution of the disease pathogen *Batrachochytrium dendrobatidis* in non-epidemic amphibian communities of western Canada. Ecography 37: 883–893. doi: 10.1111/ecog.00360

Riley K, Berry OF, Roberts JD (2013) Do global models predicting environmental suitability for the amphibian fungus, *Batrachochytrium dendrobatidis*, have local value to conservation managers? Journal of Applied Ecology 50(3): 713–720.

Rimer RL, Briggler JT (2010) Occurrence of the amphibian chytrid fungus (*Batrachochytrium dendrobatidis*) in Ozark caves, Missouri, USA. Herpetological Review 41: 175–177.

Rios-Sotelo G, Figueroa-Valenzuela R, Vredenburg VT (2018) Retrospective survey reveals extreme rarity of amphibian fungal pathogen *Batrachochytrium dendrobatidis* in Japanese amphibians from 1890–1990s. Herpetological Review 49(2): 247–252.

Rittmann SE, Muths E, Green DE (2003) *Pseudacris triseriata* (Western Chorus Frog) and *Rana sylvatica* (Wood Frog). Chytridiomycosis. Herpetological Review 34: 53.

Rivera B, Cook K, Andrews K, Atkinson MS, Savage AE (2019) Pathogen dynamics in an invasive frog compared to native species. EcoHealth 16: 222–234. https://doi.org/10.1007/s10393-019-01432-4

Rizkalla CE (2009) First reported detection of *Batrachochytrium dendrobatidis* in Florida, USA. Herpetological Review 40: 189–190.

Rizkalla CE (2010) Increasing detections of *Batrachochytrium dendrobatidis* in central Florida, USA. Herpetological Review 41: 180–181.

Robinson CW, McNulty SA, Titus VR (2018) No safe space: prevalence and distribution of *Batrachochytrium dendrobatidis* in amphibians in a highly-protected landscape. Herpetological Conservation and Biology 13(2): 373–382.

Rodriguez D, Becker CG, Pupin NC, Haddad CFB, Zamudio KR (2014) Long-term endemism of two highly divergent lineages of the amphibian-killing fungus in the Atlantic Forest of Brazil. Molecular Ecology 23: 774–787. doi: 10.1111/mec.12615. Data file from Dryad.

Rodriguez EM, Gamble T, Hirt MV, Cotner S. (2009) Presence of *Batrachochytrium dendrobatidis* at the headwaters of the Mississippi River, Itasca State Park, Minnesota, USA. Herpetological Review 40: 48–50.

Rodríguez-Brenes S, Rodriguez D, Ibáñez R, Ryan MJ (2016) Spread of amphibian chytrid fungus across lowland populations of Túngara Frogs in Panamá. PLoS ONE 11(5): e0155745. doi: 10.1371/journal.pone.0155745.

Rodríguez-Contreras A, Señaris JC, Lampo M, Rivero R (2008) Rediscovery of *Atelopus cruciger* (Anura: Bufonidae) with notes on its current status in the Cordillera de La Costa, Venezuela. Oryx 42(2): 301–304. doi: 10.1017/S0030605308000082

Rogers KB, Banulis T (2004) Repatriation of boreal toads *Bufo boreas* on the Grand Mesa, Colorado, In Rogers KB (editor) Boreal Toad Research Report: 2003, pp. 2–12. Colorado Division of Wildlife, Fort Collins, CO, USA. Available from: http://wildlife.state.co.us/Research/Aquatic/BorealToad/, or http://wildlife.state.co.us/NR/rdonlyres/1013C627-855D-4AEC-9D74-278847451617/0/2003BUBOreport.pdf, accessed 24 September 2009.

Rollins AW, Copeland JE, Barker H, Satterfield D (2013) The distribution of *Batrachochytrium dendrobatidis* across the southern Appalachian states, USA. Mycosphere 4(2): 250–254. Doi: 10.5943/mycosphere/4/2/7

Ron SR, Merino-Viteri A (2000) Amphibian declines in Ecuador: overview and first report of chytridiomycosis from South America. Froglog 42: 2–3.

Rosa GM, Anza I, Moreira PL, Conde J, Martins F, Fisher MC, Bosch J (2013) Evidence of chytrid-mediated population declines in common midwife toad in Serra da Estrela, Portugal. Animal Conservation 16: 306–315. doi: 10.1111/j.1469-1795.2012.00602.x

Rosa GM, Sabino-Pinto J, Laurentino TG, Martel An, Pasmans F, Rebelo R, Griffiths RA, Stöhr AC, Marschang RE, Price SJ, Garner TWJ, Bosch J (2017) Impact of asynchronous emergence of two lethal pathogens on amphibian assemblages. Scientific Reports 7: 43260. doi: 10.1038/srep43260.

Rosen PC, Schwalbe CR (2002) Final report to AGFD [Arizona Game and Fish Department] Heritage Program (IIPAM I99016), and USFWS [U.S. Fish and Wildlife Service].

Ross L, Wright M, Wiskirchen K, Grace J, Lennon C, Mantooth J, Schenider D, Hudman SP, Kelrick MI, Montgomery CE (2014) Prevalence of *Batrachochytrium dendrobatidis* in three frog species of the Bighorn National Forest, Wyoming, USA. Herpetological Review 45(4): 615–616.

Roth T, Foley J, Worth J, Piovia-Scott J, Pope K, Lawler S (2013) Bacterial flora on Cascades frogs in the Klamath Mountains of California. Comparative Immunology, Microbiology and Infectious Diseases 36: 591–598. DOI: http://dx.doi.org/10.1016/j.cimid.2013.07.002

Rothermel BB, Walls SC, Mitchell JC, Dodd CK, Irwin LK, et al. (2008) Widespread occurrence of the amphibian chytrid fungus (*Batrachochytrium dendrobatidis*) in the southeastern United States. Diseases of Aquatic Organisms 82: 3–18.

Rothermel BB, Travis ER, Miller DL, Hill RL, McGuire JL, Yabsley MJ (2013) High occupancy of stream salamanders despite high Ranavirus presence in a Southern Appalachians watershed. EcoHealth 10: 184–189. doi: 10.1007/s10393-013-0843-5

Rothermel BB, Miller DL, Travis ER, Gonyor McGuire JL, Jensen JB, Yabsley MJ (2016) Disease dynamics of red-spotted newts and their anuran prey in a montane pond community. Diseases of Aquatic Organisms 118: 113–127. doi: 10.3354/dao02965

Rovito SM, Parra-Olea G, Vásquez-Almazán CR, Papenfuss TJ, Wake DB (2009) Dramatic declines in neotropical salamander populations are an important part of the global crisis. Proceedings of the National Academy of Sciences USA 106: 3231–3236.

Rowley JJL, Alford RA (2013) Hot bodies protect amphibians against chytrid infection in nature. Scientific Reports 3: 1515. doi: 10.1038/srep01515

Rowley JJL, Chan SKF, Tang WS, Speare R, Skerratt LF, et al. (2007 Hong Kong) Survey for the amphibian chytrid *Batrachochytrium dendrobatidis* in Hong Kong in native amphibians and in the international amphibian trade. Diseases of Aquatic Organisms 78: 87–95. doi:10.3354/dao01861

Rowley JJL, Skerratt LF, Alford RA, Campbell R (2007 Australia) Retreat sites of rain forest stream frogs are not a reservoir for *Batrachochytrium dendrobatidis* in northern Queensland, Australia. Diseases of Aquatic Organisms 74: 7–12.

Rowley JJL, Hoang HD, Le DTT, Dau VQ, Neang T, Cao TT (2013) Low prevalence or apparent absence of *Batrachochytrium dendrobatidis* infection in amphibians from sites in Vietnam and Cambodia. Herpetological Review 44(3): 466–469.

Roznik EA, Sapsford SJ, Pike DA, Schwarzkopf L, Alford RA (2015) Condition-dependent reproductive effort in frogs infected by a widespread pathogen. Proceedings of the Royal Society B 282: 20150694. http://dx.doi.org/10.1098/rspb.2015.0694

Ruano-Fajardo G, Toledo LF, Mott T (2016) Jumping into a trap: high prevalence of chytrid fungus in the preferred microhabitats of a bromeliad-specialist frog. Diseases of Aquatic Organisms 121: 223–232. doi: 10.3354/dao03045

Rubio AO, Kupferberg SJ, Vargas García, Ttito A, Shepack A, Catenazzi A (2018) Widespread occurrence of the antifungal cutaneous bacterium *Janthinobacterium lividum* on Andean water frogs threatened by fungal disease. Diseases of Aquatic Organisms 131: 233–238. https://doi.org/10.3354/dao03298.

Ruggeri J, Longo AV, Gaiarsa MP, Alencar LRV, Lambertini C, Leite DS, Carvalho-e-Silva SP, Zamudio KR, Toledo LF, Martins M (2015) Seasonal variation in population abundance and chytrid infection in stream-dwelling frogs of the Brazilian Atlantic Forest. PLoS ONE 10(7): e0130554. doi: 10.1371/journal.pone.0130554

Ruggeri J, Toledo LF, de Carvalho-e-Silva SP (2018a) Stream tadpoles present high prevalence but low infection loads of *Batrachochytrium dendrobatidis* (Chytridiomycota). Hydrobiologia 806: 303–311. https://doi.org/10.1007/s10750-017-3367-0

Ruggeri J, de Carvalho-e-Silva SP, James TY, Toledo LF (2018b) Amphibian chytrid infection is influenced by rainfall seasonality and water availability. Diseases of Aquatic Organisms 127: 107–115. https://doi.org/10.3354/dao03191

Ruiz A, Rueda-Almonacid JVR (2008) *Batrachochytrium dendrobatidis* and chytridiomycosis in anural amphibians of Colombia. EcoHealth 5: 27–33.

Russell DM, Goldberg CS, Waits LP, Rosenblum EB (2010) *Batrachochytrium dendrobatidis* infection dynamics in the Columbia spotted frog *Rana luteiventris* in north Idaho, USA. Diseases of Aquatic Organisms 92:223–230. doi: 10.3354/dao02286

Russell ID, Larson JG, von May R, Holmes IA, James TY, Davis Rabosky AR (2019) Widespread chytrid infection across frogs in the Peruvian Amazon suggests critical role for low elevation in pathogen spread and persistence. PLoS ONE 14(10): e0222718. https://doi.org/10.1371/journal.pone.0222718

Russell RE, Halstead BJ, Mosher BA, Muths E, Adams MJ, Grant EHC, Fisher RN, Kleeman PM, Backlin AR, Pearl CA, Honeycutt RK, Hossack BR (2019) Effect of amphibian chytrid fungus (*Batrachochytrium dendrobatidis*) on apparent survival of frogs and toads in the western USA. Biological Conservation 236: 296–304. https://doi.org/10.1016/j.biocon.2019.05.017

Ryan MJ, Latella IM, Painter CW, Giermakowski JT, Christman BL, Jennings RD, Voyles JL (2014) First record of *Batrachochytrium dendrobatidis* in the Arizona Toad (*Anaxyrus microscaphus*) in southwestern New Mexico, USA. Herpetological Review 45(4): 616–618.

Sabino-Pinto J, Bletz MC, Iturriaga M, Vences M, Rodríguez A (2017) Low infection prevalence of the amphibian chytrid fungus *Batrachochytrium dendrobatidis* (Chytridiomycetes: Rhizophydiales) in Cuba. Amphibia-Reptilia 38: 243–249. doi: 10.1163/15685381-00003100

Sacerdote-Velat A, Manjerovic MB, Santymire R (2016) Preliminary survey of *Batrachochytrium dendrobatidis* in the Chicago Region of Illinois, USA. Herpetological Review 47(1): 57–58.

Sadinski W, Roth M, Treleven S, Theyerl J, Dummer P (2010) Detection of the chytrid fungus, *Batrachochytrium dendrobatidis*, on recently metamorphosed amphibians in the north-central United States. Herpetological Review 41: 170–175.

Saenz D, Adams CK, Pierce JB, Laurencio D (2009) Occurrence of *Batrachochytrium dendrobatidis* in an anuran community in the southeastern Talamanca region of Costa Rica. Herpetological Review 40: 311–313.

Saenz D, Kavanagh BT, Kwiatkowski MA (2010) *Batrachochytrium dendrobatidis* detected in amphibians from national forests in eastern Texas, USA. Herpetological Review 41: 47–79.

Saenz D, Hall TL, Kwiatkowski MA (2015) Effects of urbanization on the occurrence of *Batrachochytrium dendrobatidis*: do urban environments provide refuge from the amphibian chytrid fungus? Urban Ecosystems 18: 333–340. doi: 10.1007/s11252-014-0398-4

Sánchez D, Chacón-Ortiz A, León F, Han BA, Lampo M (2008) Widespread occurrence of an emerging pathogen in amphibian communities of the Venezuelan Andes. Biological Conservation 141: 2898–2905. doi: 10.1016/j.biocon.2008.08.009

Santos Barrera G, Peralta Garcia A (2018) Earliest record of *Batrachochytrium dendrobatidis* in amphibian populations of Baja California, Mexico. Herpetological Review 49(4): 693–695.

Sapsford SJ, Alford RA, Schwarzkopf L (2013) Elevation, temperature, and aquatic connectivity all influence the infection dynamics of the amphibian chytrid fungus in adult frogs. PLoS ONE 8(12): e82425. doi: 10.1371/journal.pone.0082425

Sapsford SJ, Voordouw MJ, Alford RA, Schwarzkopf L (2015) Infection dynamics in frog populations with different histories of decline caused by a deadly disease. Oecologia 179: 1099–1110. doi: 10.1007/s00442-015-3422-3

Savage AE, Sredl MJ, Zamudio KR (2011) Disease dynamics vary spatially and temporally in a North American amphibian. Biological Conservation 144: 1910–1915. doi: 10.1016/j.biocon.2011.03.018

Savage AE, Grismer LL, Anuar S, Onn CK, Grismer JL, Quah E, Muin MA, Ahmad N, Lenker M, Zamudio KR (2011) First record of *Batrachochytrium dendrobatidis* infecting four frog families from Peninsular Malaysia. EcoHealth 8: 121–128. doi: 10.1007/s10393-011-0685-y

Scalera R, Adams MJ, Galvan SK (2008) The occurrence of *Batrachochytrium dendrobatidis* in amphibian populations in Denmark. Herpetological Review 39: 199–200.

Scheele BC, Guarino F, Osborne W, Hunter DA, Skerratt LF, Driscoll DA (2014) Decline and re-expansion of an amphibian with high prevalence of chytrid fungus. Biological Conservation 170: 86–91. http://dx.doi.org/10.1016/j.biocon.2013.12.034

Scheele BC, Driscoll DA, Fischer J, Fletcher AW, Hanspach J, Vörös J, Hartel T (2015a Anim Cons) Landscape context influences chytrid fungus distribution in an endangered European amphibian. Animal Conservation 18: 480–488. doi: 10.1111/acv.12199

Scheele BC, Hunter DA, Skerratt LF, Brannelly LA, Driscoll DA (2015b Biol Cons) Low impact of chytridiomycosis on frog recruitment enables persistence in refuges despite high adult mortality. Biological Conservation 182: 36–43. http://dx.doi.org/10.1016/j.biocon.2014.11.032

Scheele BC, Hunter DA, Banks SC, Pierson JC, Skerratt LF, Webb R, Driscoll DA (2016) High adult mortality in disease-challenged frog populations increases vulnerability to drought. Journal of Animal Ecology 85: 1453–1460. doi: 10.1111/1365-2656.12569

Scheele BC, Hunter DA, Brannelly LA, Skerratt LF, Driscoll DA (2017) Reservoir-host amplification of disease impact in an endangered amphibian. Conservation Biology 31(3): 592–600. doi:10.1111/cobi.12830

Schlaepfer MA, Sredl MJ, Rosen PC, Ryan MJ (2007) High prevalence of *Batrachochytrium dendrobatidis* in wild populations of lowland leopard frogs *Rana yavapaiensis* in Arizona. EcoHealth 4: 421–427.

Schloegel LM, Picco AM, Kilpatrick AM, Davies AJ, Hyatt AD, Daszak P (2009) Magnitude of the US trade in amphibians and presence of *Batrachochytrium dendrobatidis* and ranavirus infection in imported American bullfrogs (*Rana catesbeiana*). Biological Conservation 142: 1420–1426. doi: 10.1016/j.biocon.2009.02.004

Schloegel LM, Ferreira CM, Hipolito M, Longcore JE, Hyatt AD, Yabsley M, Martins AMCRPF, Mazzoni R, Davies AJ, Daszak P (2010) The North American bullfrog as a reservoir for the spread of *Batrachochytrium dendrobatidis* in Brazil. Animal Conservation 13(Suppl. 1): 53–61. doi: 10.1111/j.1469-1795.2009.00307.x

Schloegel LM, Toledo LF, Longcore JE, Greenspan SE, Vieira CA, Lee M, Zhao S, Wangen C, Ferreira CM, Hipolito M, Davies AJ, Cuomo CA, Daszak P, James TY (2012) Novel, panzootic and hybrid genotypes of amphibian chytridiomycosis associated with the bullfrog trade. Molecular Ecology 21: 5162–5177. doi: 10.1111/j.1365-294X.2012.05710.x

Schock DM, Ruthig GR, Collins JP, Kutz SJ, Carrière S, et al. (2010) Amphibian chytrid fungus and ranaviruses in the Northwest Territories, Canada. Diseases of Aquatic Organisms 92: 231–240. doi: 10.3354/dao02134.

Schrenker EA (2017) Measuring the presence of the amphibian pathogen *Batrachochytrium dendrobatidis* in East Tennessee. Honors Thesis, University of Tennessee at Chattanooga. https://scholar.utc.edu/honors-theses/95/

Seeley KE, D’Angelo MD, Gowins C, Greathouse J (2016) Prevalence of *Batrachochytrium dendrobatidis* in Eastern Hellbenders (*Cryptobranchus alleganiensis*) populations in West Virginia, USA. Journal of Wildlife Diseases 52(2): 391–394. doi: 10.7589/2015-02-152

Seimon TA, Seimon A, Daszak P, Halloy SRP, Schloegel LM, et al. (2007) Upward range extension of Andean anurans and chytridiomycosis to extreme elevations in response to tropical deglaciation. Global Change Biology 13: 288–299. doi: 10.1111/j.1365-2486.2006.01278.x

Seimon TA, Ayebare S, Sekisambu R, Muhindo E, Mitamba G, Greenbaum E, Menegon M, Pupin F, McAloose D, Ammazzalorso A, Meirte D, Lukwago W, Behangana M, Seimon A, Plumptre AJ (2015) Assessing the threat of amphibian chytrid fungus in the Albertine Rift: past, present and future. PLoS ONE 10(12): e0145841. doi: 10.1371/journal.pone.0145841

Seimon TA, Seimon A, Yager K, Reider K, Delgado A, Sowell P, Tupayachi A, Konecky B, McAloose D, Halloy S (2017) Long-term monitoring of tropical alpine habitat change, Andean anurans, and chytrid fungus in the Cordillera Vilcanota, Peru: results from a decade of study. Ecology and Evolution 7: 1527–1540. doi: 10.1002/ece3.2779

Sette CM, Vredenburg VT, Zink AG (2015) Reconstructing historical and contemporary disease dynamics: a case study using the California slender salamander. Biological Conservation 192: 20–29. http://dx.doi.org/10.1016/j.biocon.2015.08.039

Shaw SD, Skerrat LF, Haigh A, Bell BD, Daglish L, Bishop PJ, Summers R, Moreno V, Melzer S, Ohmer M, Herbert S, Gleeson D, Rowe L, Speare R (2013) The distribution and host range of *Batrachochytrium dendrobatidis* in New Zealand, 1930–2010. Ecology 94(9): 2108. Ecological Archives E094-192.

Shin J, Bataille A, Kosch TA, Waldman B (2014) Swabbing often fails to detect amphibian chytridiomycosis under conditions of low infection load. PLoS ONE 9(10): e111091. doi:10.1371/journal.pone.0111091

Sigafus BH, Schwalbe CR, Hossack BR, Muths E (2014) Prevalence of the amphibian chytrid fungus (*Batrachochytrium dendrobatidis*) at Buenos Aires National Wildlife Refuge, Arizona, USA. Herpetological Review 45(1): 41–42.

Simoncelli F, Fagotti A, Dall’Olio R, Vagnetti D, Pascolini R, et al. (2005) Evidence of *Batrachochytrium dendrobatidis* infection in water frogs of the *Rana esculenta* complex in central Italy. EcoHealth 2: 307–312.

Simpkins C, Hero J-M, Van Sluys M (2010) Detecting the western limits for *Batrachochytrium dendrobatidis* in southeastern Queensland, Australia. Herpetological Review 41: 454–456.

Simpkins CA, Kriger K, Hero J-M (2017) Prevalence of *Batrachochytrium dendrobatidis* on amphibians from low pH, oligotrophic waterbodies. Herpetological Review 48(4): 775–776.

Skerratt LF, Berger L, Hines HB, McDonald KR, Mendez D, Speare R (2008) Survey protocol for detecting chytridiomycosis in all Australian frog populations. Diseases of Aquatic Organisms 80(2): 85–94. doi: 10.3354/dao01923

Skerratt LF, McDonald KR, Hines HB, Berger L, Mendez D, Phillott AD, Cashins SD, Murray KA, Speare R (2010) Application of the survey protocol for chytridiomycosis to Queensland, Australia. Diseases of Aquatic Organisms 92: 117–129. doi: 10.3354/dao02272

Slough BG (2009) Amphibian chytrid fungus in Western Toads (*Anaxyrus boreas*) in British Columbia and Yukon, Canada. Herpetological Review 40: 319–321.

Smith KG, Weldon C, Conradie W, du Preez LH (2007) Relationships among size, development, and *Batrachochytrium dendrobatidis* infection in African tadpoles. Diseases of Aquatic Organisms 74: 159–164.

Smith SN, Watters JL, Ellsworth ED, Davis DR, Siler CD (2019) Assessment of *Batrachochytrium dendrobatidis* and ranavirus among wild amphibians from four Philippine islands. Herpetological Review 50(4):

Smith TC, Picco AM, Knapp R (2017) Ranaviruses infect mountain yellow-legged frogs (*Rana muscosa* and *Rana sierrae*) threatened by *Batrachochytrium dendrobatidis*. Herpetological Conservation and Biology 12:149–159.

Solís R, Lobos G, Walker SF, Fisher M, Bosch J (2010) Presence of *Batrachochytrium dendrobatidis* in feral populations of *Xenopus laevis* in Chile. Biological Invasions 12: 1641–1646. Doi: 10.1007/s10530-009-9577-2 [Solis]

Solís R, Penna M, De la Riva I, Fisher MC, Bosch J (2015) Presence of *Batrachochytrium dendrobatidis* in anurans from the Andes highlands of northern Chile. Herpetological Journal 24: 55–59. [Solis]

Sonn JM, Utz RM, Richards-Zawacki CL (2019) Effects of latitudinal, seasonal, and daily temperature variations on chytrid fungal infections in a North American frog. Ecosphere 10(11): article e02892. doi: 10.1002/ecs2.2892 ; data in Dryad: https://doi.org/10.5061/dryad.7h44j0zqd

Soorae PT, Al Abdessalaam T, Tourenq C, Shuriqi MK, Al Mehairbi M (2012) Preliminary analyses suggest absence of the amphibian chytrid fungus in native and exotic amphibians of the United Arab Emirates. Salamandra 48(3): 173-176.

Soto-Azat C, Cunningham AA (2010) An invasive frog and endemic threatened amphibians: Impacts of chytridiomycosis in Chile. Conference abstract. EcoHealth 2010, Global Ecohealth Challenges; Multiple Perspectives, 18–20 August 2010, London, UK. pp. 75–76. Abstracts available online at: https://profileproductions.eventtrac.co.uk/system/attachments/168/original/EcoHealth_Final_online_programme_abstractbook.pdf

Soto-Azat C, Clarke BT, Poynton JC, Cunningham AA (2009) Widespread historical presence of *Batrachochytrium dendrobatidis* in African pipid frogs. Diversity Distrib 2009: 1–6. doi: 10.1111/j.1472-4642.2009.00618.x

Soto-Azat C, Valenzuela-Sánchez A, Clarke BT, Busse K, Ortiz JC, Barrientos C, Cunningham AA (2013) Is chytridiomycosis driving Darwin’s Frogs to extinction? PLoS ONE 8(11): e79862. doi: 10.1371/journal.pone.0079862

Soto-Azat C, Peñafiel-Ricaurte A, Price SJ, Sallaberry-Pincheira N, García MP, Alvarado-Rybak M, Cunningham AA (2016) *Xenopus laevis* and emerging pathogens in Chile. EcoHealth 13: 775–785. doi: 10.1007/s10393-016-1186-9.

Souza MJ, Gray MJ, Colclough P, Miller DL (2012) Prevalence of infection by *Batrachochytrium dendrobatidis* and *Ranavirus* in Eastern Hellbenders (*Cryptobranchus alleganiensis alleganiensis*) in eastern Tennessee. Journal of Wildlife Diseases 48(3): 560–566.

Spaulding SH, Cox JJ, Maigret TA, Drayer AN, Richards JM, Treanor J (2018) Low-level *Batrachochytrium dendrobatidis* detection persists in plethodontid salamanders following timber harvest in Kentucky, USA. Herpetological Review 49(2): 258–262.

Speare R, Berger L (2005) Chytridiomycosis in amphibians in Australia. Available at: http://www.jcu.edu.au/school/phtm/PHTM/frogs/chyspec.htm 26 January 2005. (2005).

Spitzen-van der Sluijs A, Martel A, Wombwell E, Van Rooij P, Zollinger R, Woeltjes T, Rendle M, Haesebrouck F, Pasmans F (2011) Clinically healthy amphibians in captive collections and at pet fairs: a reservoir of *Batrachochytrium dendrobatidis*. Amphibia-Reptilia 32: 419–423. doi: 10.1163/017353711X57983.

Spitzen-van der Sluijs A, Martel A, Hallmann CA, Bosman W, Garner TWJ, van Rooij P, Jooris R, Haesebrouck F, Pasmans F (2014) Environmental determinants of recent endemism of *Batrachochytrium dendrobatidis* infections in amphibian assemblages in the absence of disease outbreaks. Conservation Biology 28(5): 1302–1311. doi: 10.1111/cobi.12281

Sredl M, Caldwell D (2000) Wintertime population surveys - Call for volunteers. Sonoran Herpetologist (Tucson Herpetological Newsletter) 13: 1.

Sredl MJ et al. (2002) Nongame and Endangered Wildlife Program Technical Report 208. Arizona Game and Fish Department, Phoenix, Arizona.

Stagni G, Scoccianti C, Fusini R (2002) Segnalazione di chytridiomicosi in popolazioni di *Bombina pachypus* (Anura, Bombinatoridae) dell'Appennino Tosco-emiliano. IV Congresso della Societas Herpetologica Italica, a Ercolano.

Stagni G, Dall’Olio R, Fusini U, Mazzotti S, Scoccianti C, Serra A (2004) Declining populations of Apennine yellow-bellied toad *Bombina pachypus* in the northern Apennines (Italy): is *Batrachochytrium dendrobatidis* the main cause? Italian Journal of Zoology 2004(Suppl. 2): 151–154.

St-Amour V, Wong WM, Garner TWJ, Lesbarrères D (2008) Anthropogenic influence on the prevalence of two amphibian pathogens. Emerging Infectious Diseases 14: 1175–1176. [no locations or species given, but may represent some of the unpublished data in the database from this author]

St-Amour V, Garner TWJ, Schulte-Hostedde AI, Lesbarrères D (2010) Effects of two amphibian pathogens on the developmental stability of Green Frogs. Conservation Biology 24(3): 788–794. doi: 10.1111/j.1523-1739.2009.01400.x

Stark T, Laurijssens C, Weterings M, Martel A, Köhler G, Pasmans F (2017) Prevalence of *Batrachochytrium dendrobatidis* in a Nicaraguan, micro-endemic Neotropical salamander, *Bolitoglossa mombachoensis*. Amphibia-Reptilia 38: 102–107. doi: 10.1163/15685381-00003077.

Steiner SL, Lehtinen RM (2008) Occurrence of the amphibian pathogen *Batrachochytrium dendrobatidis* in Blanchard’s Cricket Frog (*Acris crepitans blanchardi*) in the U.S. Midwest. Herpetological Review 39: 193–196.

Stevens SD, Prescott DRC, Whiteside DP (2012) Occurrence and prevalence of chytrid fungus (*Batrachochytrium dendrobatidis*) in amphibian species of Alberta. Alberta Species at Risk Report No. 143. March 2012. Edmonton, Alberta, Canada: Alberta Environment / Alberta Sustainable Resource Development. https://open.alberta.ca/publications/9781460100318

Stockwell MP, Bower DS, Bainbridge L, Clulow J, Mahony MJ (2015) Island provides a pathogen refuge within climatically suitable area. Biodiversity and Conservation 24: 2583–2592. doi: 10.1007/s10531-015-0946-0

Stockwell MP, Bower DS, Clulow J, Mahony MJ (2016) The role of non-declining amphibian species as alternative hosts for *Batrachochytrium dendrobatidis* in an amphibian community. Wildlife Research 43: 341–347. http://dx.doi.org/10.1071/WR15223

Stutz WE, Blaustein AR, Briggs CJ, Hoverman JT, Rohr JR, Johnson PTJ (2018) Using multi-response models to investigate pathogen coinfections across scales: Insights from emerging pathogens of amphibians. Methods in Ecology and Evolution 9: 1109–1120. doi: 10.1111/2041-210X.12938

Šunje E, Pasmans F, Maksimović Z, Martel A, Rifatbefović M (2018) Recorded mortality in the vulnerable Alpine salamander, *Salamandra atra prenjensis* (Amphibia: Caudata), is not associated with the presence of known amphibian pathogens. Salamandra 54(1): 75–79.

Sura P, Janulis E, Profus P (2010) Chytridiomikoza – śmiertelne zagrożenie dla płazów. [Chytridiomycosis – a mortal danger for amphibians.] Chrońmy Przyrodę Ojczystą 66(6): 406–421. [in Polish]

Suriyamongkol T, Villamizar-Gomez A, Forstner MRJ, Mali I (2019) Detection of *Batrachochytrium dendrobatidis* in eastern New Mexico, USA. Herpetological Review 50(2): 300–303.

Sweeney R (2016) First detection of the amphibian chytrid fungus (*Batrachochytrium dendrobatidis*) in St. Vincent and the Grenadines. Herpetological Review 47(2): 212–214.

Swei, A, Rowley JJL, Rödder D, Diesmos MLL, Diesmos AC, Briggs CJ, Brown R, Cao TT, Cheng TL, Chong RA, Han B, Hero J-M, Hoang HD, Kusrini MD, Le DTT, McGuire JA, Meegaskumbura M, Min M-S, Mulcahy DG, Neang T, Phimmachak S, Rao D-Q, Reeder NM, Schoville SD, Sivongxay N, Srei N, Stöck M, Stuart BL, Torres LS, Tran DTA, Tunstall TS, Vieites D, Vredenburg VT (2011) Is chytridiomycosis an emerging infectious disease in Asia? PLoS ONE 6(8): e23179. doi: 10.1371/journal.pone.0023179

Symonds EP, Hines HB, Bird PS, Morton JM, Mills PC (2007) Surveillance for *Batrachochytrium dendrobatidis* using *Mixophyes* (Anura: Myobatrachidae) larvae. Journal of Wildlife Diseases 43: 48–60.

Sztatecsny M, Glaser F (2011) From the eastern lowlands to the western mountains: first records of the chytrid fungus *Batrachochytrium dendrobatidis* in wild amphibian populations from Austria. Herpetological Journal 21: 87–90.

Sztatecsny M, Hödl W (2009) Can protected mountain areas serve as refuges for declining amphibians? Potential threats of climate change and amphibian chytridiomycosis in an alpine amphibian population. Eco.mont 1(2): 19–24.

Talbott K, Wolf TM, Sebastian P, Abraham M, Bueno I, McLaughlin M, Harris T, Thompson R, Pessier AP, Travis D (2018) Factors influencing detection and co-detection of *Ranavirus* and *Batrachochytrium dendrobatidis* in Midwestern North American anuran populations. Diseases of Aquatic Organisms 128: 93–103. http://doi.org/10.3354/dao03217

Talley BL, Lips KR, Ballard SR (2011) *Batrachochytrium dendrobatidis* in *Siren intermedia* in Illinois, USA. Herpetological Review 42(2): 216–217.

Talley BL, Muletz CR, Vredenburg VT, Fleischer RC, Lips KR (2015) A century of *Batrachochytrium dendrobatidis* in Illinois amphibians (1888–1989). Biological Conservation 182: 254–261. http://dx.doi.org/10.1016/j.biocon.2014.12.007

Tamukai K, Une Y, Tominaga A, Suzuki K, Goka K (2014) *Batrachochytrium dendrobatidis* prevalence and haplotypes in domestic and imported pet amphibians in Japan. Diseases of Aquatic Organisms 109: 165–175. doi: 10.3354/dao02732

Tarrant J, Cilliers D, du Preez LH, Weldon C (2013) Spatial assessment of amphibian chytrid fungus (*Batrachochytrium dendrobatidis*) in South Africa confirms endemic and widespread infection. PLoS ONE 8(7): e69591. doi: 10.1371/journal.pone.0069591

Tarvin RD, Peña P, Ron SR (2014) Changes in population size and survival in *Atelopus spumarius* (Anura: Bufonidae) are not correlated with chytrid presence. Journal of Herpetology 48(3):291–297. doi: 10.1670/11-269

Tatarian P, Tatarian G (2010) Chytrid infection of *Rana draytonii* in the Sierra Nevada, California. Herpetological Review 41: 325–327.

Techangamsuwan S, Sommanustweechai A, Kamolnorranart S, Siriaroonrat B, Khonsue W, Pirarat N (2017) Emerging chytrid fungal pathogen *Batrachochytrium dendrobatidis*, in zoo amphibians in Thailand. Acta Veterinaria-Beograd 67(4): 525–529. doi: 10.1515/acve-2017-0042

Terrell VCK, Engbrecht NJ, Pessier AP, Lannoo MJ (2014) Drought reduces chytrid fungus (*Batrachochytrium dendrobatidis*) infection intensity and mortality but not prevalence in adult Crawfish Frogs (*Lithobates areolatus*). Journal of Wildlife Diseases 50(1): 56–62. doi: 10.7589/2013-01-016

Thien TN, Martel A, Brutyn M, Bogaerts S, Sparreboom M, Haesebrouck F, Fisher MC, Beukma W, Van TD, Chiers K, Pasmans F (2013) A survey for *Batrachochytrium dendrobatidis* in endangered and highly susceptible Vietnamese salamanders (*Tylototriton* spp.). Journal of Zoo and Wildlife Medicine 44(3): 627–633.

Thompson LM, Pugh B, McDonald LA, Estrada A, Horn K, Gilman BLC, Belden LK, Mitchell JC, Grayson KL (2019) Surveys for population persistence and *Bd* at the northeastern range edge of the Eastern Lesser Siren. Northeaster Naturalist 26(2): 410–419. URL: https://doi.org/10.1656/045.026.0216

Thompson PD, Fridell RA, Wheeler KK, Bailey CL (2004) Distribution of *Bufo boreas* in Utah. Herpetological Review 35: 255–257.

Timpe EK, Graham SP, Gagliardo RW, Hill RL, Levy MG (2008) Occurrence of the fungal pathogen *Batrachochytrium dendrobatidis* in Georgia’s amphibian populations. Herpetological Review 39: 447–449.

Tinsley RC, Coxhead PG, Stott LC, Tinsley MC, Piccinni MZ, Guille MJ (2015) Chytrid fungus infections in laboratory and introduced *Xenopus laevis* populations: assessing the risks for U.K. native amphibians. Biological Conservation 184: 380–388. http://dx.doi.org/10.1016/j.biocon.2015.01.034

Tobler U, Schmidt BR (2010) Within- and among-population variation in chytridiomycosis-induced mortality in the toad *Alytes obstetricans*. PLoS ONE 5(6): e10927. doi:10.1371/journal.pone.0010927

Tobler U, Borgula A, Schmidt BR (2012) Populations of a susceptible amphibian species can grow despite the presence of a pathogenic chytrid fungus. PLoS ONE 7(4): e34667. doi:10.1371/journal.pone.0034667

Todd H, Fritzler JM, Kazmaier RT, Johnson JB (2019) Prevalence of *Batrachochytrium dendrobatidis* and ranavirus in western Texas, USA. Herpetological Review 50(3): 505–507.

Todd-Thompson M, Miller DL, Super PE, Gray MJ (2009) Chytridiomycosis-associated mortality in a *Rana palustris* collected in Great Smoky Mountains National Park, Tennessee, USA. Herpetological Review 40: 321–323.

Toledo LF, Britto FB, Araujo OGS, Giasson LMO, Haddad CFG (2006a) The occurrence of *Batrachochytrium dendrobatidis* in Brazil and the inclusion of 17 new cases of infection. South American Journal of Herpetology 1: 185–191.

Toledo LF, Haddad CFB, Carnaval ACOQ, Britto FB (2006b) A Brazilian anuran (*Hylodes magalhaesi*: Leptodactylidae) infected by *Batrachocytrium dendrobatidis*: a conservation concern. Amphibian and Reptile Conservation 4: 17–21.

Tominaga A, Irwin KJ, Freake MJ, Suzuki K, Goka K (2013) *Batrachochytrium dendrobatidis* haplotypes on the hellbender *Cryptobranchus alleganiensis* are identical to global strains. Diseases of Aquatic Organisms 102: 181–186. doi: 10.3354/dao02561

Tupper TA, Streicher JW, Greenspan SE, Timm BC, Cook RP (2011) Detection of *Batrachochytrium dendrobatidis* in anurans of Cape Cod National Seashore, Barnstable County, Massachusetts, USA. Herpetological Review 42(1): 62–65.

Tupper TA, Bozarth CA, Jones KS, Cook RP (2014) Detection of *Batrachochytrium dendrobatidis* in the Eastern Spadefoot, *Scaphiopus holbrookii*, at Cape Cod National Seashore, Barnstable County, Massachusetts, USA. Herpetological Review 45(3): 445–447.

Tupper TA, Fuchs LD, O’Connor-Love C, Aguilar R, Bozarth C, Fernandez D (2017) Detection of the pathogenic fungus, *Batrachochytrium dendrobatidis*, in anurans of Huntley Meadows Park, Fairfax County, Virginia. Catesbeiana (Virginia Herpetological Society) 37(2): 109–120.

Une Y, Kadekaru S, Tamukai K, Goka K, Kuroki T (2008) First reports of spontaneous chytridiomycosis in frogs in Asia. Diseases of Aquatic Organisms 82: 157–160.

Urbina J, Bredeweg EM, Garcia TS, Blaustein AR (2018) Host-pathogen dynamics among the invasive American bullfrog (*Lithobates catesbeianus*) and chytrid fungus (*Batrachochytrium dendrobatidis*). Hydrobiologia 817: 267–277. https://doi.org/10.1007/s10750-018-3614-z

Urbina JC, Galeano SP (2011) *Batrachochytrium dendrobatidis* detected in amphibians of the central Andean Cordillera of Colombia. Herpetological Review 42(4): 558–560.

Valencia-Aguilar A, Ruano-Fajardo G, Lambertini C, da Silva Leite D, Toledo LF, Mott T (2015) Chytrid fungus acts as a generalist pathogen infecting species-rich amphibian families in Brazilian rainforests. Diseases of Aquatic Organisms 114: 61–67. doi: 10.3354/dao02845

Valencia-Aguilar A, Toledo LF, Vital MVC, Mott T (2016) Seasonality, environmental factors, and host behavior linked to disease risk in stream-dwelling tadpoles. Herpetologia 72(2): 98–106.

Valenzuela-Sánchez A, Schmidt BR, Uribe-Rivera DE, Costas F, Cunningham AA, Soto-Azat C (2017) Cryptic disease-induced mortality may cause host extinction in an apparently stable host-parasite system. Proceedings of the Royal Society B 284: 20171176. http://dx.doi.org/10.1098/rspb.2017.1176

van der Hoek Y, Emmanuel F, Twahirwa JC, Tuyisenge MF, Tuyisenge D (2019) Amphibian chytrid fungus *Batrachochytrium dendrobatidis* detected at high elevations in Volcanoes National Park, Rwanda. Herpetological Review 50(4): 727–729.

Van Rooij P, Martel A, Nerz J, Voitel S, Van Immerseel F, Haesebrouck F, Pasmans F (2011) Detection of *Batrachochytrium dendrobatidis* in Mexican bolitoglossine salamanders using an optimal sampling protocol. EcoHealth 8: 237–243. doi: 10.1007/s10393-001-0704-z

Van Sluys M, Hero J-M (2009) How does chytrid infection vary among habitats? The case of *Litoria wilcoxii* (Anura, Hylidae) in SE Queensland, Australia. EcoHealth 6: 579-583. doi: 10.1007/s10393-010-0278-1

Van Sluys M, Ferreira TK, Lamarão FRM, Moraes MO (2007) *Batrachochytrium dendrobatidis* infects *Melanophryniscus moreirae* (Anura Bufonidae) from an Atlantic Rainforest area (Itatiaia), southeastern Brazil. FrogLog 84: 6–8. http://www.amphibians.org/wp-content/uploads/2011/08/FrogLog84.pdf

Vásquez-Ochoa A, Carmona PB, Salcedo LDP, Franco-Correa M (2012) Detección y cuantificación de *Batrachochytrium dendrobatidis* en anfibios de las regions Andina Central, Oriental, Orinoquia y Amazonia de Colombia. Herpetotropicos 8(1-2): 13–21.

Velásquez-E BE, Castro F, Bolívar-G W, Herrera MI (2008) Infección por el hongo quitrido *Batrachochytrium dendrobatidis* en anuros de la Cordillera Occidental de Colombia [Infection by the chytrid fungus *Batrachochytrium dendrobatidis* in anurans of the Cordillera Occidental of Colombia] Herpetotropicos 4: 65–70 [in Spanish].

Velo-Antón G, Rodríguez D, Savage AE, Parra-Olea G, Lips KR, Zamudio KR (2012) Amphibian-killing fungus loses genetic diversity as it spreads across the New World. Biological Conservation 146: 213–218. DOI: 10.1016/j.biocon.2011.12.003

Venegas PJ, Catenazzi A, Siu-Ting K, Carillo J. (2008) Two new harlequin frogs (Anura: *Atelopus*) from the Andes of northern Peru. Salamandra 44: 163–176.

Venesky MD, Brem FM (2008) Occurrence of *Batrachochytrium dendrobatidis* in southwestern Tennesee, USA. Herpetological Review 39: 319–320.

Vieira CA, Almeida CHLN, Lambertini C, Leite DDS, Toledo LF (2012) First record of *Batrachochytrium dendrobatidis* in Paraná, Brazil. Herpetological Review 431: 93–94.

Vieira CA, Toledo LF, Longcore JE, Longcore JR (2013) Body length of *Hylodes* cf. *ornatus* and *Lithobates catesbeianus* tadpoles, depigmentation of mouthparts, and presence of *Batrachochytrium dentrobatidis* are related. Brazilian Journal of Biology 73(1): 195–199.

Viertel B, Veith M, Schick S, Channing A, Kigoolo S, Baeza-Urrea O, Sinsch U, Lötters S. (2012) The stream-dwelling larva of the Ruwenzori River Frog, *Amietia ruwenzorica*, its buccal cavity and pathology of chytridiomycosis. Zootaxa 3400: 43–57.

Villamizar-Gómez A, Farr W, Hahn D, Dixon JR, Lazcano D, Forstner MRJ (2015) Absence of *Batrachochtyrium dendrobatidis* in eighteen species of amphbians from a variety of habitats in Tamaulipas, Mexico. Herpetological Review 46(1): 34–37.

Villamizar-Gómez A, Forstner MRJ, Suriyamongkol T, Forks KN, Grant WE, Wang H-H, Mali I (2016) Prevalence of *Batrachochytrium dendrobatidis* in two sympatric treefrog species, *Hyla cinerea* and *Hyla versicolor*. Herpetological Review 47(4): 601–605.

Vojar J, Havlíková B, Solský M, Jablonski D, Iković V, Baláž V (2017) Distribution, prevalence and amphibian hosts of *Batrachochytrium dendrobatidis* in the Balkans. Salamandra 53(1): 44–49. [Havlikova, Solsky, Ikovic, Balaz]

Voordouw MJ, Adama D, Houston B, Govindarajulu P, Robinson J (2010) Prevalence of the pathogenic chytrid fungus, *Batrachochytrium dendrobatidis*, in an endangered population of Northern Leopard Frog, *Rana pipiens*. BMC Ecology 10: 6. http://www.biomedcentral.com/1472-6785/10/6

Vörös J, Jelić D (2011) First steps to survey chytrid fungus in Croatia [Prvi koraci u istraživanju chytrid gljivica u Hrvatskoj]. Hyla 2011(1): 31–34. [Voros, Jelic]

Vörös J, Price L, Donnellan SC (2011) *Batrachochytrium dendrobatidis* on the endemic frog *Litoria raniformis* in South Australia. Herpetological Review 422: 220–223. [Voros]

Vörös J, Satasook C, Bates P, Wangkulangkul S (2012) First record of the amphibian chytrid fungus, *Batrachochytrium dendrobatidis* in Thailand. Herpetology Notes 5: 519–521. [Voros]

Vörös J, Bosch J, Dan A, Hartel T (2013) First record of *Batrachochytrium dendrobatidis* on amphibians in Romania. North-western Journal of Zoology 9(2): art. 132510. [Voros]

Vörös J, Herczeg D, Fülöp A, Gál TJ, Dán A, Harmos K, Bosch J (2018) *Batrachochytrium dendrobatidis* in Hungary: an overview of recent and historical occurrence. Acta Herpetologica 13(2): 125–140. doi: 10.13128/Acta_Herpetol-22611 [Voros, Fulop, Gal, Dan]

Vredenburg VT, Knapp RA, Tunstall TS, Briggs CJ (2010) Dynamics of an emerging disease drive large-scale amphibian population extinctions. Proceedings of the National Academy of Sciences USA 107(21): 9689–9694. https://doi.org/10.1073/pnas.0914111107

Vredenburg VT, du Preez L, Raharivololoniaina L, Vieites DR, Vences M, Weldon C (2012) A molecular survey across Madagascar does not yield positive records of the amphibian chytrid fungus *Batrachochytrium dendrobatidis*. Herpetology Notes 5: 507–517.

Vredenburg VT, Felt SA, Morgan EA, McNally SVG, Wilson S, Green SL (2013) Prevalence of *Batrachochytrium dendrobatidis* in *Xenopus* collected in Africa (1871–2000) and in California (2001–2010). PLoS ONE 8(5): e63791. doi: 10.1371/journal.pone.0063791

Waddle AW, Levy JE, Rivera R, van Breukelen F, Nash M, Jaeger JR (2019) Population-level resistance to chytridiomycosis is life-stage dependent in an imperiled amphibian. EcoHealth 16: 701–711. https://doi.org/10.1007/s10393-019-01446-y

Wagner N, Neubeck C, Guicking D, Finke L, Wittich M, Weising K, Geske C, Veith M (2017) No evidence for effects of infection with the amphibian chytrid fungus on populations of yellow-bellied toads. Diseases of Aquatic Organisms 123: 55–65. doi: 10.3354/dao03090

Waldman B, van de Wolfshaar K, Andjic V, Klena J, Bishop P et al. (2001) Chytridiomycosis and frog mortality in New Zealand. New Zealand Journal of Zoology 28: 372.

Walker S, Bosch J, James TY, Litvintseva AP, Valls JAO et al. (2008) Invasive pathogens threaten species recovery programs. Current Biology 18: R853–R854.

Warne RW, LaBumbard B, LaGrange S, Vredenburg VT, Catenazzi A (2016) Co-infection by chytrid fungus and *Ranaviruses* in wild and harvested frogs in the tropical Andes. PLoS ONE 11(1): e0145864. doi: 10.1371/journal.pone.0145864

Watters JL, Flanagan RL, Davis DR, Farkas JK, Kerby JL, Labonte MJ, Penrod ML, Siler CD (2016) Screening natural history collections for historical presence of *Batrachochytrium dendrobatidis* in anurans from Oklahoma, USA. Herpetological Review 47(2): 214–220.

Watters JL, Davis DR, Yuri T, Siler CD (2018) Concurrent infection of *Batrachochytrium dendrobatidis* and Ranavirus among native amphibians from northeastern Oklahoma, USA. Journal of Aquatic Animal Health 30: 291–301. doi: 10.1002/aah.10041

Watters JL, McMillin SL, Marhanka EC, Davis DR, Farkas JK, Kerby JL, Siler CD (2019) Seasonality in *Batrachochytrium dendrobatidis* detection in amphibians in central Oklahoma, USA. Journal of Zoo and Wildlife Medicine 50(2): 492–497.

Wei Y, Xu K, Zhu D-Z, Chen X-F, Wang X-L. 2010. Early-spring survey for *Batrachochytrium dendrobatidis* in wild *Rana dybowskii* in Heilongjiang Province, China. Diseases of Aquatic Organisms 92: 241–244. doi: 10.3354/dao02172

Weinstein SB (2009) An aquatic disease on a terrestrial salamander: individual and population level effects of the amphibian chytrid fungus, *Batrachochytrium dendrobatidis*, on *Batrachoseps attenuatus* (Plethodontidae). Copeia 4: 653–660.

Weldon C (2005) Chytridiomycosis an emerging infectious disease of amphibians in South Africa. PhD Dissertation. North-West University, Potchefstroom. Available: http://www.puk.ac.za/opencms/export/PUK/html/fakulteite/natuur/soo/drk/aacrg/chytrid/Chytrid-host_species_of_chytridiomycosis_in_Africa.doc. Accessed on August 6, 2007.

Weldon C, Du Preez LH (2004) Decline of the Kihansi Spray Toad, *Nectophrynoides asperginus*, from the Udzungwa Mountains, Tanzania. Froglog 62: 2–3.

Weldon C, Du Preez L, Vences M (2008) Lack of detection of the amphibian chytrid fungus (*Batrachochytrium dendrobatidis*) in Madagascar. In: Andreone F, ed. A conservation strategy for the amphibians of Madagascar. Monografie del Museo Regionale di Scienze Naturali di Torino, XLV (2008): 95–106.

Weldon C, Dalton DL, Kotze A (2014) Amphibian survey and current absence of *Batrachochytrium dendrobatidis* (*Bd*) in Ivoloina Park, Toamasina (eastern Madagascar). African Journal of Herpetology 63(1): 70–78.

Weldon C, Channing A, Misinzo G, Cunningham AA (2020) Disease driven extinction in the wild of the Kihansi spray toad, *Nectophrynoides asperginis*. African Journal of Herpetology, DOI: 10.1080/21564574.2020.1752313

Whitfield SM, Kerby J, Gentry LR, Donnelly MA (2012) Temporal variation in infection prevalence by the amphibian chytrid fungus in three species of frogs at La Selva, Costa Rica. Biotropica 44(6): 779–784. doi: 10.1111/j.1744-7429.2012.00872.x

Whitfield SM, Geerdes E, Chacon I, Ballestero Rodriguez E, Jimenez RR, Donnelly MA, Kerby JL (2013) Infection and co-infection by the amphibian chytrid fungus and ranavirus in wild Costa Rican frogs. Diseases of Aquatic Organisms 104: 173–178. doi: 10.3354/02598

Whitfield SM, Alvarado G, Abarca J, Zumbado H, Zuñiga I, Wainwright M, Kerby J (2017) Differential patterns of *Batrachochytrium dendrobatidis* infection in relict amphibian populations following sever disease-associated declines. Diseases of Aquatic Organisms 126: 33–41. https://doi.org/10.3354/dao03154

Williams LA, Groves JD (2014) Prevalence of the amphibian pathogen *Batrachochytrium dendrobatidis* in Eastern Hellbenders (*Cryptobranchus alleganiensis*) in western North Carolina, USA. Herpetological Conservation and Biology 9(3): 454–467.

Wilson EA, Briggs CJ, Dudley TL (2018) Invasive African clawed frogs in California: A reservoir for or predator against the chytrid fungus? PLoS ONE 13(2): e0191537. doi: https://doi.org/10.1371/journal.pone.0191537

Wilson TP, Barbosa JM, Carver EA, Reynolds BR, Richards D, Team Salamander, Wilson TM (2015) *Batrachochytrium dendrobatidis* prevalence in two ranid frogs on a former United States Department of Defense installation in southeastern Tennessee. Herpetological Review 46(1): 34–41.

Wimsatt J, Feldman SH, Heffron M, Hammond M, Roth Ruehling MP, Grayson KL, Mitchell JC (2014) Detection of pathogenic *Batrachochytrium dendrobatidis* using water filtration, animal and bait testing. Zoo Biology 33:577–585. DOI: 10.1002/zoo.21154

Windstam ST, Olori JC (2014) Proportion of hosts carrying *Batrachochytrium dendrobatidis*, causal agent of amphibian chytridiomycosis, in Oswego County, NY in 2012. Northeastern Naturalist 21(1): 25–34.

Wixson JG, Rogers KB. 2009. Detecting *Batrachochytrium dendrobatidis* in the wild when amphibians are absent. Herpetological Review 40(3): 313–316.

Wolff BG, Conway SM, Dabney CJ (2012) *Batrachochytrium dendrobatidis* and ranavirus in anurans inhabiting decorative Koi ponds near Minneapolis, Minnesota, USA. Herpetological Review 43(3): 427–429.

Wolff BG, Wurm E, Conway S, Kinzer K (2014) *Batrachochytrium dendrobatidis* infection rates differ over short distances between natural lakes and artificial ponds in Minnesota, USA. Herpetological Review 45(3): 447–449.

Wombwell EL, Garner TWJ, Cunningham AA, Quest R, Pritchard S, Rowcliffe JM, Griffiths RA (2016) Detection of *Batrachochytrium dendrobatidis* in amphibians imported into the UK for the pet trade. EcoHealth 13: 456–466. doi: 10.1007/s10393-016-1138-4

Wood LR, Griffiths RA, Schley L (2009) Amphibian chytridiomycosis in Luxembourg. Bulletin de la Societe des Naturalistes Luxembourgeois 110: 109–114.

Woodhams DC, Vredenburg VT, Simon M-A, Billheimer D, Shakhtour B et al. (2007) Symbiotic bacteria contribute to innate immune defenses of the threatened mountain yellow-legged frog, *Rana muscosa*. Biological Conservation 138: 390–398.

Woodhams DC, Kilburn VL, Reinert LK, Voyles J, Medina D et al. (2008) Chytridiomycosis and amphibian population declines continue to spread eastward in Panama. EcoHealth 5: 268–274 doi: 10.1007/s10393-008-0190-0.

Woodhams DC, Kenyon N, Bell SC, Alford RA, Chen S, Billheimer D, Shyr Y, Rollins-Smith LA (2010) Adaptations of skin peptide defences and possible response to the amphibian chytrid fungus in populations of Australian green-eyed treefrogs, *Litoria genimaculata*. Diversity and Distributions 16: 703–712. doi: 10.1111/j.1472-4642.2010.00666.x

Wunder JL, Lampazzi NM, Acre KD, Bent NJ, Canter SA, Chapman AM, Davies MA, Kashan D, Keiley JW, Macintyre RI, Milton TF, Weichler KL, Wilson MJ, Takahashi MK (2012) Promoting amphibian conservation through the college classroom: detection of *Batrachochytrium dendrobatidis* among local amphibians. Herpetological Conservation and Biology 7(3): 462–469. http://herpconbio.org/Volume_7/Issue_3/Wunder_etal_2012.pdf

Yang H, Baek H, Speare R, Webb R, Park S et al. (2009) First detection of the amphibian chytrid fungus *Batrachochytrium dendrobatidis* in free-ranging populations of amphibians on mainland Asia: survey in South Korea. Diseases of Aquatic Organisms 86: 9–13.

Young MK, Allison GT, Foster K (2007) Observations of boreal toads (*Bufo boreas boreas*) and *Batrachochytrium dendrobatidis* in south-central Wyoming and north-central Colorado. Herpetological Review 38: 146–150.

Zampiglia M, Canestrelli D, Chiocchio A, Nascetti G (2013) Geographic distribution of the chytrid pathogen *Batrachochytrium dendrobatidis* among mountain amphibians along the Italian peninsula. Diseases of Aquatic Organisms 107: 61–68. doi: 10.3354/dao02655

Zancolli G, Storfer A, Rödel M-O (2013) Detection of *Batrachochytrium dendrobatidis* in river frogs (genus *Amietia*) on Mount Kilimanjaro, Tanzania. Herpetological Review 44: 611–614.

Zevallos S, Elías RK, Berenguel RA, Weaver TJ, Reading RP (2016) *Batrachochytrium dendrobatidis* in confiscated *Telmatobius* in Lima, Peru. Journal of Wildlife Diseases 52(4): 949–952. doi: 10.7589/2016-01-006.

Zellmer AJ, Richards CL, Martens LM (2008) Low prevalence of *Batrachochytrium dendrobatidis* across *Rana sylvatica* populations in southeastern Michigan, USA. Herpetological Review 39: 196–199.

Zhu W, Bai C, Wang S, Soto-Azat C, Li X, Liu X, Li Y (2014) Retrospective survey of museum specimens reveals historically widespread presence of *Batrachochytrium dendrobatidis* in China. EcoHealth 11: 241–250. doi: 10.1007/s10393-013-0894-7

Zhu W, Fan L, Soto-Azat C, Yan S, Gao X, Liu X, Wang S, Liu C, Yang X, Li Y (2016) Filling a gap in the distribution of *Batrachochytrium dendrobatidis*: evidence in amphibians from northern China. Diseases of Aquatic Organisms 118: 259–266. doi: 10.3354/dao02975

Zimkus BM, Larson JG (2013) Assessment of the amphibians of Batéké Plateau National Park, Gabon, including results of chytrid pathogen tests. Salamandra 49(3): 159–170.

Zippel KC, Tabaka C (2008) Amphibian chytridiomycosis in captive *Acris crepitans blanchardi* (Blanchard’s Cricket Frog) collected from Ohio, Missouri, and Michigan, USA. Herpetological Review 39: 192–193.

Zumbado-Ulate H, Bolaños F, Gutiérrez-Espeleta G, Puschendorf R (2014) Extremely low prevalence of *Batrachochytrium dendrobatidis* in frog populations from Neotropical dry forest of Costa Rica supports the existence of a climatic refuge from disease. EcoHealth 11: 593–602. doi: 10.1007/s10393-014-0967-2

Zumbado-Ulate H, García-Rodríguez A, Vredenberg VT, Searle C (2019) Infection with *Batrachochytrium dendrobatidis* is common in tropical lowland habitats: implications for amphibian conservation. Ecology and Evolution 9: 4917–4930. doi: 10.1002/ece3.5098

1. Formerly family Discoglossidae, before Frost et al. (2006). [↑](#footnote-ref-1)
2. No other species of *Astylosternus* have been tested in Nigeria, so this positive sample is included in the table. [↑](#footnote-ref-2)
3. Moved out of Leiopelmatidae since Olson et al. 2013 [↑](#footnote-ref-3)
4. Based on the location of the record, this is not *Nectophrynoides asperginis*, which has (or perhaps had) an extremely restricted range—the spray zone of a single waterfall in the Kihansi Gorge of the Udzungwa Mountains of Tanzania. [↑](#footnote-ref-4)
5. The family Strabomantidae was folded back into the Craugastoridae. [↑](#footnote-ref-5)
6. The family Craugastoridae is one of three subdivided by Hedges et al. (2008) from the family Brachycephalidae as assigned by Frost et al. (2006). All species in the genus *Craugastor* were formerly known as *Eleutherodactylus*, and in several cases were reported as such in their first occurrence with *Bd*. [↑](#footnote-ref-6)
7. This is the only positive sample from the genus *Noblella*. [↑](#footnote-ref-7)
8. All sp. nov. detections are included in the table and counted in the tallies, as they represent unnamed, new species not otherwise covered in the data herein. [↑](#footnote-ref-8)
9. Genus *Rhinoderma* moved to family Rhinodermatidae; genera *Odontophrynus* and *Proceratophrys* moved to family Odontophrynidae. [↑](#footnote-ref-9)
10. The species of *Hyloxalus* shown were formerly placed in the genus *Colostethus* (Frost 2008). [↑](#footnote-ref-10)
11. The species name for *Hyloxalus fascianiger* was corrected to *Hyloxalus fascianigrus* in Frost (2007). [↑](#footnote-ref-11)
12. Reported at www.google.com/hostednews/afp/article/ALeqM5iqFIa4AFOylJaFUUECJw0z0-99Rg [↑](#footnote-ref-12)
13. The family Eleutherodactylidae is one of three subdivided by Hedges et al. (2008) from the family Brachycephalidae as assigned by Frost et al. (2006). [↑](#footnote-ref-13)
14. All species now in the genus *Diasporus* were formerly known as *Eleutherodactylus*, and were reported as such in their first occurrence with *Bd*. [↑](#footnote-ref-14)
15. Families Phyllomedusidae and Pelodryadidae split off from Hylidae and are treated separately in this table. [↑](#footnote-ref-15)
16. http://www.jcu.edu.au/school/phtm/PHTM/frogs/chyspec.htm 26 January 2005 [↑](#footnote-ref-16)
17. This sample is included here because it is the only positive for the genus in Venezuela. [↑](#footnote-ref-17)
18. This is the only member of the genus *Leptolalax* sampled, and the only detection. [↑](#footnote-ref-18)
19. This is the only member of the genus *Ophryophryne* sampled, and the only detection. [↑](#footnote-ref-19)
20. This is the only member of the genus *Arcovomer* sampled, and the only detection. [↑](#footnote-ref-20)
21. Family Pelodryadidae recently separated from the Hyldae. [↑](#footnote-ref-21)
22. Some *Litoria jungguy* may originally have been reported as *Litoria lesueurii* or *L. wilcoxii*, if published before or circa the division among *L. lesueurii*, *L. wilcoxii*, and *L. jungguy*. [↑](#footnote-ref-22)
23. Some *Litoria wilcoxii* may originally have been reported as *Litoria lesueurii* or *L. jungguy*, if published before or circa the division among *L. lesueurii*, *L. wilcoxii*, and *L. jungguy*. [↑](#footnote-ref-23)
24. Formerly placed in the family Petropedetidae. [↑](#footnote-ref-24)
25. Formerly placed in the family Hylidae. [↑](#footnote-ref-25)
26. These were the only detections in the genus *Ptychadena* in Cameroon, though there were other samples with non-detections in the genus from the country. [↑](#footnote-ref-26)
27. Formerly placed in the family Ranidae. [↑](#footnote-ref-27)
28. Now treated as a (possibly extinct) subspecies of *Rana (Lithobates) chiricahuensis*. [↑](#footnote-ref-28)
29. This is the only positive sample from North Macedonia. [↑](#footnote-ref-29)
30. This is the only member of the genus *Philautus* sampled in Laos, and the only detection. A second unnamed species had a detection in Malaysia, but there were other positive *Philautus* in Malaysia, so the genus-only sample is not reported here. [↑](#footnote-ref-30)
31. Formerly family Dicamptodontidae. [↑](#footnote-ref-31)
32. Recently moved from the genus *Euproctus*, based on a new understanding of population and genetic relationships (Frost 2009). [↑](#footnote-ref-32)
33. Reported with *Bd* as *Triturus alpestris*, briefly *Mesotriton alpestris*, now (according to Frost 2009) *Ichthyosaura alpestris*. [↑](#footnote-ref-33)
34. Reported with *Bd* as *Triturus helveticus*; recently reclassified as *Lissotriton* (Frost 2009). [↑](#footnote-ref-34)
35. Reported with *Bd* as *Triturus vulgaris*; recently reclassified as *Lissotriton* (Frost 2009). [↑](#footnote-ref-35)
