# Supplementary material for: Global Patterns of the Fungal Pathogen Batrachochytrium dendrobatidis Support Conservation Urgency
Source: Front Vet Sci. 2021 Jul 16;8:685877. doi: 10.3389/fvets.2021.685877 (PMC8322974; doi:10.3389/fvets.2021.685877)
Supplement: Supplementary Appendix 1 — Detailed methods. [file Data_Sheet_1.zip › Data Sheet 1/Table S3.docx]

**Table S3.** *Batrachochytrium dendrobatidis* (*Bd*) detections by country, with source citations (see Data References following Table S2,). Countries with detections are shown in **bold face**. CLO = country-level location only, designating country centroid records. Cap. = captive sample. Countries with only country-level records are shown in grey; all of them have only negative samples.

| **Country sampled** | **Sources reporting detections** | **Sources reporting only non-detections** |
| --- | --- | --- |
| **Albania** | Vojar et al. 2017 |  |
| **Argentina** | Agostini and Burrowes 2015; Agostini et al. 2015; Arellano et al. 2009, 2017; Barrionuevo and Mangione 2006; Delgado et al. 2012; Fox et al. 2006; Ghirardi 2011; Ghirardi et al. 2009, 2011, 2014, 2017a, 2017b, 2018; Gutierrez et al. 2010; Herrera et al. 2005; Lescano et al. 2013 | Barrasso et al. 2009; Barrionuevo and Ponssa 2008 |
| Armenia |  | Ouellet et al. 2005 (CLO, 2 spp.) |
| **Australia** | Aziz et al. 2011; Berger et al. 1998, 1999; Brannelly et al. 2015, 2016; Briggs and Burgin 2004; Catullo et al. 2018; Clemann et al. 2009; Edwards et al. 2019; Gillespie et al. 2015; Grogan et al. 2016; Hagman and Alford 2015; Heard et al. 2014; Howard et al. 2010, 2012; Hunter et al. 2009, 2010; Kindermann et al. 2012, 2017; Klop-Toker et al. 2016; Kriger and Hero 2006, 2007a, 2007b, 2008; Kriger et al. 2007; Letoof et al. 2013; Morehouse et al. 2003; Murray et al. 2010, 2013; Narayan et al. 2014; North and Alford 2008; Obendorf 2005; Obendorf and Dalton 2006; Ocock et al. 2013; Pauza and Driessen 2008; Pauza et al. 2010; Phillott et al. 2013; Puschendorf et al. 2011, 2013; Retallick et al. 2004; Riley et al. 2013; Rowley et al. 2007a; Roznik et al. 2015; Sapsford et al. 2013, 2015; Scheele et al. 2014, 2015, 2016, 2017; Simpkins et al. 2010, 2017; Skerratt et al. 2010; Speare and Berger 2005; Stockwell et al. 2015, 2016; Symonds et al. 2007; Van Sluys and Hero 2009; Vörös et al. 2011; Woodhams et al. 2010 | Hauselberger and Alford 2012; Hoskin et al. 2018; McLelland et al. 2013; Murray et al. 2009 |
| **Austria** | Sztatecsny and Glaser 2011 | Lötters et al. 2012; Sztatecsny and Hodl 2009; Garner et al. 2005 (CLO, 1 sp.) |
| Barbados |  | Ouellet et al. 2005 (CLO, 1 sp.) |
| **Belgium** | Martel et al. 2012; Spitzen-van der Sluijs et al. 2014; Mutschmann et al. 2000 (cap., CLO); Pasmans et al. 2004 (CLO, cap.), 2010 (CLO); An Martel (unpub.); |  |
| **Belize** | Kaiser and Pollinger 2012 |  |
| Benin |  | Penner et al. 2013; Mark-Oliver Rödel, unpub. (CLO, 5 spp.) |
| **Bolivia** | Barrionuevo et al. 2008; De la Riva and Burrowes 2011; Burrowes and De la Riva 2017; Burrowes et al. 2020; Cossel et al. 2014; Lindquist et al. 2016 |  |
| Bosnia and Herzegovina |  | Šunje et al. 2018 |
| **Botswana** | Weldon 2005 | Vredenburg et al. 2013 |
| **Brazil** | Amorim et al. 2019; Carnaval et al. 2005, 2006; Carvalho et al. 2017; Coutinho et al. 2015; De Paula et al. 2012; Ferriera et al. 2008; Garner et al. 2006; Gründler et al. 2012; Jenkinson et al. 2016; Lambertini et al. 2016, 2017; Navarro-Lozano et al. 2018; Rodriguez et al. 2014; Ruano-Fajardo et al. 2016; Ruggeri et al. 2015, 2018a, 2018b; Schloegel et al. 2010, 2012; Toledo et al. 2006a, 2006b; Valencia-Aguilar et al. 2015, 2016; Van Sluys et al. 2007; Vieira et al. 2012, 2013 |  |
| Brunei |  | Kaiser and Grafe 2011 |
| Burkina Faso |  | Penner et al. 2013; Mark-Oliver Rödel, unpub. (CLO, 2 spp.) |
| **Cambodia** | Gaertner et al. 2011; Gilbert et al. 2012; Mendoza et al. 2011 | Rowley et al. 2013; Swei et al. 2011 |
| **Cameroon** | Baláž et al. 2012; Doherty-Bone et al. 2013; Gower et al. 2013; Hirschfeld et al. 2016; Miller et al. 2018; Soto-Azat et al. 2009 | Blackburn et al. 2010; Doherty-Bone et al. 2008 |
| **Canada** | Adams et al. 2007; Charbonneau 2006; D’Aoust-Messier et al. 2015; Deguise and Richardson 2009; Forzán et al. 2010; Garner et al. 2006; Jongsma et al. 2019; McMillan et al. 2019; Morehouse et al. 2003; Ouellet et al. 2005; Raverty and Reynolds 2001; Richardson et al. 2014; Schock et al. 2010; Slough 2009; St-Amour et al. 2010; Stevens et al. 2012; Voordouw et al. 2010; Canadian Cooperative Wildlife Health Centre, unpub.; Kris Kendell, unpub.; Northern Leopard Frog Recovery Team, unpub.; Alan Pessier, unpub.; Valerie St-Amour, unpub.; TWJ Garner unpub.; | Paetow et al. 2012 |
| Central African Republic |  | Mark-Oliver Rödel, unpub. (CLO, 3 spp.) |
| **Chile** | Bacigalupe et al. 2017; Bourke et al. 2010, 2011; Fenolio et al. 2013; Solís et al. 2010, 2015; Soto-Azat et al. 2013, 2016; Valenzuela-Sánchez et al. 2017; Roberto Solís, unpub. |  |
| **China** | Bai et al. 2010, 2012; Zhu et al. 2014, 2016 | Ouellet et al. 2005; Swei et al. 2011; Wei et al. 2010 |
| **Colombia** | Acevedo et al. 2016; Flechas et al. 2012, 2013, 2015, 2017; Urbina and Galeano 2011; Vásquez-Ochoa et al. 2012; Velásquez-E. et al. 2008; Laboratorio de Herpetologica, Beatriz E. Velásquez-E., unpub. | Gower et al. 2013; Ruiz and Rueda-Almonacid 2008; Canadian Cooperative Wildlife Health Centre, unpub.; |
| **Costa Rica** | Cheng et al. 2011; Goldberg et al. 2009; Lips et al. 2003, 2006; Picco and Collins 2007; Puschendorf 2003; Puschendorf et al. 2006a, 2009; Saenz et al. 2009; Whitfield et al. 2012, 2013, 2017; Zumbado-Ulate et al. 2014, 2019; Caren Goldberg, Tanya Hawley and Lisette Watts, unpub. | Govindarajulu et al. 2006; Puschendorf et al. 2006b; Richards-Hrdlicka 2013 |
| Cote D’Ivoire |  | Penner et al. 2013; Mark-Oliver Rödel, unpub. (CLO, 8 spp.) |
| Croatia |  | Baláž et al. 2014; Garner et al. 2005; Lastra González et al. 2019; Vörös and Jelić 2011 |
| **Cuba** | Cádiz et al. 2018; Díaz et al. 2007; Sabino-Pinto et al. 2017; (?) Rodriguez, unpub. |  |
| **Czech Republic** | Baláž et al. 2013, 2014a, 2014b; Civiš et al. 2012; Havlíková et al. 2015 (CLO, cap.); Lastra González et al. 2019 | Chajma and Vojar 2016; Garner et al. 2005 (CLO, 1 sp.); Ouellet et al. 2005 (CLO, 1 sp.) |
| **Democratic Republic of the Congo** | Greenbaum et al. 2008, 2014, 2015; Seimon et al. 2015 | Vredenburg et al. 2013 |
| **Denmark** | Scalera et al. 2008; Uli Reyer, unpub. | Muths et al. 2009 |
| **Dominica** | Hudson et al. 2019; Malhotra et al. 2007; MC Fisher, unpub.; |  |
| **Dominican Republic** | Joglar et al. 2007 |  |
| **Ecuador** | Berger et al. 1999; Bresciano et al. 2015; McCracken et al. 2009; Ron and Merino-Viteri 2000; Tarvin et al. 2014 |  |
| **El Salvador** | Felger et al. 2007; Lawson et al. 2011 |  |
| Estonia |  | Baláž et al. 2014 |
| **Ethiopia** | Gower et al. 2012 |  |
| Fiji |  | Narayan et al. 2011; Ouellet et al. 2005 (CLO) |
| Finland |  | Patrelle et al. 2012 |
| **France** | Garner et al. 2006; Michaels et al. 2018; Ouellet et al. 2012; Susan Walker, unpub.; Matthew Fisher, unpub. | Miaud et al. 2016; Pasmans et al. 2013; Garner et al. 2005 (CLO) |
| **French Guiana** | Courtois et al. 2015; Rendle et al. 2015 | Gower et al. 2013 |
| **Gabon** | Bell et al. 2011; Jongsma et al. 2016 | Bell et al. 2012; Daversa et al. 2011; Gratwicke et al. 2011; Zimkus and Larson 2013 |
| Gambia |  | Mark-Oliver Rödel, unpub. (CLO, 2 spp.) |
| **Germany** | Böll et al. 2012; Lötters et al. 2018; Mutschmann et al. 2000; Ohst et al. 2013; Rasmussen et al. 2012; Wagner et al. 2017 | Lötters et al. 2012; Garner et al. 2005 (CLO, 1 sp.) |
| Ghana |  | Penner et al. 2013; Vredenburg et al. 2013; Mark-Oliver Rödel, unpub. (CLO, 7 spp.) |
| **Greece** | Azmanis et al. 2016 | Garner et al. 2005 |
| Grenada |  | Drake et al. 2014 |
| **Guatemala** | Cheng et al. 2011; Mendelson et al. 2005, 2014; Rovito et al. 2009 |  |
| Guinea |  | Penner et al. 2013; Eli Greenbaum, unpub.; Mark-Oliver Rödel, unpub. (CLO, 3 spp.) |
| Guyana |  | Gower et al. 2013; Mathie et al. 2018 |
| **Honduras** | Blooi et al. 2017; Gutsche et al. 2015; Kolby and Padgett-Flohr 2009; Kolby et al. 2010, 2015b; Puschendorf et al. 2006b |  |
| Hong Kong^[[1]](#footnote-1)^ | Kolby et al. 2014 (cap.) | Rowley et al. 2007b (cap./wild) |
| **Hungary** | Baláž et al. 2014; Garner et al. 2005; Gál et al. 2012; Vörös et al. 2018; J. Vörös, E. Sos, R. Dankovics, A. Hettyey, F. Hock, unpub. |  |
| **India** | Dahanukar et al. 2013; Molur et al. 2015; Mutnale et al. 2018; Nair et al. 2011 |  |
| **Indonesia** | Kusrini et al. 2008; Swei et al. 2011 |  |
| Iran |  | Ouellet et al. 2005 (CLO, 1 sp.) |
| Ireland |  | Gandola and Hendry 2013 |
| **Italy** | Adams et al. 2008; Bielby et al. 2009, 2013; Bovero et al. 2008; Canestrelli et al. 2013; Di Rosa et al. 2007; Federici et al. 2008; Garner et al. 2005, 2006; Grasselli et al. 2019; Simoncelli et al. 2005; Stagni et al. 2000, 2004; Zampiglia et al. 2013; John Bielby, unpub.; MC Fisher, unpub. | Canessa et al. 2013; Chiari et al. 2013 (Sardinia); Ficetola et al. 2011; Pasmans et al. 2013 |
| **Jamaica** | Holmes et al. 2012, 2014 | Ouellet et al. 2005 (CLO, 1 sp.) |
| **Japan** | Goka et al. 2009; Kadekaru et al. 2016; Rios-Sotelo et al. 2018; Tamukai et al. 2014 (CLO, cap.); Une et al. 2008; Sumio Okada, unpub. (cap.); http://www.promedmail.org | Garner et al. 2006 |
| Kazakhstan |  | Swei et al. 2011 |
| **Kenya** | Berger and Speare 2000; Kielgast et al. 2009; Vredenburg et al. 2013; chyglob.htm |  |
| **Kyrgyzstan** | Swei et al. 2011 | Ouellet et al. 2005 (CLO, 1 sp.) |
| **Laos** | Swei et al. 2011 | Gilbert et al. 2012 |
| Latvia |  | Ouellet et al. 2005 (CLO, 2 spp.) |
| **Lesotho** | Weldon 2005; Mark-Oliver Rödel, unpub. (CLO), 1 spp. |  |
| Liberia |  | Penner et al. 2013; Mark-Oliver Rödel, unpub. (CLO, 2 spp.) |
| **Luxembourg** | Wood et al. 2009 |  |
| Madagascar^[[2]](#footnote-2)^ | *Bletz et al. 2015,* Kolby 2014 (cap.) | Crottini et al. 2011, 2014; Kolby et al. 2015a; Rabemananjara et al. 2011; Vredenburg et al. 2012; Weldon et al. 2008, 2014 |
| **Malawi** | Conradie et al. 2011; Soto-Azat et al. 2009 |  |
| **Malaysia** | Leblanc et al. 2014; Savage et al. 2011; Swei et al. 2011 | Kaiser and Grafe 2011 |
| **Mexico** | Basanta et al. 2019; Cheng et al. 2011; Frías -Alvarez et al. 2008; Galindo-Bustos et al. 2014; Hale et al. 2005; Hernández-Martínez et al. 2019; Huss et al. 2013; Köhler et al. 2016, 2019a; Luja et al. 2012; Luría-Manzano et al. 2011; Mendoza-Almeralla et al. 2016; Murrieta-Galindo et al. 2014; Peralta-García et al. 2018; Santos Barrera and Peralta-García 2018; Van Rooij et al. 2011 Velo-Antón 2012; David E. Green, unpub. | Rovito et al. 2009; Villamizar-Gómez et al. 2015; Jessi Krebs, unpub. |
| Mongolia |  | Swei et al. 2011 |
| **Montenegro** | Lastra González et al. 2019; Vojar et al. 2017 |  |
| **Montserrat** | Garcia et al. 2009; Hudson et al. 2019 | Garcia et al. 2007 |
| **Morocco** | El Cadi et al. 2019; El Mouden et al. 2011; Jaime Bosch, unpub. |  |
| **Mozambique** | Conradie et al. 2016 |  |
| Myanmar |  | Köhler et al. 2019b; Swei et al. 2011 |
| Namibia |  | Vredenburg et al. 2013 |
| **Netherlands** | Kik et al. 2012 (CLO, cap.); Spitzen-van der Sluijs et al. 2011 (cap.), 2014 (wild) |  |
| **New Zealand** | Bell et al. 2004; Shaw et al. 2013; Waldman et al. 2001; Amanda Haigh, unpub. |  |
| **Nicaragua** | García-Roa et al. 2014; Stark et al. 2017 |  |
| **Nigeria** | Imasuen et al. 2011; Reeder et al. 2011 | Vredenburg et al. 2013; Ouellet et al. 2005 (CLO, 1 sp.) |
| **North Korea** | Fong et al. 2015 |  |
| **North Macedonia** | Vojar et al. 2017 |  |
| Oman |  | Chaber et al. 2016 |
| Pakistan |  | Swei et al. 2011; unpub. data, uploaded without contact info |
| **Panama** | Berger et al. 1998; Brem and Lips 2008; Hertz et al. 2018; Kilburn et al. 2010; Lindquist et al. 2011; Lips et al. 2006; Morehouse et al. 2003; Rebollar et al. 2014; Richards-Zawacki 2010; Rodríguez-Brenes et al. 2016; Velo-Antón 2012; Woodhams et al. 2008 | Lips et al. 2003 |
| Papua New Guinea |  | Dahl et al. 2012; Ralph Cutter, unpub.; Ouellet et al. 2005 (CLO, 1 sp.) |
| **Peru** | Berenguel et al. 2016; Burkart et al. 2017; Catenazzi et al. 2010, 2011, 2013, 2014; Kosch et al. 2012; Lötters et al. 2005; Rubio et al. 2018; Russell et al. 2019; Seimon et al. 2007, 2017; Venegas et al. 2008; Warne et al. 2016; O. De la Riva, unpub. | Enciso et al. 2008 |
| **Philippines** | Swei et al. 2011; Smith et al. 2019; Agence France-Presse (Arvin Diesmos and Rafe Brown) |  |
| **Poland** | Czeczuga et al. 2011; Kolenda et al. 2017; Sura et al. 2010 (CLO, 1 sp.) | Lastra González et al. 2019 |
| **Portugal** | Rosa et al. 2013, 2017; Garner et al. 2005 (CLO, 1 sp.); MC Fisher, unpub. |  |
| **Puerto Rico** | Barber 2012; Burrowes et al. 2008; Longo and Burrowes 2010; Longo and Zamudio 2017; Longo et al. 2010, 2013; David E. Green, unpub.; P. Burrowes, A. Longo, R Joglar, unpub. |  |
| Republic of the Seychelles |  | Labisko et al. 2015 |
| **Romania** | Scheele et al. 2015a (Anim. Cons.); Vörös et al. 2013 |  |
| **Russian Federation** | Reshetnikov et al. 2014 | Civiš et al. 2013; Ouellet et al. 2005 (CLO, 2 spp.) |
| **Rwanda** | Seimon et al. 2015; van der Hoek et al. 2019 | Vredenburg et al. 2013 |
| **Sao Tome and Principe** | Hydeman et al. 2013 |  |
| **Serbia** | Mali et al. 2017 |  |
| Sierra Leone |  | Penner et al. 2013; Mark-Oliver Rödel, unpub. (CLO, 4 spp.) |
| **Singapore** | Chong et al. 2018; Gilbert et al. 2012 |  |
| **Slovakia** | Baláž et al. 2014b | Lastra González et al. 2019 |
| Slovenia |  | Baláž et al. 2014b; Garner et al. 2005 |
| **South Africa** | Conradie et al. 2011; Hopkins and Channing 2003; Lane et al. 2003; Smith et al. 2007; Soto-Azat et al. 2009; Tarrant et al. 2013; Weldon 2005; chyglob.htm; C. Weldon, unpub. | Vredenburg et al. 2013; Mark-Oliver Rödel, unpub. (CLO, 1 sp.) |
| **South Korea** | An and Waldman 2016; Bataille et al. 2013; Borzée et al. 2017; Shin et al. 2014; Swei et al. 2011; Yang et al. 2009 | Fong et al. 2015 |
| **Spain** | Bosch and Martinez-Solano 2006; Bosch et al. 2001, 2013; Fernández -Beaskoetxea et al. 2015; Fernández-Loras et al. 2019; Gabor et al. 2013, 2017; Garner et al. 2009; Hidalgo-Vila et al. 2012; Lastra González et al. 2019; Medina et al. 2015; Oficialdegui et al. 2019; Walker et al. 2008; Jaime Bosch, unpub.; Susan Walker, unpub. | Baláž et al. 2014b; Obon et al. 2013; Steiner and Lehtinen 2008 |
| **Sri Lanka** | Swei et al. 2011 |  |
| **St. Vincent and the Grenadines** | Sweeney 2016 |  |
| Suriname |  | Becker et al. 2016 |
| **Swaziland (Eswatini)** | Weldon 2005 |  |
| **Sweden** | Kärvemo et al. 2018, 2019 | Garner et al. 2005 (CLO, 1 sp.) |
| **Switzerland** | Garner et al. 2005; Lötters et al. 2012; Tobler and Schmidt 2010; Tobler et al. 2012 |  |
| Taiwan |  | Lehtinen et al. 2008 |
| **Tanzania** | Gower et al. 2013; Makange et al. 2014; Moyer and Weldon 2006; Weldon and du Preez 2004; Weldon et al. 2019; Zancolli et al. 2013 | Vredenburg et al. 2013 (CLO, 1 sp.); TWJ Garner, unpub. |
| **Thailand** | Techangamsuwan et al. 2017; Vörös et al. 2012 | Freitas et al. 2019; McLeod et al. 2008 |
| **Trinidad and Tobago** | Alemu et al. 2008, 2013; Patel et al. 2012 | Greener et al. 2017; Richard Lehtinen, unpub. |
| **Turkey** | Erismis et al. 2014; Göçmen et al. 2013 |  |
| **Uganda** | Goldberg et al. 2007; Seimon et al. 2015; Soto-Azat et al. 2009; Viertel et al. 2012 |  |
| United Arab Emirates |  | Chaber et al. 2016; Soorae et al. 2012 |
| **United Kingdom** | Arai 2008; Cunningham and Minting 2008; Cunningham et al. 2005; Garner et al. 2005; Michaels et al. 2018 (cap.); Tinsley et al. 2015; Wombwell et al. 2016; TWJ Garner, unpub. | Allain and Goodman 2017; May et al. 2011; Gandola and Hendry 2013 (Northern Ireland) |
| **United States** | AJ Adams et al. 2017a, 2017b; MJ Adams et al. 2007, 2010; Addis et al. 2015; Alminas et al. 2010; Araos et al. 2017; Augustine and Neff 2016; Bakkegard and Pessier 2010; Bakland 2018; Bartkus 2009; Bales et al. 2015; Battaglin et al. 2016; Bauer et al. 2018 (cap.); Beard and O’Neill 2005; Becker et al. 2012; Beyer et al. 2015; Bettaso and Rachowicz 2006; Blackburn et al. 2015; Blackley 2016; Bletz and Harris 2013; Bodinof et al. 2011; Boivin 2012; Bradley et al. 2002; Brannelly et al. 2012, 2018; Briggler et al. 2008; Briggs et al. 2010; Brocco 2017; Brodman and Briggler 2008; Brown and Kerby 2013; Brown et al. 2019; Byrne et al. 2008; Carey and Livo 2009; Carey et al. 1999; Caruso and Lips 2013; Chatfield et al. 2009, 2013; Chiari et al. 2017; Chinnadurai et al. 2009; Churgin et al. 2013 (cap.); chyglob.html; Daszak et al. 2005; Davidson and Chambers 2011; Davidson et al. 2003; Davis et al. 2012; De Léon et al. 2017; Drake et al. 2007; Duncan Pullen et al. 2010; Ecoclub Amphibian Group et al. 2016; Eskew et al. 2014; Fellers et al. 2001, 2011; Firkins 2015; Forrest and Schlaepfer 2011; Forrest et al. 2015; Gaertner et al. 2007, 2009a, 2009b, 2010, 2012; Garner et al. 2006; Gaudreau et al. 2010; Gaulke et al. 2011; Glorioso et al. 2017; Gluesenkamp et al. 2018; Gonyor et al. 2011; Goodman and Ararso 2012; Goodman et al. 2019; Campbell Grant et al. 2008; Gratwicke et al. 2011; Green and Dodd 2007; Green and Kagarise Sherman 2001; Green and Muths 2005; Groner and Relyea 2010; Hanlon et al. 2014; Harner et al. 2011, 2013; Hasken et al. 2009; Hayes et al. 2009; Hill and Levy 2014; Hill et al. 2011; Horner et al. 2017; Hossack et al. 2010; Huang and Wilson 2013; Hughey et al. 2014; Huss et al. 2013; Hyman and Collins 2012; Igleski and Nicholson 2014; Jaeger et al. 2017; Jenkinson et al. 2016; Johnson et al. 2018; Julian et al. 2016, 2019; Kiemnec-Tyburczy et al. 2012; Kinney et al. 2011, 2012; Klemish et al. 2012; Korfel and Hetherington 2014; Krynak et al. 2012; Lannoo et al. 2011; Lauer et al. 2007; Lenker et al. 2014; Longcore et al. 2007; Longo et al. 2019; Love et al. 2016; Lovich et al. 2008; Lowe 2009; Marhanka et al. 2017; Marshall et al. 2019; McTaggart et al. 2014; Moffitt et al. 2015; Monsen-Collar et al. 2010; Montanucci 2009; Morehouse et al. 2003; Morell 1999; Morgan et al. 2007; Mosher et al. 2018; Mowry et al. 2017; Muelleman and Montgomery 2013; Muletz-Wolz et al. 2019; BG Murphy et al. 2015; PJ Murphy et al. 2009; Muths et al. 2003, 2008; Newman et al. 2019; Olori et al. 2018; Padgett-Flohr and Longcore 2005, 2007; Parker et al. 2002; Patillo and Parris 2016; Pearl and Green 2005; Pearl et al. 2007, 2009; Pessier et al. 1999; Petersen et al. 2016; Peterson and McKenzie 2014; Peterson et al. 2007; Phillips et al. 2014; Pilliod et al. 2010; Piovia-Scott et al. 2011; Rachowicz et al. 2006; Raffel et al. 2010; Reeder et al. 2012; Reeves 2008; Reeves and Green 2006; Reeves et al. 2017; Regester et al. 2012, 2016; Richards-Hrdlicka et al. 2013; Rimer and Briggler 2010; Rittmann et al. 2003; Rivera et al. 2019; Rizkalla 2009, 2010; Robinson et al. 2018; Rodriguez et al. 2009; Rogers and Banulis 2004; Rollins et al. 2013; Rosen and Schwalbe 2002; Ross et al. 2014; Roth et al. 2013; Rothermel et al. 2008, 2013, 2016; Russell et al. 2010; Sacerdote-Velat et al. 2016; Sadinski et al. 2010; Saenz et al. 2010, 2015; Savage et al. 2011; Schlaepfer et al. 2007; Schloegel et al. 2009 (cap.); Seeley et al. 2016; Sette et al. 2015; Smith et al. 2017; Sonn et al. 2019; Souza et al. 2012; Spaulding et al. 2018; Sredl and Caldwell 2000; Sredl et al. 2002; Steiner and Lehtinen 2008; Stutz et al. 2017; Suriyamongkol et al. 2019; Talbott et al. 2018; Talley et al. 2011, 2015; Tatarian and Tatarian 2010; Terrell et al. 2014; Thompson et al. 2004; Timpe et al. 2008; Todd et al. 2019; Todd-Thompson et al. 2009; Tominaga et al. 2013; Tupper et al. 2011, 2014, 2017; Urbina et al. 2018; Velo-Antón 2012; Venesky and Brem 2008; Vieira et al. 2013; Villamizar-Gómez et al. 2016; Vredenburg et al. 2010, 2013; Watters et al. 2016, 2018, 2019; Weinstein 2009; Williams and Groves 2014; Wilson et al. 2015; Wimsatt et al. 2014; Windstam and Olori 2014; Wixson and Rogers 2009; Wolff et al. 2012, 2014; Woodhams et al. 2007, 2008; Wunder et al. 2012; Young et al. 2007; Zellmer et al. 2008; Zippel and Tabaka 2008; Amy Lind and Rob Grasso, unpub.; Alan Pessier, unpub.; Arizona Game & Fish Dept., unpub., Bryce Maxell, unpub.; Cathy Brown, unpub.; Cynthia Tait, unpub.; Caren Goldberg and Lisette Watts, unpub.; D. McGriff, unpub.; David Pilliod and Erin Muths, unpub.; D. Sumerlin, unpub.; D.E. Green, unpub.; Jennifer Loda, unpub.; Jason Nachtmann, unpub.; Joy Ware, unpub.; Evelyn Bull, unpub.; Erin Muths, unpub.; J. Mendelson, unpub.; G. Padgett-Flohr, unpub.; G. Lipps, unpub.; Gibbons et al., unpub.; J. Kolby, unpub.; J. Longcore, unpub.; J. Lowe, unpub.; J. Moore, unpub.; Janice Engle, unpub; Jessi Krebs, unpub.; Karen Pope unpub.; K. DiLeo, unpub.; J.C. Mitchell, DE Green, unpub.; N. Nieto, unpub.; P. Johnson, unpub.; P. Rosen and D. Caldwell, unpub.; R. Fisher, unpub.; R. Knapp, unpub., M. Hahr, unpub.; S. Kupferberg, unpub.; S. Muskopf, unpub.; S. Wagner et al. unpub.; T. James, unpub.; T. Jones, unpub.; Tulsa Zoo, unpub.; USFS Sierra Nevada Amphibian Monitoring Program, unpub.; V. Hemingway, unpub.; US Fish & Wildlife Service, unpub. | Becker and Harris 2010; Blackburn 2001; Chestnut et al. 2008; Glenney et al. 2010; Hale et al. 2005; Keitzer et al. 2011; Malhotra et al. 2007; Fenolio et al. 2013; Grummer and Leaché 2016; Guthrie et al. 2017; Isidoro-Ayza et al. 2019; Muletz et al. 2014; Polasik et al. 2016; Ramesh et al. 2013; Schrenker 2017; Thompson et al. 2019; Waddle et al. 2019; D. Bradford, unpub.; Doug Woodhams, unpub.; K. DiLeo, unpub. |
| **Uruguay** | Bardier et al. 2011; Borteiro et al. 2009, 2014; Garner et al. 2006; Lambertini et al. 2017; Laufer et al. 2018 |  |
| **Venezuela** | Bonaccorso et al. 2003; Hanselmann et al. 2004; Lampo et al. 2006a, 2006b, 2008, 2017; Nicolás 2007; Rodríguez-Contreras et al. 2008; Sánchez et al. 2008; J. Longcore, unpub. | Lampo and Senaris 2006; Márquez et al. 2010 |
| **Vietnam** | Rowley et al. 2013; Swei et al. 2011 | Gilbert et al. 2012; Thien et al. 2013 |
| Zambia |  | Vredenburg et al. 2013; M.C. Fisher, unpub. |

1. We did not count country as having *Bd* detected if the only positive sample came from a captive animal. [↑](#footnote-ref-1)
2. The positive field results for Madagascar have been called into question. Other positive samples were for captive animals, and we do not count a country as having a detection based only on captive animals. See Discussion. [↑](#footnote-ref-2)
