# Supplementary figures and images for: Global Patterns of the Fungal Pathogen Batrachochytrium dendrobatidis Support Conservation Urgency
Source: Front Vet Sci. 2021 Jul 16;8:685877. doi: 10.3389/fvets.2021.685877 (PMC8322974; doi:10.3389/fvets.2021.685877)

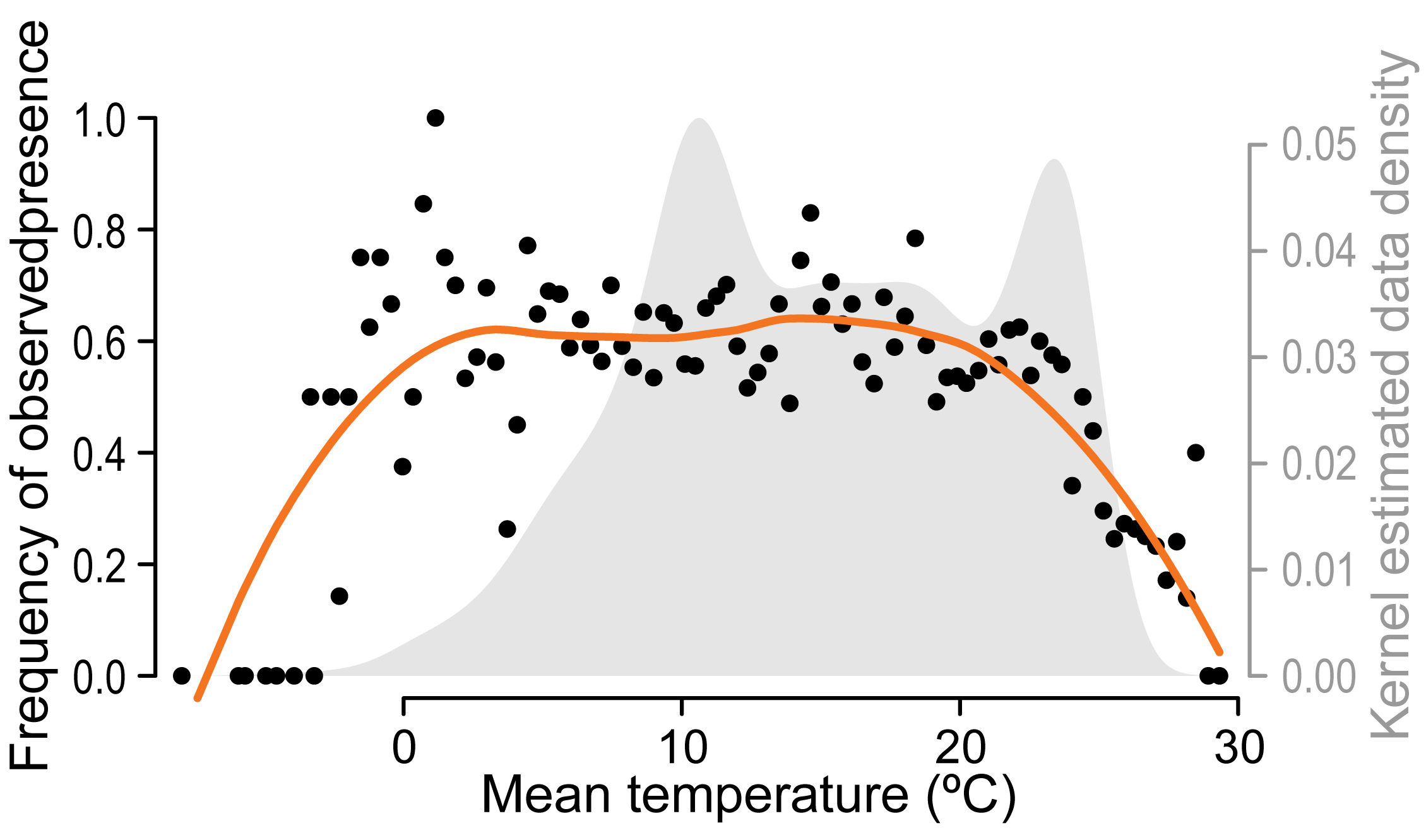

Supplement: Supplementary Appendix 1 — Detailed methods. [file Data_Sheet_1.zip › Data Sheet 1/Figure S2.tif]

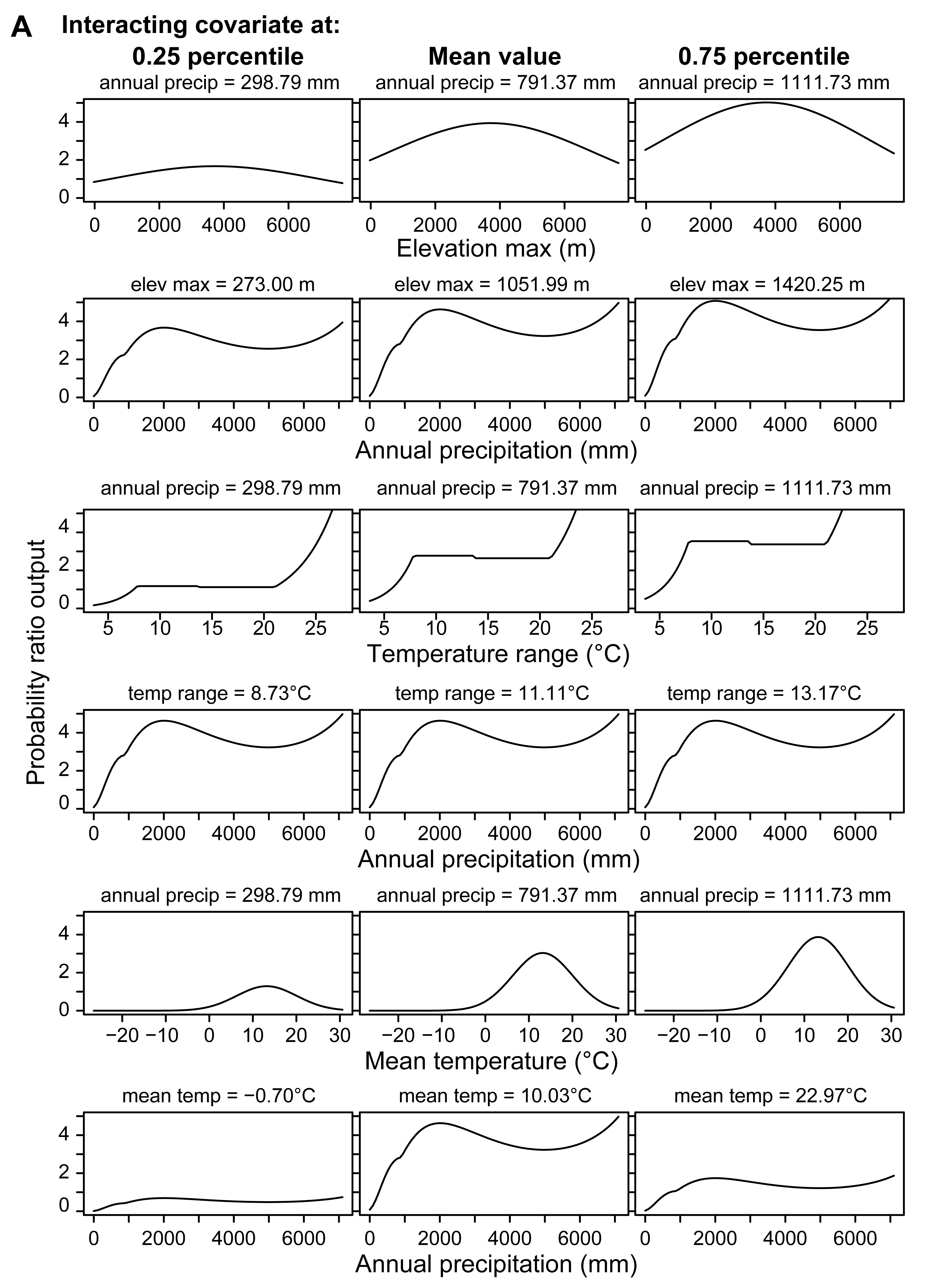

Supplement: Supplementary Appendix 1 — Detailed methods. [file Data_Sheet_1.zip › Data Sheet 1/Figure S3A.tif]

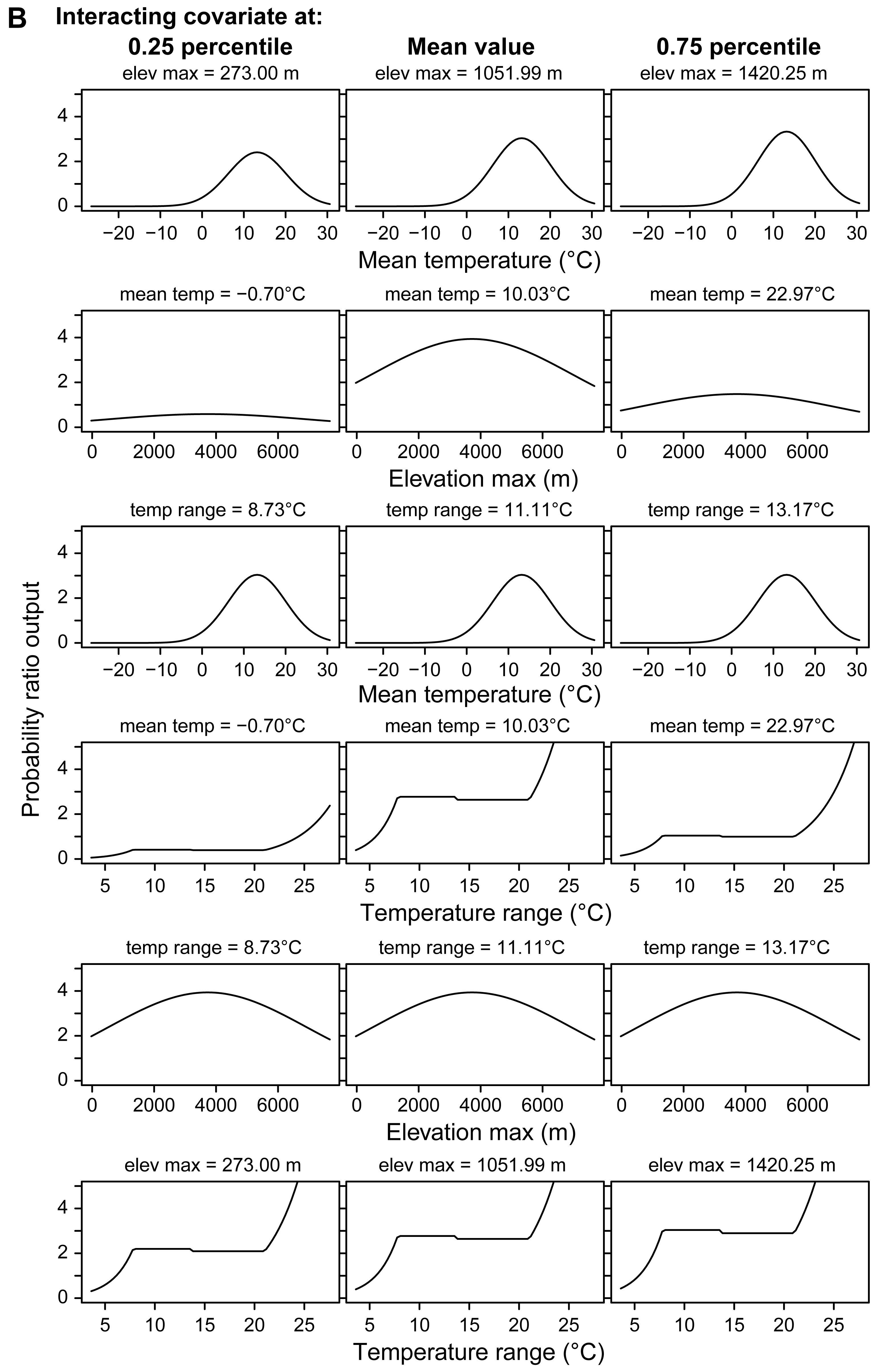

Supplement: Supplementary Appendix 1 — Detailed methods. [file Data_Sheet_1.zip › Data Sheet 1/Figure S3B.tif]

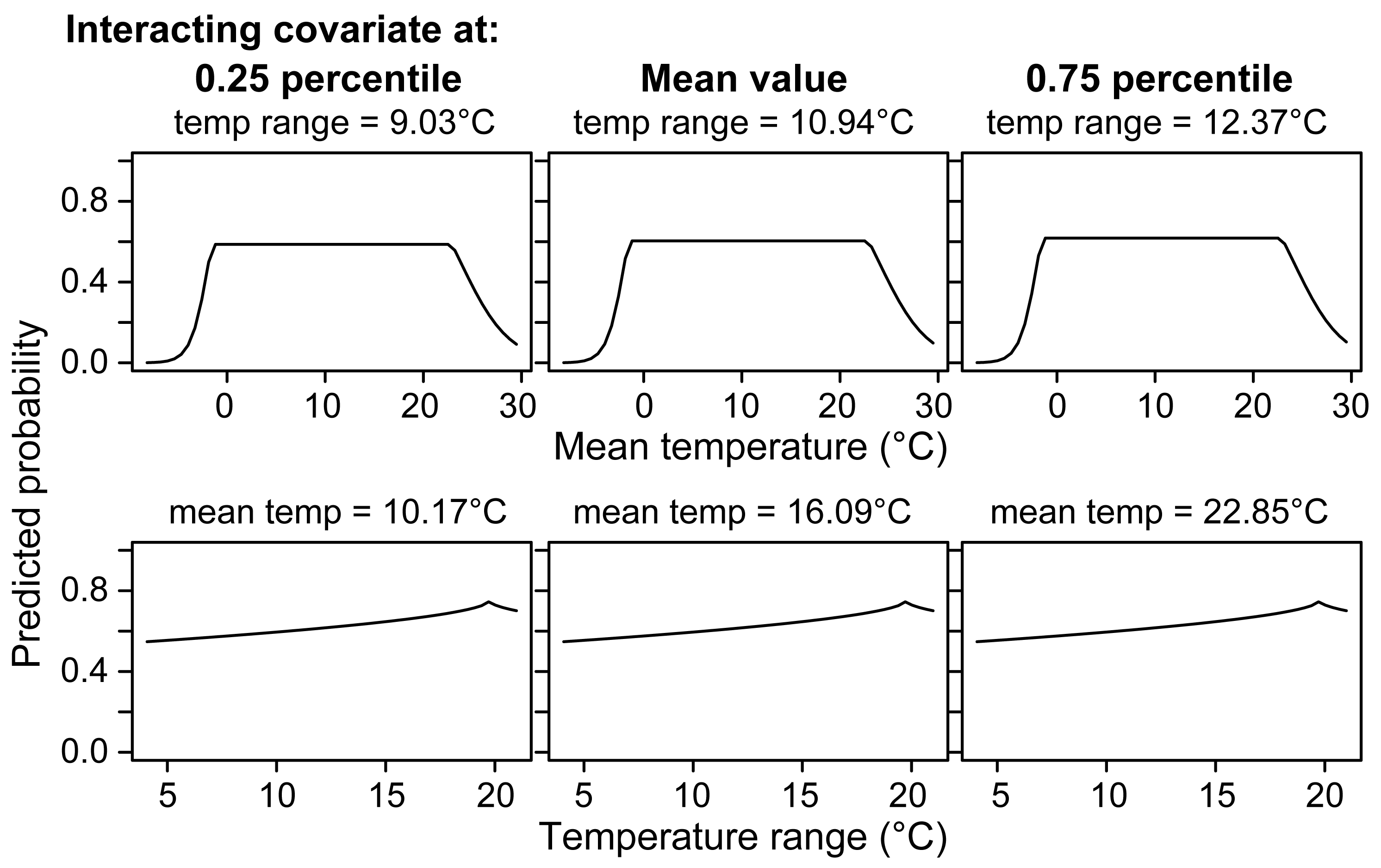

Supplement: Supplementary Appendix 1 — Detailed methods. [file Data_Sheet_1.zip › Data Sheet 1/Figure S4.tif]

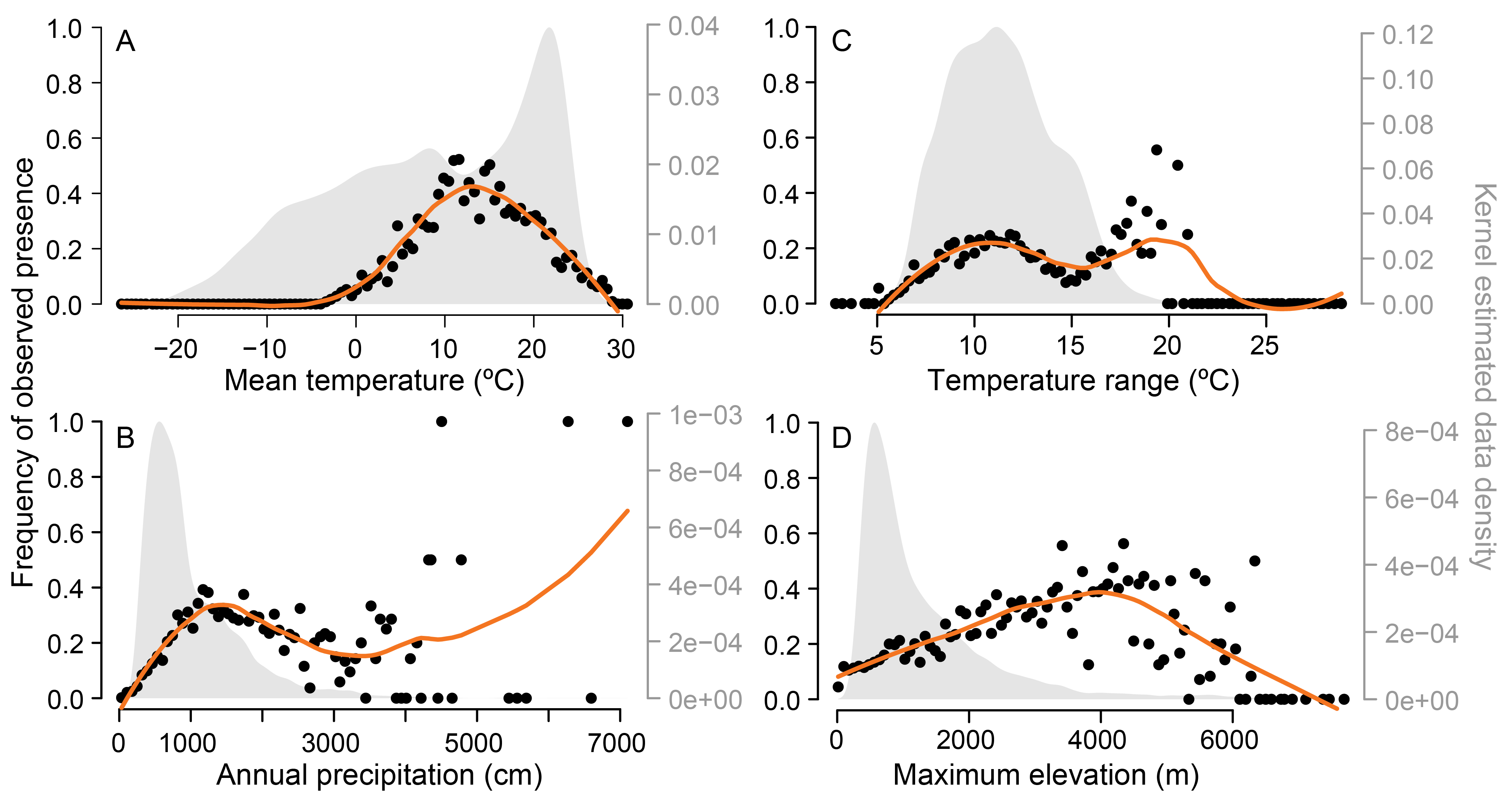

Supplement: Supplementary Appendix 1 — Detailed methods. [file Data_Sheet_1.zip › Data Sheet 1/Figure S1.tif]
